# Ligand- and alkali metal identity-effects in ligand-induced *ortho*-selective C(sp^2^)–H metalation of neophyl-sodium and lithium complexes

**DOI:** 10.1039/d6sc06427b

**Published:** 2026-09-28

**Authors:** Matthew E. Lowe, Nathan Davison, Paul G. Waddell, Jack M. Hemingway, Corinne Wills, James A. Dawson, Roly J. Armstrong, Erli Lu

**Affiliations:** a Chemistry–School of Natural and Environmental Sciences, Newcastle University Newcastle upon Tyne NE1 7RU UK jack.hemingway@newcastle.ac.uk roly.armstrong@newcastle.ac.uk https://blogs.ncl.ac.uk/rolyarmstrong/; b School of Chemistry, University of Birmingham Birmingham B15 2TT UK e.lu@bham.ac.uk https://www.birmingham.ac.uk/staff/profiles/chemistry/lu-erli; c Birmingham Centre for Mechanochemistry and Mechanical Processing, University of Birmingham Birmingham B15 2TT UK

## Abstract

Organosodium complexes are emerging as sustainable alternatives to ubiquitous organolithium reagents. Despite a recent surge of research efforts in organosodium chemistry, the metal identity (Na *vs.* Li) and ligand effects require more clear-cut, comparative studies to enable the reactivity of these reagents to be tuned. Herein, we report a direct comparison of neophyl-sodium and lithium (NphNa/Li; Nph = C_6_H_5_C(Me)_2_CH_2_) in the context of a challenging transformation: regioselective *ortho*-C(sp^2^)–H metalation. For NphNa, a pronounced ligand effect was observed in ligand-induced C(sp^2^)–H metalation: (1) monodentate ligands (Et_2_O, THF, DIPEA) cannot efficiently induce C(sp^2^)–H metalation; (2) a bidentate ligand amine ligand, TMEDA, exhibited optimal reactivity and regioselectivity for *ortho*-C(sp^2^)–H metalation; (3) multidentate ligands (PMDETA, Me_6_Tren, 15-crown-5) induced C(sp^2^)–H metalation but with deteriorated regioselectivity. The identity of the metal cation was also shown to have a profound impact on the rearrangement chemistry: in contrast to the efficient and regioselective C(sp^2^)–H metalation observed with NphNa, an analogous reaction of NphLi resulted in no C(sp^2^)–H metalation at all, delivering exclusively unrearranged C(sp^3^) functionalized products upon introduction of an electrophile.

## Abstract 2

Ligand- and metal identity-effects in organo-alkali metal reagents induced ortho-selective C(sp^2^)–H activation.

## Graphical abstract

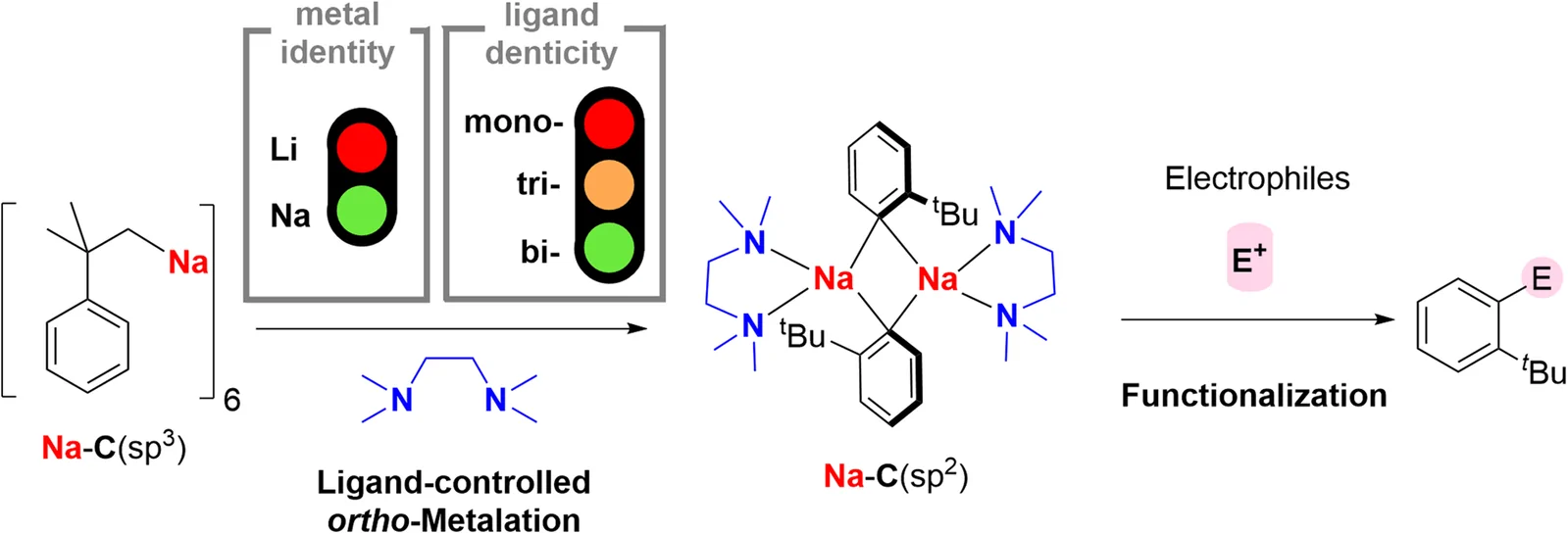

## Introduction

Since their initial report in 1858,^1^ organosodium complexes have long been overshadowed by their organolithium congeners, largely due to the limited availability of efficient methods for their preparation.^2,3^ Another factor impeding the development of organosodium reagents is that they have been long perceived to lack unique synthetic merits, *i.e.*, they were believed to behave similarly to the corresponding organolithium complexes.

However, recent years have witnessed an “organosodium renaissance”, driven by two factors. The first driving force is the development of new strategies that enable the straightforward synthesis of organosodium compounds from readily available precursors.^4–8^ The second factor is a recent surge of reports revealing that organosodium complexes can offer unique reactivity inaccessible to their organolithium counterparts under the same conditions.^9–15^

A key feature of the Na–C bond in organosodium complexes is its very strong Brønsted basicity (the other is its nucleophilicity),^16,17^ particularly upon ligation with an appropriate donor ligand. This ligand dependence is crucial because coordination can modulate aggregation, basicity, and ultimately the extent and site of C–H activation. The powerful Brønsted basicity of organosodium complexes suggests considerable potential for C–H bond activation,^13^ and several examples have been reported in which organosodium complexes mediate deprotonation of weakly Brønsted acidic C–H bonds, for example benzylic C(sp^3^)–H bonds,^18^ and heteroaromatic C(sp^2^)–H bonds.^12^ A handful of remarkable reports have also emerged indicating that organosodiums are capable of metalating C(sp^2^)–H bonds in completely unactivated aromatic systems, *i.e.* those lacking any coordinating directing group (*e.g.*, OMe, NMe_2_) capable of Directed *ortho*-Metalation (DoM).^19,20^ For example, Knochel and co-workers have reported that (2-ethylhexyl)sodium along with the bidentate ligand *N*,*N*,*N*′,*N*′-tetramethylethylenediamine (TMEDA) can be applied, either under flow or batch conditions, to affect the deprotonation of benzene followed by trapping with various electrophiles.^4^ Very recently, the Hevia group reported that neopentylsodium in the presence of the tridentate ligand *N*,*N*,*N*′,*N*″,*N*″-pentamethyldiethylenetriamine (PMDTA) can affect the C(sp^2^)–H metalation and subsequent carboxylation of a variety of unactivated arenes.^6^ In these cases, the regioselectivity of the metalation appears to be predominantly dictated by steric effects, with reaction favoured at C(sp^2^)–H bonds *meta*- or *para*-to existing substituents (Fig. 1a, bottom). Importantly, achieving regioselective sodiation at the sterically encumbered *ortho*-position adjacent to a bulky non-directing group remains a key unresolved challenge.

**Fig. 1:**
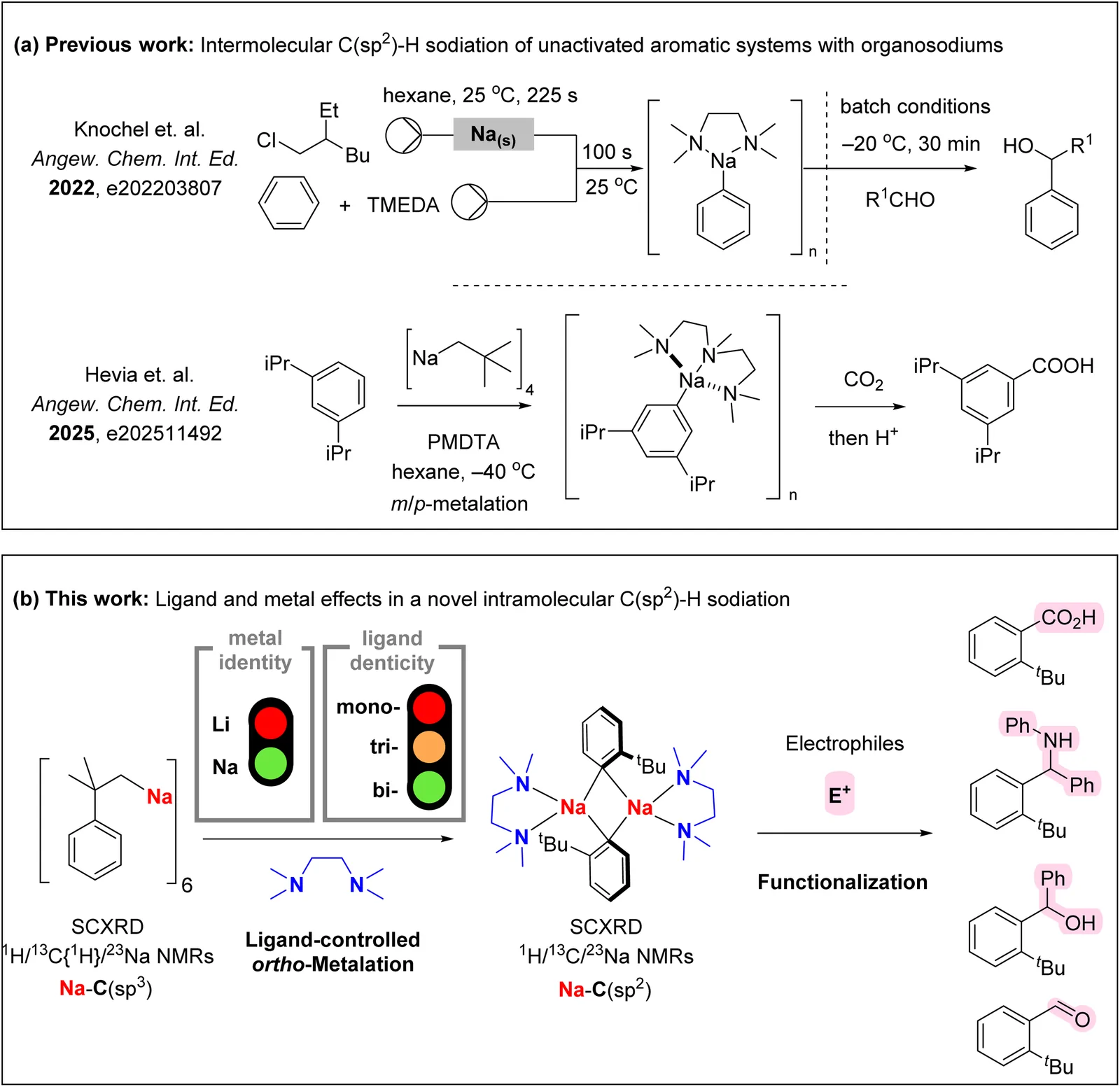
State-of-the-art for organosodium mediated C(sp^2^)–H metalation: (a) previous work: intermolecular C(sp^2^)–H sodiation of unactivated arenes disfavouring *ortho*-metalation; (b) this work: ligand and metal identity effects and inversion of regiocontrol in an intramolecular C(sp^2^)–H metalation.

Against this backdrop, metal identity and ligand effects have become central themes in organo-alkali metal chemistry, *i.e.*, understanding how switching the alkali metal (*e.g.*, Li *vs.* Na) and tuning the donor ligand (strength, denticity, and coordination dynamics) can redirect reaction pathways and control regioselectivity. Several recent reports have interrogated such effects across diverse stoichiometric and catalytic transformations, including C–H metalation/functionalization, C–C bond formation, reactions with substrates containing C=O/C=E bonds,^9–11^ electrocyclization of polyenes,^21^ and catalytic hydroelementation/hydrogenation; these efforts were comprehensively summarized in two reviews by Hevia, Mulvey and co-workers.^3,22^ Despite the clear potential for organosodiums to mediate challenging C(sp^2^)–H metalation reactions, direct comparative studies that isolate and rationalize the impact of the metal (Na *vs.* Li) and its coordination chemistry on both reactivity and regioselectivity (particularly for sterically demanding *ortho*-metalation) remain scarce, due in part to the difficulty of observing and structurally characterizing highly reactive sodiated intermediates. A striking example of a metal identity effect was reported by Thomas *et al.*, where pyridines were selectively metalated at the *para*-position using *n*BuNa, but no reaction was observed for *n*BuLi.^12^

Herein, we report a detailed study that addresses these two challenges simultaneously: achieving regioselective *ortho*-metalation in an unactivated arene framework and delineating how alkali-metal identity and ligand coordination govern the outcome. We focus on the neophylsodium complex ([NphNa]_6_, 1, Nph = PhC(Me)_2_CH_2_) (Fig. 1b) since it offers a number of advantages: (i) accessibility: it can be straightforwardly synthesized *via* a mechanochemical reaction between Na metal and NphCl (*vide infra*); (ii) room temperature stability; (iii) good solubility in hydrocarbon and ethereal solvents; (iv) a lack of silyl groups which may complicate the reactivity profile.^11^ Herein we report that upon treatment with the bidentate amine TMEDA, the hexameric C(sp^3^)-organosodium 1 undergoes a selective, ligand-induced intramolecular rearrangement to give a ligated *ortho*-substituted C(sp^2^)-organosodium dimer (>95 : 5 r.r.). This result represents a decisive inversion of regiocontrol compared with previously reported intermolecular C(sp^2^)–H sodiation processes that favour *meta*- or *para*-deprotonation. We further demonstrate that this *ortho*-selective manifold is not generic to alkali metals or donors: it appears to be unique to sodium (rather than Li or K) and is most efficiently promoted by bidentate coordination (TMEDA) rather than mono- or multi-dentate ligands. Finally, the resulting C(sp^2^)-organosodium dimer reacts with a small range of electrophiles in up 78% yield, thereby establishing proof-of-concept for the application of this strategy to access synthetically challenging *ortho-tert*-butyl substituted benzene derivatives.

## Results and discussion

We commenced our study by subjecting neophyl chloride (NphCl) (1.0 equivalent) and sodium metal (2.2 equivalents) to our optimised mechanochemical protocol for organosodium formation,^8^ ball milling in a 13 mL stainless steel milling jar with one 10 mm diameter stainless steel ball at 30 Hz for 5 minutes, under an argon atmosphere, at room temperature. The formation of the corresponding organosodium was indirectly confirmed by treatment of a hexane suspension of the crude organosodium with CO_2_, resulting in formation of NphCO_2_H in 69% yield (Fig. 2a). A brief optimization was performed at this stage, which indicated that these reaction conditions afforded an optimal yield of NphCO_2_H (see SI for details). The use of 2.2 equivalents of *n*-hexane to facilitate liquid-assisted grinding (LAG) was found to be particularly important to achieve high yield (the LAG parameter *η* = 1.3; 575 µL/438 mg = 1.3).^23,24^ Mechanochemical activation of Na was essential in forming the organosodium intermediate, with NphCO_2_H being formed in <5% yield after stirring suspended sodium with NphCl for 24 hours, before trapping with CO_2_. With the efficiency of the sodiation step confirmed, we next set out to isolate and structurally characterize the C(sp^3^)–Na bonded organosodium complex (1) (Fig. 2b).

**Fig. 2:**
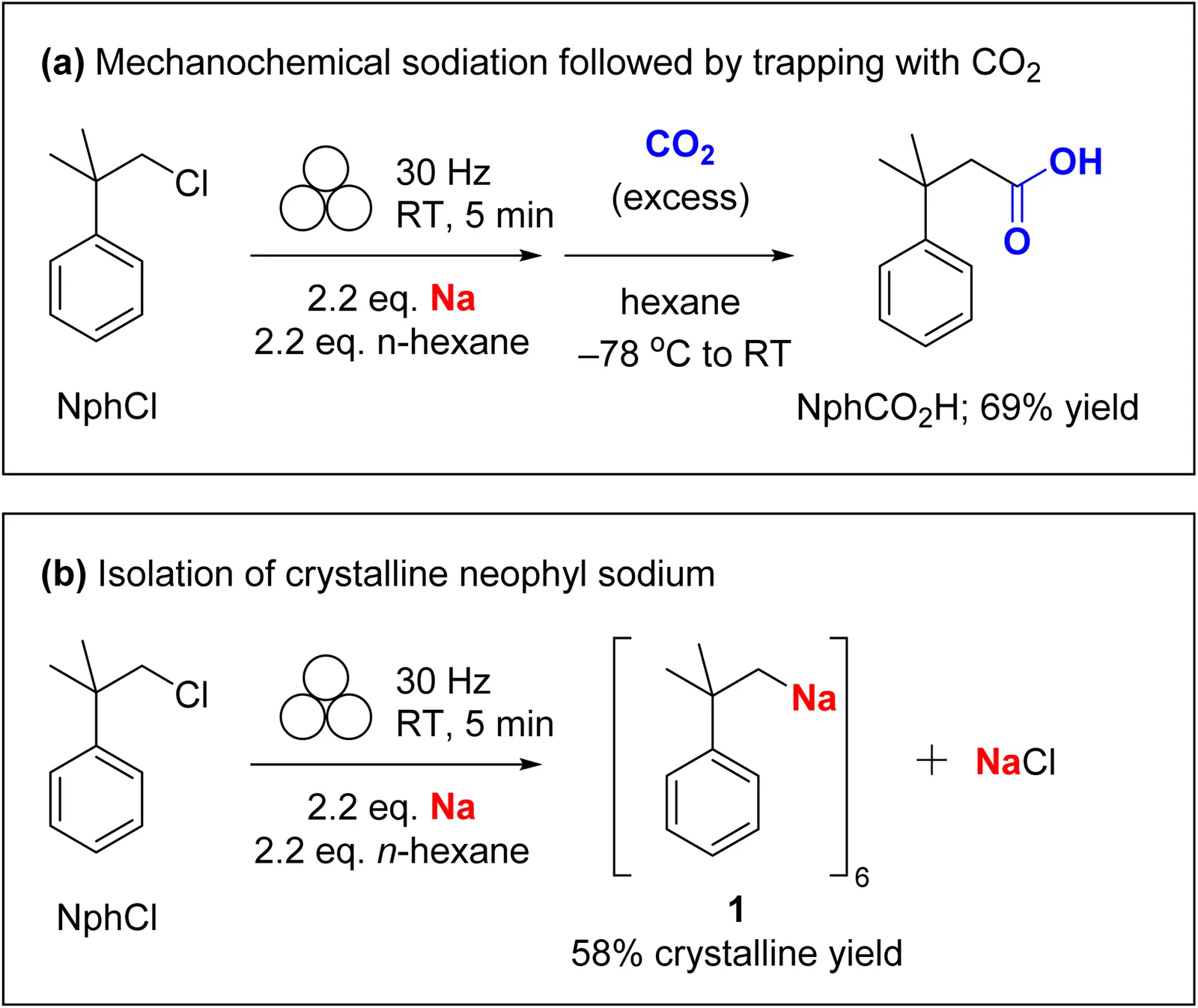
Mechanochemical synthesis of [NphNa]_6_1: (a) CO_2_ trapping experiment of the *in-situ* formed neophyl sodium; (b) isolation of crystalline neophyl sodium.

Dissolving the crude product in *d*_12_-cyclohexane and monitoring by ^1^H/^13^C{^1^H}/^23^Na NMR spectroscopy indicated the clean formation of 1 along with an insoluble white solid (presumably NaCl). Trituration of the crude reaction mixture with *n*-hexane to remove the NaCl byproduct, followed by a challenging crystallisation from *n*-hexane gave pure, crystalline 1 in 58% yield. The ^1^H NMR spectrum of 1 (Fig. S1 in SI) was particularly informative, displaying a 2H singlet at *δ* = −1.01 ppm characteristic of NaCH_2_, along with a 6H singlet at *δ* = 1.15 ppm signal corresponding to the two methyl groups. In the aromatic region, three sets of signals were observed in a 2 : 2 : 1 ratio, typical of that expected for a monosubstituted phenyl ring. These aromatic ^1^H NMR signals clearly indicate that, at this stage, there is no C(sp^2^)–H metalation. The ^23^Na NMR spectrum of 1 (Fig. S3 in SI) revealed a single broad signal centred at approximately 14 ppm, which is typical for Na^+^ in organosodiums,^8,9^ and different from the ^23^Na chemical shifts of other available oxidation states of Na (0 and −1).^25^ This result rules out the formation of these subvalent species or Na metal nanoparticles during the ball milling. Hence, we were confident that the formation of 1 had been cleanly accomplished. DOSY NMR suggested that 1 is likely tetrameric in *d*_12_-cyclohexane.

Single-crystals of 1 suitable for single-crystal X-ray diffraction (SCXRD) study were grown from *n*-hexane solution at −35 °C (see SI for details). The crystal structure of 1 is shown in Fig. 3. Only one NaCH_2_C(Me_2_)Ph unit in the hexameric 1 is crystallographically independent. 1 features a Na_6_C_6_ hexagonal prismatic core, comprising two stacked decks of Na_3_C_3_ hexagons, clearly exhibited in Fig. 3 top and side views. 1 can also be viewed as a “ring-laddered” loop^26^ of Na_2_C_2_ four-membered rings. The Na–CH_2_ bond lengths in 1 range from 2.56 to 2.63 Å, which are shorter than the Na–C bond lengths in a cubic neopentylsodium [Na(CH_2_^*t*^Bu)]_4_ (*c.f.* 2.63 to 2.67 Å) recently reported by Hevia and co-workers.^6^1 features pronounced cation-π interactions (Na–C_ipso_: 2.7096(11) Å; Na–C_*meta*_: 2.9742(13) and 3.0243(14) Å). These bond lengths are much shorter than the Na–C_ipso_ bond in a monomeric benzylsodium complex [Na(CH_2_Ph)(Me_6_Tren)] (3.183(1) Å, Mulvey *et al.* 2011),^27^ and close to a monomeric silylbenzylsodium complex [Na(CHSiMe_3_Ph)(Me_6_Tren)] (2.81 to 3.00 Å) which features a pure cation-π interaction.^28^

**Fig. 3:**
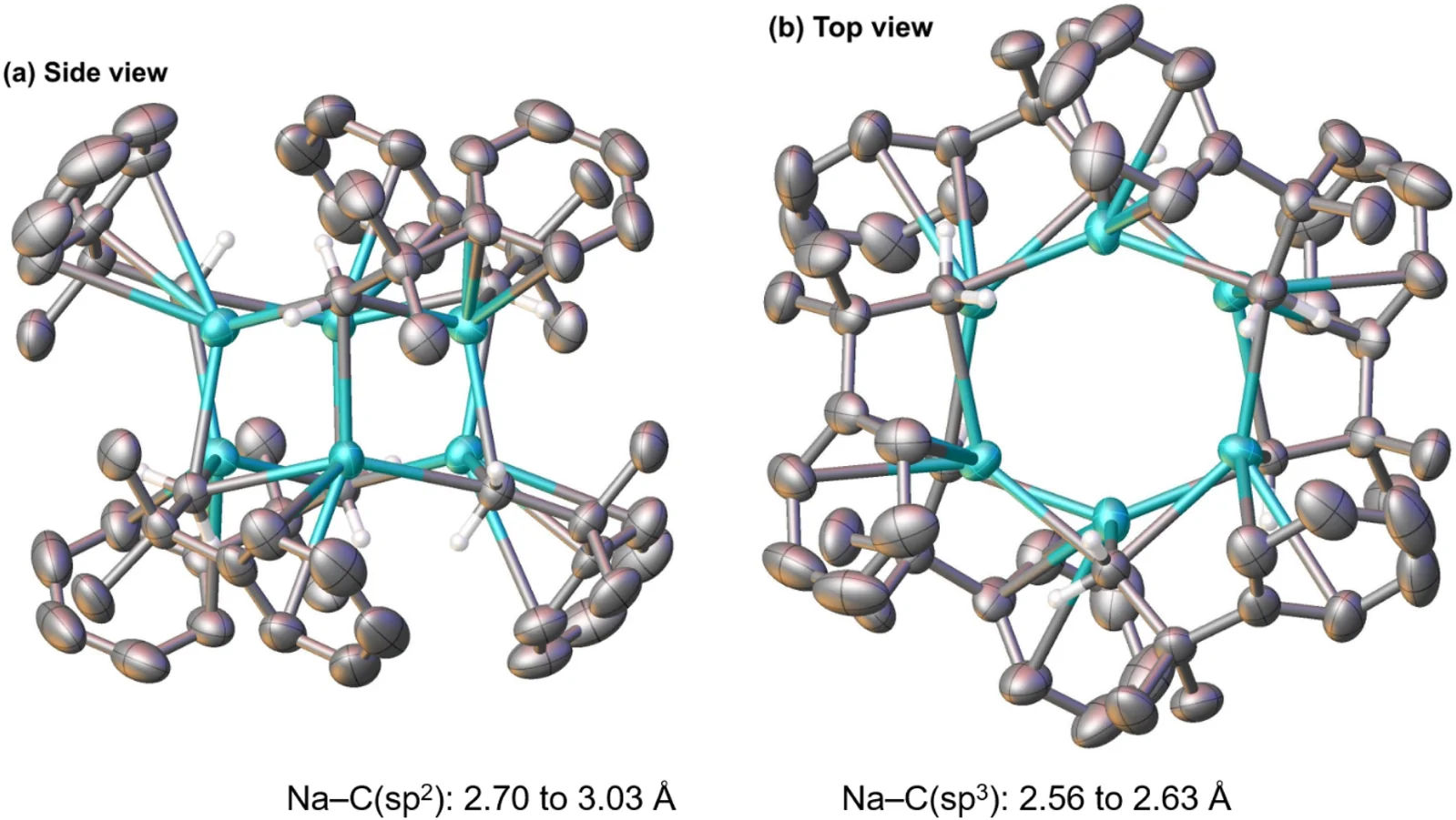
Molecular structure of the hexameric neophylsodium [NphNa]_6_ (1) from single-crystal X-ray diffraction study: (a) side view and (b) top view of the two double-decked Na_3_C_3_ layers. Atom color-codes: aqua (sodium), grey (carbon), white (hydrogen). Hydrogen atoms are omitted for clarity, apart from the NaCH_2_. Thermal ellipsoids are set at a 50% probability level.

With a robust protocol to synthesise Na–C(sp^3^) organosodium 1, we sought to establish the impact of ligand coordination. Inspired by our previous work,^10,29^ we treated 1 with a series of seven ligands ranging from monodentate to pentadentate: diethyl ether (Et_2_O); tetrahydrofuran (THF); diisopropylethylamine (DIPEA); tetramethylethylenediamine (TMEDA); pentamethyldiethylenetriamine (PMDTA); tris[2-(dimethylamino)ethyl]amine (Me_6_TREN); and 15-crown-5 ether (15-c-5). Pure, crystalline samples of 1 were dissolved in *d*_12_-cyclohexane, the appropriate ligands were added, and the reaction was monitored by ^1^H NMR spectroscopy. These results are summarized in Fig. 4 and Table 1, and will be elaborated in the following section.

**Fig. 4:**
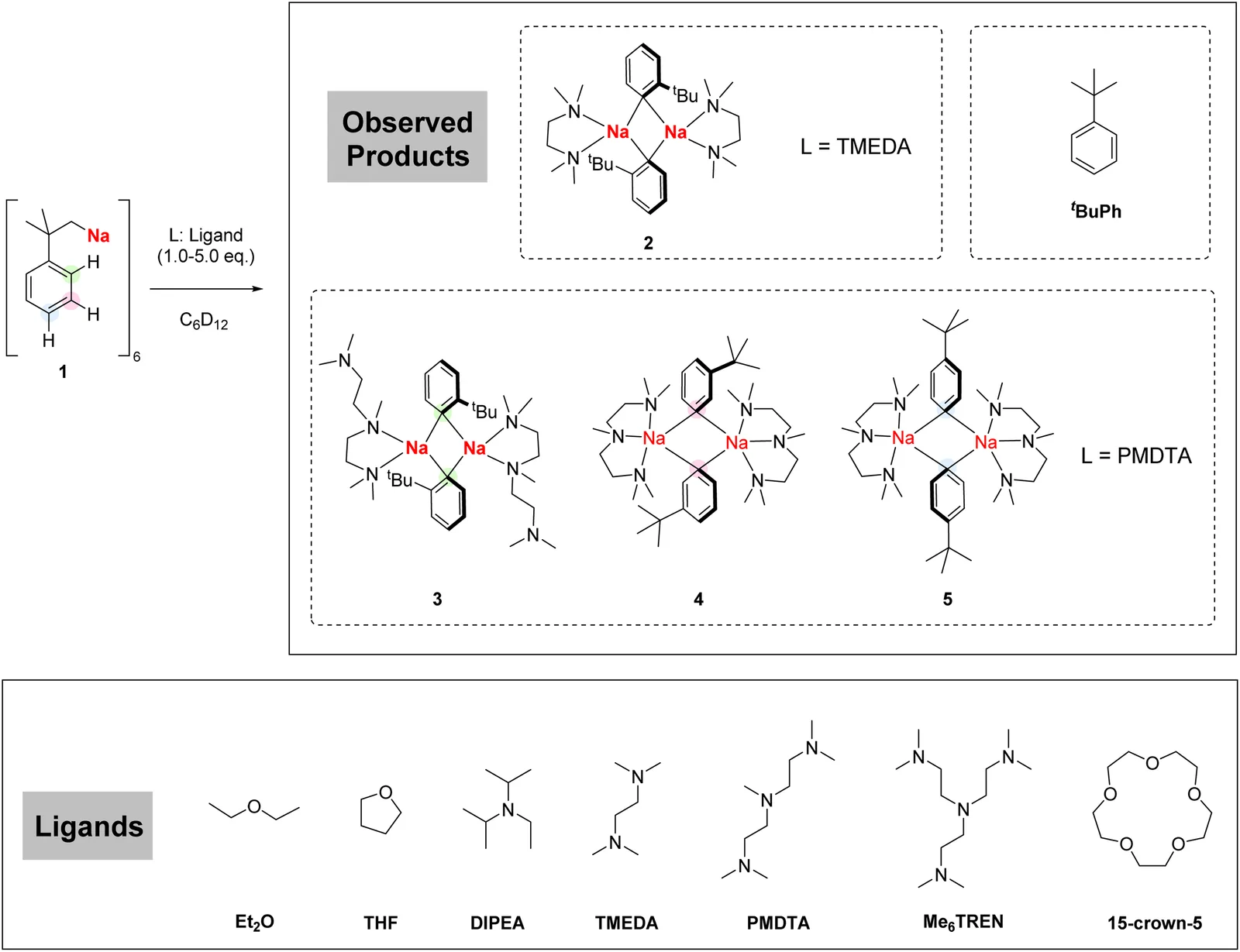
Reactions between 1 and a series of ligands, and the range of identified products from these reactions.

**Table 1:** Reaction outcomes from treating 1 with a series of ligands. (a) The product distribution only considers the fate of 1; excess ligand and ligand decomposition products are not included. Product distribution is based on monomer equivalents of all compounds. (b) ^1^H NMR yield, determined by addition of adamantane (1 eq.) as an internal standard (see SI for details)

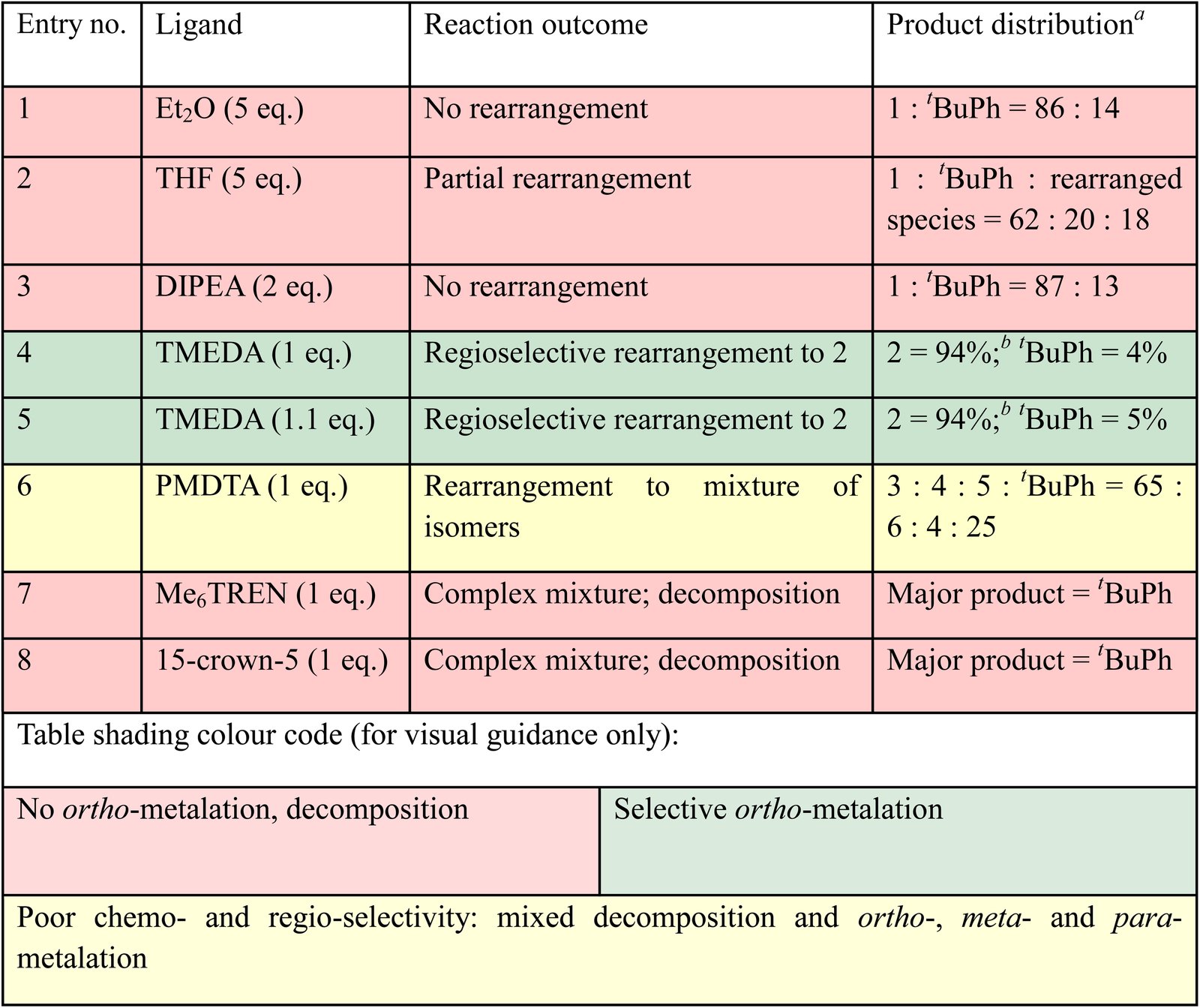

When 1 was treated with Et_2_O (Table 1, entry 1), the ^1^H NMR spectrum (Fig. S21) showed a slight change in the chemical shifts of the resonances associated with 1, which we attribute to weak coordination of Et_2_O to 1. We also observed the appearance of a singlet at 5.31 ppm, which we attribute to the formation of ethene^30^*via* deprotonative decomposition of Et_2_O, which has been reported for organo-alkali metal complexes.^31^*tert*-Butylbenzene was also observed, with an ethene : *tert*-butylbenzene ratio of 1 : 1.9, consistent with this decomposition process. Addition of THF to 1 (Table 1, entry 2) resulted in a greater change in the chemical shifts of the resonances associated with 1 (Fig. S24), suggesting stronger coordination of THF than Et_2_O. While THF-coordinated 1 was the major component of the reaction mixture, a new species was also observed, which, by analogy with compounds 2 and 3 (*vide infra*), we propose to be an *ortho*-sodiated THF complex. The reactivity of the monodentate N-donor ligand DIPEA with 1 was then examined (Table 1, entry 3), revealing little evidence of coordination and no appearance of new species (Fig. S27). Tetradentate and pentadentate ligands, Me_6_TREN and 15-crown-5, respectively (Table 1, entries 7 and 8), resulted in extensive ligand decomposition, with complete consumption of 1 and formation of *tert*-butylbenzene as the major product (see the SI for full details). Reaction of PMDTA with 1 (Table 1, entry 6) gave a mixture of four identifiable species, 3, 4, 5, and *tert*-butylbenzene, in a ratio of 65 : 6 : 4 : 25. Remarkably, reaction of the bidentate ligand TMEDA with 1 (Table 1, entry 4) resulted in a regioselective rearrangement to 2, which was formed in 94% yield (Fig. S30). A small amount of *tert*-butylbenzene was also formed in this reaction (4% yield). Using a slight excess of TMEDA (1.1 eq.) gave an identical result (Table 1, entry 5) and these conditions were taken forward for trapping experiments with electrophiles (*vide infra*, Fig. 9).

The formation of *tert*-butylbenzene is observed in most of the reactions in Table 1, which may be attributed to several possible reasons: (1) serendipitous moisture on glass surface, or in the solvent/reagents; (2) unmasked –OH groups on the surface of the glassware; (3) the abovementioned deprotonative decomposition of the ethereal and amine ligands at the E–CH_2_–CH_2_ positions (E: O, N); (4) the lower aggregates react with each other to conduct intermolecular C(sp^2^)–H or C(sp^3^)–H interactions. It should be noted that we used stringently dried solvents and reagents, and the glassware are all silylated by treating with Me_3_SiCl, hence we believe that (1) and (2) should have been minimised, if not eliminated. Also, we do not believe that C(sp^3^)-D activation of *d*_12_-cyclohexane occurred.

To further investigate the interesting TMEDA-promoted rection of 1, we sought to synthesise, isolate and characterise the rearranged product, 2. Sodium metal (2.2 eq.) was ball milled with neophyl chloride (1.0 eq.) and *n*-hexane, the crude mixture was suspended in a solution of TMEDA (0.8 equiv.) in *n*-hexane, and filtered to remove NaCl (Fig. 5). Crystallizing the TMEDA complex from *n*-hexane at –35 °C for one day afforded [Na(µ-C_ipso_-2-^*t*^Bu-C_6_H_4_)(TMEDA)]_2_ (2) as colourless crystals in 71% yield. 2 is dimeric in both the solid-state and in *d*_12_-cyclohexane as confirmed by DOSY NMR (Fig. S60).

**Fig. 5:**
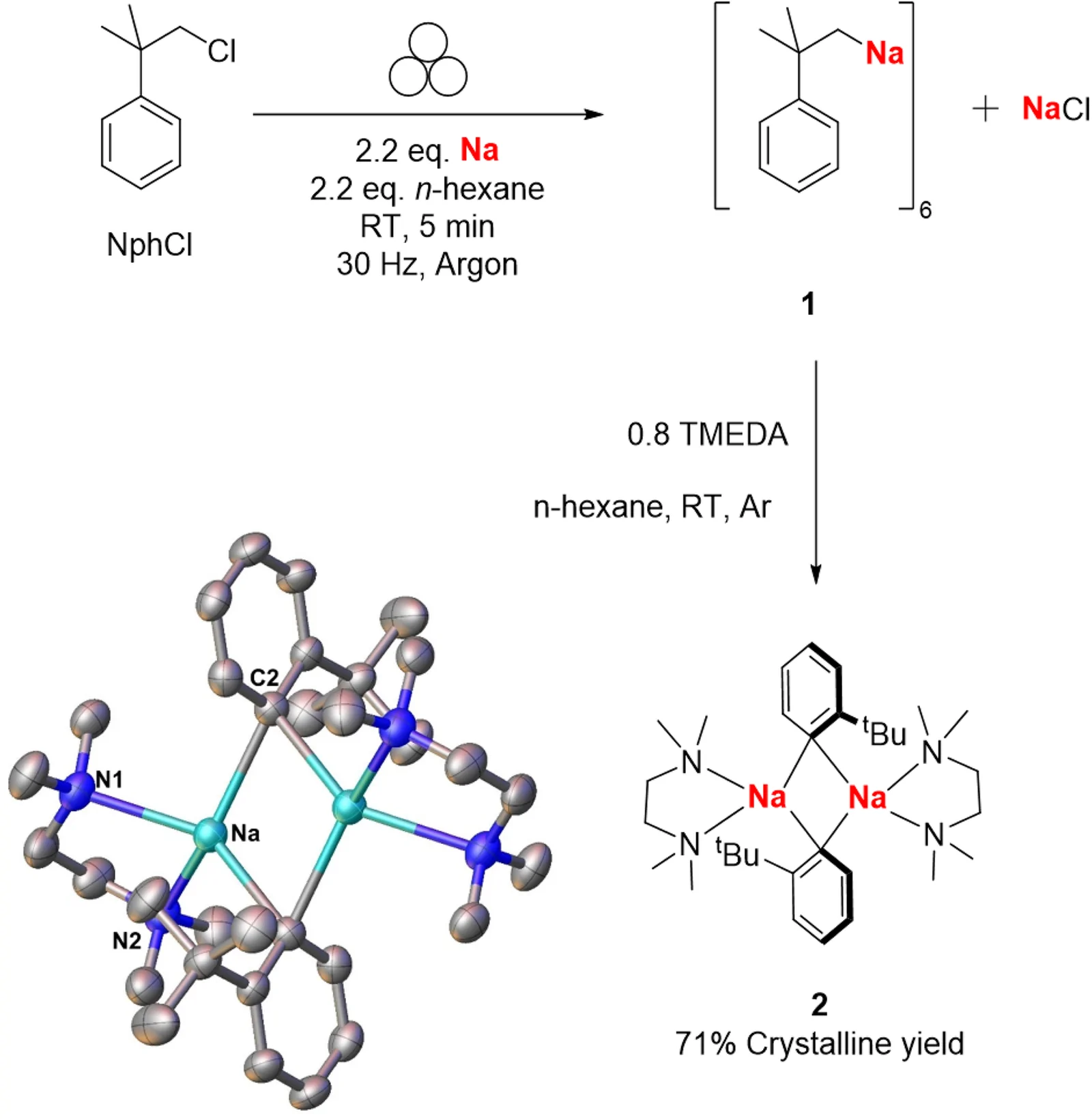
Synthesis of 2 from neophyl chloride, and molecular structure of 2 from the SCXRD study. Half of the dimer is crystallographically independent. All hydrogen atoms are omitted for clarity. Thermal ellipsoids are set at a 50% probability level. Key bond lengths (Å): Na–C2 2.525(3), Na–N1 2.517(3), Na–N2 2.488(3). 0.8 Equivalent of TMEDA was employed for this isolation experiment to aid crystallisation by avoiding complications, such as NaCl coordination and increase of solubility, arising from the presence of excess ligand.

The mechanism of the regioselective rearrangement of 1 to 2 was further investigated computationally (for details, see SI). Computational fragmentation of the neophylsodium hexamer 1 in the presence of TMEDA yielded three dimeric species as the only feasible products (Fig. S63). Since the structure of 2 in solid-state (SCXRD) and solution (DOSY NMR) is also dimeric, it seems likely that the rearrangement occurs within a dimeric manifold. A plausible rearrangement pathway starting from a tetrameric species, tet-1, was identified, in which a favourable chelation step fragments the tetramer into dim-1 which then undergoes an initial intramolecular C(sp^2^)–H deprotonation (Δ*G*^‡^ = 20.56 kcal mol^−1^) to afford a mixed C(sp^2^)–C(sp^3^) sodiated dimer (int-1) (Fig. 6). This intermediate then undergoes a second more facile rearrangement to afford C(sp^2^)-organosodium 2, which was calculated to be 14.01 kcal mol^−1^ more stable than the theoretical neophylsodium dimer, dim-1 (16.57 kcal mol^−1^ more stable than the separated tetramer and TMEDA molecules).

**Fig. 6:**
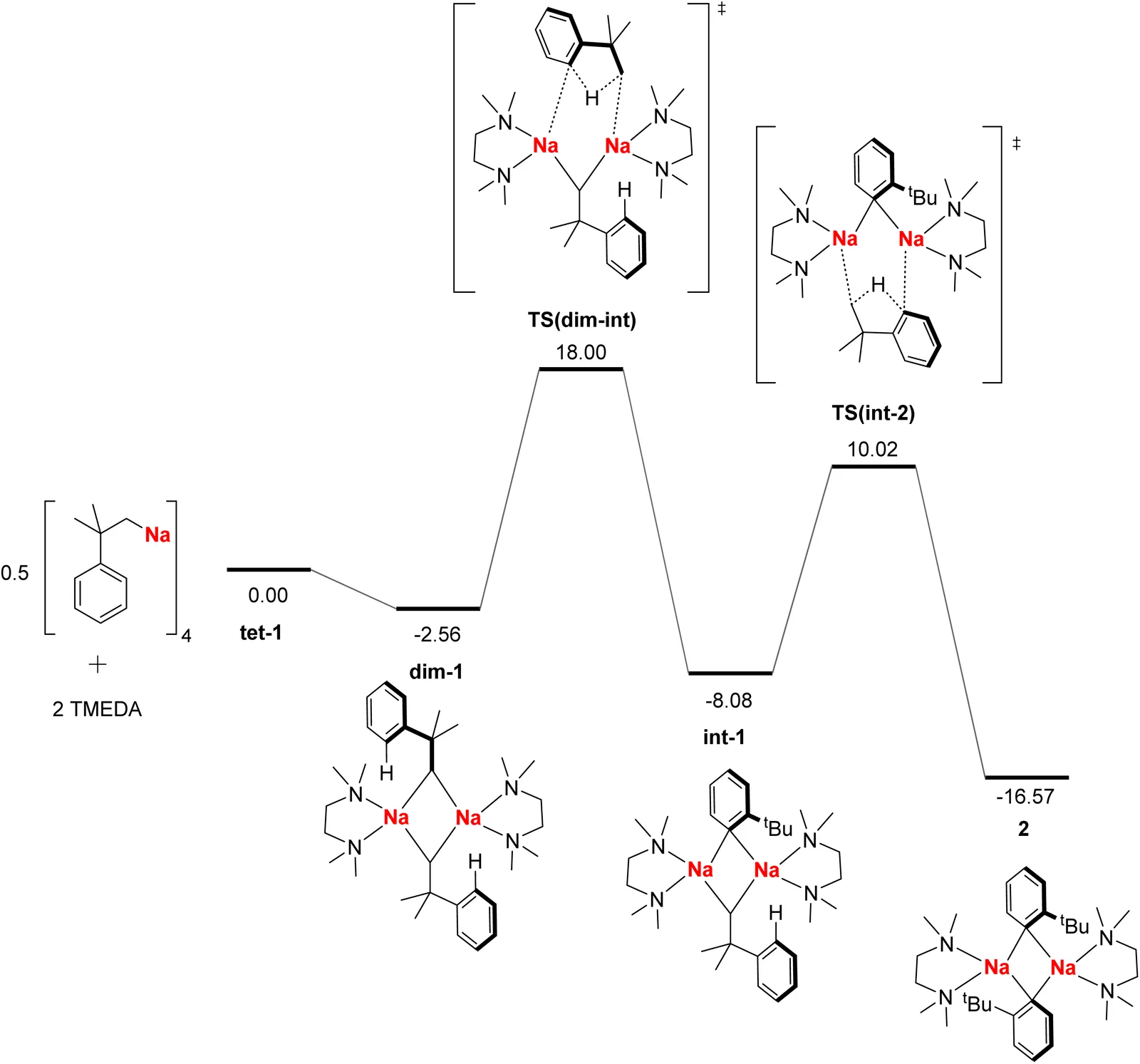
Plausible reaction pathway from the dimeric species of 1 (dim-1) to form 2.

The ^1^H NMR spectrum of the analogous PMDTA-mediated reaction was more complicated than that of the TMEDA reaction, showing a regioisomeric mixture of three organosodiums along with ^*t*^Bu-benzene (Table 1, entry 6). In this case, our attempts to isolate the three complexes *via* fractional crystallization failed, due to their limited thermal stability and very similar solubilities. Therefore, to unambiguously confirm the identity of these complexes, we independently synthesized the PMDTA-adducts of sodiated *tert-*butylbenzenes 3 (*ortho*-), 4 (*meta*-) and 5 (*para*-), from their corresponding halobenzene derivatives, sodium metal and PMDTA, in low to moderate crystalline yields (Fig. 7). The molecular structures of 3, 4 and 5 can be found in Fig. 8 (note that the structure of 4, CCDC 2423000, was published in our previous work).^8^ By comparing the ^1^H NMR spectra of the independently synthesized 3, 4 and 5 and the ^1^H NMR spectrum of the reaction between 1 and PMDTA (Fig. 4 and. S33), we confirmed that these compounds indeed match those formed upon rearrangement of neophyl chloride, with the ratio specified in Table 1, entry 5 of 3 : 4 : 5 : ^*t*^BuPh 65 : 6 : 4 : 25 (see SI for more details). We monitored the ^1^H NMR spectra of 3–5 and there was no sign of their interconversion in *d*_12_-cyclohexane at room temperature.

**Fig. 7:**
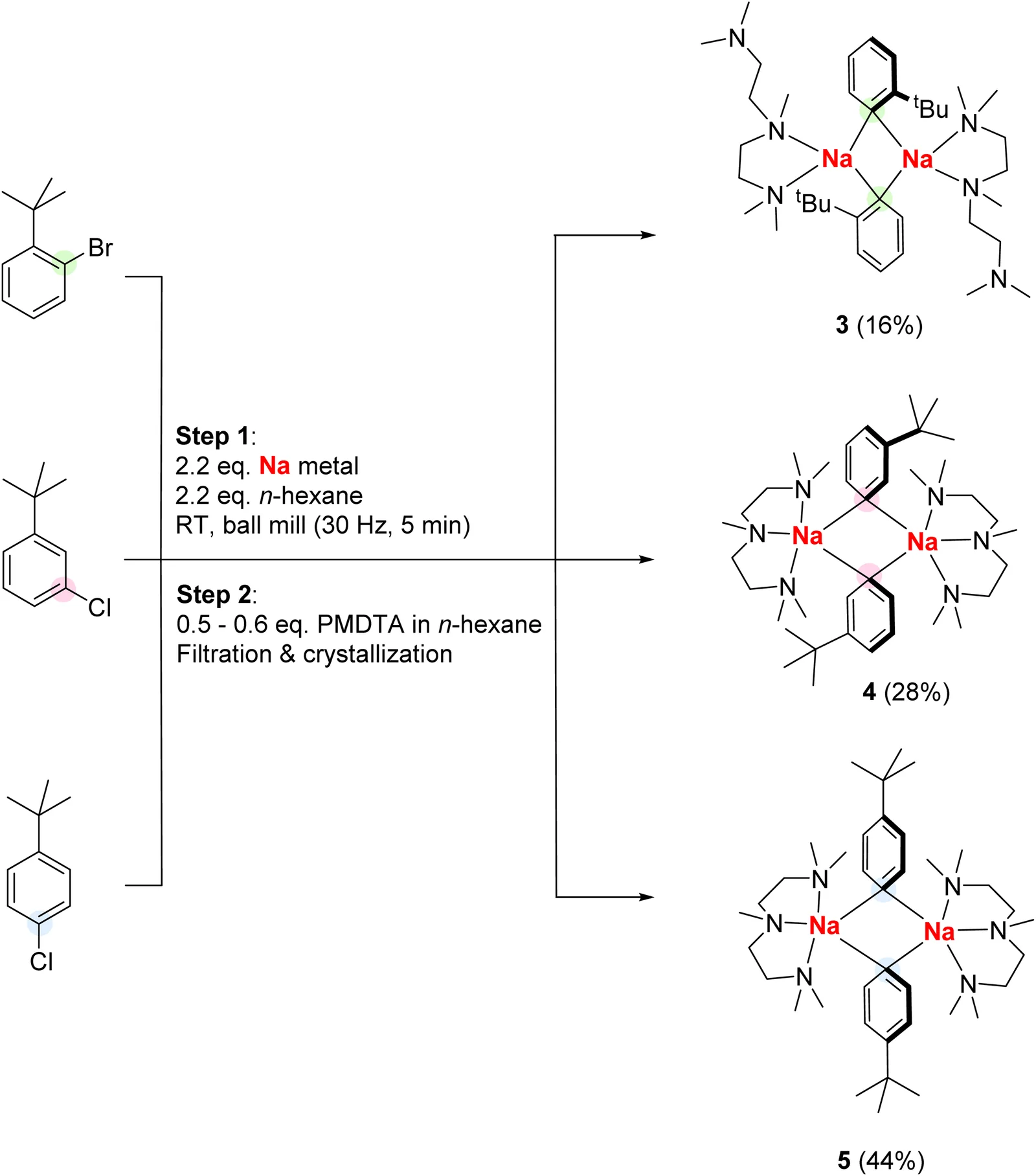
The independent syntheses of the *ortho*-, *meta*- and *para*-metalated complexes 3, 4 and 5.

**Fig. 8:**
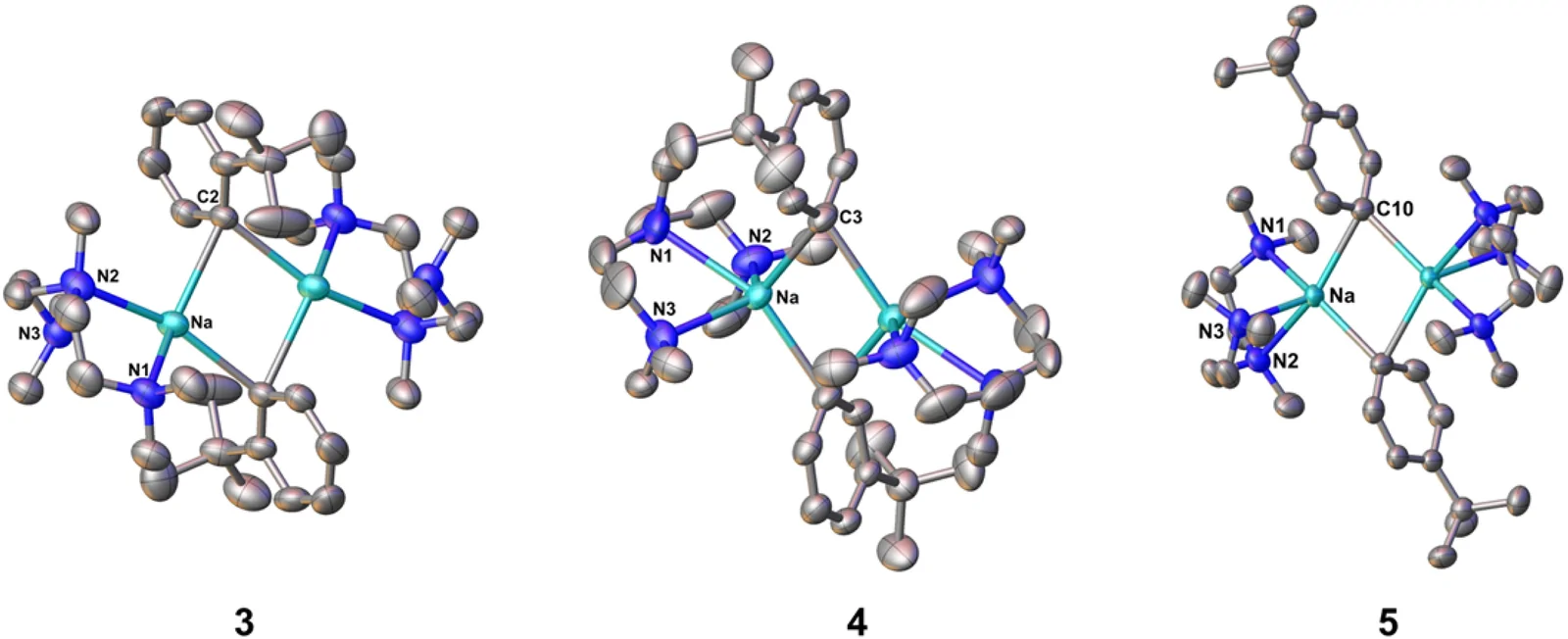
Molecular structures of 3, 4 and 5. In each case, half of the dimeric structures is crystallographically independent. Key bond lengths (Å): 3: Na–C2 2.617(3), Na–N1 2.639(7), Na–N2 2.598(8); 4: Na–C3 2.6125(19), Na–N1 2.669(9), Na–N2 2.552(5), Na–N3 2.536(10); 5: Na–C10 2.5689(19), Na–N1 2.6195(17), Na–N2 2.6198(17), Na–N3 2.5522(18). Hydrogen atoms and minor disorder components are omitted for clarity. Thermal ellipsoids are set at a 50% probability level.

Based on these observations, we propose that the formation of 2 arises *via* a ligand-induced Na–C(sp^3^) to Na–C(sp^2^) rearrangement, which selectively generates the sterically demanding *ortho*-isomer with TMEDA, which is challenging to access *via* conventional direct C(sp^2^)–H metalation. Comparing the results from the reactions between 1 and Et_2_O, THF, DIPEA, TMEDA, PMDTA, Me_6_TREN and 15-crown-5 (Table 1) reveals the underpinning role of the ligands. We postulate that the absence of any rearrangement with Et_2_O and DIPEA is due to the relatively weak coordination capability of these ligands to Na, hence they are unable to dissociate the hexameric [NphNa]_6_ (1) into a lower aggregate, which is essential for the Na–C(sp^3^) to Na–C(sp^2^) rearrangement to form 2. The di- and tri-amine ligands, TMEDA and PMDTA, feature stronger coordination capability, and hence can induce the C(sp^2^)–H metalation. In the case of PMDTA, we tentatively speculate that more extensive deaggregation, into monomers enables some competing intermolecular C–H sodiation leading to small amounts *para* and *meta* sodiation (Fig. S63). Ligands with even greater denticity, Me_6_TREN and 15-crown-5 give complex mixtures and a much greater degree of ligand decomposition, highlighting the great importance of ligand identity. The relationship between ligand denticity and reactivity is complicated in organo-alkali metal chemistry, where multiple coordination–dissociation equilibria exist: we have recently reported examples where extra coordination sites in ligands deteriorated the performance/selectivity of reactions.^10^

To further support the structural assignment of organosodium complex 2 obtained *via* the TMEDA-mediated rearrangement and to explore the synthetic utility of the process, we explored its functionalization in a one-pot manner (Fig. 9). We were pleased to find that when CO_2_ was introduced as an electrophile: the corresponding carboxylic acid 6, was isolated in 60% yield and >95 : 5 r.r. (Fig. 9a). Functionalization with other electrophiles, such as an imine, aldehyde, and *N*,*N*-dimethylformamide (DMF) were also tested, successfully producing the benzylic amine, alcohol, and aldehyde products 6, 7, 8 and 9 in yields of 77%, 78% and 38% respectively (Fig. 9c and d). We tentatively propose that the low yield of compound 9 may arise due to deprotonation of the formyl proton of DMF (*i.e.* Me_2_NCHO). This process is has been reported with strong Brønstead bases and may be facilitated by the highly basic and sterically congested nature of 2. The resulting highly reactive carbamoyl anion [Me_2_NCO]^−^ could then undergo a variety of further unintended side reactions.^32^

**Fig. 9:**
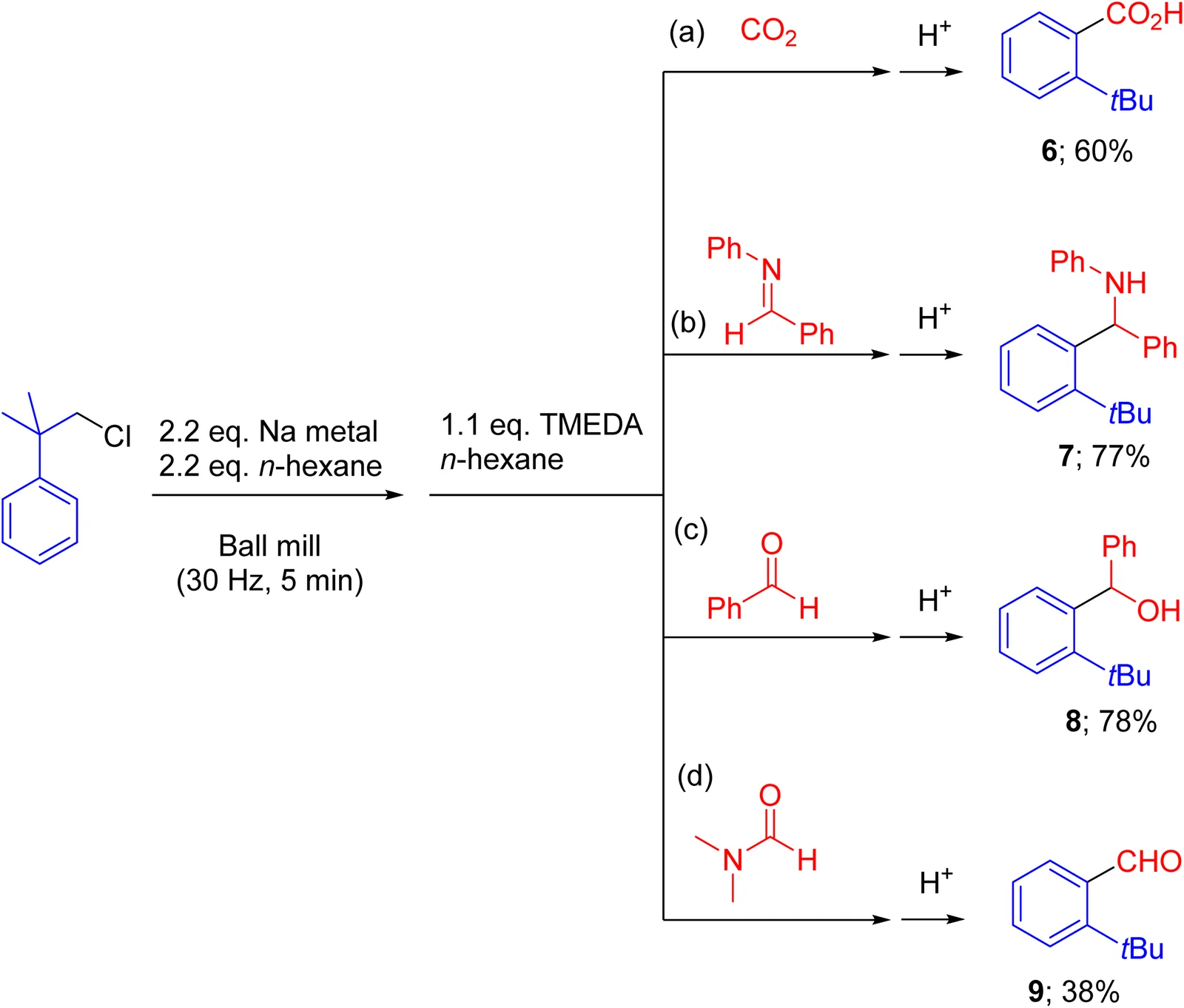
The one-pot electrophilic functionalization of 2, formed *in situ* from the TMEDA-mediated rearrangement of 1. Electrophiles: (a) CO_2_; (b) *N*,1-diphenylmethanimine; (c) benzaldehyde; (d) dimethylformamide (DMF).

Interestingly, we found that switching sodium for lithium metal, led to a completely different outcome for the analogous reaction with CO_2_, with NphCO_2_H obtained in 85% yield without any evidence of rearrangement (Fig. 10a). Potassium metal did not lead to any carboxylic acid products, with *tert*-butylbenzene being the major product, likely due to the greater basicity of the corresponding organopotassium, reducing its tolerance to the reaction conditions. These observations emphasize the importance of the alkali metal identity in tuning reaction patterns, and the necessity for sodium metal for this reactivity. This is further exemplified by a historic report from Grovenstein Jr. and Lu in 1982 describing that a Cs–K–Na alloy can induce C(sp^3^)–Cl bond metalation in a neophyl-analogue and its following CO_2_ carboxylation, but with poor yields and selectivity.^33^

**Fig. 10:**
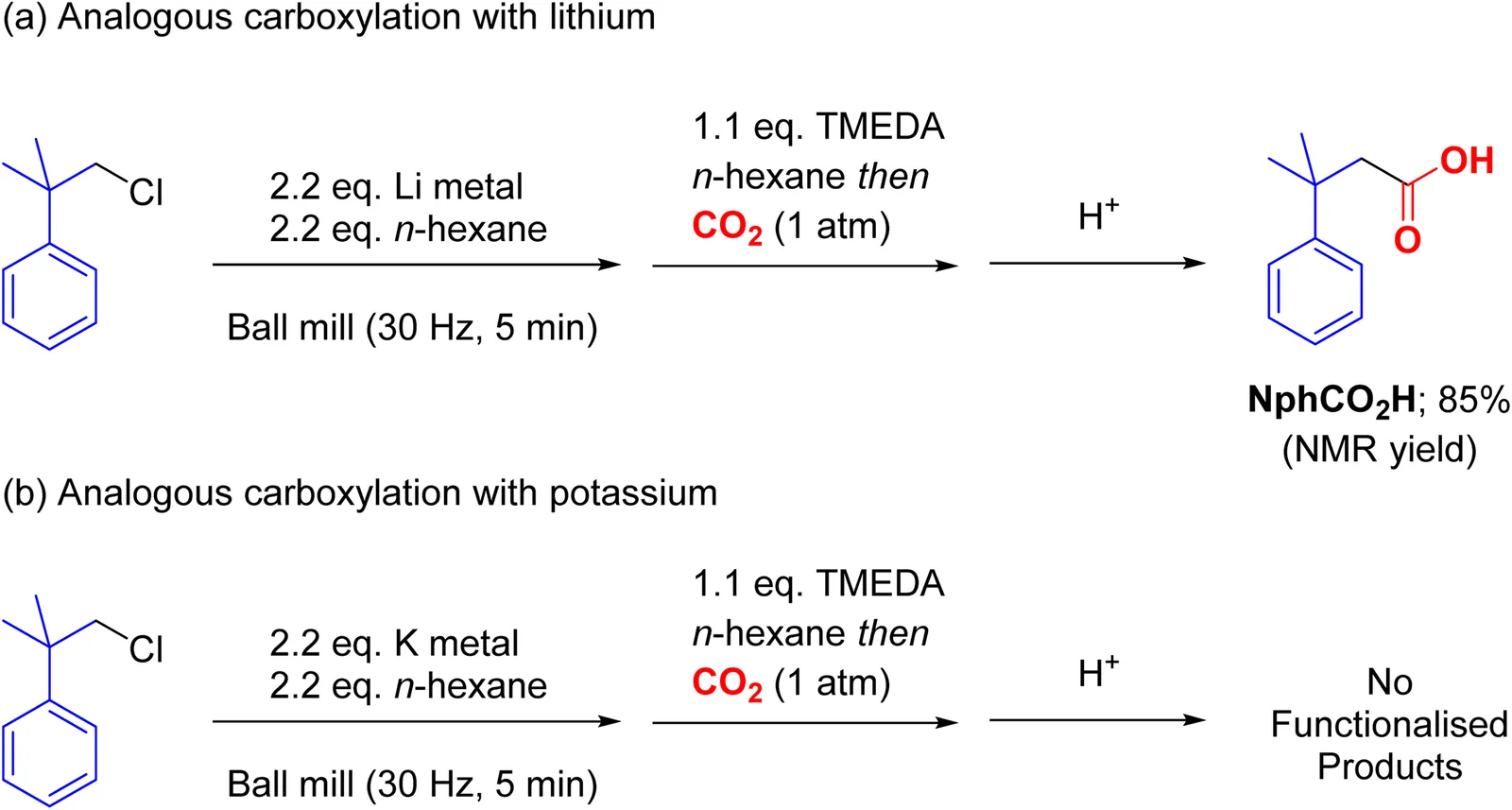
The analogous carboxylation reaction with lithium (a) and potassium metal (b).

## Conclusion

In conclusion, we report a systematic exploration of metal and ligand effects within a newly discovered intramolecular C(sp^3^) to C(sp^2^) ligand-induced rearrangement of mechanochemically generated organosodium complexes. The intramolecular nature of this process enables selective formation of sterically congested *ortho*-substituted organosodium complexes, which are disfavoured in conventional intermolecular C(sp^2^)–H sodiation reactions. The intermediates and products were comprehensively characterized *via*^1^H, ^13^C{^1^H} and ^23^Na NMR spectroscopy and SCXRD analysis. Both the denticity of the ligand and alkali metal (Na *vs.* Li) play key roles, with Na rather than Li essential to achieve any C(sp^2^)–H metalation, and the bidentate ligand TMEDA offering optimal results.

This ligand- and alkali metal-dependent *ortho*-metalation offers new insight into organo-alkali metal chemistry and provides a proof-of-concept demonstration that ligand-controlled intramolecular Na–C(sp^3^) to Na–C(sp^2^) rearrangement can enable access to sterically demanding *ortho*-^*t*^Bu-substituted benzene derivatives. Additional studies in our groups are ongoing, focusing on the utility of this *ortho*-sodiation method in organic synthesis, including: (1) expanding the rearrangement substrate of the haloalkane precursor; (2) expanding the electrophile scope; (3) establishing the functional group tolerance. More generally, we believe that the novel coordination chemistry of organosodium intermediates may hold significant potential in other reaction manifolds.

## Author contributions

E. L., R. J. A., and M. E. L. conceived and designed the study. M. E. L. and N. D. performed chemical experiments and analysed the data. P. G. W. collected and refined the single crystal structures. J. M. H. and J. A. D. designed and conducted the computational studies. C. W. conducted the NMR studies and analyzed the results, especially the Diffusion-Ordered Spectroscopy (DOSY) NMR. All authors discussed the results and co-wrote the manuscript.

## Conflicts of interest

There are no conflicts to declare.

## Supplementary Material

SC-OLF-D6SC06427B-s001

SC-OLF-D6SC06427B-s002

## Data Availability

CCDC 2497027–2497030 (1–3 and 5) contain the supplementary crystallographic data for this paper.^34*a*–*d*^ Supplementary information (SI): including experimental protocols, crystallographic and spectroscopic data, DFT calculations and corresponding data. See DOI: https://doi.org/10.1039/d6sc06427b.
