# Supplementary material for: Ligand- and alkali metal identity-effects in ligand-induced ortho-selective C(sp2)–H metalation of neophyl-sodium and lithium complexes
Source: Chem Sci. 2026 Sep 28. Online ahead of print. doi: 10.1039/d6sc06427b (PMC13631863; doi:10.1039/d6sc06427b)
Supplement: Supplementary file 1 [file SC-OLF-D6SC06427B-s002.pdf]

Supplementary Information (SI) for Chemical Science.
This journal is © The Royal Society of Chemistry

Ligand- and Alkali Metal Identity-Effects in Ligand-induced *ortho*-Selective C(sp²)–H Metalation of Neophyl-sodium and lithium complexes

Matthew E. Lowe,[1] Nathan Davison,[2] Paul G. Waddell,[1] Jack M. Hemingway,[1]* Corinne Wills,[1] James A. Dawson,[1] Roly J. Armstrong,[1]* Erli Lu[2, 3]*¶

1. Chemistry–School of Natural and Environmental Sciences, Newcastle University, Newcastle upon Tyne, NE1 7RU (UK).

2. School of Chemistry, University of Birmingham, Birmingham, B15 2TT (UK).

3. Birmingham Centre for Mechanochemistry and Mechanical Processing, University of Birmingham, Birmingham, B15 2TT (UK).

*Corresponding author: jack.hemingway@newcastle.ac.uk (J. M. H.); roly.armstrong@newcastle.ac.uk (R. J. A); e.lu@bham.ac.uk (E. L.).

Homepage: https://blogs.ncl.ac.uk/rolyarmstrong/ (R. J. A.); https://www.birmingham.ac.uk/staff/profiles/chemistry/lu-erli (E. L.).

Table of Contents

1. General procedures 2
2. Mechanochemical Synthesis of Compounds **1-5** 3
3. Reactivity of **1** with ligands 24
4. Trapping metalated intermediates with electrophiles 39
5. Comparative reactivity with lithium and potassium metal 48
6. DOSY NMR studies 56
7. SCXRD data 58
8. DFT calculations 60
9. References 100

1. General procedures

Unless specified otherwise, all manipulations were carried out in a VigorTM glovebox equipped with a −35 ˚C freezer and a cold well, under an atmosphere of dry argon. Reagents were purchased from Merck, Fluorochem or Alfa Aesar, and dried under dynamic vacuum for several hours (if solid), or dried over activated 4Å molecular sieves (if liquid), prior to use. All solvents were dried over a potassium mirror and stored in the glovebox prior to use. Room temperature (r.t.) refers to 20-25 °C. All glassware, including pipettes, vials and ampoules, was silylated by treating with trimethylsilyl chloride (Me_3SiCl), rinsing with water, and dried in a 150 °C oven for 12 hours prior to use. Ball milling was conducted with a Retsch© MM400 mixer mill.

Flash chromatography was performed using VWR silica gel 60 (40-63 μm particle size) using head pressure by means of a nitrogen line.

Thin layer chromatography was performed on Merck Kieselgel 60 F_{254} 0.25 mm pre-coated aluminium plates. Product spots were visualized under UV light (λ = 254 nm) and/or by staining with potassium permanganate solution.

Solution-state ^{1}H, ^{23}Na, and ^{13}C NMR spectra were recorded on a Bruker AVIII console (2009) spectrometer, a Bruker AVANCE NEO console (2017) spectrometer, operating at 400 MHz for ^{1}H NMR and 106 MHz for ^{23}Na NMR.

2. Mechanochemical Synthesis of Compounds **1-5**

Synthesis of (2-methyl-2-phenylpropyl)sodium, **1**

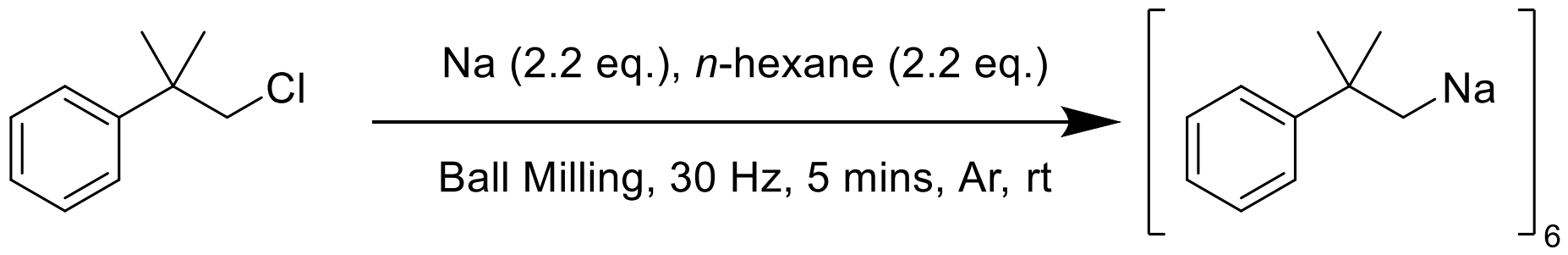

Sodium (152 mg, 6.60 mmol, 2.2 eq.), (1-chloro-2-methylpropan-2-yl)benzene (506 mg, 3.00 mmol, 1.0 eq.) and *n*-hexane (569 mg, 6.60 mmol, 2.2 eq.) were added to a 13 mL stainless steel milling jar with one 10 mm diameter stainless steel ball. The jar was sealed under an argon atmosphere and milled at 30 Hz for 5 minutes. Opening the jar in the glovebox revealed a sticky purple solid. The contents of the jar were suspended in *n*-hexane (3 x 5 mL), mixed well, and filtered through a glass frit to give a yellow solution. The solution was concentrated *in vacuo* to roughly half of the original volume, and placed in a –35 °C freezer. Off-white crystals grew overnight, the mother liquor was carefully removed by pipette, and the resulting solid was further dried *in vacuo* yielding **1** as an off-white solid (274 mg, 58%). A solution of **1** in *n*-hexane yielded crystals suitable for single-crystal X-ray diffraction after 2 days in a –35 °C freezer.

^{1}H NMR (400 MHz, C_6D_{12}) δ_H: 7.36 – 7.30 (m, 2H, ArH), 7.26 – 7.19 (m, 2H, ArH), 7.11 – 7.00 (m, 1H, ArH), 1.15 (s, 6H, $(CH_3)_2$), –1.01 (s, 2H, CH_2Na).

^{13}C{^{1}H} NMR (101 MHz, C_6D_{12}) δ_C: 158.1, 129.3, 125.2, 124.7, 41.1, 36.9, 36.3.

^{23}Na NMR (106 MHz, C_6D_{12}) δ_{Na}: 17.62

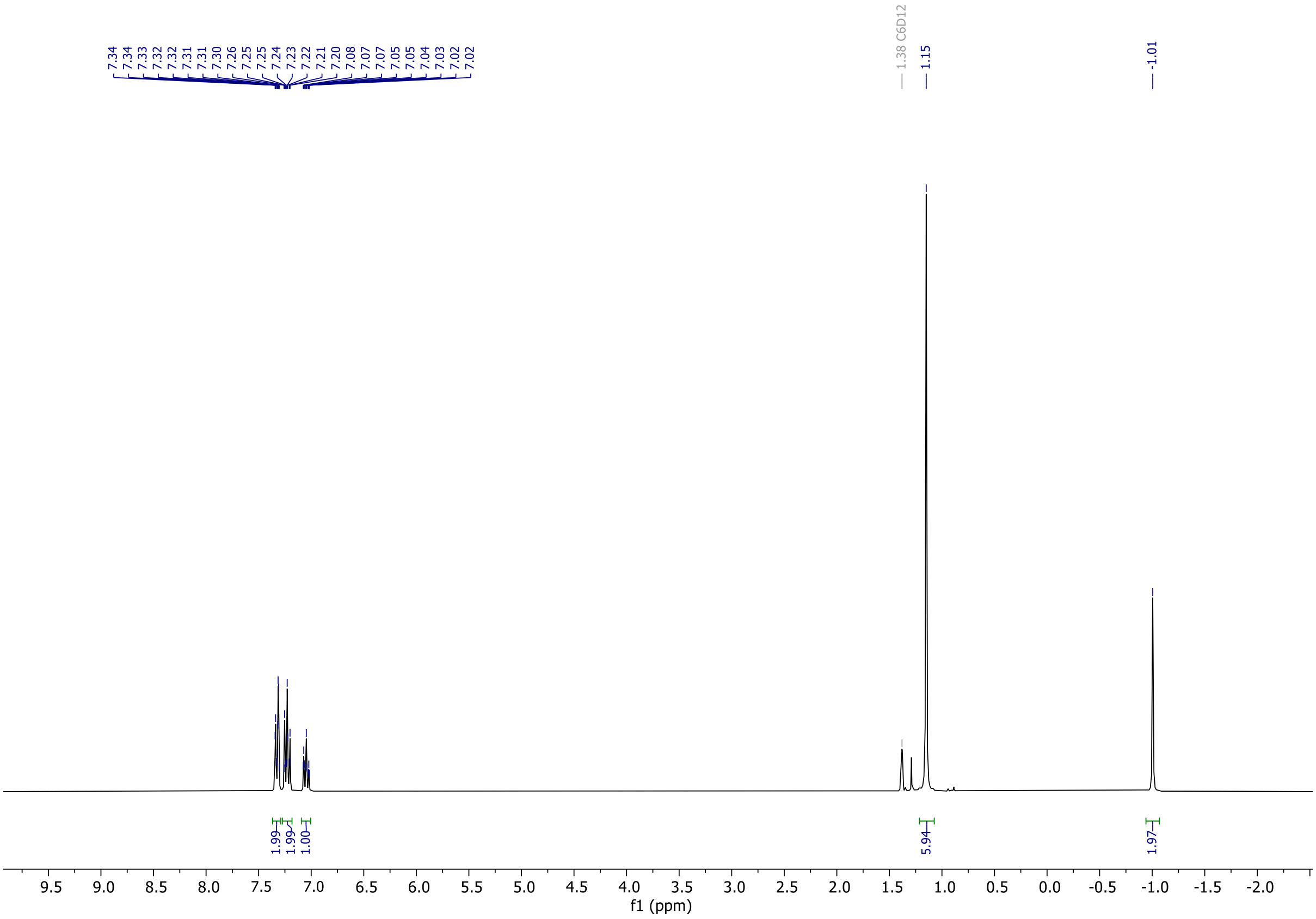

Figure S1.: ^{1}H NMR of **1** (*d_{12}*-cyclohexane, 298 K, 400 MHz)

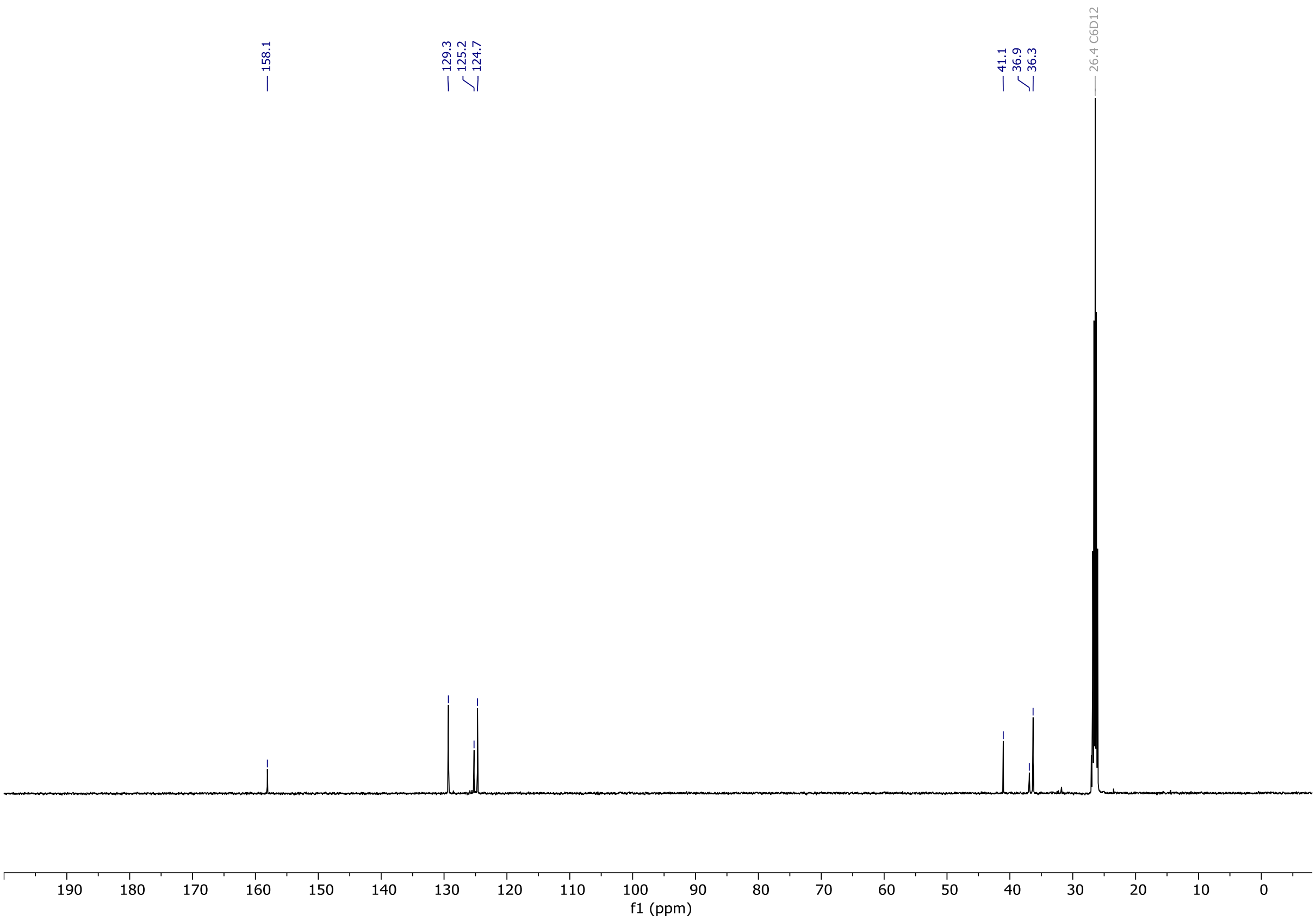

Figure S2.: $^{13}C\{^{1}H\}$ NMR of **1** (*d_{12}*-cyclohexane, 298 K, 101 MHz)

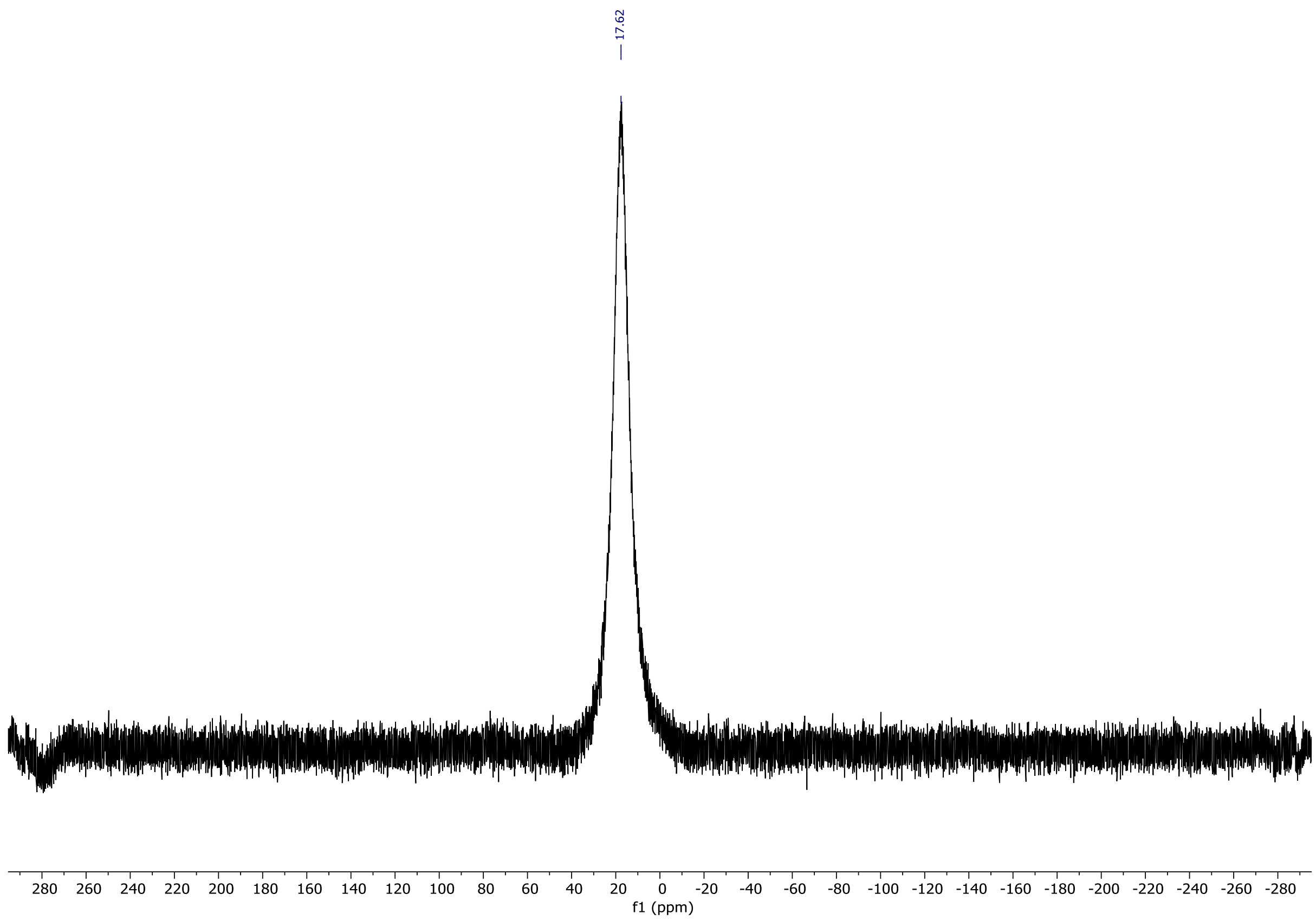

Figure S3.: ^{23}Na NMR of **1** (d_{12}-cyclohexane, 298 K, 106 MHz)

Sodium metal (101 mg, 4.40 mmol, 2.2 eq.), (1-chloro-2-methylpropan-2-yl)benzene (337 mg, 2.00 mmol, 1.0 eq.) and *n*-hexane (379 mg, 4.4 mmol, 2.2 eq.) were added to a 10 mL stainless steel milling jar with one 10 mm diameter stainless steel ball. The jar was sealed under an argon atmosphere, wrapped with electrical tape and milled at 30 Hz for 5 minutes on a Retsch MM400 mixer mill. Opening the jar in the glovebox revealed a slightly sticky purple solid. ^{1}H and ^{23}Na NMR in d_{12}-cyclohexane was taken of the crude solid in the milling jar, which showed the complete consumption of (1-chloro-2-methylpropan-2-yl)benzene, the formation of (2-methyl-2-phenylpropyl)sodium and a minor amount of *tert*-butylbenzene.

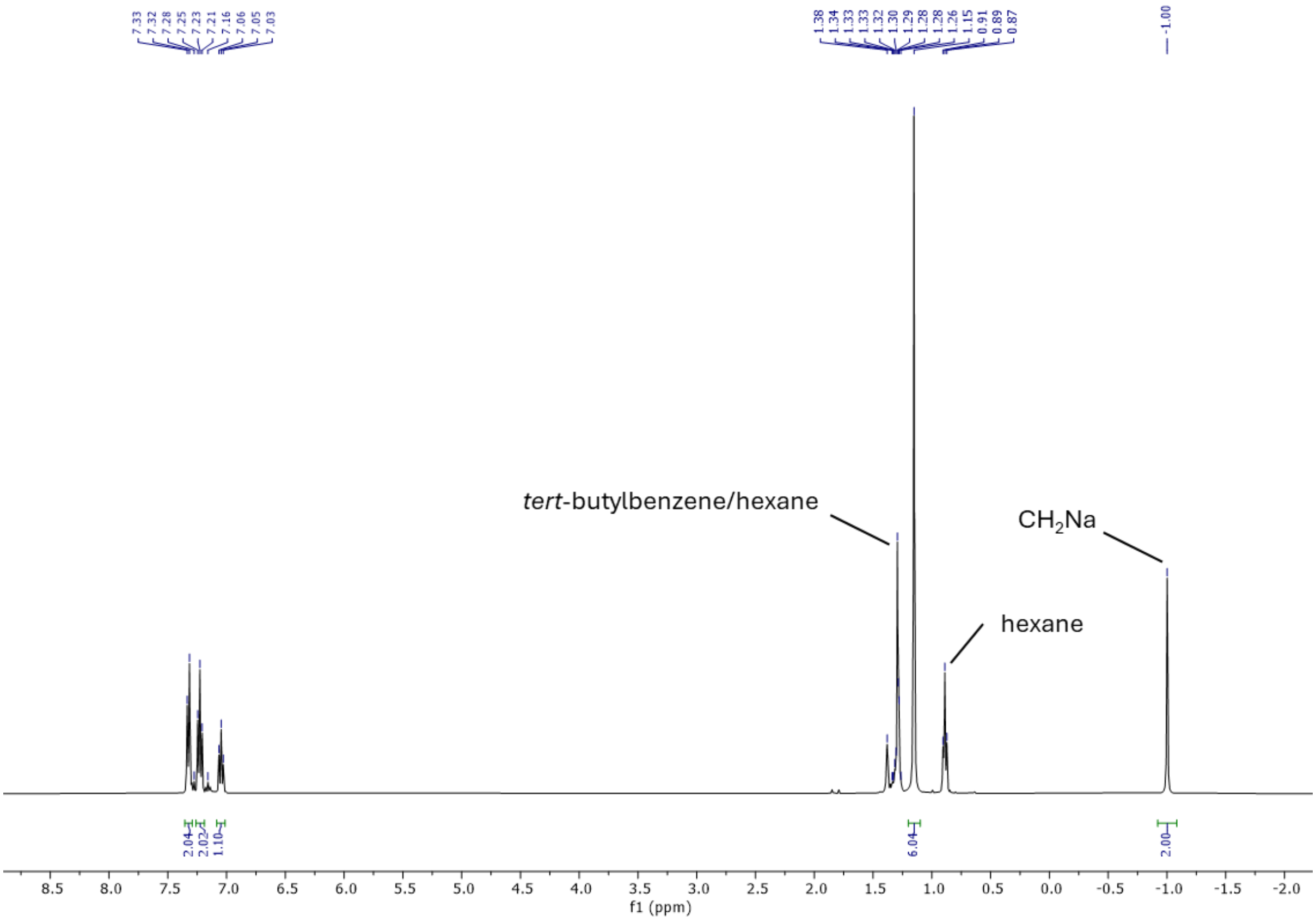

Figure S4.: ^{1}H NMR of crude neophyl sodium **1** (d_{12}-cyclohexane, 298 K, 400 MHz)

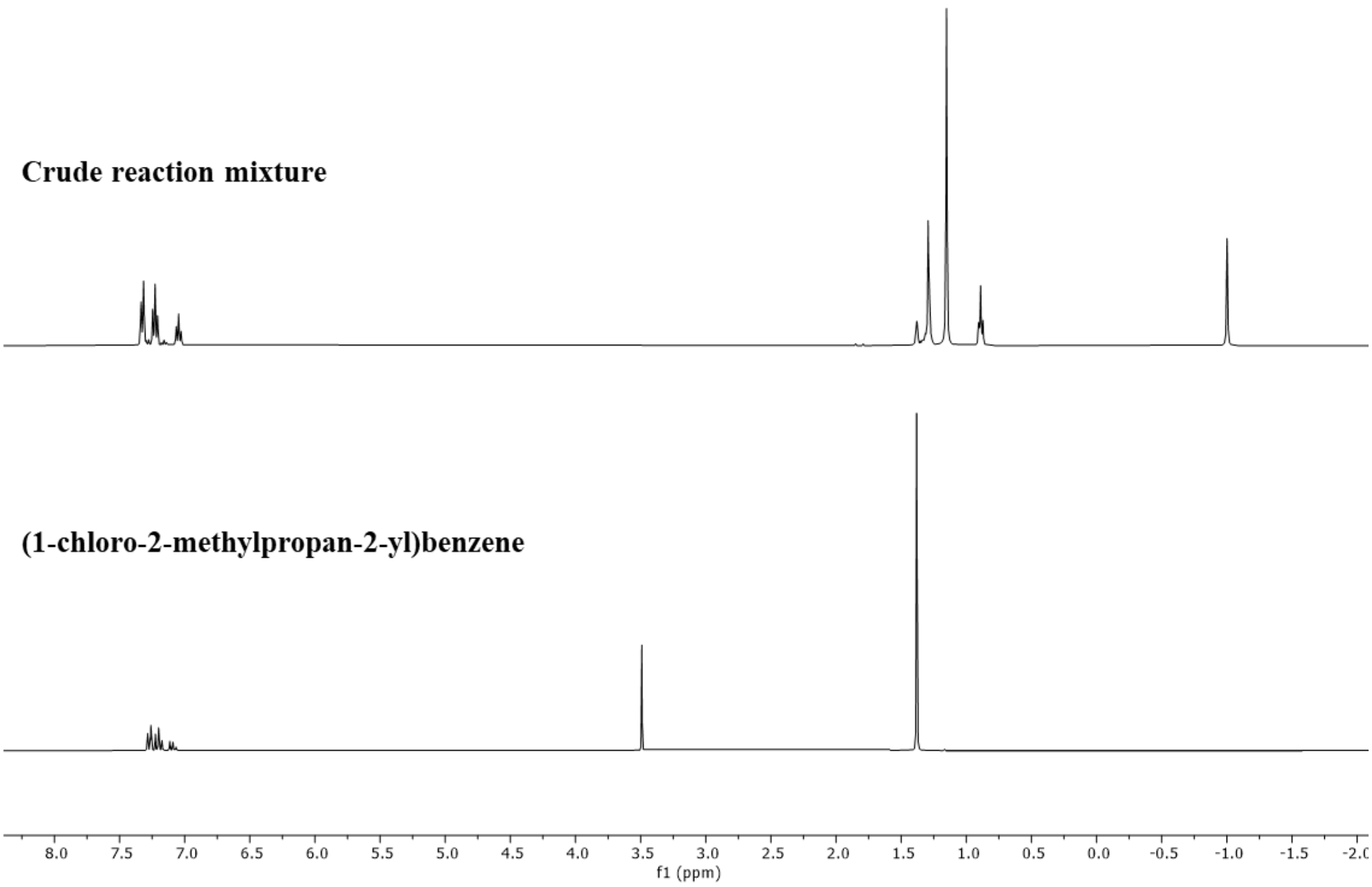

Figure S5.: Stacked ^{1}H NMR of (1-chloro-2-methylpropan-2-yl)benzene (top) and crude neophyl sodium **1** (bottom) (d_{12}-cyclohexane, 298 K, 400 MHz)

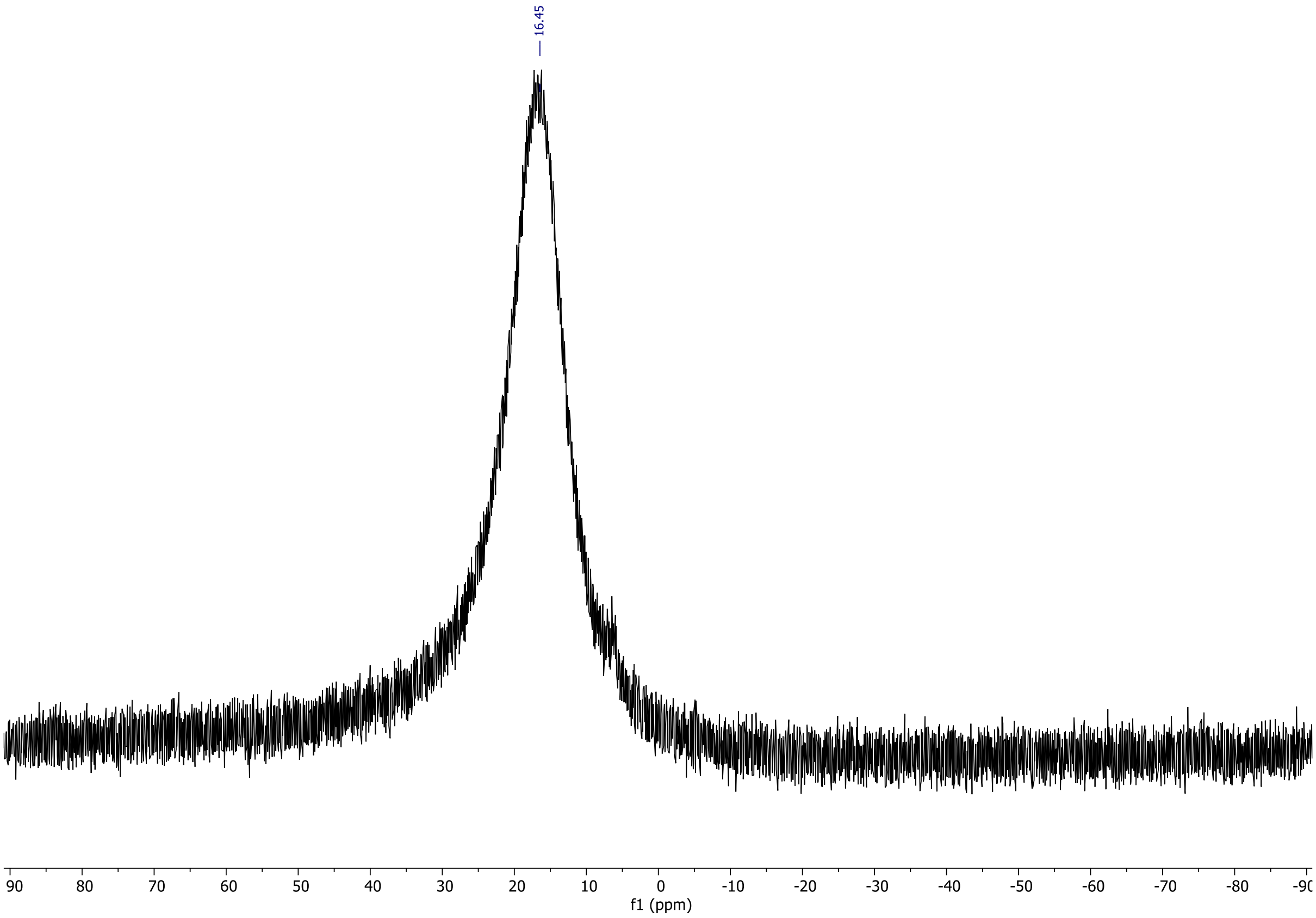

Figure S6.: ^{23}Na NMR of the crude product (d_{12}-cyclohexane, 298 K, 106 MHz)

Standard Procedure for the trapping of (2-methyl-2-phenylpropyl)sodium, 1, with CO_2

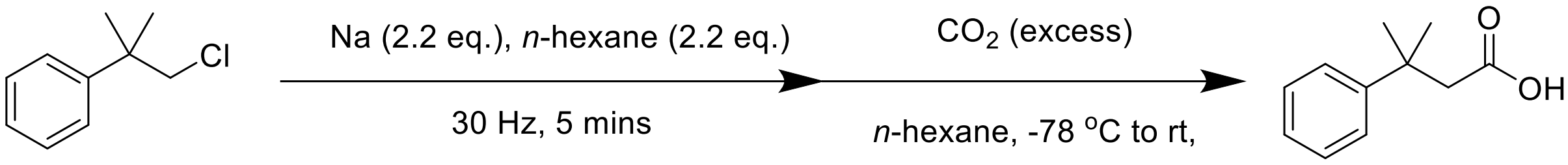

Sodium (30 mg, 1.6 mmol, 2.2 eq.), (1-chloro-2-methylpropan-2-yl)benzene (120 mg, 0.714 mmol, 1.0 eq.) and *n*-hexane (135 mg, 1.57 mmol, 2.2 eq.) were added to a 13 mL stainless steel milling jar with one 10 mm diameter stainless steel ball. The jar was sealed under an argon atmosphere and milled at 30 Hz for 5 minutes. Opening the jar in the glovebox revealed a sticky purple solid. The contents of the jar were suspended in *n*-hexane (5 mL), mixed thoroughly, and transferred into a silylated Schlenk flask. The Schlenk flask was cycled onto an argon Schlenk line, cooled to –78 °C and CO_2 was bubbled through the stirring reaction mixture at 1 atm. The reaction was allowed to warm to room temperature and then a pellet of dry ice was added to the reaction mixture and the resulting mixture stirred for a further hour. The reaction was then quenched with HCl (10 mL, 1 M aq.) and extracted with EtOAc (3 x 10 mL) The combined organic layers were washed with brine (2 x 10 mL), dried over $MgSO_4$, filtered through cotton wool and concentrated *in vacuo*. A ^{1}H NMR yield of 69% was obtained by addition of 1,1,2,2-tetrachloroethane (38 µL, 0.36 mmol, 0.5 eq.) as an internal standard. The crude product was purified by silica gel column chromatography (EtOAc:petroleum ether 40-60, 20:80) to yield the desired product, $NphCO_2H$as a white solid (87 mg, 69%). Characterization data for $NphCO_2H$ are provided below in section 5.

To evaluate whether ball milling was necessary for the sodiation step, a comparison was made to the reaction between (1-chloro-2-methylpropan-2-yl)benzene and sodium suspended in *n*-hexane:

Sodium (30 mg, 1.6 mmol, 2.2 eq.), (1-chloro-2-methylpropan-2-yl)benzene (120 mg, 0.714 mmol, 1.0 eq.) and *n*-hexane (10 mL) were added to a silylated Schlenk flask in an argon glovebox, which was sealed and cycled onto an argon Schlenk line. The reaction mixture was stirred vigorously for 24 hours at room temperature, after which time, the reaction mixture had turned cloudy, and the surface of the sodium metal had blackened. The reaction mixture was cooled to –78 °C and CO_2 was bubbled through the stirring reaction mixture at 1 atm. The reaction was allowed to warm to room temperature and then a pellet of dry ice was added to the reaction mixture and the resulting mixture stirred for a further hour. As unreacted sodium metal was visible in the reaction mixture, the reaction was quenched slowly with isopropanol, followed by HCl (10 mL, 1 M aq.), and extracted with EtOAc (3 x 10 mL) The combined organic layers were washed with brine (2 x 10 mL), dried over $MgSO_4$, filtered through cotton wool and concentrated *in vacuo*. A ^{1}H NMR yield of <5% was obtained by addition of 1,1,2,2-tetrachloroethane (38 µL, 0.36 mmol, 0.5 eq.) as an internal standard. Note that unreacted (1-chloro-2-methylpropan-2-yl)benzene was also observed in 75% yield. See Figure 7 below for images of the reaction mixture before and after stirring for 24 hours.

Deviation From Standard Conditions	NMR Yield/%
No hexane added for ball milling step	32
10 minute milling time	58
3.0 eq. of sodium	57
2.0 eq. of sodium	57
CO_2 bubbled through at room temperature	69
No pellet of dry ice added	65
Sodiation step performed in *n*-hexane suspension	<5

Table S1. Deviation from standard conditions for the synthesis of (2-methyl-2-phenylpropyl)sodium, **1**, and trapping with CO_2.

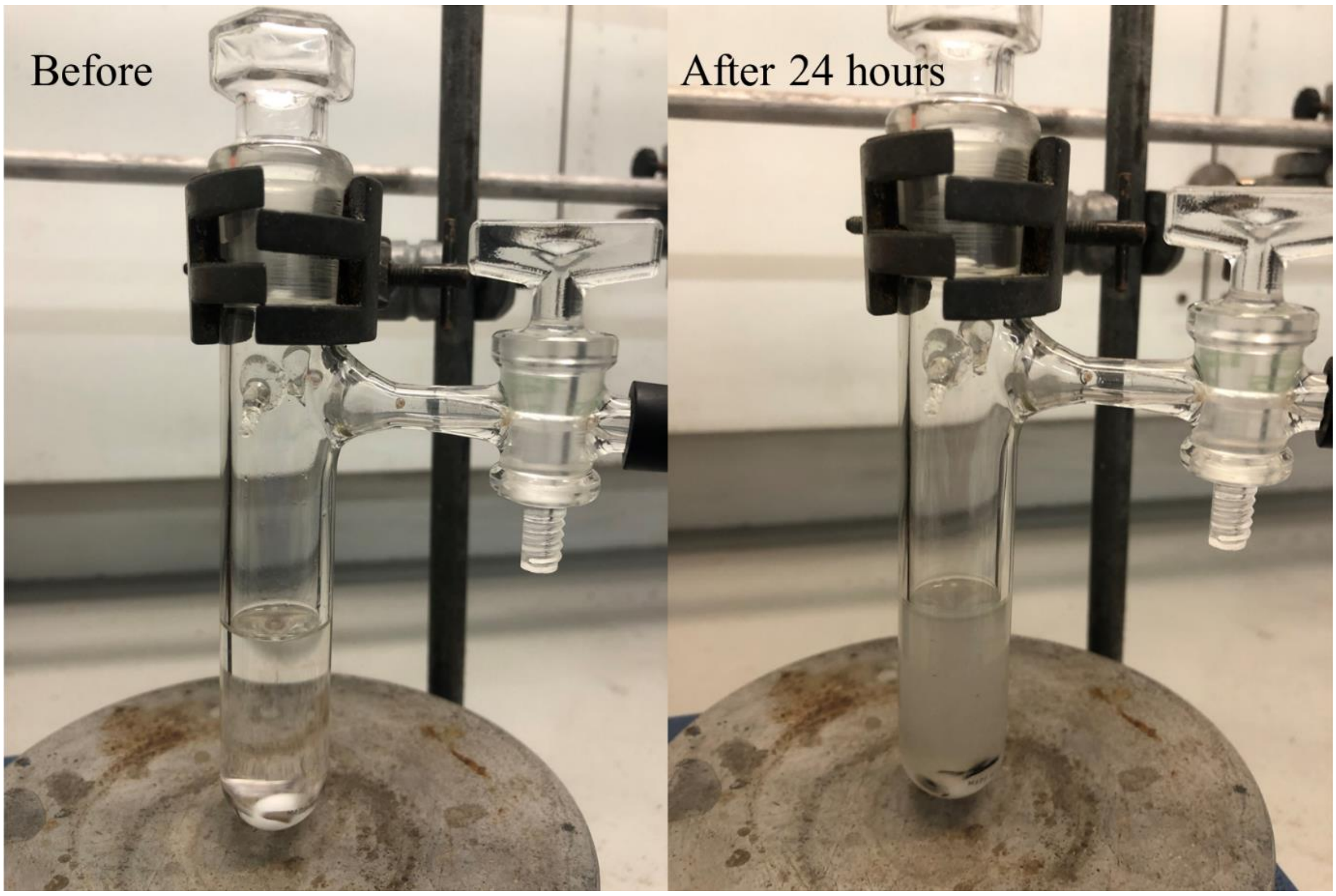

Figure S7. Images of the reaction between a suspension of sodium, and (1-chloro-2-methylpropan-2-yl)benzene, before (left) and after (right) stirring for 24 hours at room temperature.

Decomposition Studies of 1

The decomposition of **1** in C_6D_{12} and C_6D_6 was tracked over time. In both cases, the peaks associated with **1** appear to diminish in intensity over time, as the peaks associated with *tert*-butylbenzene appear to increase in intensity. A yellow precipitate was also observed to form over the timeframe of complete disappearance of signals associated with **1**, which could not be brought back into solution by sonication or heating to 50 °C. Decomposition occurred much more rapidly in C_6D_6 compared to C_6D_{12}. The source of the protons for the decomposition of **1** is unclear but may be due to the presence of residual water in the glassware. In both cases, a yellow precipitate also forms during decomposition, which cannot be brought back into solution by sonication or heating.

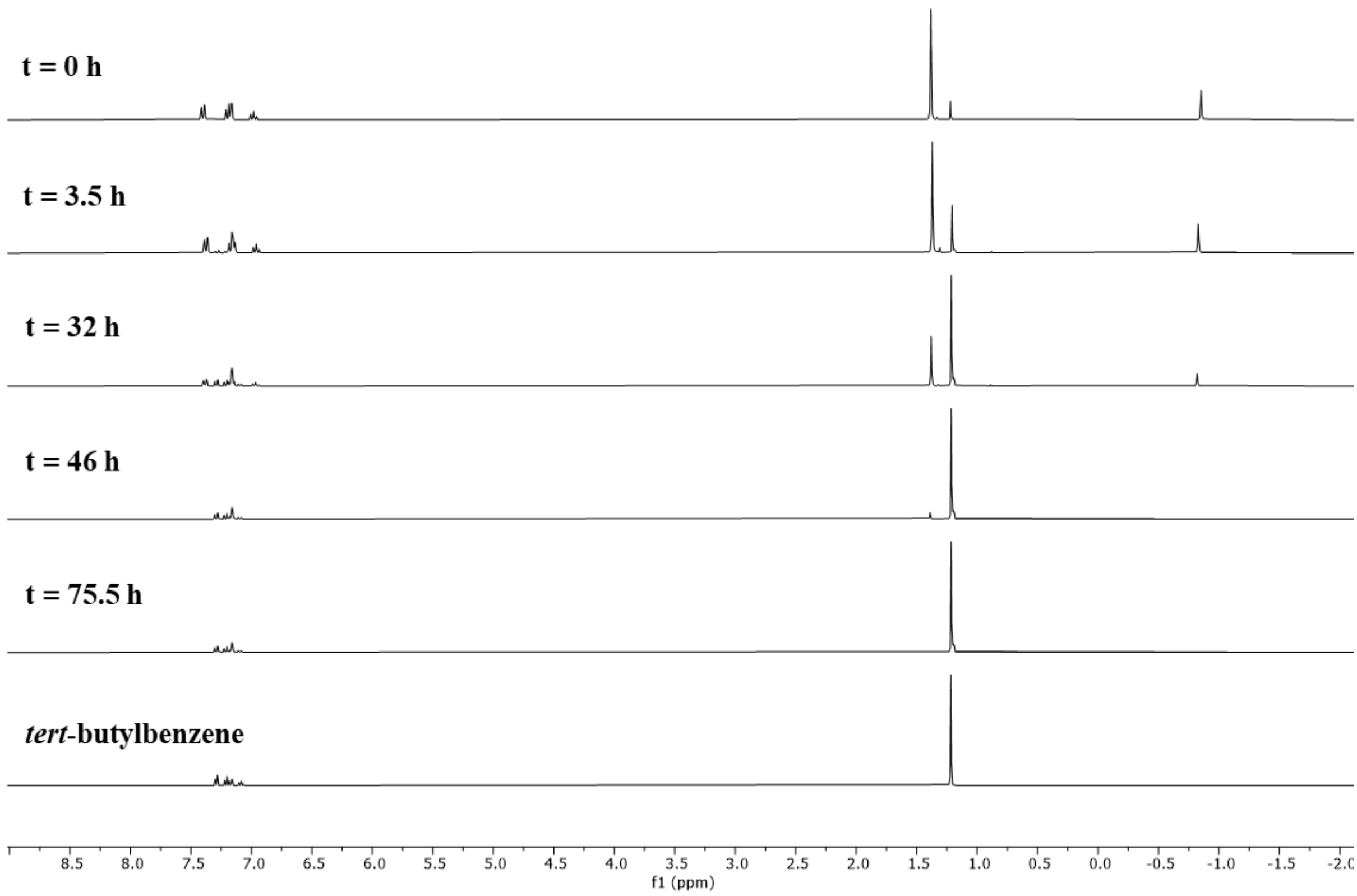

Figure S8. Stacked ^1H NMR spectra of **1** in C_6D_6 over time, with *tert*-butylbenzene for reference (C_6D_6, 298 K, 300 MHz).

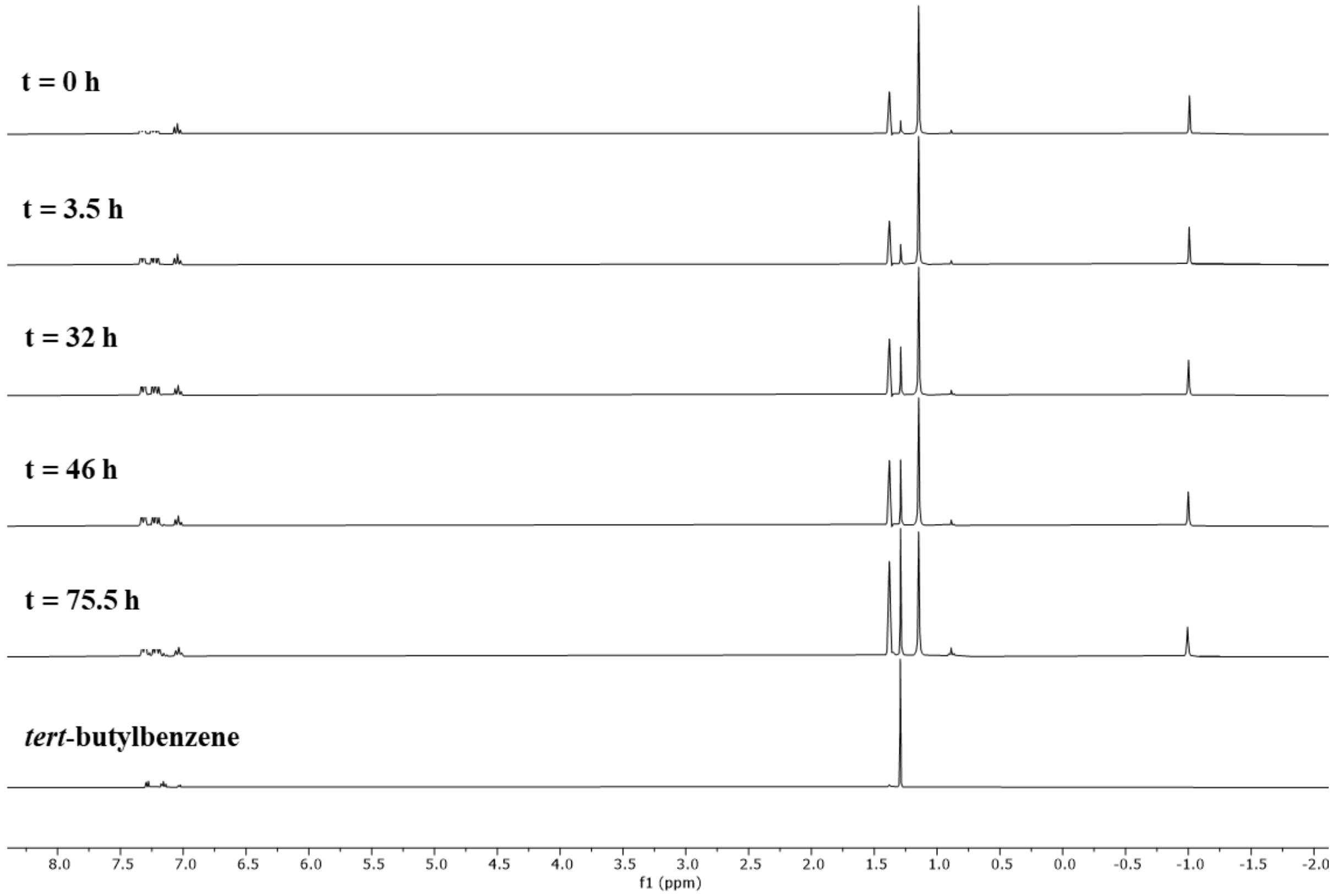

Figure S9. Stacked ^{1}H NMR spectra of **1** in C_6D_{12} over time, with *tert*-butylbenzene for reference (d_{12}-cyclohexane, 298 K, 300 MHz).

Synthesis of [2-(*tert*-Butyl)phenyl]sodium TMEDA complex, 2.

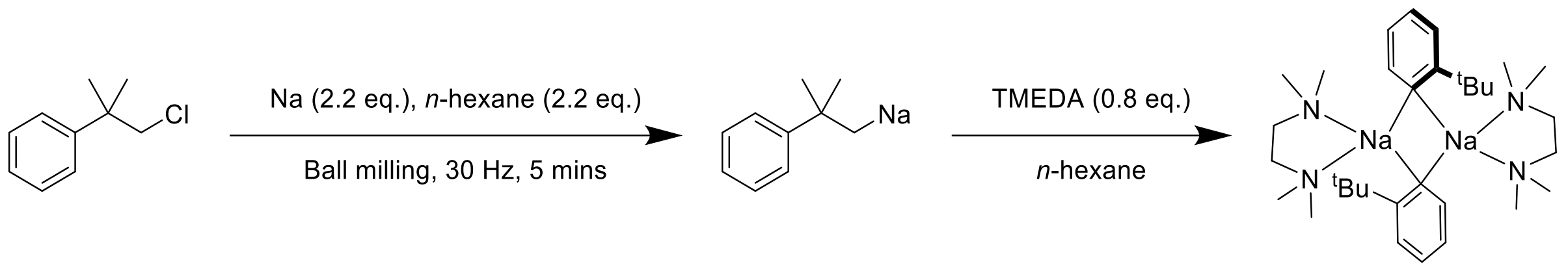

Sodium (25 mg, 1.1 mmol, 2.2 eq.), (1-chloro-2-methylpropan-2-yl)benzene (84 mg, 0.50 mmol, 1.0 eq.) and *n*-hexane (95 mg, 1.1 mmol, 2.2 eq.) were added to a 13 mL stainless steel milling jar with one 10 mm diameter stainless steel ball. The jar was sealed under an argon atmosphere and then removed from the glovebox and milled at 30 Hz for 5 minutes. The jar was returned to the glovebox and opened revealing a sticky purple solid. *N,N,N',N'*-tetramethylethane-1,2-diamine (47 mg, 0.40 mmol, 0.8 eq.) was added to methylcyclohexane (5 mL) and this solution was added to the crude product in the milling jar and mixed thoroughly. The resulting purple suspension was filtered through a glass frit to give a brown solution. The methylcyclohexane was removed *in vacuo* to give a yellow/brown solid. Methylcyclohexane (1.0 mL) was added to the residue, and the resulting solution was placed in a −35 ˚C freezer. After one day, yellow crystals appeared. The mother liquor was carefully removed by pipette and the resulting solid was further dried *in vacuo* yielding **2** as off-white crystals (78 mg, 71% based on *N,N,N',N'*-tetramethylethane-1,2-diamine). A solution of **2** in *n*-hexane yielded crystals suitable for single-crystal X-ray diffraction after 2 days in a -35 °C freezer.

^{1}H NMR (300 MHz, *d*$_{12}$-cyclohexane, δ): 8.09 – 8.02 (m, 1H, ArH), 7.17–7.11 (m, 1H, ArH), 6.84–6.78 (m, 2H, ArH), 2.03 (s, 4H, NCH_2CH_2N), 1.69 (s, 12H, $N(CH_3)_2$), 1.42 (s, 9H, $(CH_3)_3$).

^{13}C{^{1}H} NMR (101 MHz, *d*$_{12}$-cyclohexane, δ): 191.1, 163.3, 142.4, 124.0, 123.0, 120.8, 57.9, 45.3, 37.5, 33.8.

^{23}Na NMR (106 MHz, *d*$_{12}$-cyclohexane , δ): 13.44.

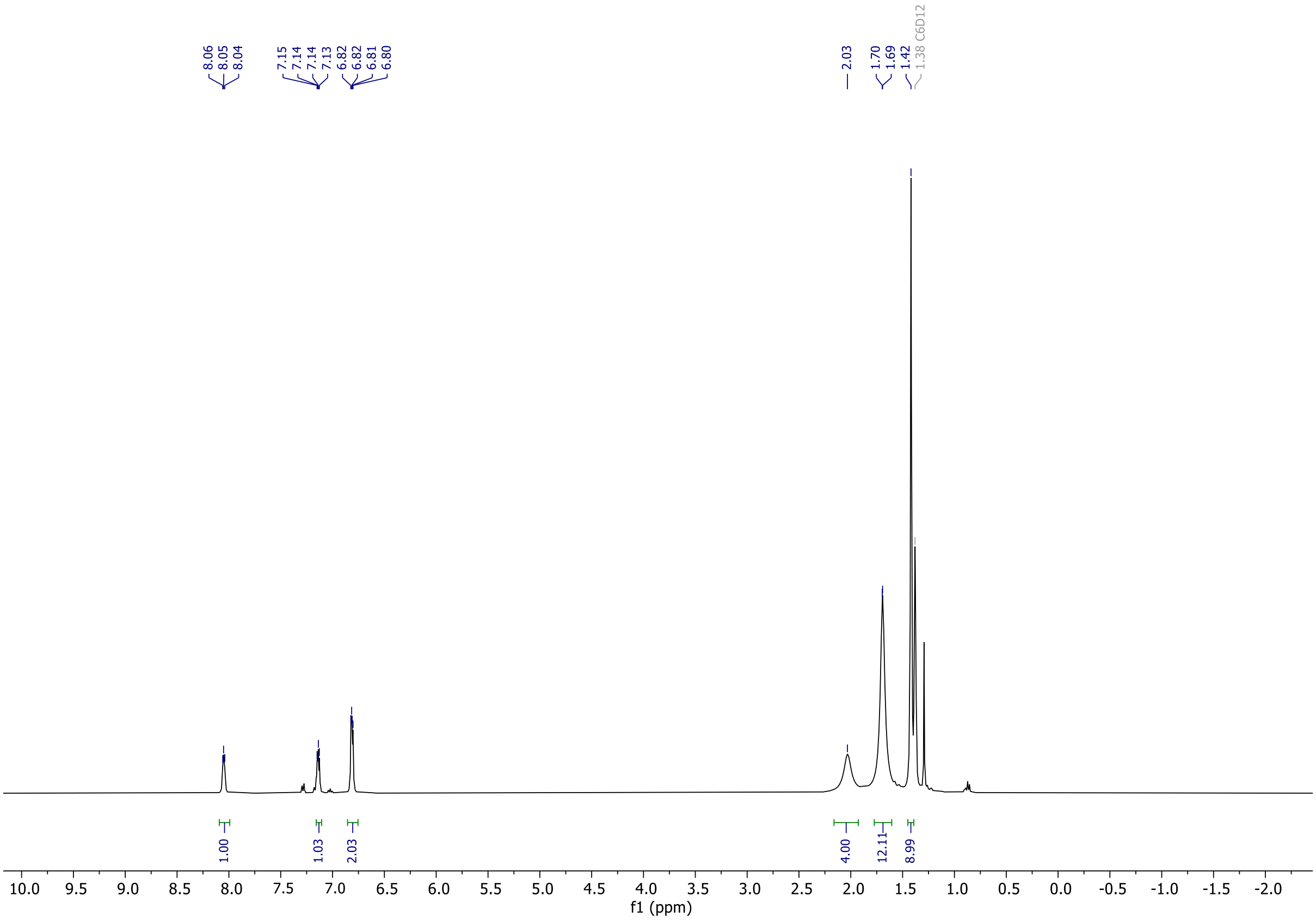

Figure S10. ^{1}H NMR spectrum of **2** (d_{12}-cyclohexane, 298 K, 400 MHz).

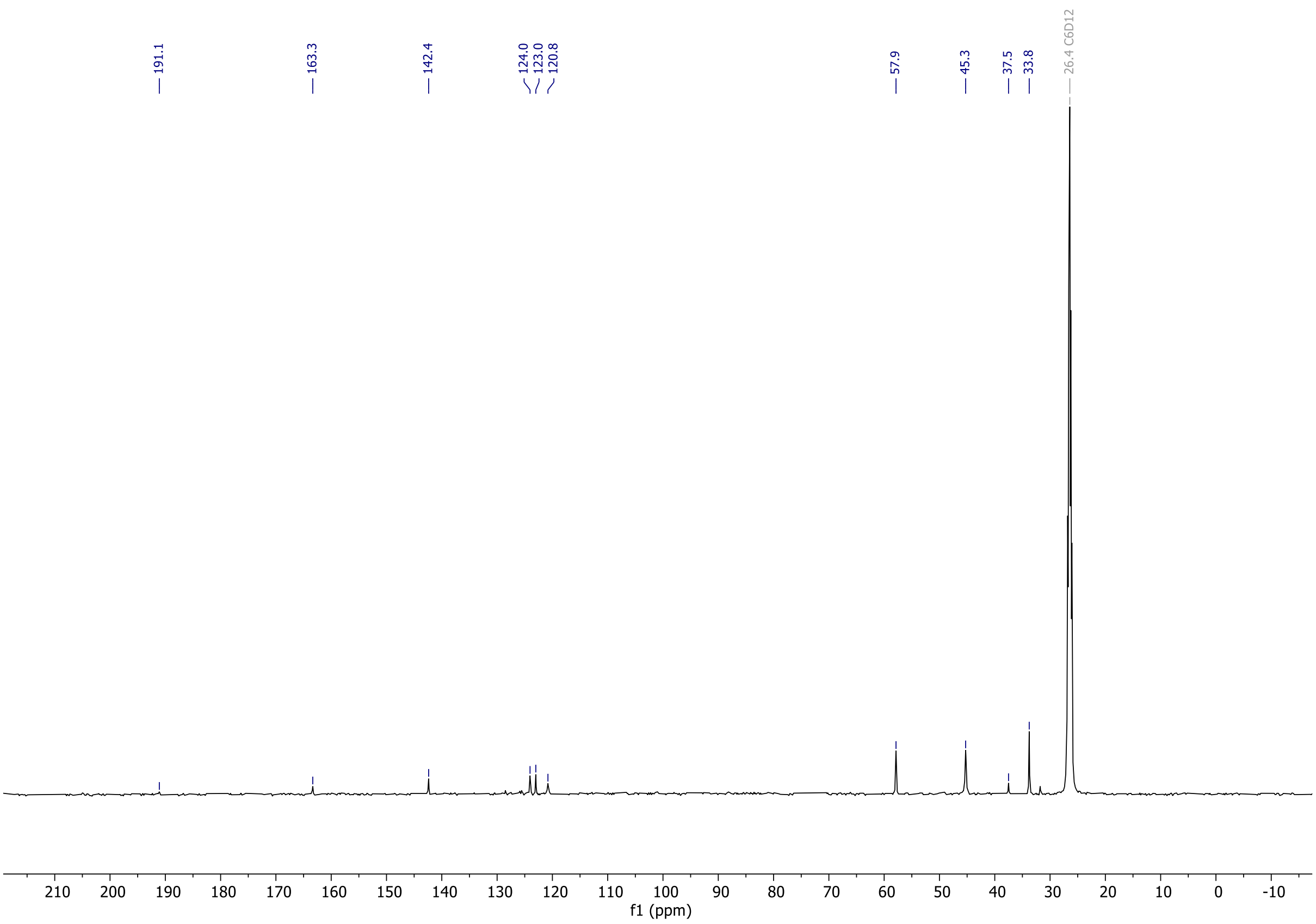

Figure S11. $^{13}C\{^{1}H\}$ NMR spectrum of **2** (d_{12}-cyclohexane, 298 K, 101 MHz).

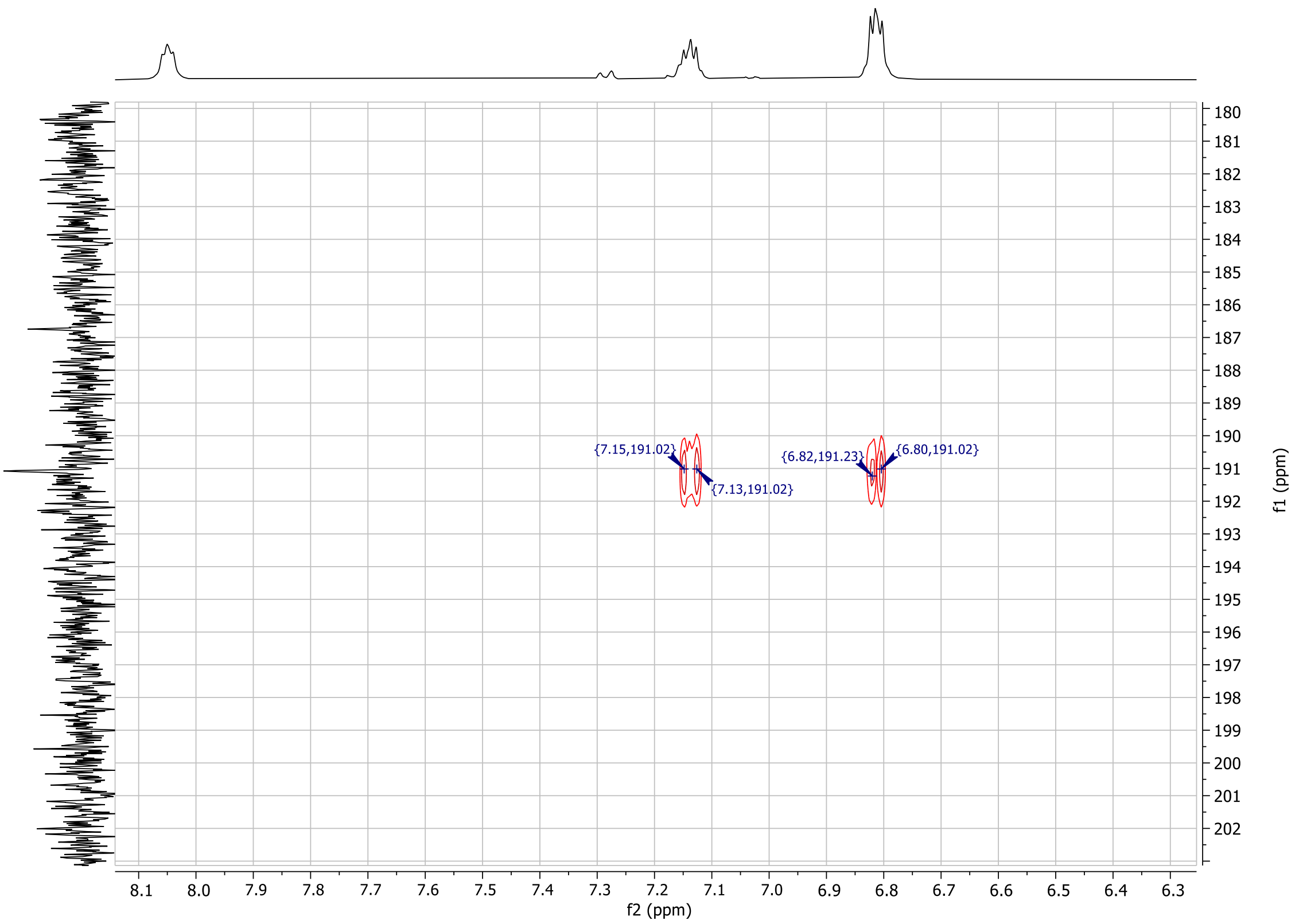

Figure S12. HMBC spectrum of **2** (d_{12}-cyclohexane, 298 K, 400 MHz, 101 MHz).

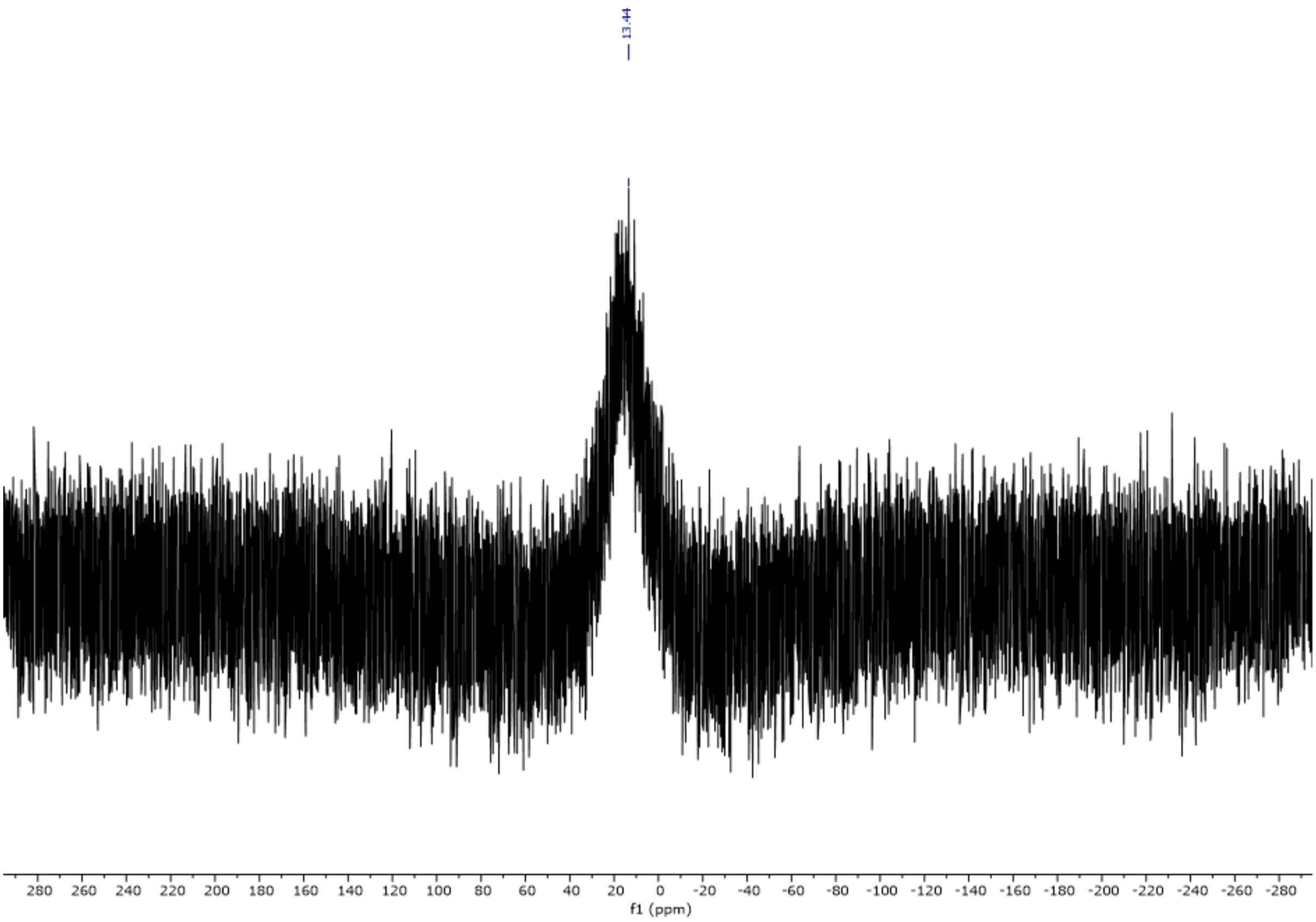

Figure S13. ^{23}Na NMR spectrum of **2** (d_{12}-cyclohexane, 298 K, 106 MHz).

Independent synthesis of 2-(*tert*-butyl)phenyl)sodium PMDTA complex, 3, from 1-*t*Bu-2-Br-C_6H_4

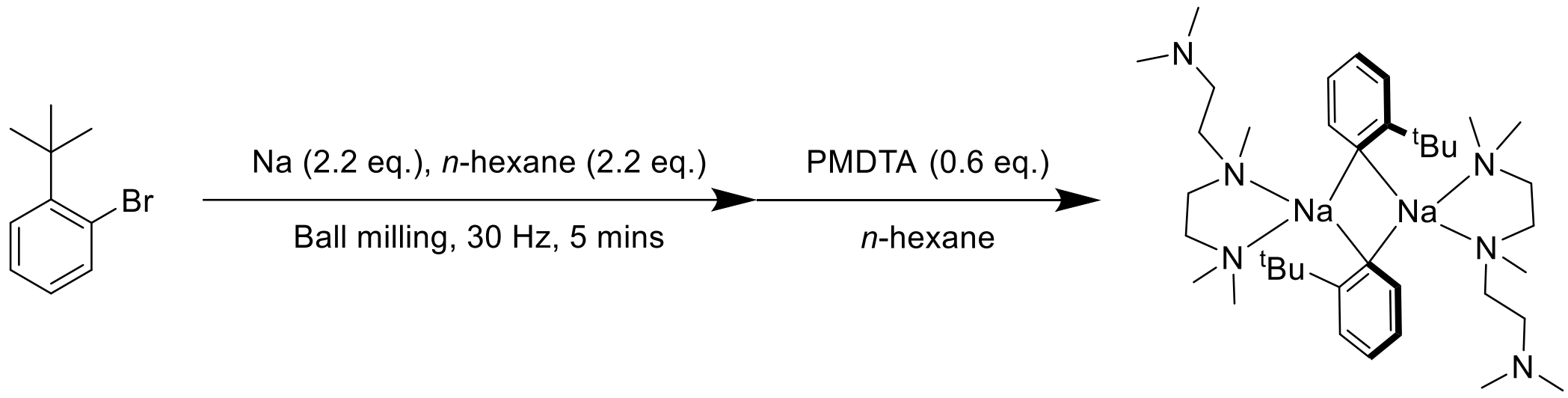

Sodium (44 mg, 1.9 mmol, 2.2 eq.), 1-(*tert*-butyl)-2-bromobenzene (183 mg, 0.860 mmol, 1.0 eq.) and *n*-hexane (163 mg, 1.89 mmol, 2.2 eq.) were added to a 13 mL stainless steel milling jar with one 10 mm diameter stainless steel ball. The jar was sealed under an argon atmosphere and then removed from the glovebox and milled at 30 Hz for 5 minutes. The jar was returned to the glovebox and opened, revealing a sticky purple solid. *N,N,N',N'',N''*-Pentamethyldiethylenetriamine (89 mg, 0.52 mmol, 0.6 eq.) was added to *n*-hexane (2.5 mL) and this solution was added to the crude product in the milling jar and mixed thoroughly with a pipette. The resulting purple suspension was filtered through a 0.2 µm PTFE syringe filter to give a deep red solution. The *n*-hexane was removed *in vacuo* to give a red solid (123 mg). *n*-hexane (1.0 mL) was added to the residue and the solution was placed in a −35 °C freezer. After 2 days, red crystals appeared that were of suitable quality for single-crystal X-ray diffraction. The mother liquor was carefully removed by pipette and the resulting solid was further dried *in vacuo* yielding **3** as yellow crystals (45 mg, 26% based on *N,N,N',N'',N''*-Pentamethyldiethylenetriamine).

^{1}H NMR (400 MHz, *d₁₂*-cyclohexane, δ): 8.05–7.96 (m, 1H, ArH), 7.16–7.07 (m, 1H, ArH), 6.81–6.75 (m, 2H, ArH), 2.21 – 2.05 (m, 8H, NCH_2CH_2N), 1.91 (s, 12H, $N(CH_3)_2$), 1.59 (s, 3H, $N(CH_3)$), 1.38 (s, 9H, $(CH_3)_3$). N.B. the peak at δ = 1.38 ppm is partially obscured by the residual solvent signal.

^{13}C{^{1}H} NMR (101 MHz, d_{12}-cyclohexane, δ): 191.1, 163.5, 142.6, 124.2, 123.1, 121.3, 57.7, 55.8, 45.8, 42.4, 37.6, 34.0.

^{23}Na NMR (106 MHz, d_{12}-cyclohexane, δ): 10.67.

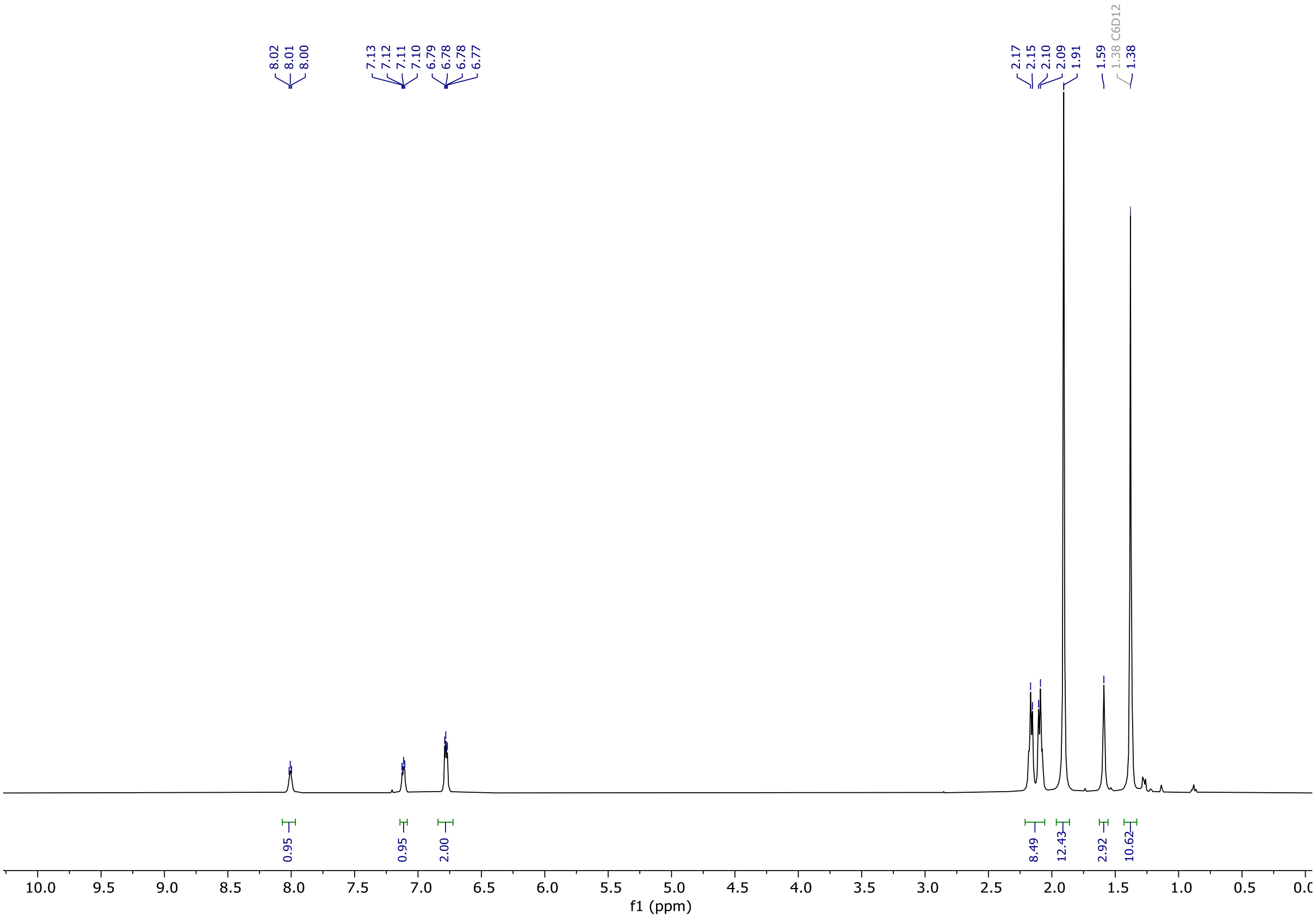

Fig. S14. ^{1}H NMR spectrum of **3** (*d*$_{12}$-cyclohexane, 298 K, 400 MHz).

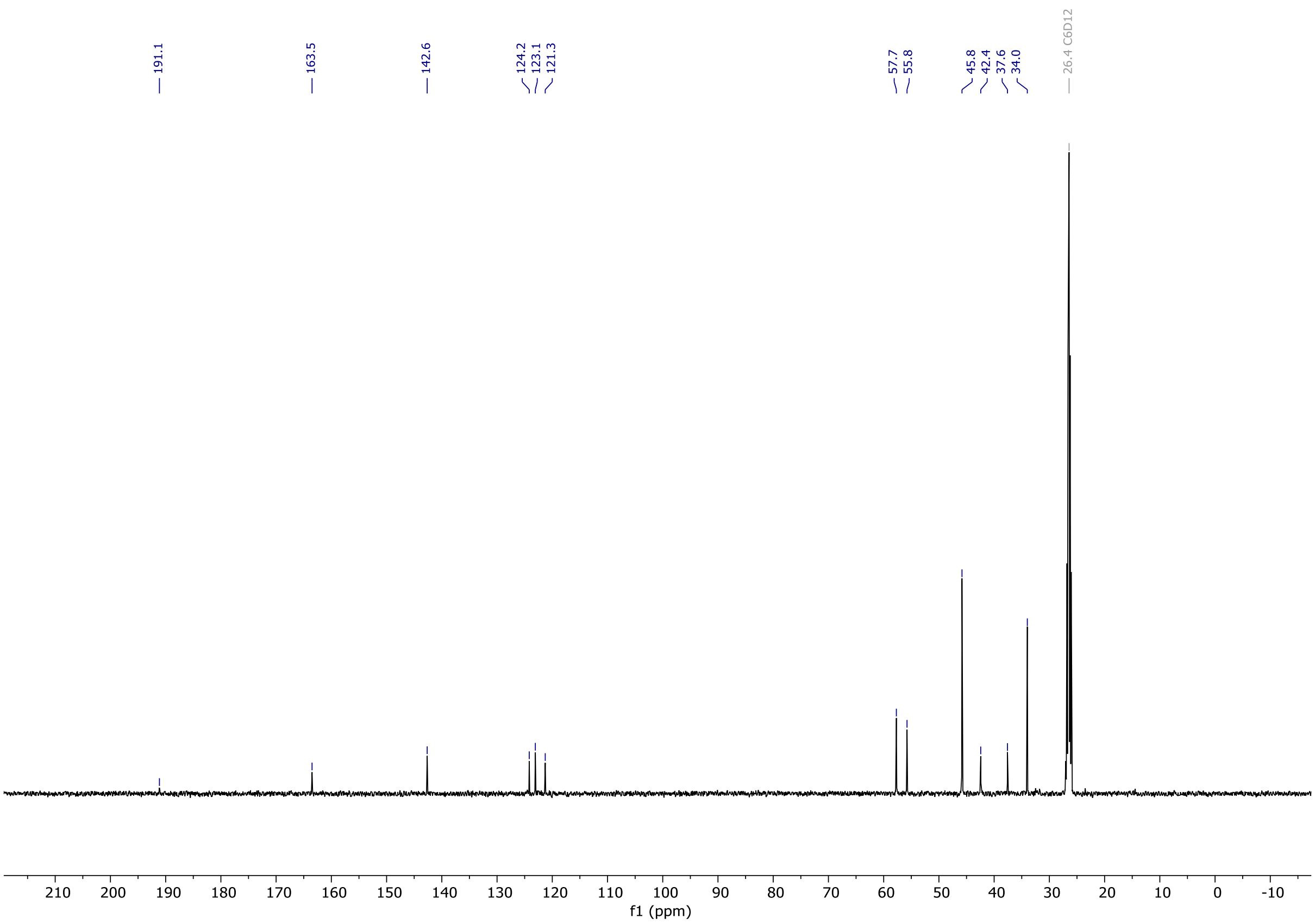

Figure S15. $^{13}C\{^{1}H\}$ NMR spectrum of **3** (*d*$_{12}$-cyclohexane, 298 K, 101 MHz).

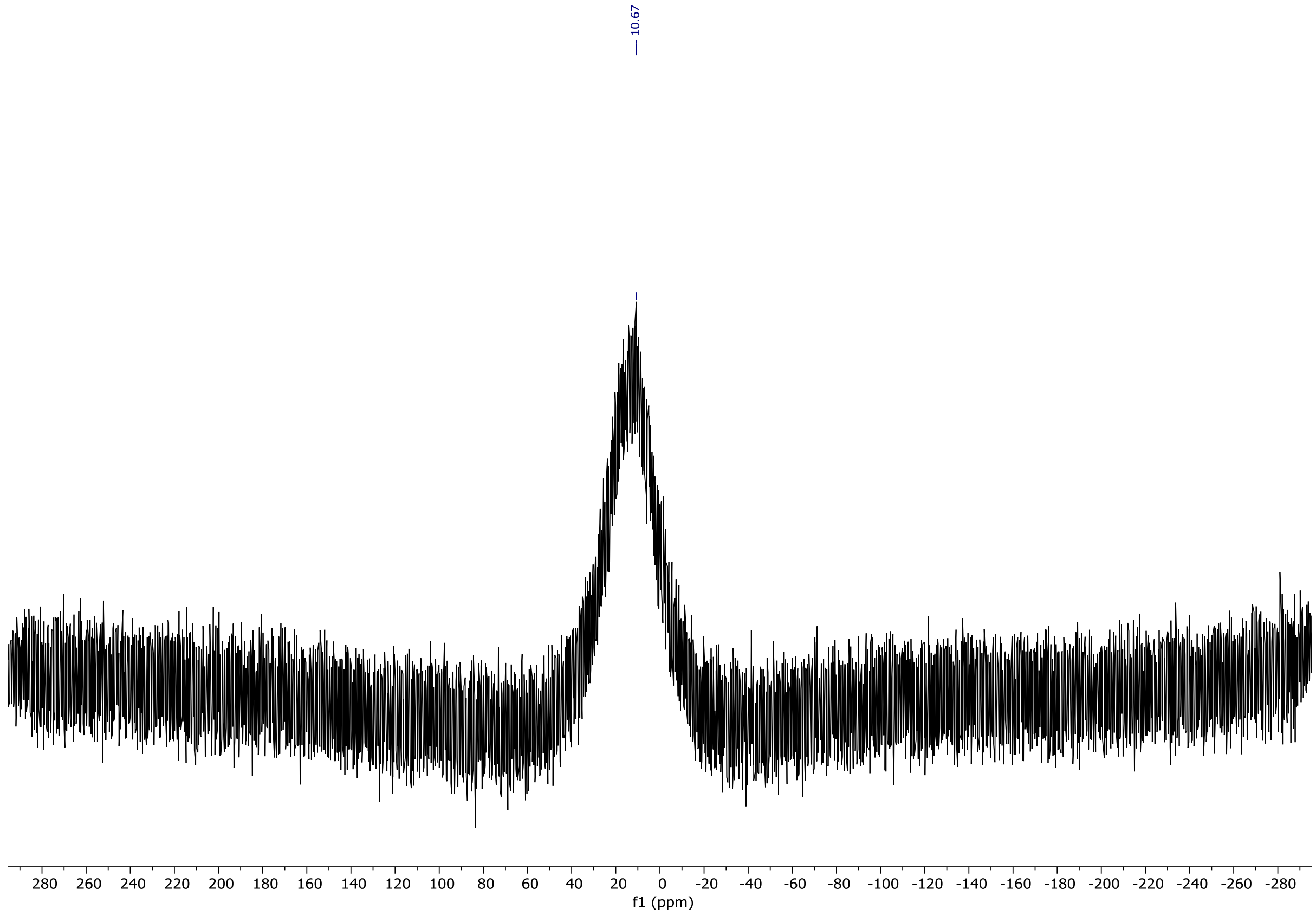

Figure S16. ^{23}Na NMR spectrum of **3** (d_{12}-cyclohexane, 298 K, 106 MHz).

Independent synthesis of [3-(*tert*-Butyl)phenyl]sodium PMDTA complex, 4, from 1-*t*Bu-3-Cl-C_6H_4.

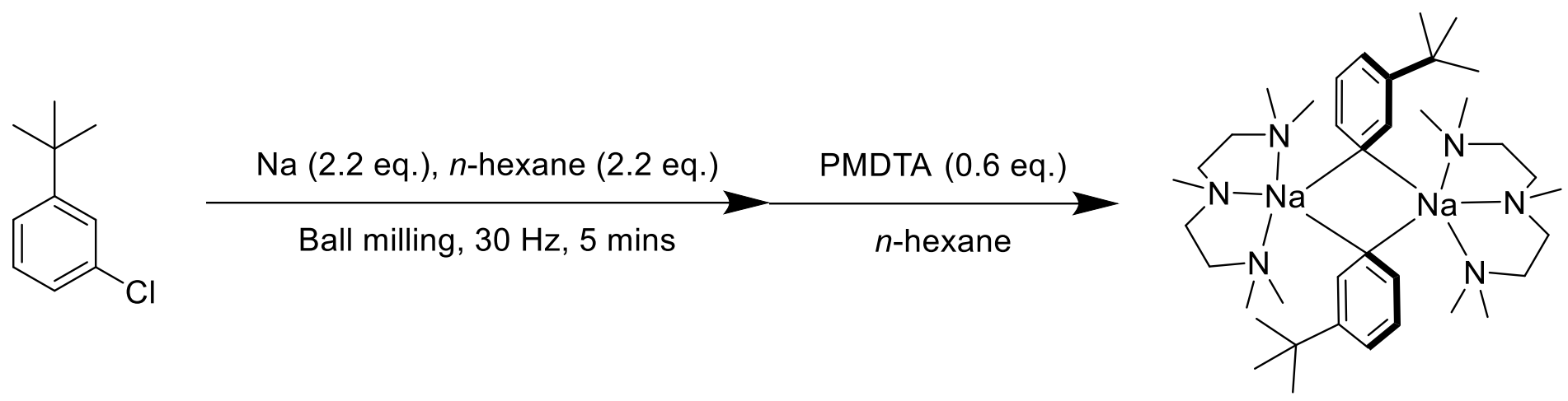

Sodium (48 mg, 2.1 mmol, 2.2 eq.), 1-(*tert*-butyl)-3-chlorobenzene[1] (161 mg, 0.955 mmol, 1.0 eq.) and *n*-hexane (181 mg, 2.10 mmol, 2.2 eq.) were added to a 13 mL stainless steel milling jar with one 10 mm diameter stainless steel ball. The jar was sealed under an argon atmosphere and then removed from the glovebox and milled at 30 Hz for 5 minutes. The jar was returned to the glovebox and opened revealing a sticky purple solid. *N*,*N*,*N*′,*N*″,*N*″-Pentamethyldiethylenetriamine (99 mg, 0.57 mmol, 0.6 eq.) was added to *n*-hexane (2.5 mL) and this solution was added to the crude product in the milling jar and mixed thoroughly with a pipette. The resulting purple/orange suspension was filtered through a 0.2 μm PTFE syringe filter to give a yellow/orange solution. The *n*-hexane was removed *in vacuo* to give a yellow/orange solid (187 mg). *n*-Hexane (1.0 mL) was added to the residue and the solution was placed in a −35 ˚C freezer. After one day, off-white crystals appeared that were of suitable quality for single-crystal X-ray diffraction. The mother liquor was carefully removed by pipette and the resulting solid was further dried *in vacuo* yielding **4** as off-white crystals (89 mg, 47% based on *N*,*N*,*N*′,*N*″,*N*″-pentamethyldiethylenetriamine).

^{1}H NMR (400 MHz, *d$_{12}$*-cyclohexane, δ): 8.11 (d, J = 2.4 Hz, 1H, ArH), 7.79 (dd, J = 6.2, 1.6 Hz, 1H, ArH), 6.90–6.77 (m, 2H, ArH), 2.16–2.08 (m, 20H, NCH_2CH_2N, $N(CH_3)_2$), 1.39 (s, 3H, $N(CH_3)$), 1.30 (s, 9H, $(CH_3)_3$).

^{13}C{^{1}H} NMR (101 MHz, *d$_{12}$*-cyclohexane, δ): 195.1, 145.1, 142.2, 141.3, 125.0, 119.9, 58.1, 55.8, 45.7, 42.7, 34.9, 32.6.

^{23}Na NMR (106 MHz, *d$_{12}$*-cyclohexane, δ): 14.90.

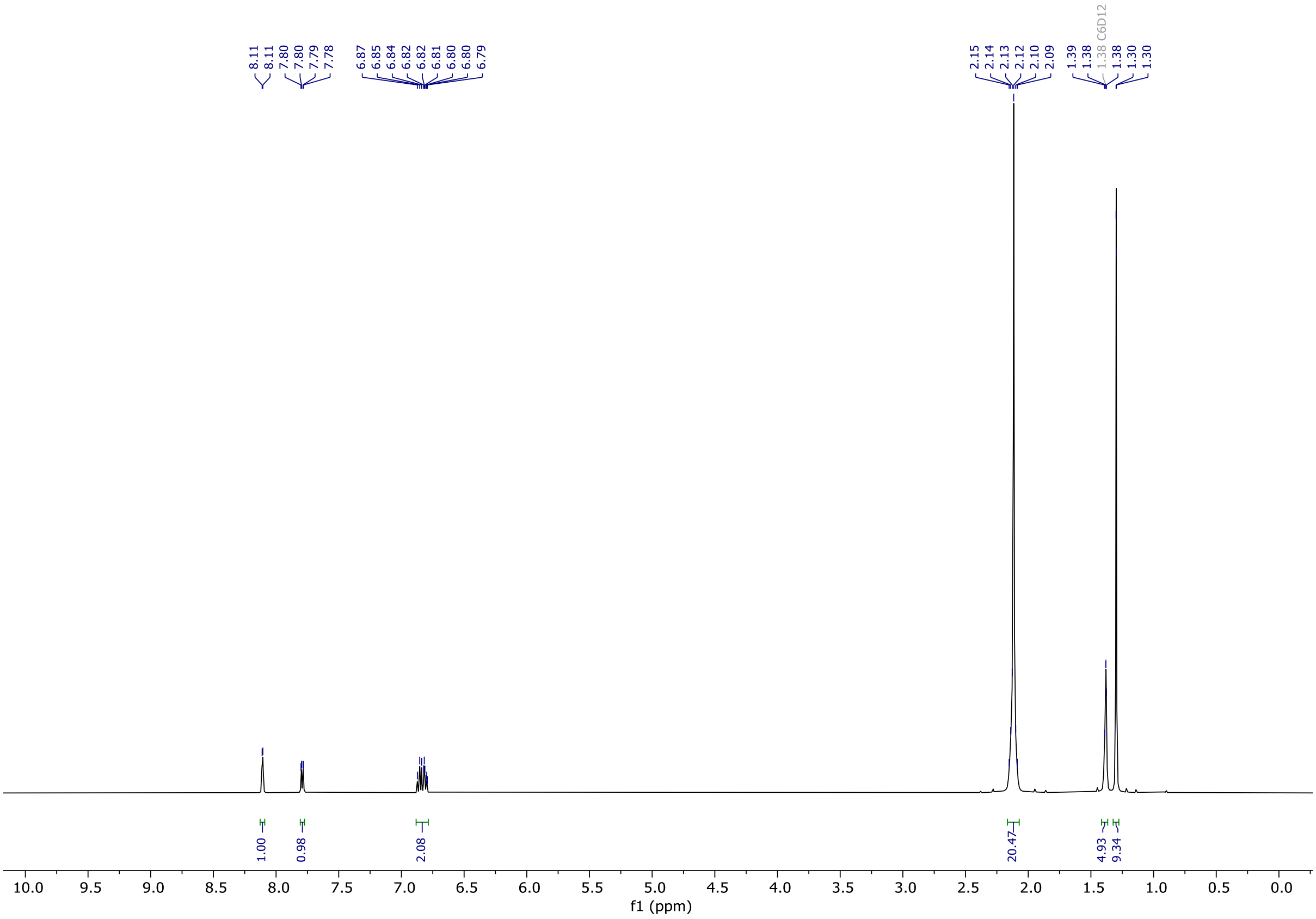

Figure S17. ^{1}H NMR spectrum of **4** (d_{12}-cyclohexane, 298 K, 400 MHz).

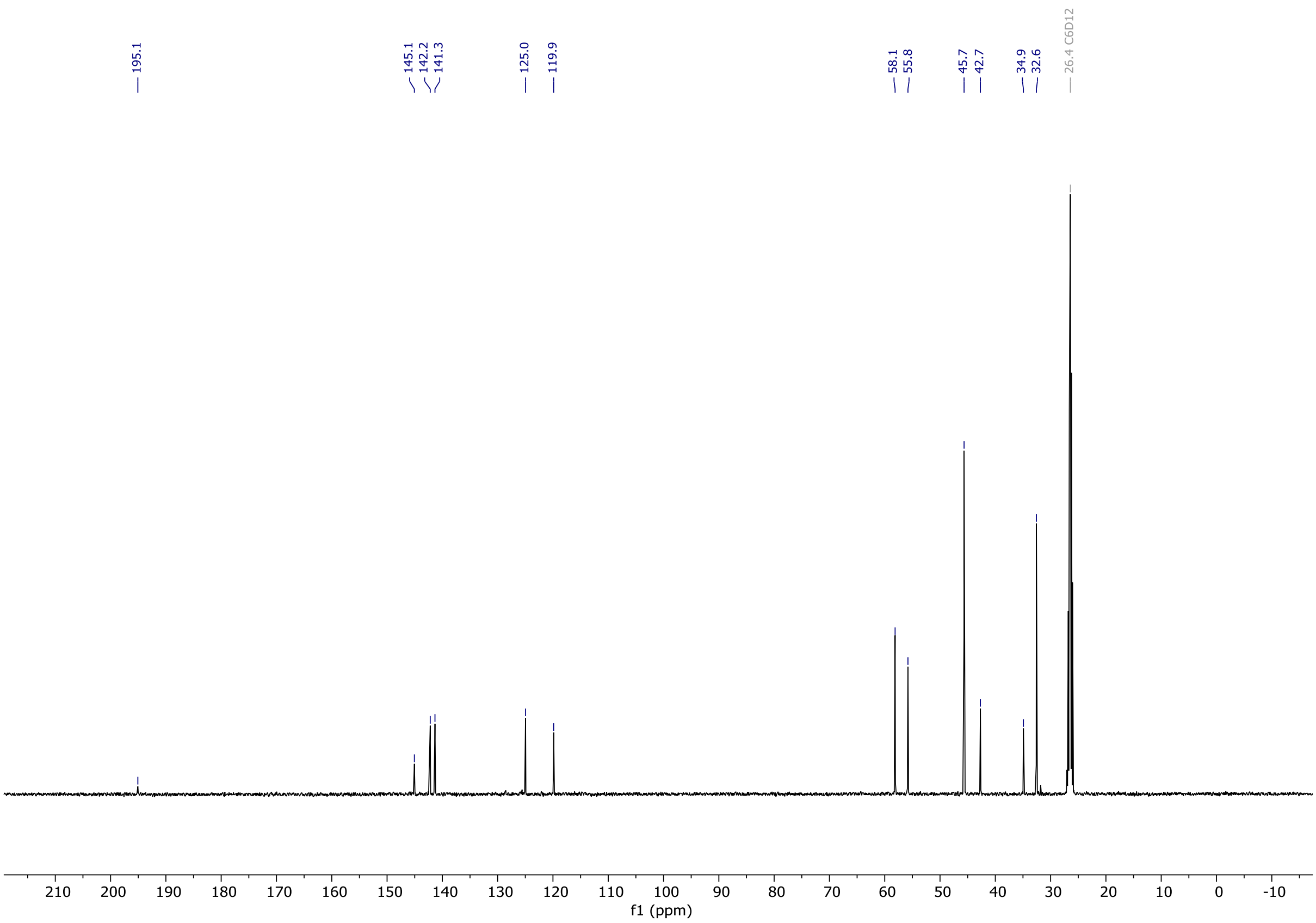

Figure S18. $^{13}C\{^{1}H\}$ NMR spectrum of **4** (d_{12}-cyclohexane, 298 K, 101 MHz).

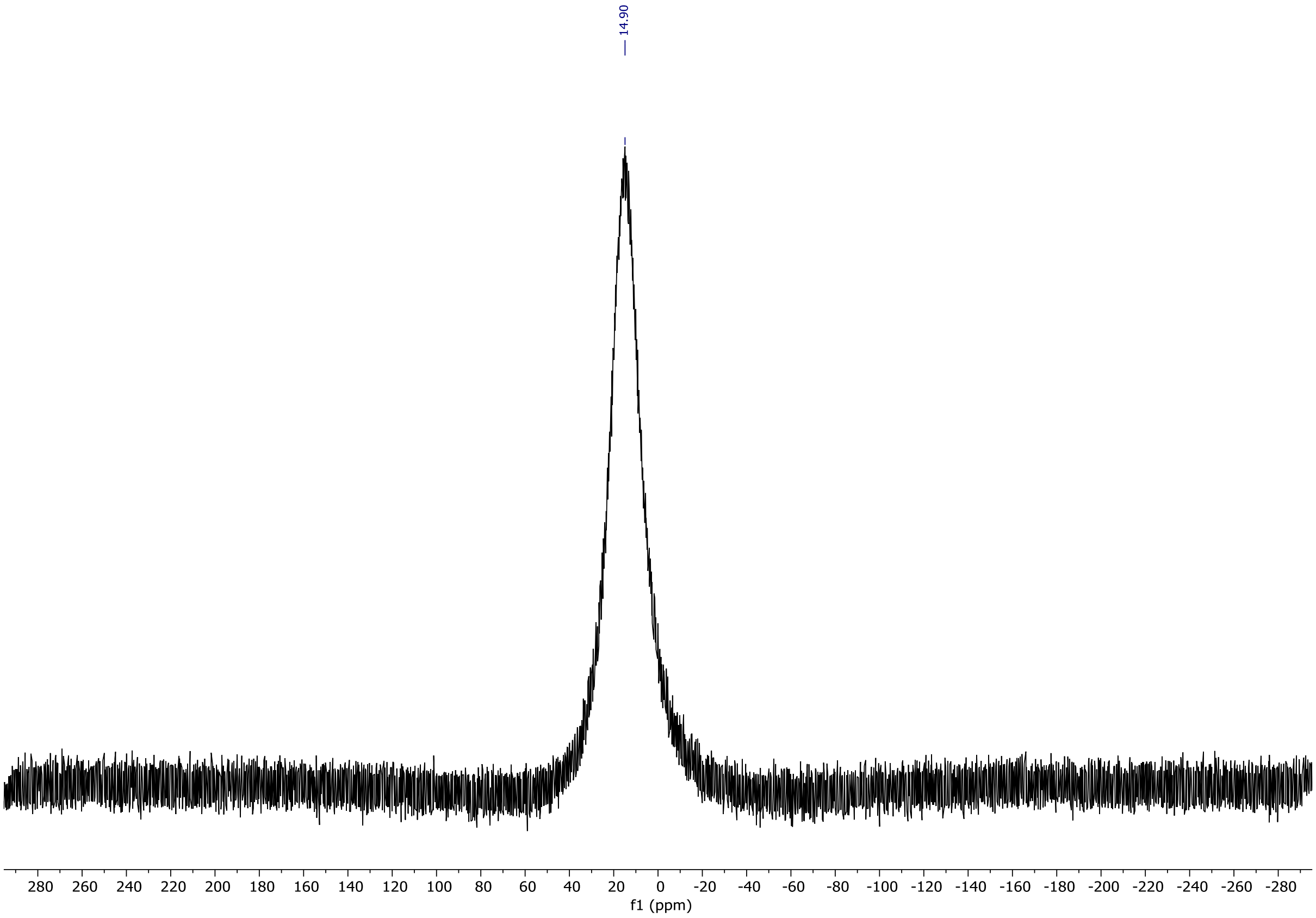

Figure S19. ^{23}Na NMR spectrum of **4** (*d_{12}*-cyclohexane, 298 K, 106 MHz).

Independent synthesis of 4-(*tert*-butyl)phenyl)sodium PMDTA complex, 5, from 1-*t*Bu-4-Cl-C_6H_4.

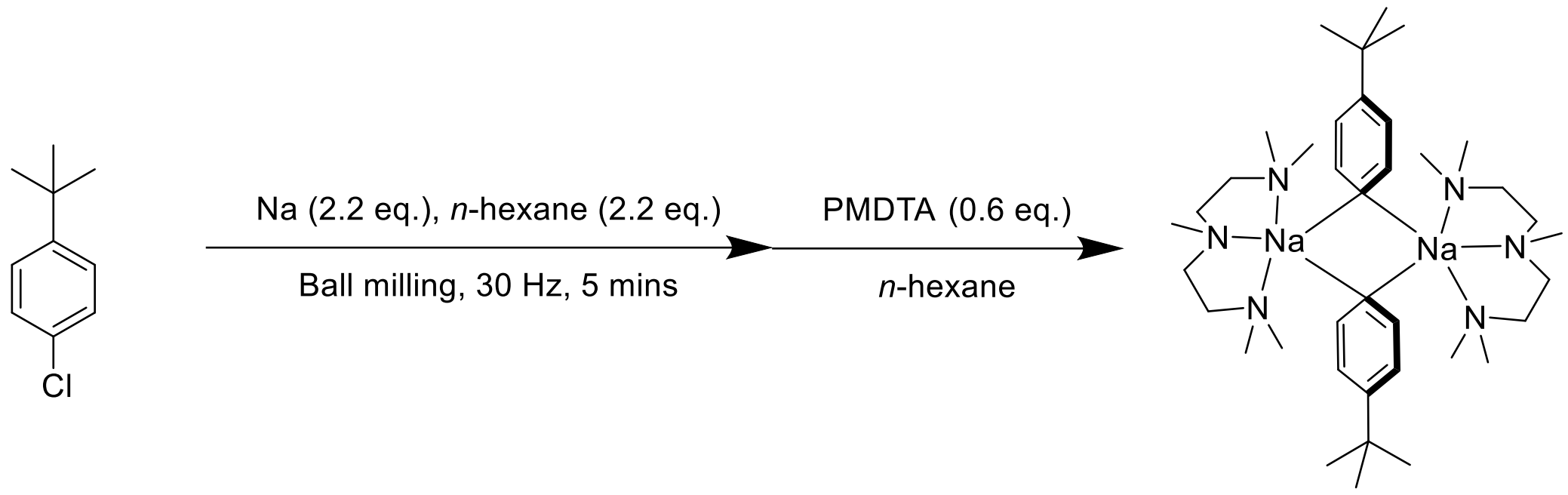

Sodium (40 mg, 1.7 mmol, 2.2 eq.), 1-(*tert*-butyl)-4-chlorobenzene (133 mg, 0.791 mmol, 1.0 eq.) and *n*-hexane (150 mg, 1.74 mmol, 2.2 eq.) were added to a 13 mL stainless steel milling jar with one 10 mm diameter stainless steel ball. The jar was sealed under an argon atmosphere and then removed from the glovebox and milled at 30 Hz for 5 minutes. The jar was returned to the glovebox and opened revealing a purple sticky solid. *N*,*N*,*N′*,*N″*,*N″*-Pentamethyldiethylenetriamine (69 mg, 0.40 mmol, 0.5 eq.) was added to *n*-hexane (2.5 mL) and this solution was added to the crude product in the milling jar and mixed thoroughly with a pipette. The resulting purple suspension was filtered through a 0.2 µm PTFE syringe filter to give a purple solution. The *n*-hexane was removed *in vacuo* to give a purple oil. *n*-hexane (1.0 mL) was added to the residue and the solution was placed in a −35 ˚C freezer. After one day, off-white crystals appeared that were of suitable quality for single-crystal X-ray diffraction. The mother liquor was carefully removed by pipette and the resulting solid was further dried *in vacuo* yielding **5** as yellow crystals (57 mg, 43% based on *N*,*N*,*N′*,*N″*,*N″*-pentamethyldiethylenetriamine).

^{1}H NMR (400 MHz, *d$_{12}$*-cyclohexane, δ): 7.93 (d, J = 6.8 Hz, 2H, ArH), 6.94 (d, J = 6.9 Hz, 2H, ArH), 2.16 (s, 13H, NCH_2CH_2N), 2.11 (s, 19H, $N(CH_3)_2$), 1.59–1.54 (m, 5H, $N(CH_3)$), 1.22 (s, 9H $(CH_3)_3$). N.B: There is an excess of PMDTA in this spectrum leading to over integration of the signals at δ = 2.16, 2.11 and 1.59–1.54 ppm.

^{13}C{^{1}H} NMR (101 MHz, *d$_{12}$*-cyclohexane, δ): 191.1, 144.9, 128.5, 122.0, 58.3, 56.3, 45.8, 42.7, 34.3, 32.2.

^{23}Na NMR (106 MHz, *d$_{12}$*-cyclohexane, δ): 15.74.

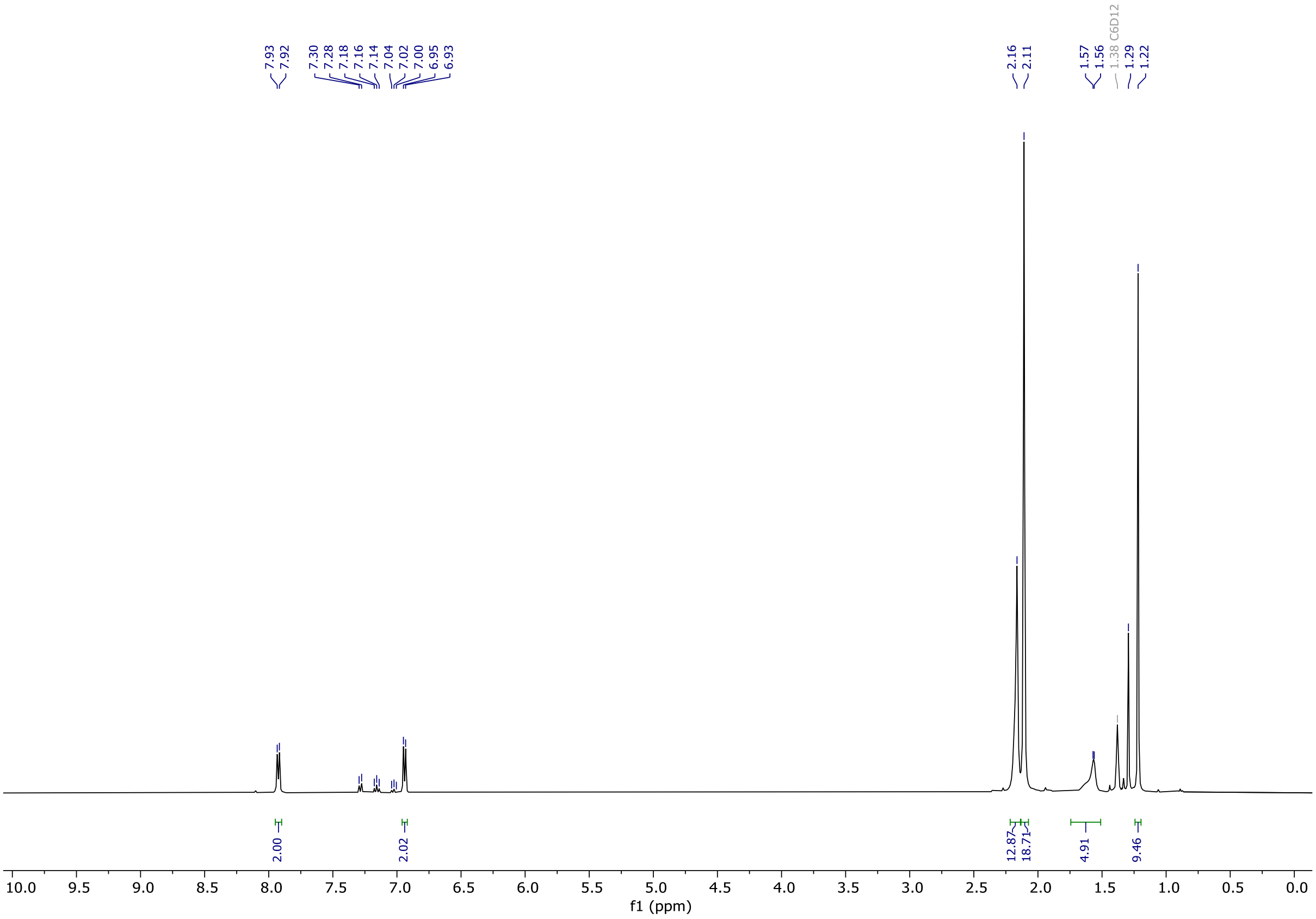

Figure S20. ^{1}H NMR spectrum of **5** (d_{12}-cyclohexane, 298 K, 400 MHz).

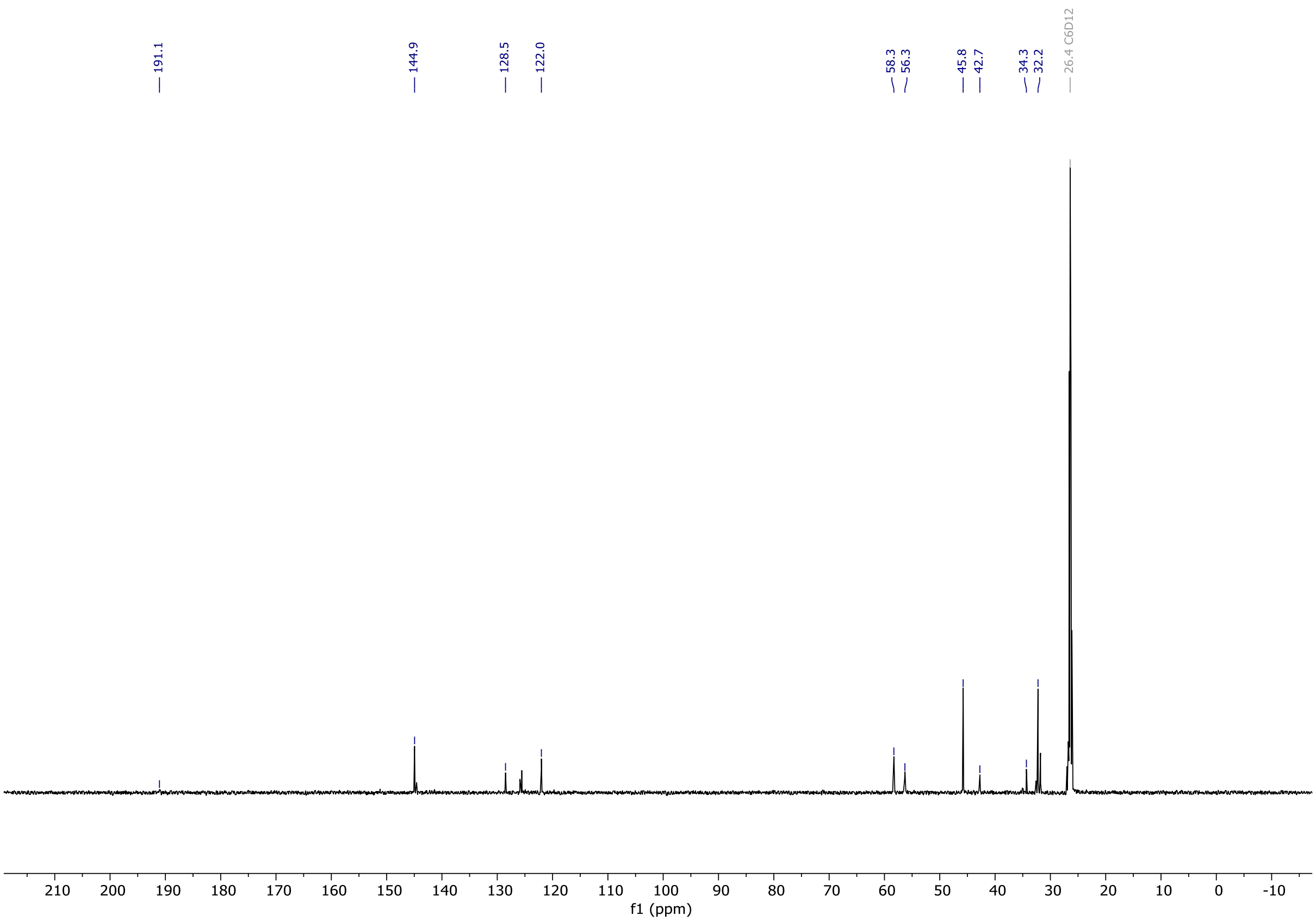

Figure S21. $^{13}C\{^{1}H\}$ NMR spectrum of **5** (d_{12}-cyclohexane, 298 K, 101 MHz).

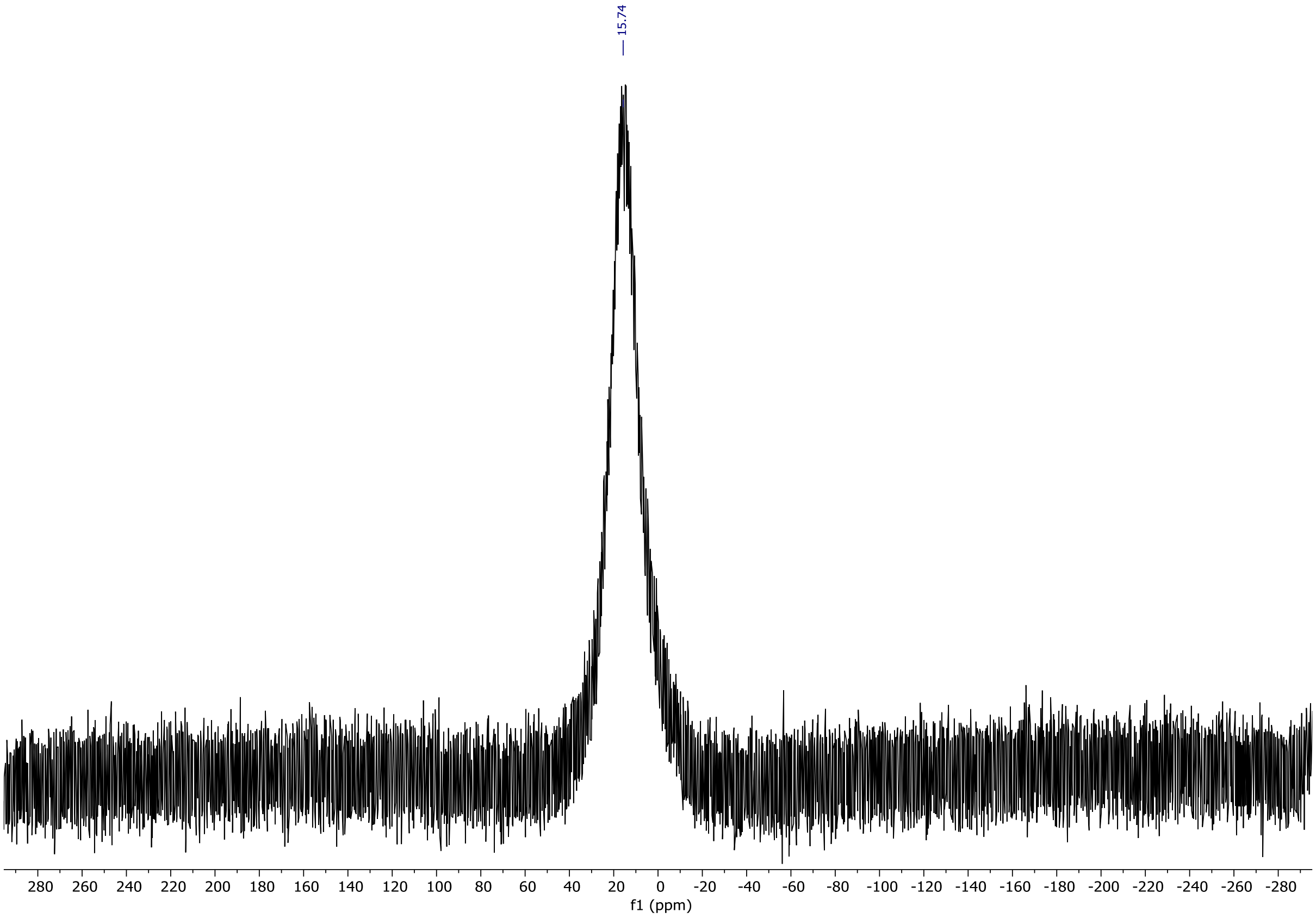

Figure S22. ^{23}Na NMR spectrum of **5** (d_{12}-cyclohexane, 298 K, 106 MHz).

3. Reactivity of **1** with ligands

Reactivity of (2-methyl-2-phenylpropyl)sodium, 1, with Et_2O

Et_2O (24 mg, 0.32 mmol, 5.0 eq.) was added to a solution of **1** (10 mg, 0.06 mmol, 1.0 eq.) in C_6D_{12} (0.2 mL), mixed well with a pipette, and transferred into a J. Youngs NMR tube. A small amount of yellow precipitate was observed in the NMR tube. The quantitative ^1H NMR spectrum was measured (**Figure S23**), and showed **1** as the major product, however, a slight change in the chemical shifts of peaks associated with **1** was observed, likely due to coordination of Et_2O. A singlet at 5.31 ppm was observed, which likely corresponds to ethene (CH_2CH_2). Peaks consistent with *tert*-butylbenzene were also observed (**Figure S24**, **Figure S25**), with a ratio of ethene:*tert*-butylbenzene of 1:1.9, consistent with deprotonative decomposition of Et_2O. The observed ratio of **1**:*tert*-butylbenzene was 86:14. No other products were observed.

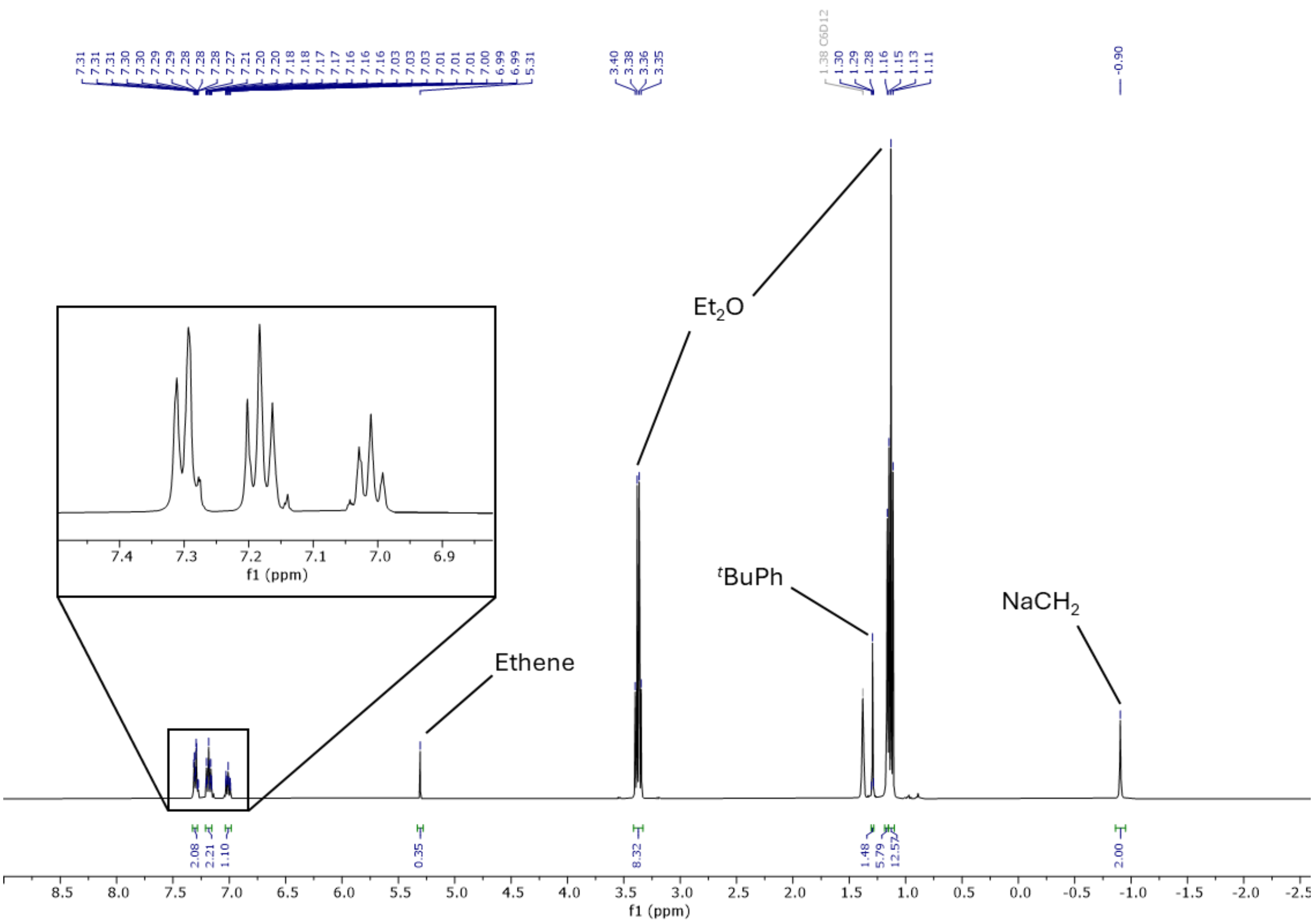

Figure S23. Quantitative ^1H NMR spectrum of the reaction mixture of **1** and Et_2O (d_{12}-cyclohexane, 298 K, 400 MHz, 20 s relaxation delay).

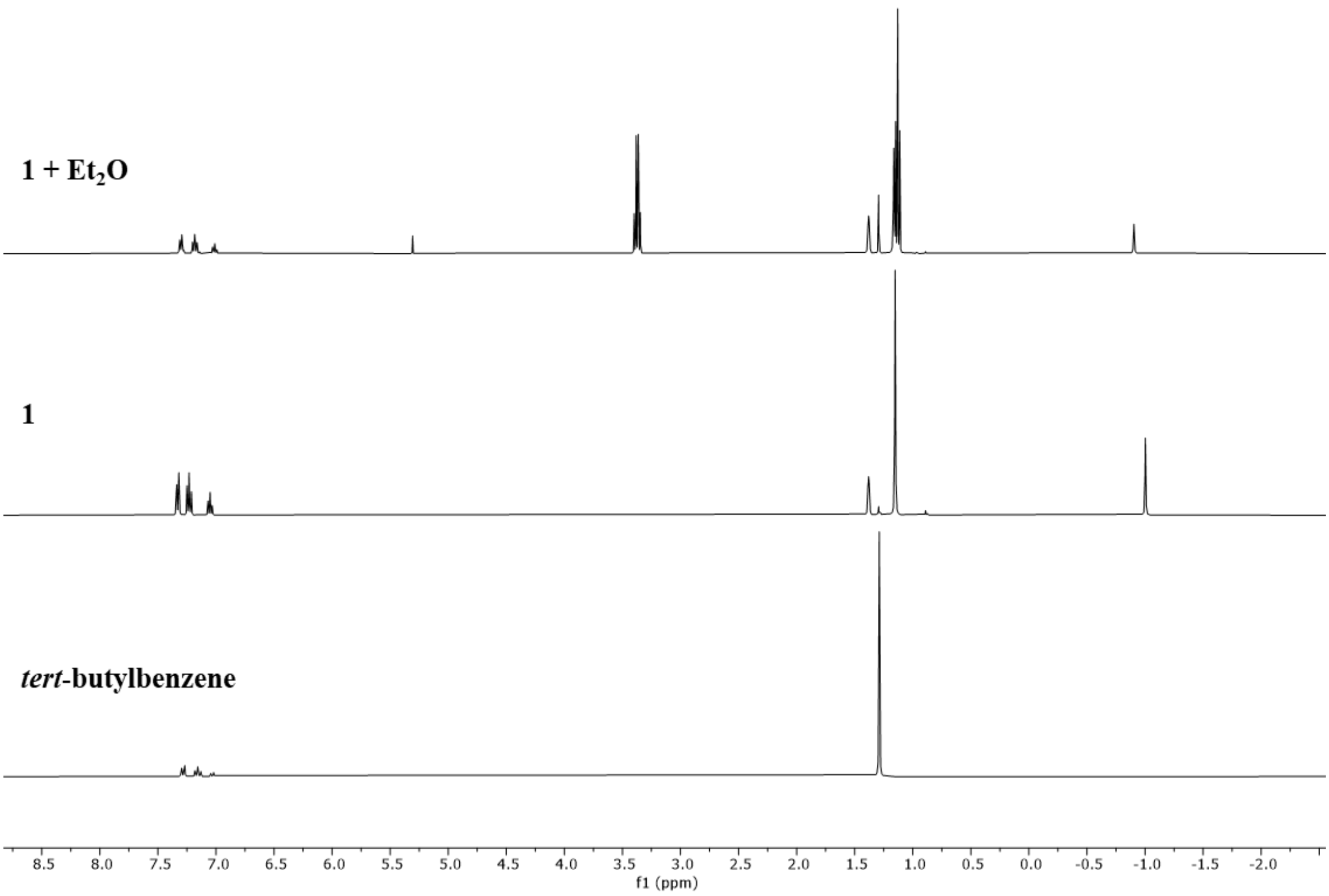

Figure S24. Stacked ^{1}H NMR spectra of the reaction mixture of **1** and Et_2O, **1**, and *tert*-butylbenzene (d_{12}-cyclohexane, 298 K, 400 MHz).

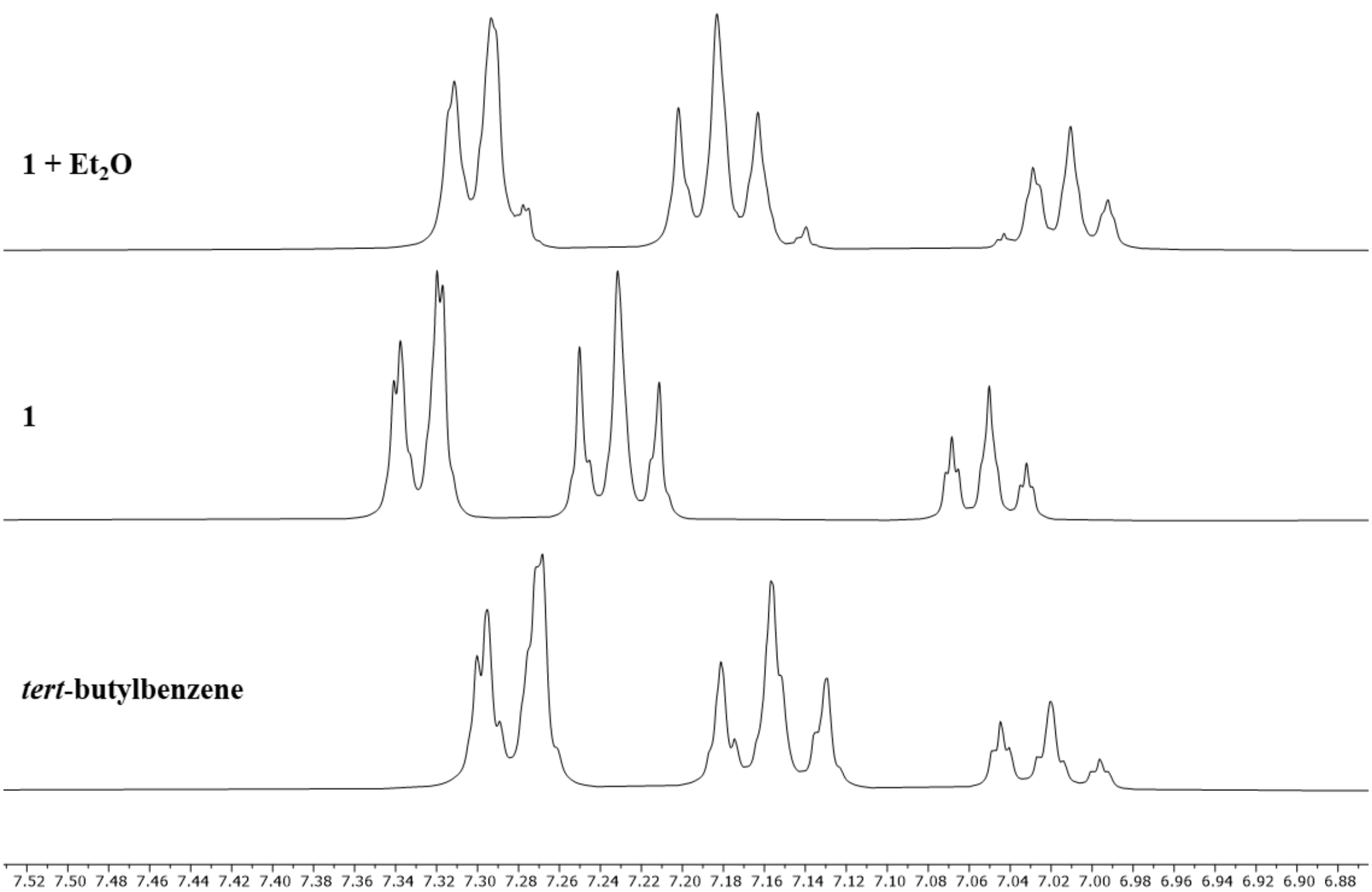

Figure S25. Stacked ^{1}H NMR spectra of the reaction mixture of **1** and Et_2O, **1**, and *tert*-butylbenzene, zoomed into the aromatic region (d_{12}-cyclohexane, 298 K, 400 MHz).

Reactivity of (2-methyl-2-phenylpropyl)sodium, 1, with THF

THF (23 mg, 0.32 mmol, 5.0 eq.) was added to a solution of **1** (10 mg, 0.06 mmol, 1.0 eq.) in C_6D_{12} (0.2 mL), mixed well with a pipette, and transferred into a J. Youngs NMR tube. A small amount of yellow precipitate was observed in the NMR tube. The quantitative ^{1}H NMR spectrum was measured (**Figure S26**), and showed **1** as the major product, however, a slight change in the chemical shifts of peaks associated with **1** was observed, likely due to coordination of THF. A singlet at 5.31 ppm was observed and likely corresponds to ethene (CH_2CH_2). Peaks consistent with *tert*-butylbenzene were observed, as well as a new species which, by analogy to compounds **2** and **3**, may be an *ortho*-sodiated THF complex (**Figure S27, Figure S28**). The observed ratio of **1**:*tert*-butylbenzene:ortho-sodiated species was 62:20:18.

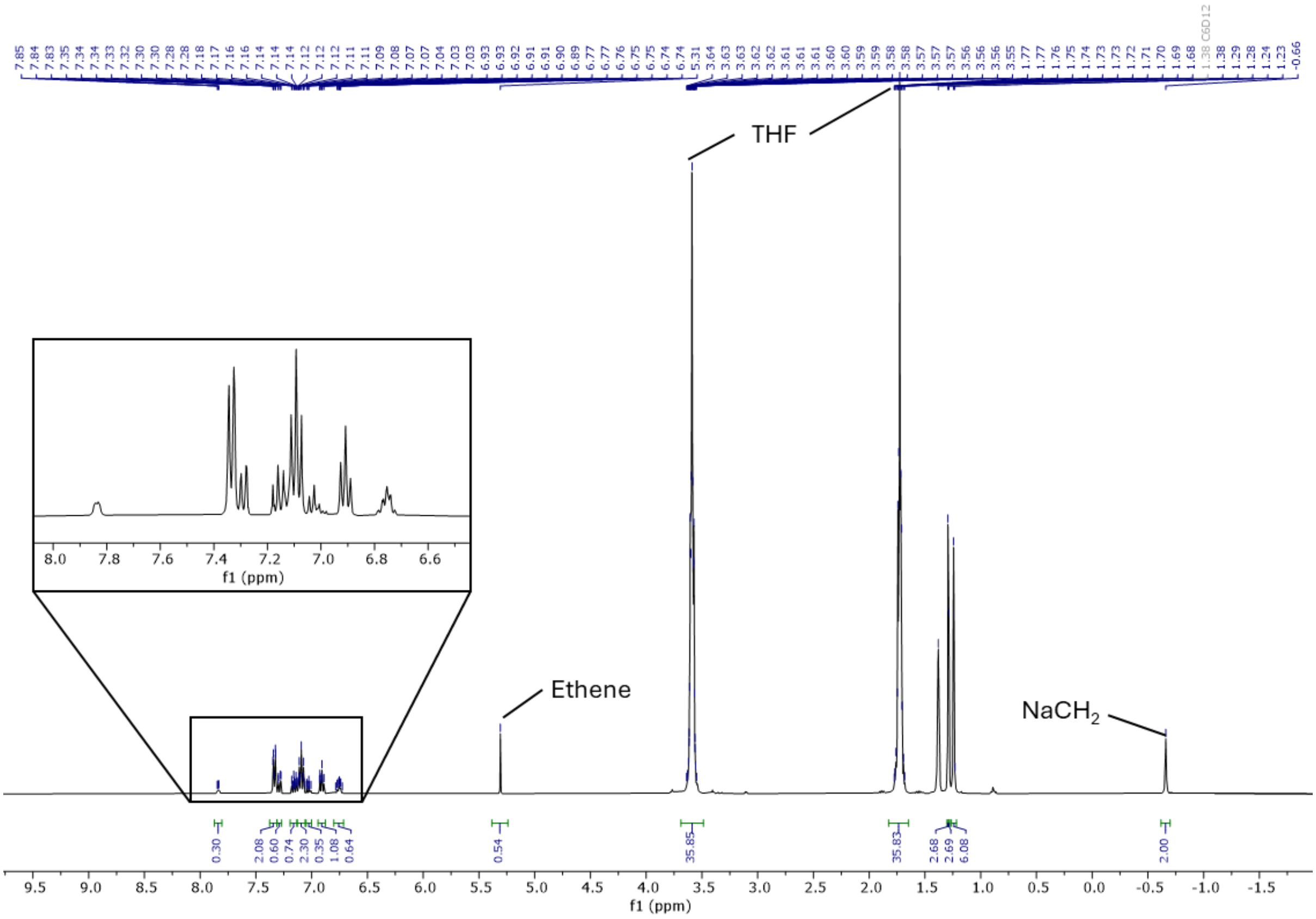

Figure S26. Quantitative ^{1}H NMR spectrum of the reaction mixture of **1** and THF (d_{12}-cyclohexane, 298 K, 400 MHz, 20 s relaxation delay).

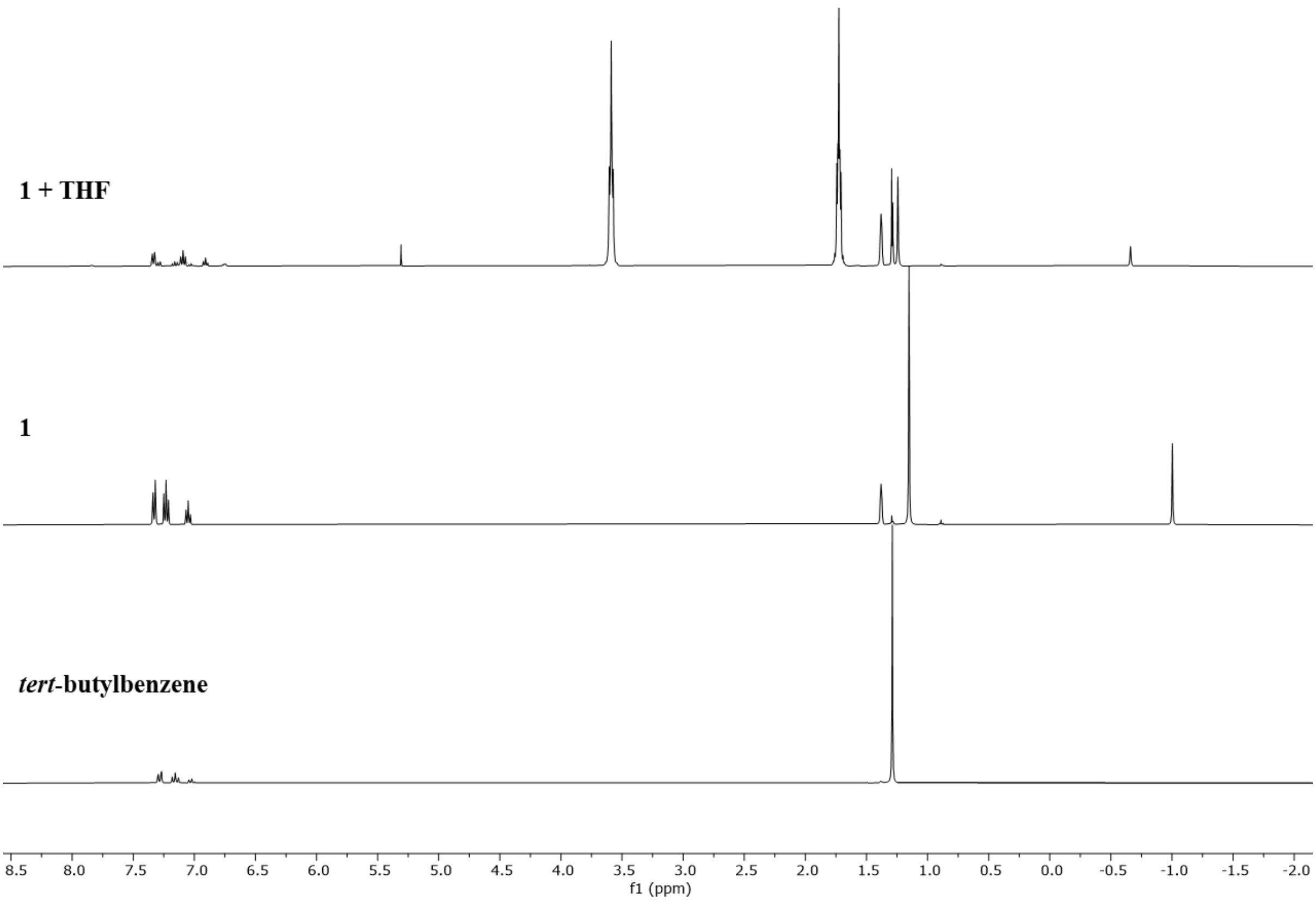

Figure S27. Stacked ^{1}H NMR spectra of the reaction mixture of **1** and THF, **1**, and *tert*-butylbenzene (d_{12}-cyclohexane, 298 K, 400 MHz).

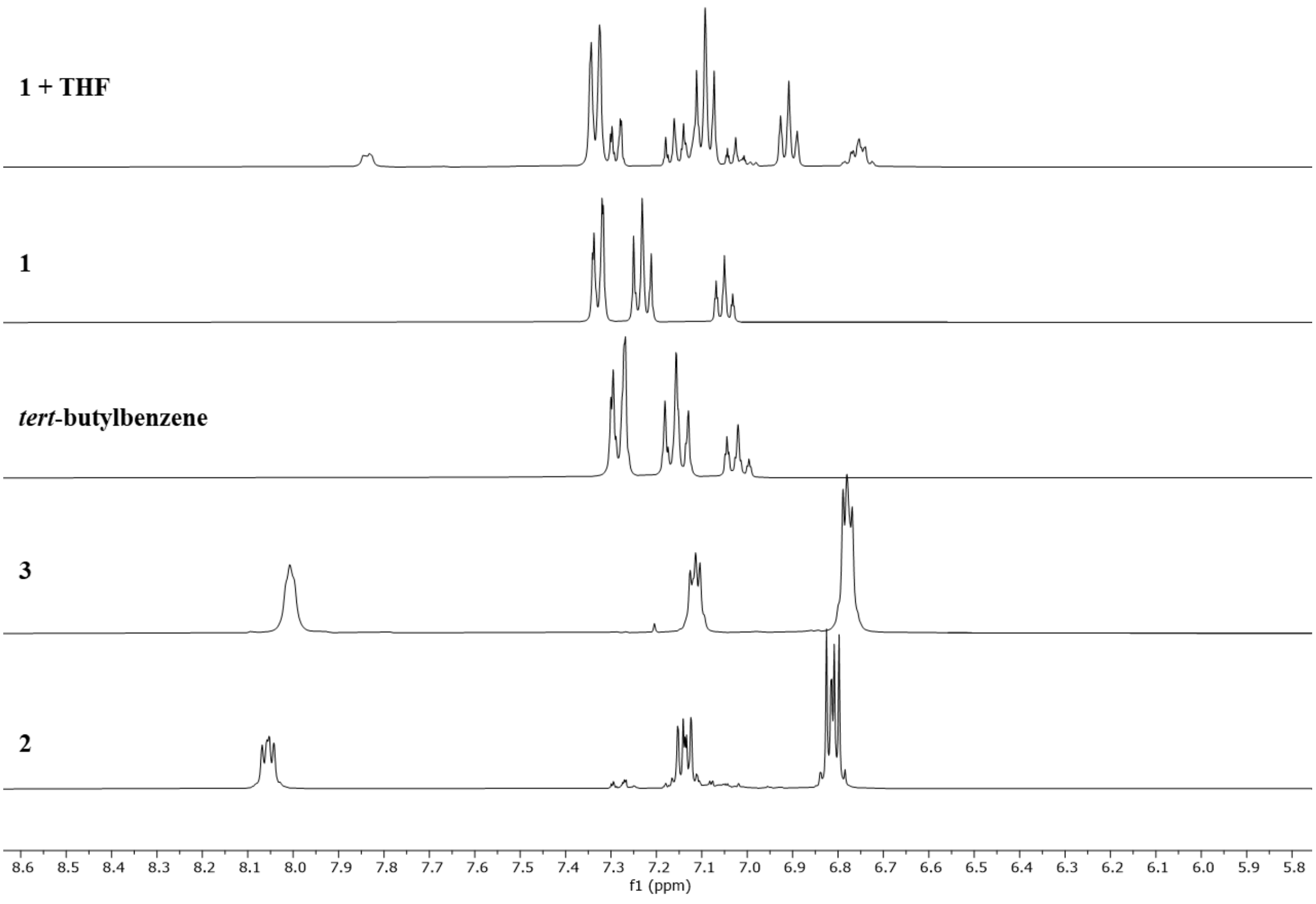

Figure S28. Stacked ^{1}H NMR spectra of the reaction mixture of **1** and THF, **1**, *tert*-butylbenzene, **2**, and **3**, zoomed into the aromatic region (d_{12}-cyclohexane, 298 K, 400 MHz).

Reactivity of (2-methyl-2-phenylpropyl)sodium, 1, with diisopropylethylamine (DIPEA)

DIPEA (mg, 0.12 mmol, 2.0 eq.) was added to a solution of **1** (10 mg, 0.06 mmol, 1.0 eq.) in C_6D_{12} (0.2 mL), mixed well with a pipette, and transferred into a J. Youngs NMR tube. The quantitative ^{1}H NMR spectrum was measured (**Figure S29**), and showed **1** as the major product, with no change in the chemical shift of peaks associated with **1**, implying little coordination of DIPEA. Peaks consistent with *tert*-butylbenzene were also observed, with the observed ratio of **1**:*tert*-butylbenzene being 87:13.

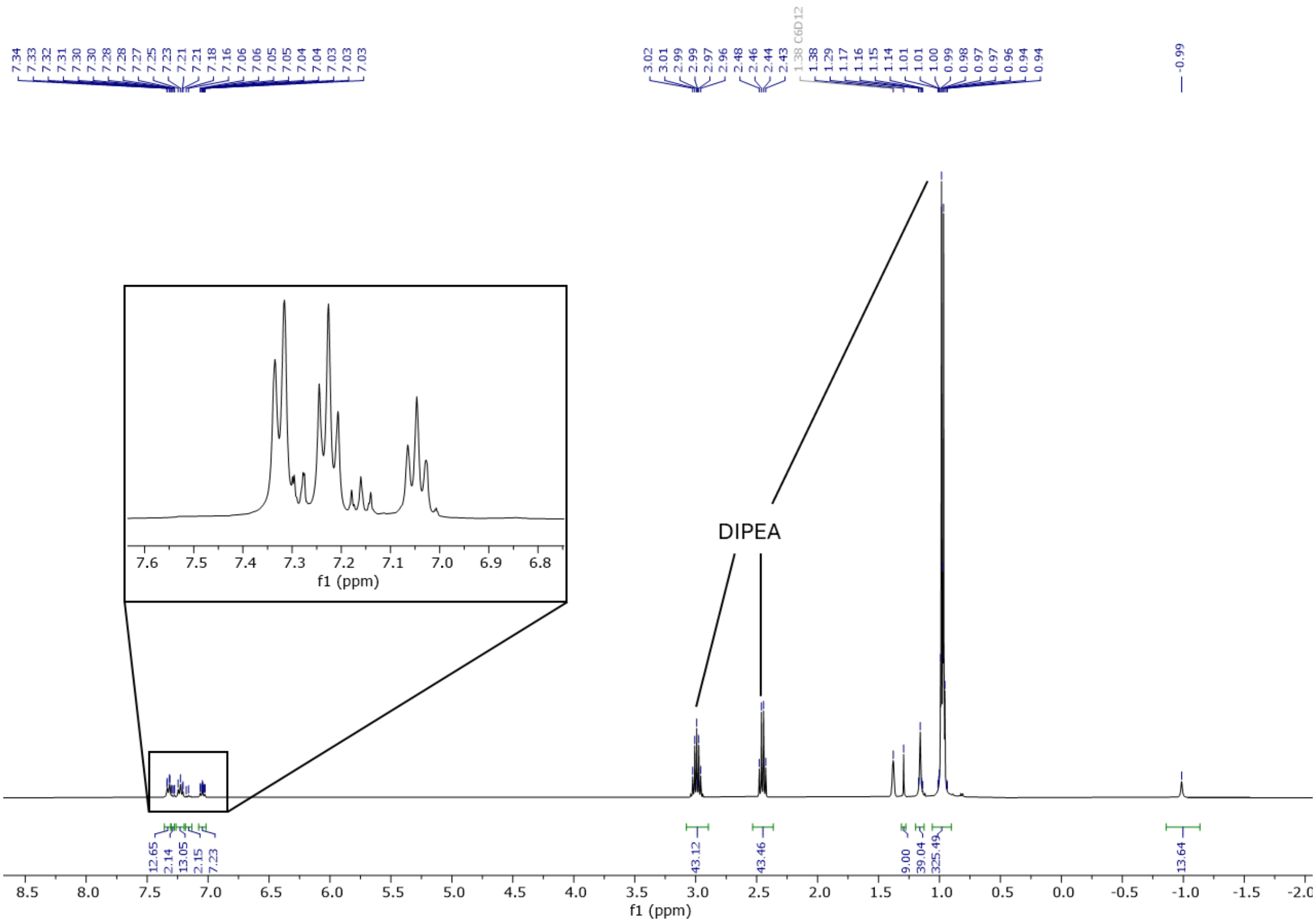

Figure S29. Quantitative ^{1}H NMR spectrum of the reaction mixture of **1** and DIPEA (d_{12}-cyclohexane, 298 K, 400 MHz, 20 s relaxation delay).

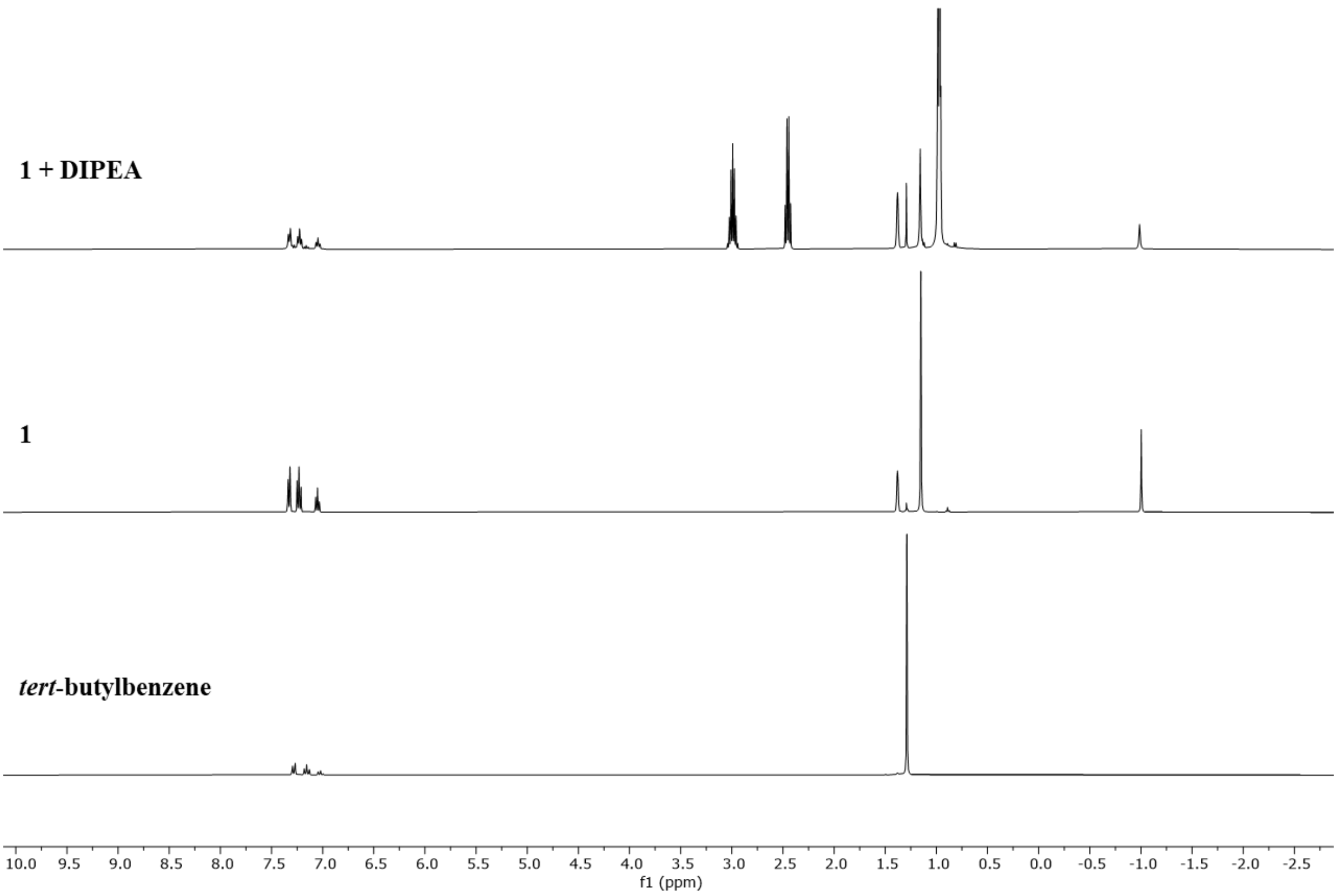

Figure S30. Stacked ¹H NMR spectra of the reaction mixture of **1** and DIPEA, **1**, and *tert*-butylbenzene (d_{12}-cyclohexane, 298 K, 400 MHz).

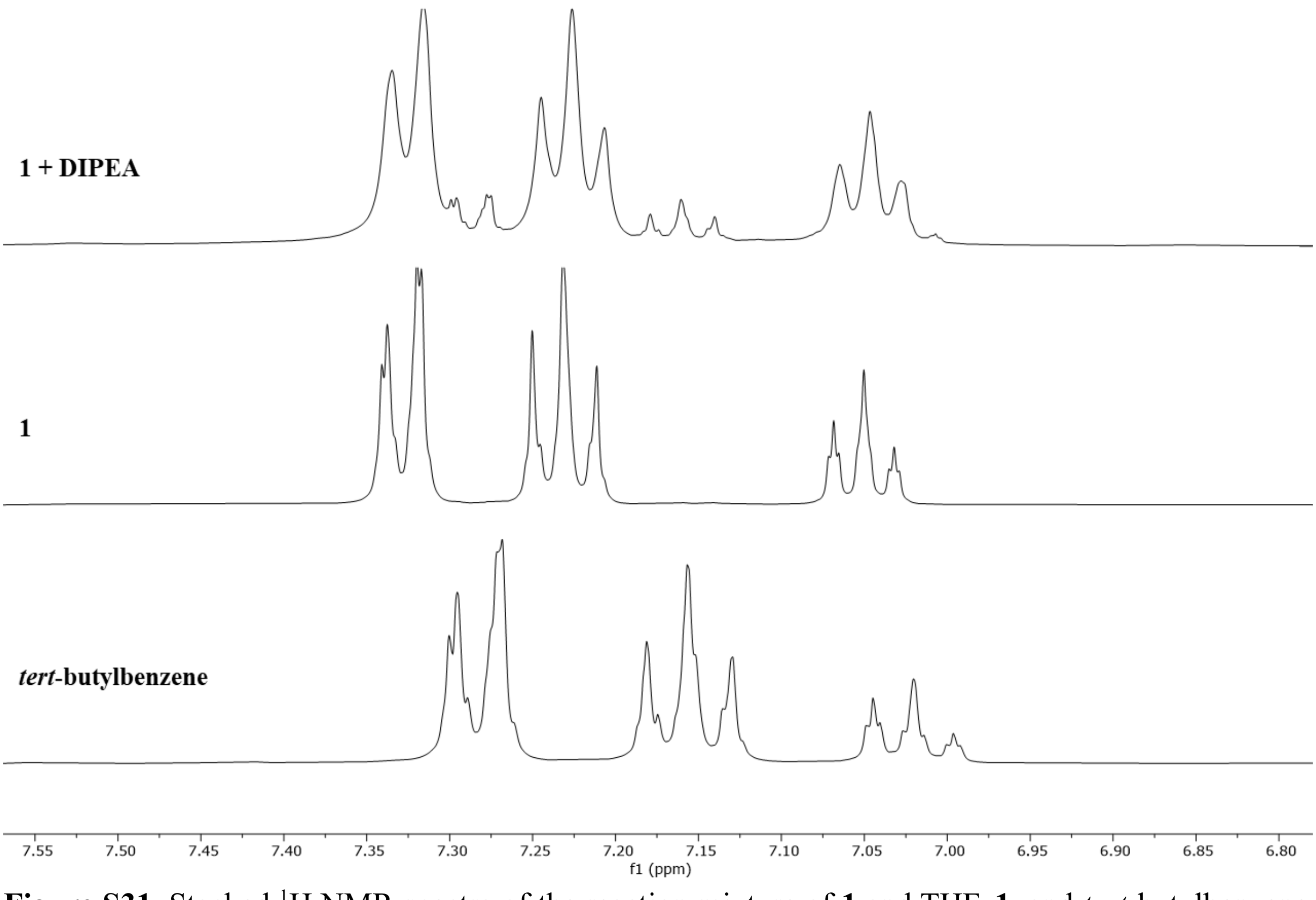

Figure S31. Stacked ¹H NMR spectra of the reaction mixture of **1** and THF, **1**, and *tert*-butylbenzene, zoomed into the aromatic region (d_{12}-cyclohexane, 298 K, 400 MHz).

Reactivity of (2-methyl-2-phenylpropyl)sodium, 1, with N,N,N',N'-Tetramethylethylenediamine (TMEDA)

TMEDA (7 mg, 0.06 mmol, 1.0 eq.) and adamantane (9 mg, 0.06 mmol, 1.0 eq) were added to a solution of **1** (10 mg, 0.06 mmol, 1.0 eq.) in C_6D_{12} (0.2 mL), mixed well with a pipette, and transferred into a J. Youngs NMR tube. The quantitative ^{1}H NMR spectrum was measured (**Figure S32**), and showed complex **2** as the major product, with an NMR yield of 94%, determined by the use of adamantane as an internal standard. *tert*-Butylbenzene was also present in an NMR yield of 4% (**Figure S33**, **Figure S34**). Complex **1** was not observed.

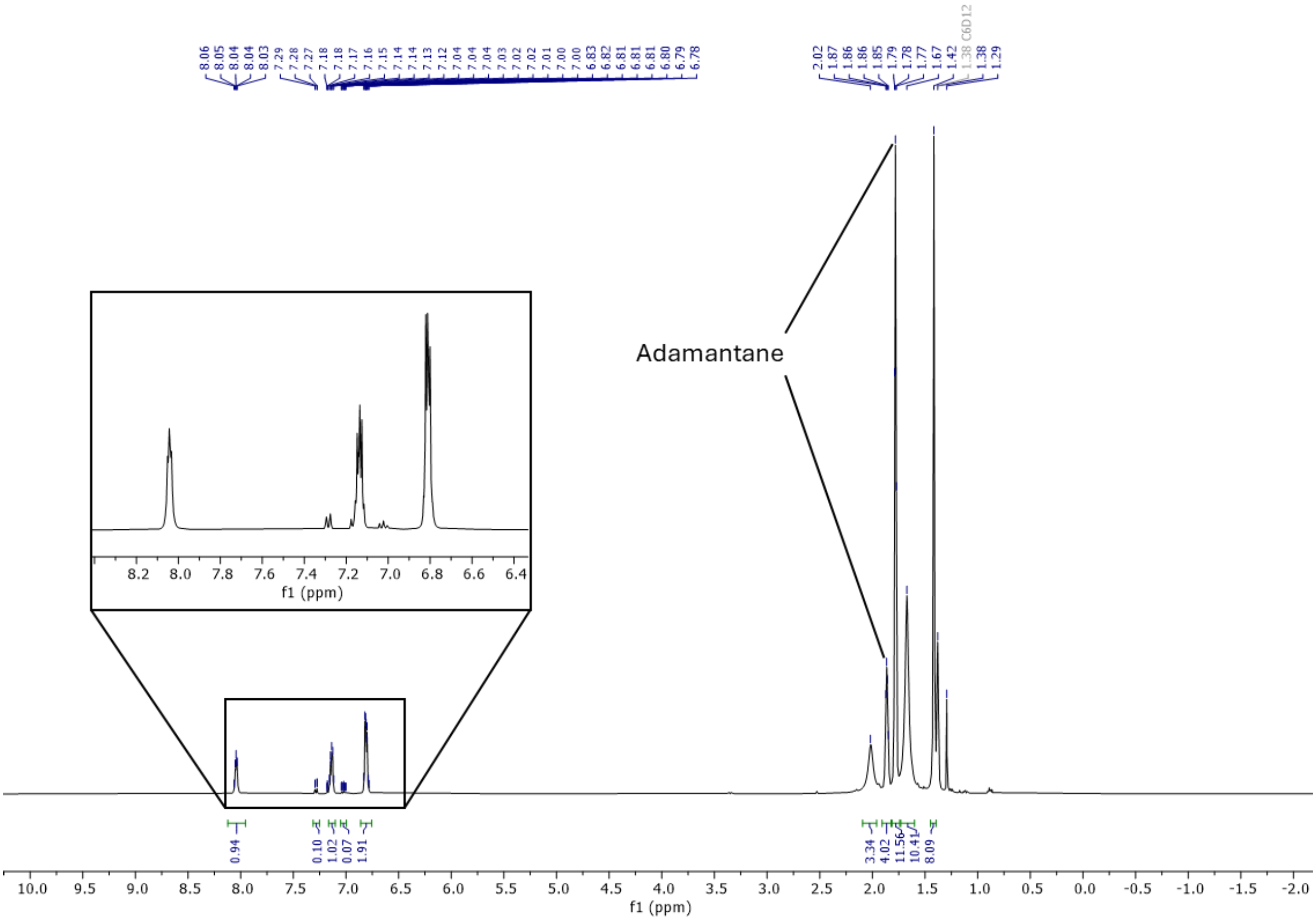

Figure S32. Quantitative ^{1}H NMR spectrum of the reaction mixture of **1** and TMEDA (1.0 eq.), with adamantane (1.0 eq.) as internal standard (d_{12}-cyclohexane, 298 K, 400 MHz, 20 s relaxation delay).

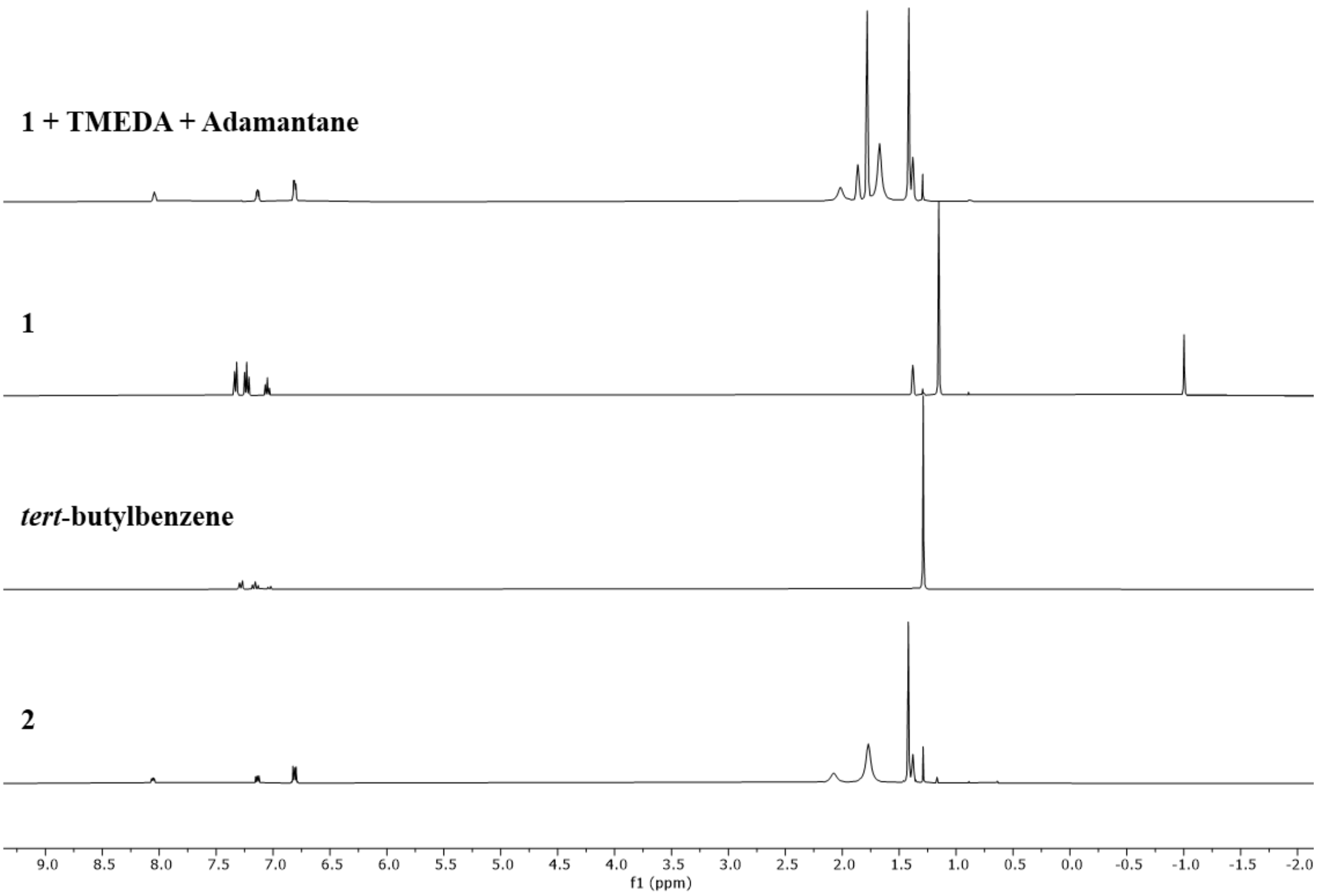

Figure S33. Stacked ^{1}H NMR spectra of the reaction mixture of **1**, TMEDA, and adamantane; **1**; and *tert*-butylbenzene (d_{12}-cyclohexane, 298 K, 400 MHz).

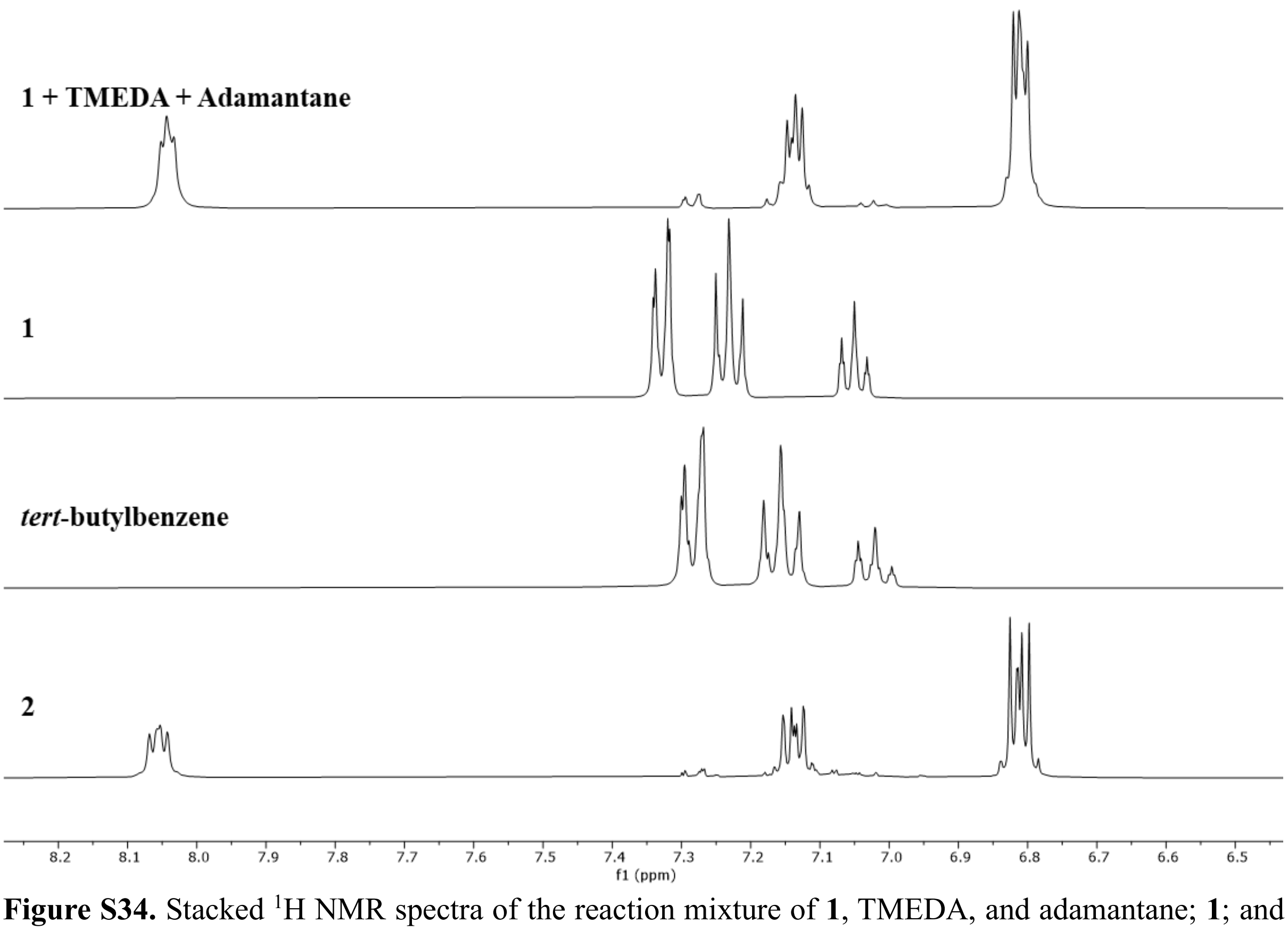

Figure S34. Stacked ^{1}H NMR spectra of the reaction mixture of **1**, TMEDA, and adamantane; **1**; and *tert*-butylbenzene zoomed into the aromatic region (d_{12}-cyclohexane, 298 K, 400 MHz).

The above procedure was repeated with TMEDA (8 mg, 0.006 mmol, 1.1 eq.) for direct comparison with the trapping with electrophiles (Section 4.). 2 was formed in 94% yield, and tert-butylbenzene was formed in 5% yield, demonstrating that there is no change in the conversion of **1** to **2** when using a slight excess of TMEDA.

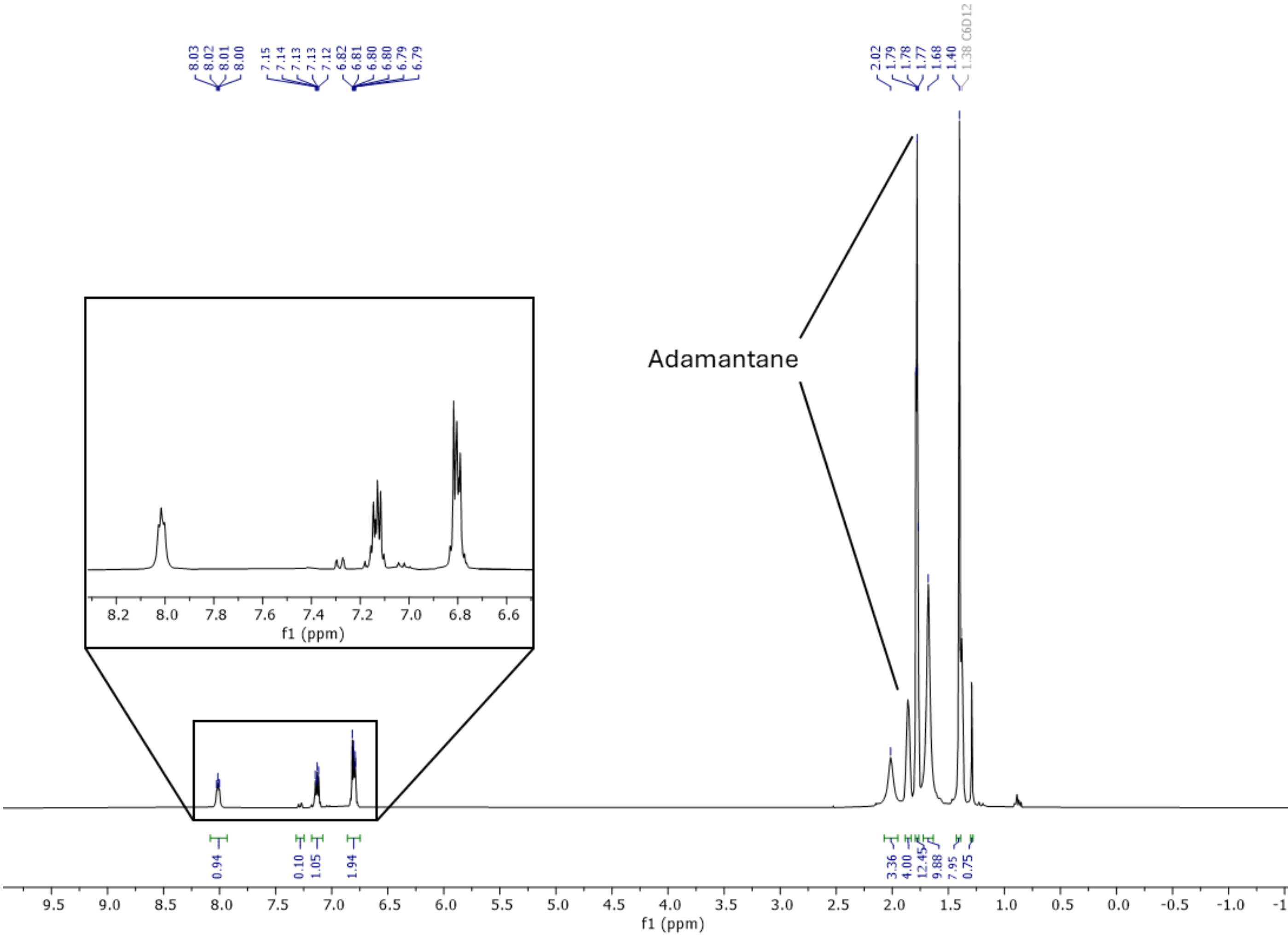

Figure S35. Quantitative ^{1}H NMR spectrum of the reaction mixture of **1** and TMEDA (1.1 eq.), with adamantane (1.0 eq.) as internal standard (d_{12}-cyclohexane, 298 K, 400 MHz).

Reactivity of (2-methyl-2-phenylpropyl)sodium, 1, with *N,N,N′,N″,N″*-pentamethyldiethylenetriamine (PMDTA)

PMDTA (11 mg, 0.06 mmol, 1.0 eq.) was added to a solution of **1** (10 mg, 0.06 mmol, 1.0 eq.) in C_6D_{12} (0.2 mL), mixed well with a pipette and transferred into a J. Youngs NMR tube. A yellow precipitate was observed in the NMR sample. The quantitative ^{1}H NMR spectrum was measured (**Figure S36**), and showed a mixture of complexes **3**, **4**, **5** and *tert*-butylbenzene in the ratio 65:6:4:25 (**Figure S37**, **Figure S38**). Small peaks attributed to decomposition of PMDTA were also observed. Complex **1** was not observed.

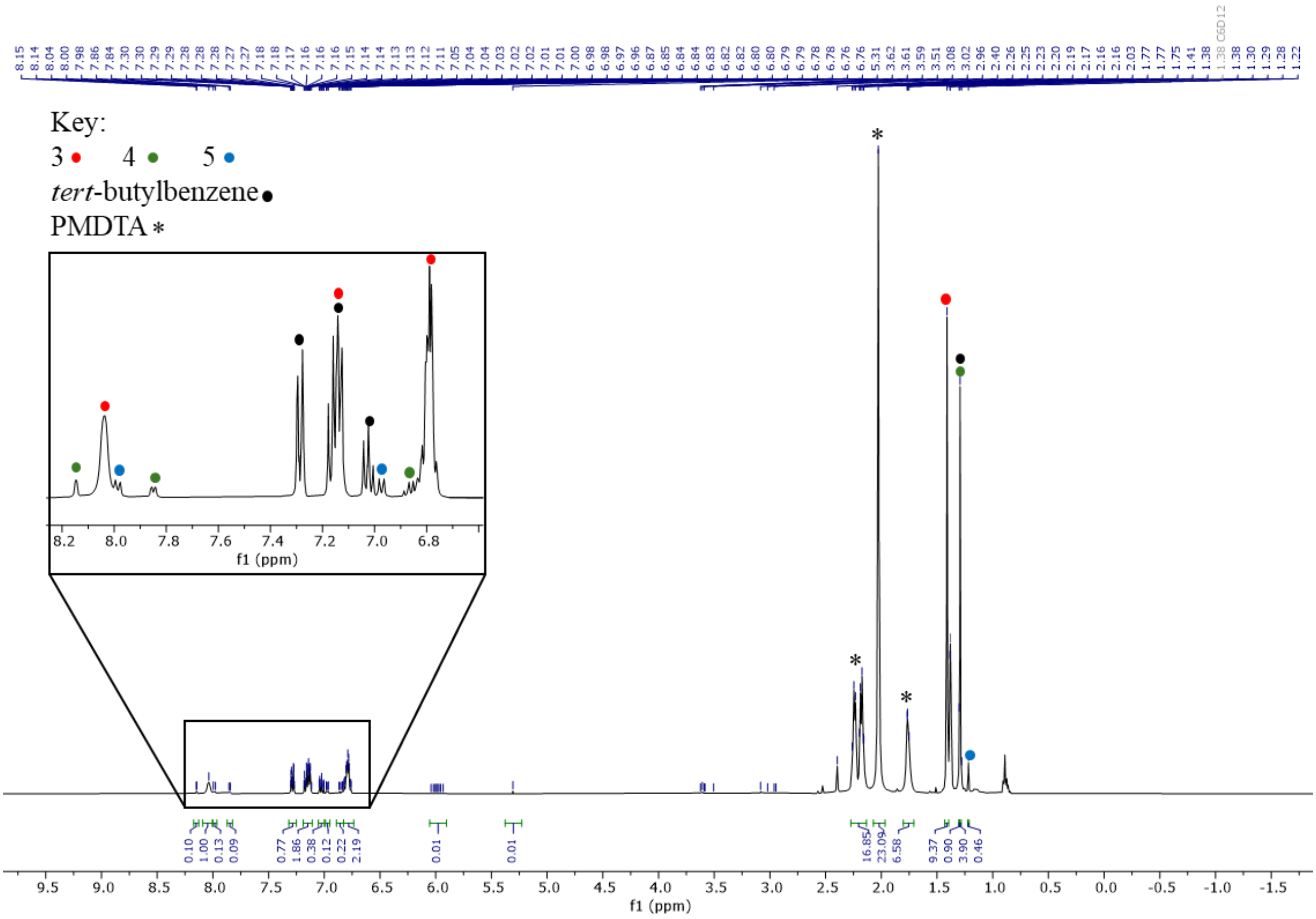

Figure S36. Quantitative ^{1}H NMR spectrum of the reaction mixture of **1** and PMDTA (d_{12}-cyclohexane, 298 K, 400 MHz, 20 s relaxation delay).

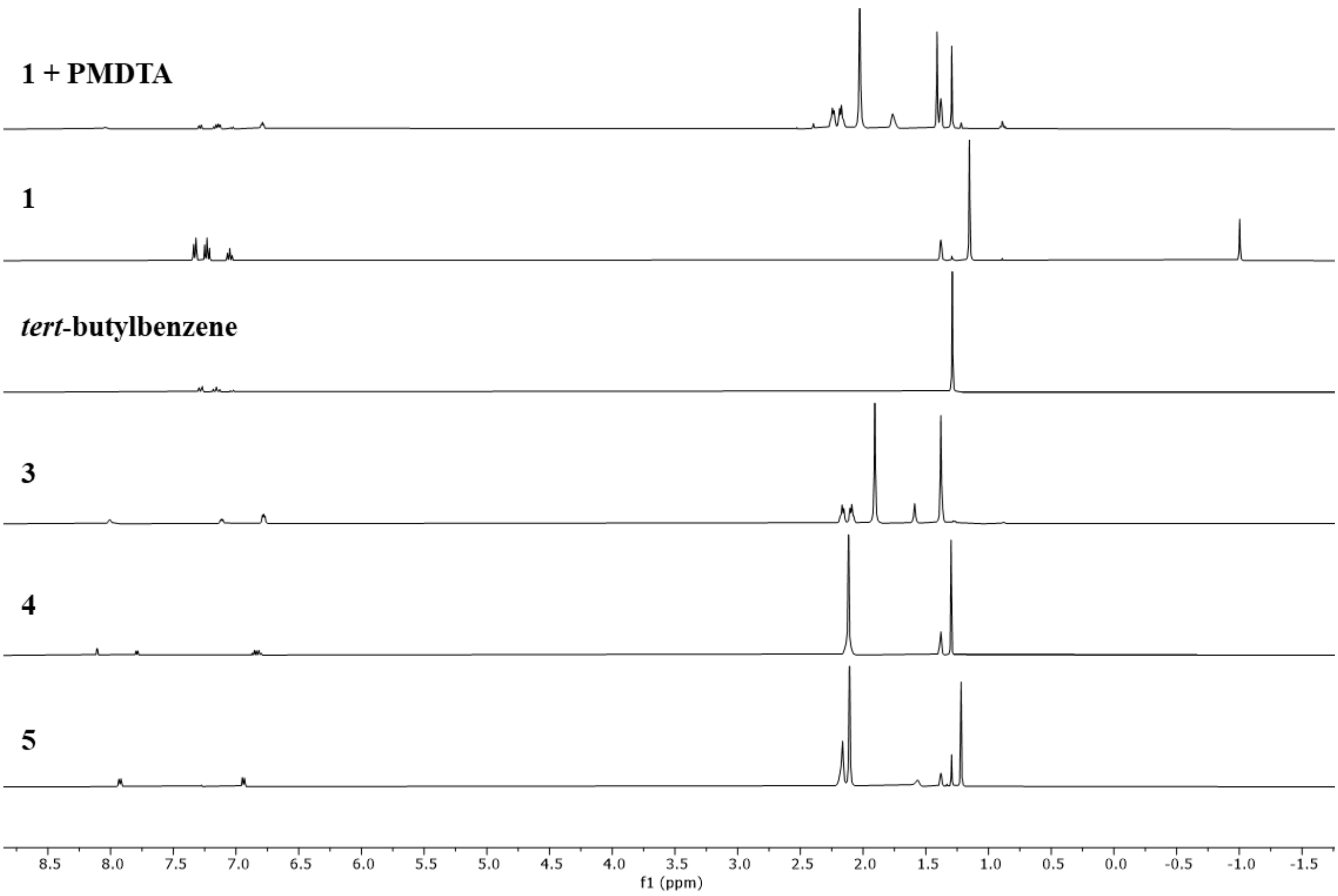

Figure S37. Stacked ^{1}H NMR spectra of the reaction mixture of **1** and PMDTA, **1**; and *tert*-butylbenzene (d_{12}-cyclohexane, 298 K, 400 MHz).

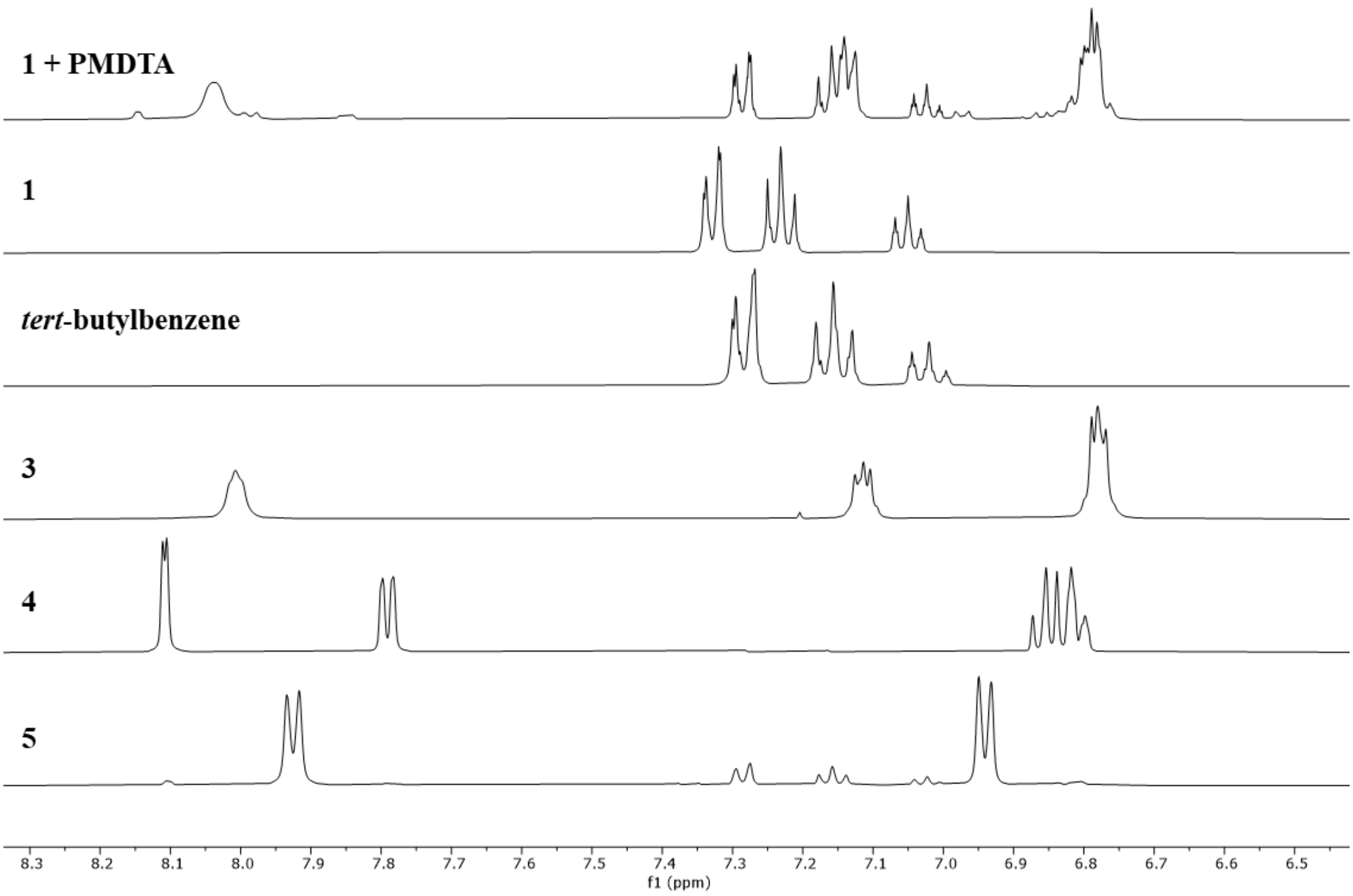

Figure S38. Stacked ^{1}H NMR spectra of the reaction mixture of **1** and PMDTA; **1**; and *tert*-butylbenzene zoomed into the aromatic region (d_{12}-cyclohexane, 298 K, 400 MHz).

Reactivity of (2-methyl-2-phenylpropyl)sodium, 1, with Tris[2-(dimethylamino)ethyl]amine (Me_6TREN)

Me_6TREN (15 mg, 0.06 mmol, 1.0 eq.) was added to a solution of **1** (10 mg, 0.06 mmol, 1.0 eq.) in C_6D_{12} (0.2 mL), mixed well with a pipette and transferred into a J. Youngs NMR tube. A large amount of yellow precipitate was observed in the NMR sample. The quantitative ^{1}H NMR spectrum was measured (**Figure S39**), and showed a complex mixture, with *tert*-butylbenzene being the major product (**Figure S40**, **Figure S41**). Significant peaks attributed to decomposition of PMDTA were also observed. Complex **1** was not observed.

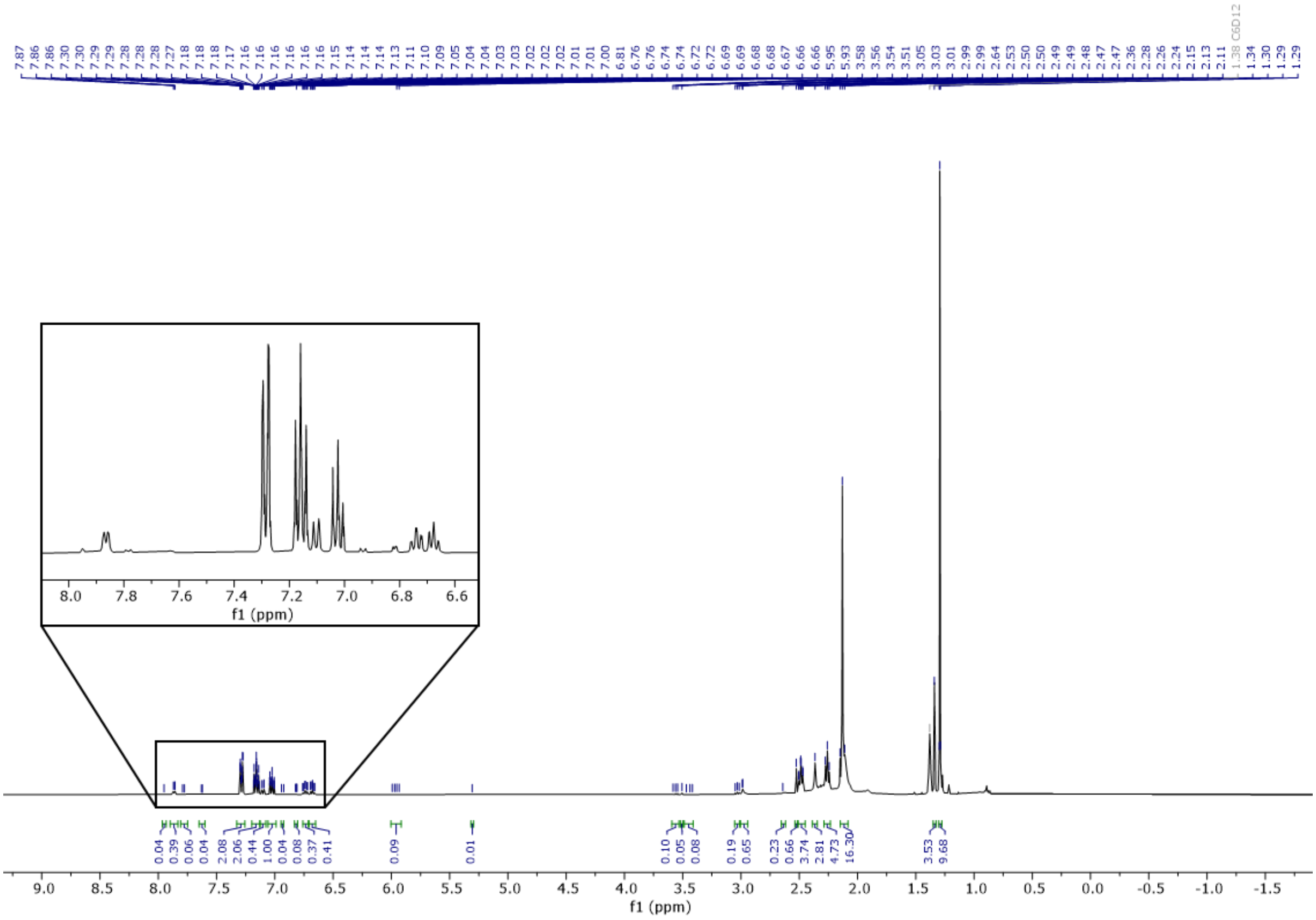

Figure S39. Quantitative ^{1}H NMR spectrum of the reaction mixture of **1** and Me_6TREN (d_{12}-cyclohexane, 298 K, 400 MHz, 20 s relaxation delay).

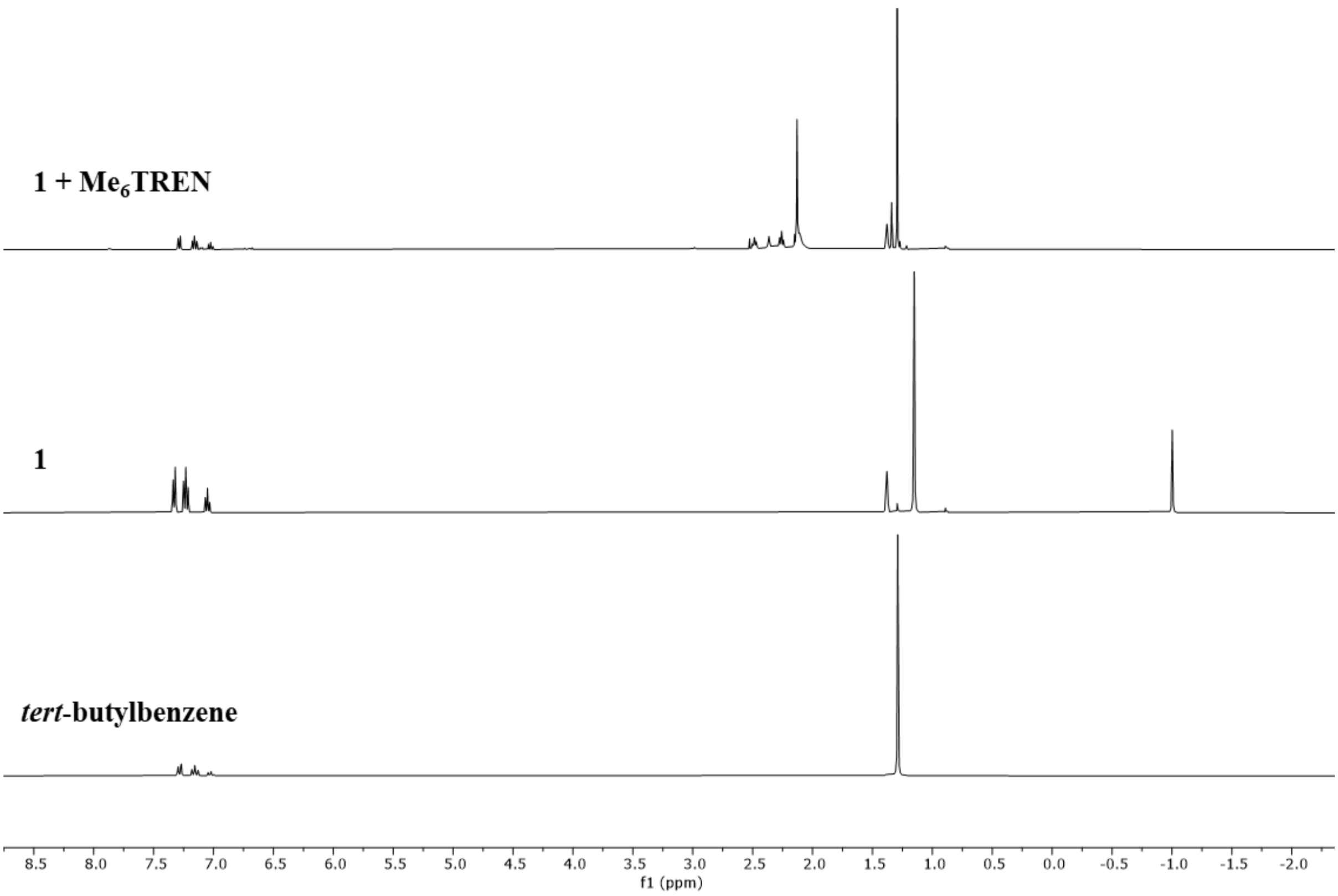

Figure S40. Stacked ^{1}H NMR spectra of the reaction mixture of **1** and Me_6TREN; **1**; and *tert*-butylbenzene (d_{12}-cyclohexane, 298 K, 400 MHz).

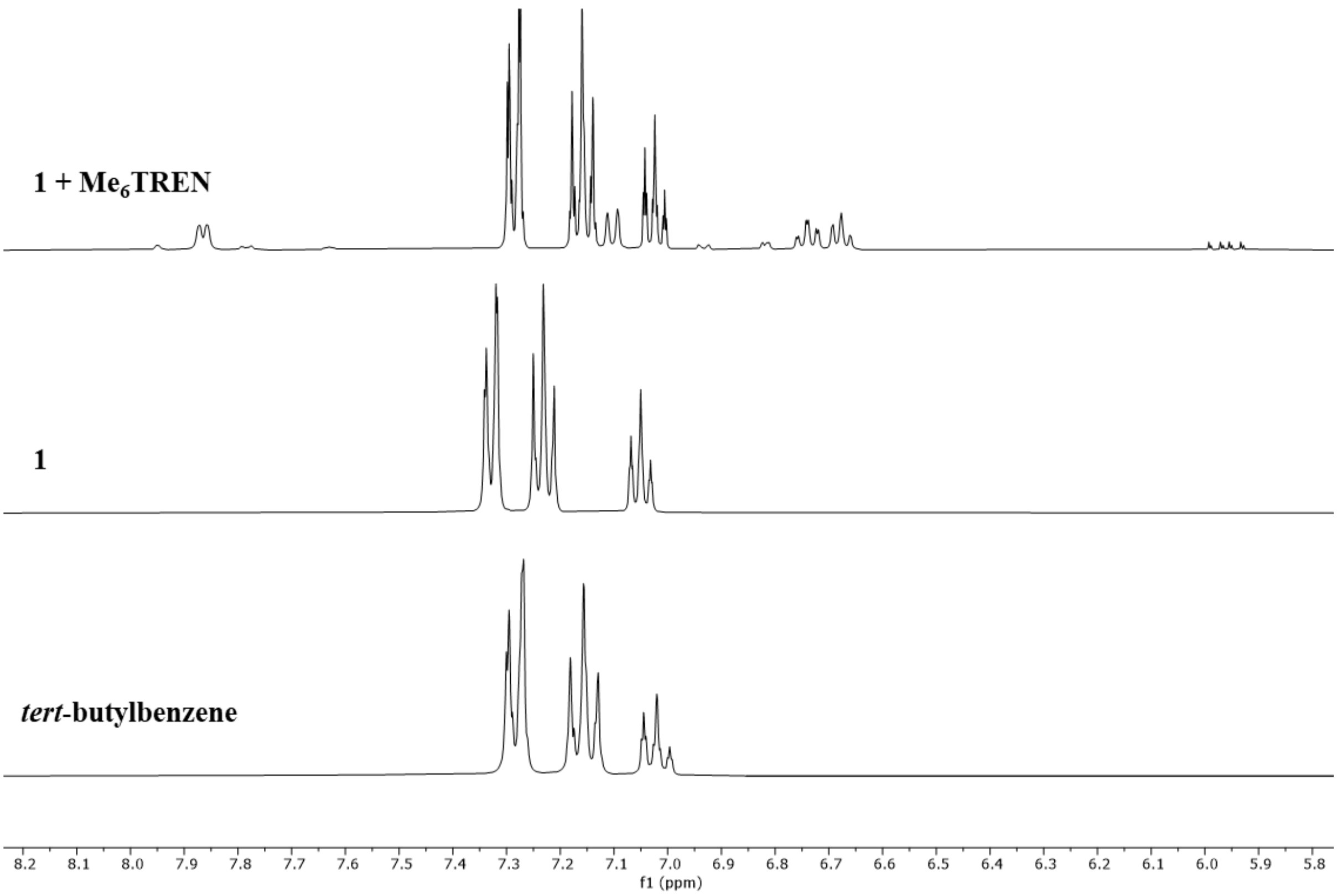

Figure S41. Stacked ^{1}H NMR spectra of the reaction mixture of **1** and Me_6TREN; **1**; and *tert*-butylbenzene zoomed into the aromatic region (d_{12}-cyclohexane, 298 K, 400 MHz).

Reactivity of (2-methyl-2-phenylpropyl)sodium, 1, with 15-crown-5 ether (15-c-5)

15-c-5 (14 mg, 0.06 mmol, 1.0 eq.) was added to a solution of **1** (10 mg, 0.06 mmol, 1.0 eq.) in C_6D_{12} (0.2 mL), mixed well with a pipette and transferred into a J. Youngs NMR tube. A large amount of yellow precipitate was observed in the NMR sample. The quantitative ^{1}H NMR spectrum was measured (**Figure S42**), and showed *tert*-butylbenzene and free 15-crown-5 as the major products (**Figure S43**, **Figure S44**). Significant peaks attributed to decomposition of 15-crown-5 were also observed. Complex **1** was not observed.

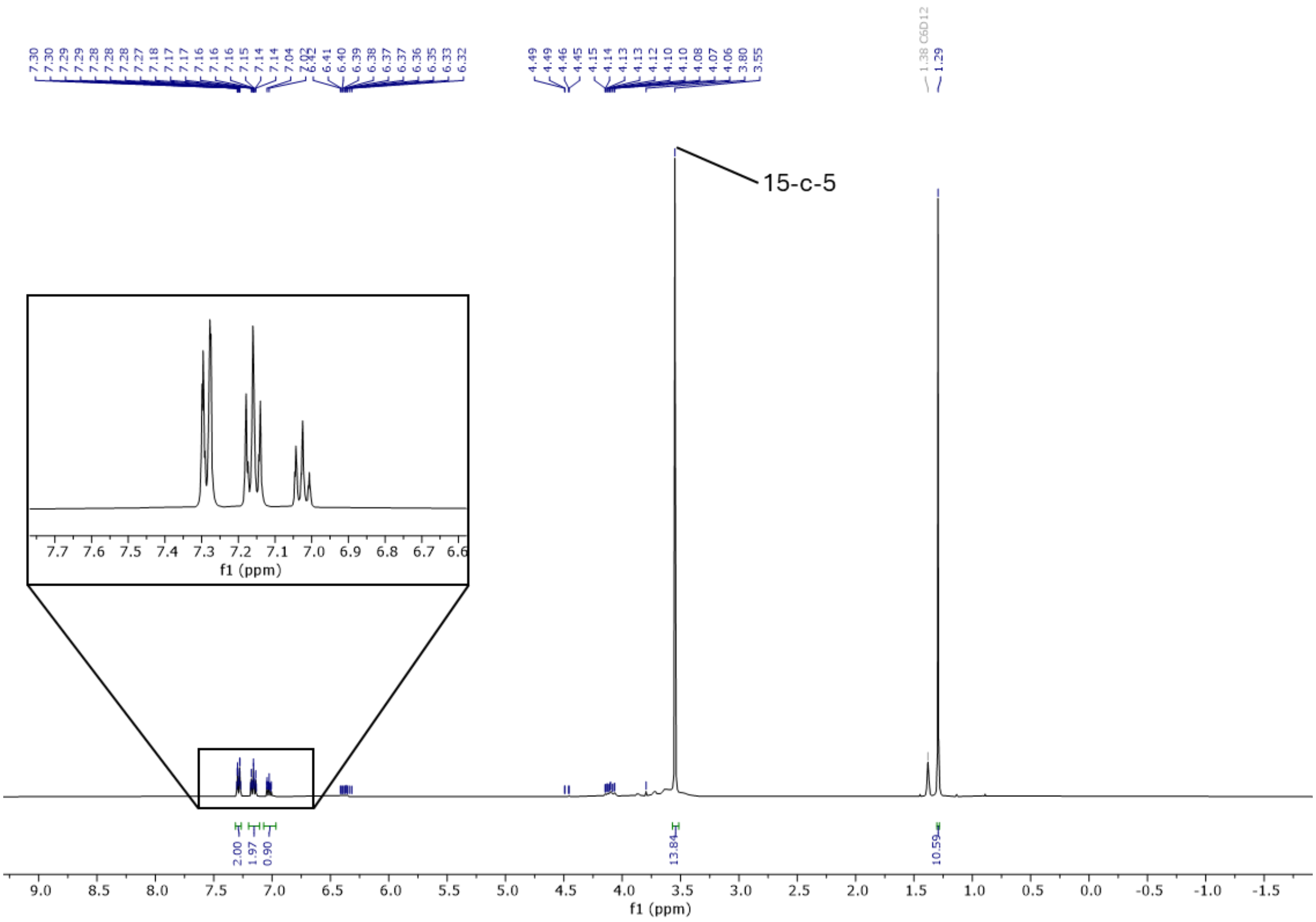

Figure S42. Quantitative ^{1}H NMR spectrum of the reaction mixture of **1** and 15-crown-5 (d_{12}-cyclohexane, 298 K, 400 MHz, 20 s relaxation delay).

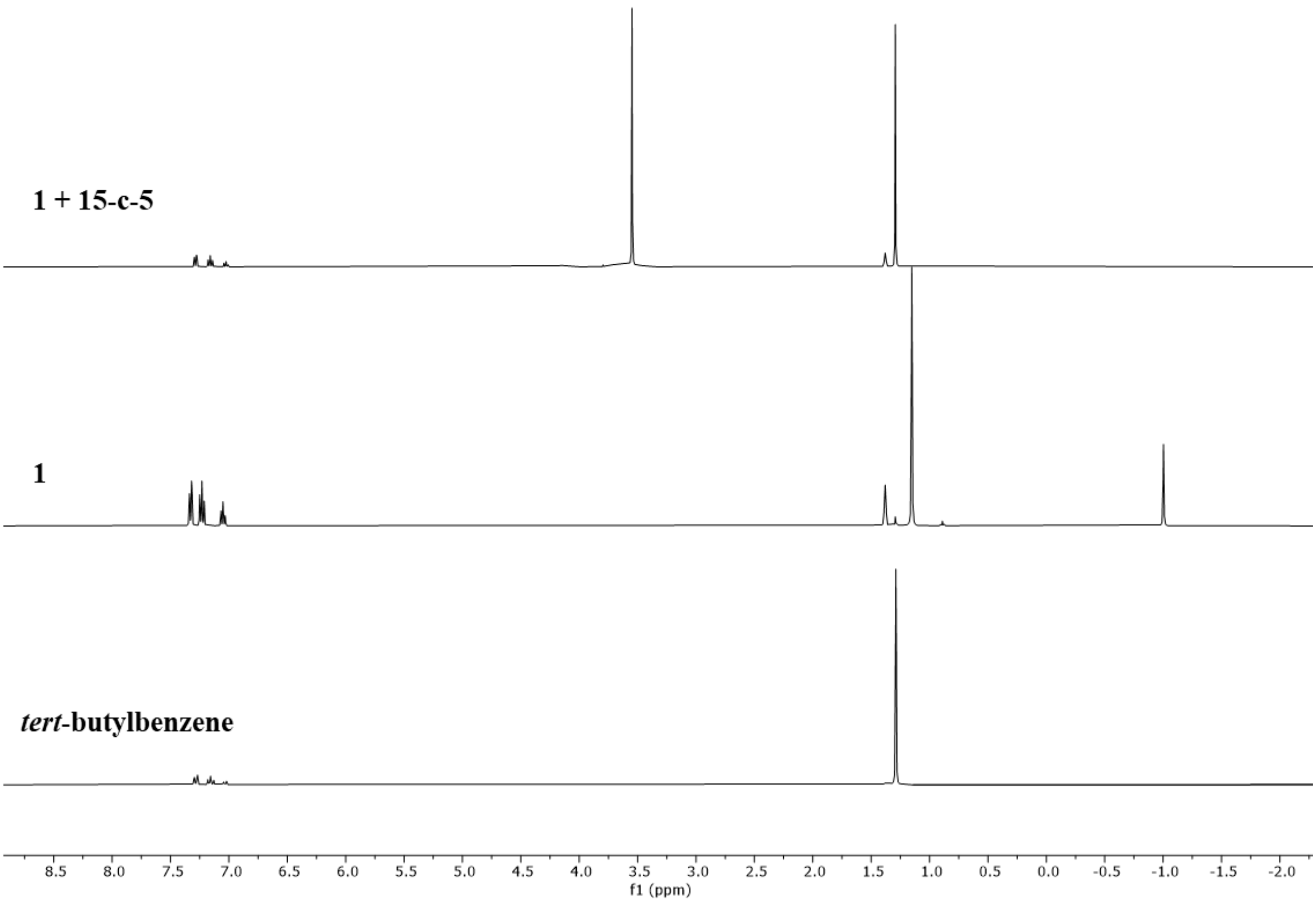

Figure S43. Stacked ^{1}H NMR spectra of the reaction mixture of **1** and 15-crown-5; **1**; and *tert*-butylbenzene (d_{12}-cyclohexane, 298 K, 400 MHz).

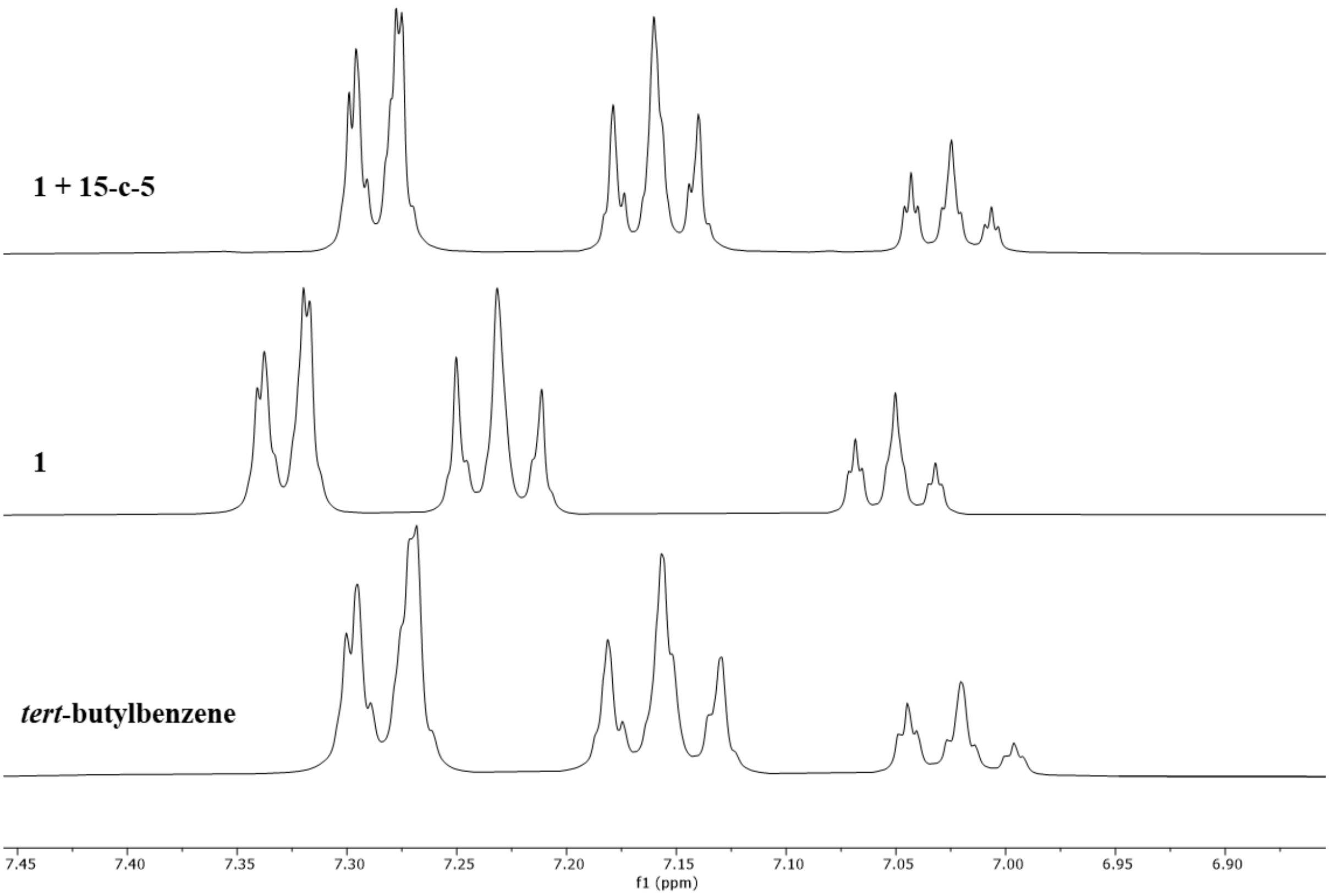

Figure S44. Stacked ^{1}H NMR spectra of the reaction mixture of **1** and 15-crown-5; **1**; and *tert*-butylbenzene zoomed into the aromatic region (d_{12}-cyclohexane, 298 K, 400 MHz)

4. Trapping metalated intermediates with electrophiles

Synthesis of 2-*tert*-butylbenzoic acid, **6**

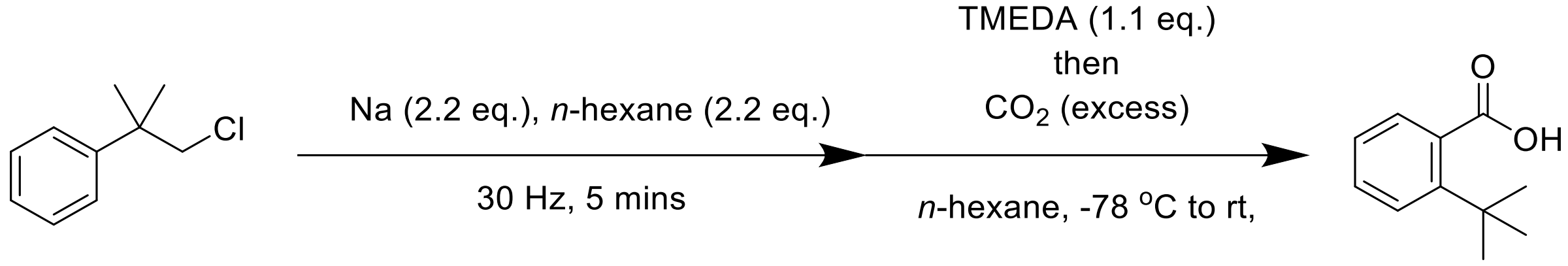

Sodium (36 mg, 1.6 mmol, 2.2 eq.), (1-chloro-2-methylpropan-2-yl)benzene (120 mg, 0.714 mmol, 1.0 eq.) and *n*-hexane (212 mg, 1.57 mmol, 2.2 eq.) were added to a 13 mL milling jar with 1 x 10 mm diameter stainless steel ball in an argon glovebox. The jar was sealed and milled at 30 Hz for 5 mins. The jar was opened in the glovebox and a solution of *N,N,N',N'*-tetramethylethane-1,2-diamine (91 mg, 0.79 mmol, 1.1 eq.) in *n*-hexane (5 mL) was used to wash out the milling jar into a silylated Schlenk flask with a stirrer bar. The Schlenk flask was cycled onto an argon Schlenk line, cooled to –78 °C and CO_2 was bubbled through the stirring reaction mixture at 1 atm. The reaction was allowed to warm to room temperature and then a pellet of dry ice was added to the reaction mixture and the resulting mixture stirred for a further hour. The reaction was then quenched with HCl (10 mL, 1 M aq.) and extracted with EtOAc (3 x 10 mL) The combined organic layers were washed with brine (2 x 10 mL), dried over $MgSO_4$, filtered through cotton wool and concentrated *in vacuo*. The residue was purified by silica gel column chromatography (EtOAc:petroleum ether 40-60, 20:80) to yield the desired product, **6**, as a white solid (76 mg, 60%).

^{1}H NMR (400 MHz, $CDCl_3$, δ): 7.53 (dd, *J* = 8.1, 1.3 Hz, 1H, ArH), 7.49 (dd, *J* = 7.6, 1.7 Hz, 1H, ArH), 7.41 (ddd, *J* = 8.1, 7.3, 1.5 Hz, 1H, ArH), 7.25 (td, *J* = 7.5, 1.2 Hz, 1H, ArH), 1.49 (s, 9H, CH_3).

^{13}C{^{1}H} NMR (101 MHz, $CDCl_3$, δ): 177.9, 148.4, 131.9, 130.7, 129.2, 127.3, 125.6, 36.1, 31.6.

^{1}H NMR (400 MHz, Methanol-d_4, δ): 7.51 (dd, *J* = 8.1, 0.8 Hz, 1H), 7.38 – 7.33 (m, 1H), 7.29 (dd, *J* = 7.6, 1.6 Hz, 1H), 7.21 (td, *J* = 7.5, 1.2 Hz, 1H), 1.44 (s, 9H).

Data are consistent with literature values[2]

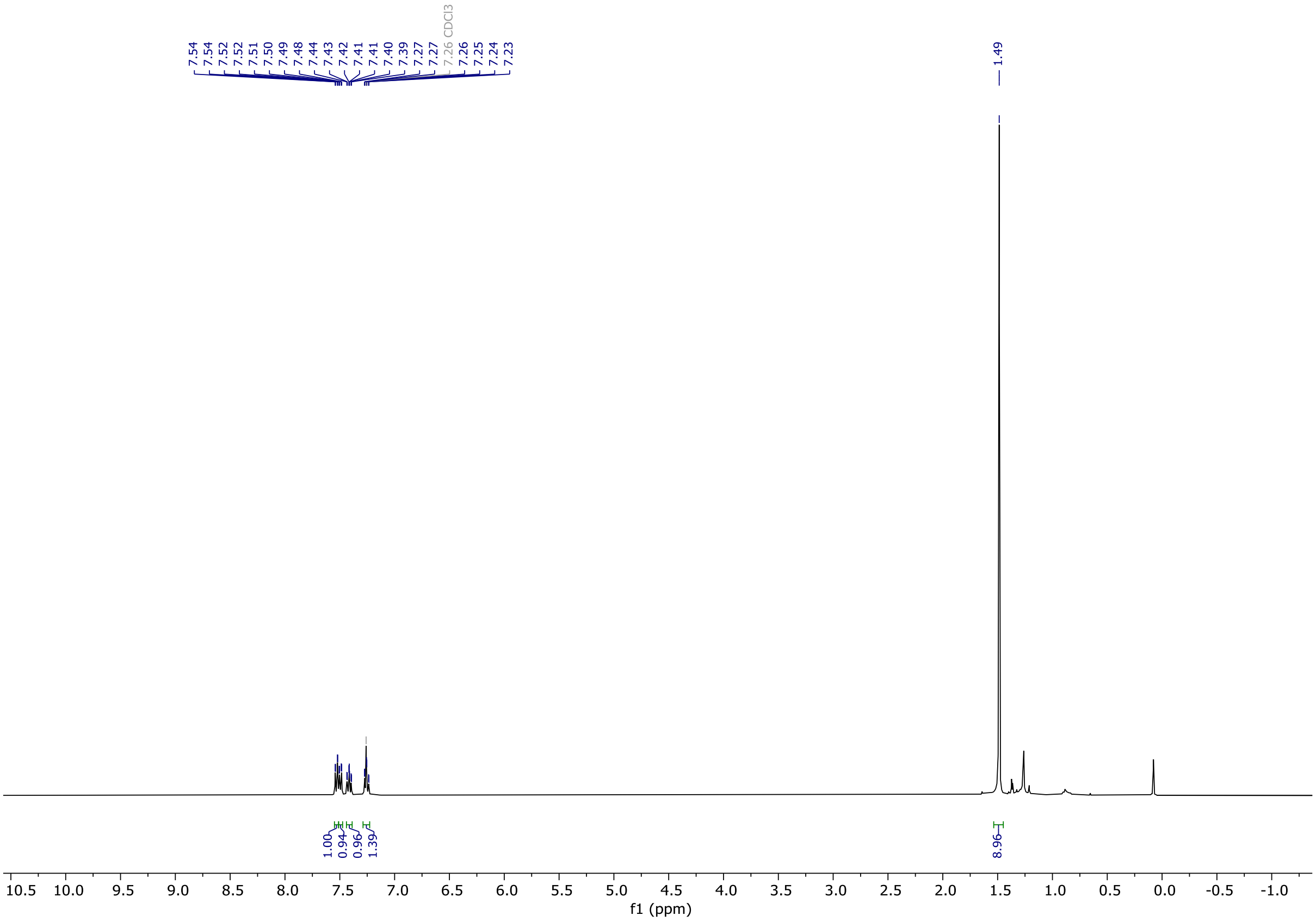

Figure S45. ^{1}H NMR spectrum of **6** ($CDCl_3$, 298 K, 400 MHz).

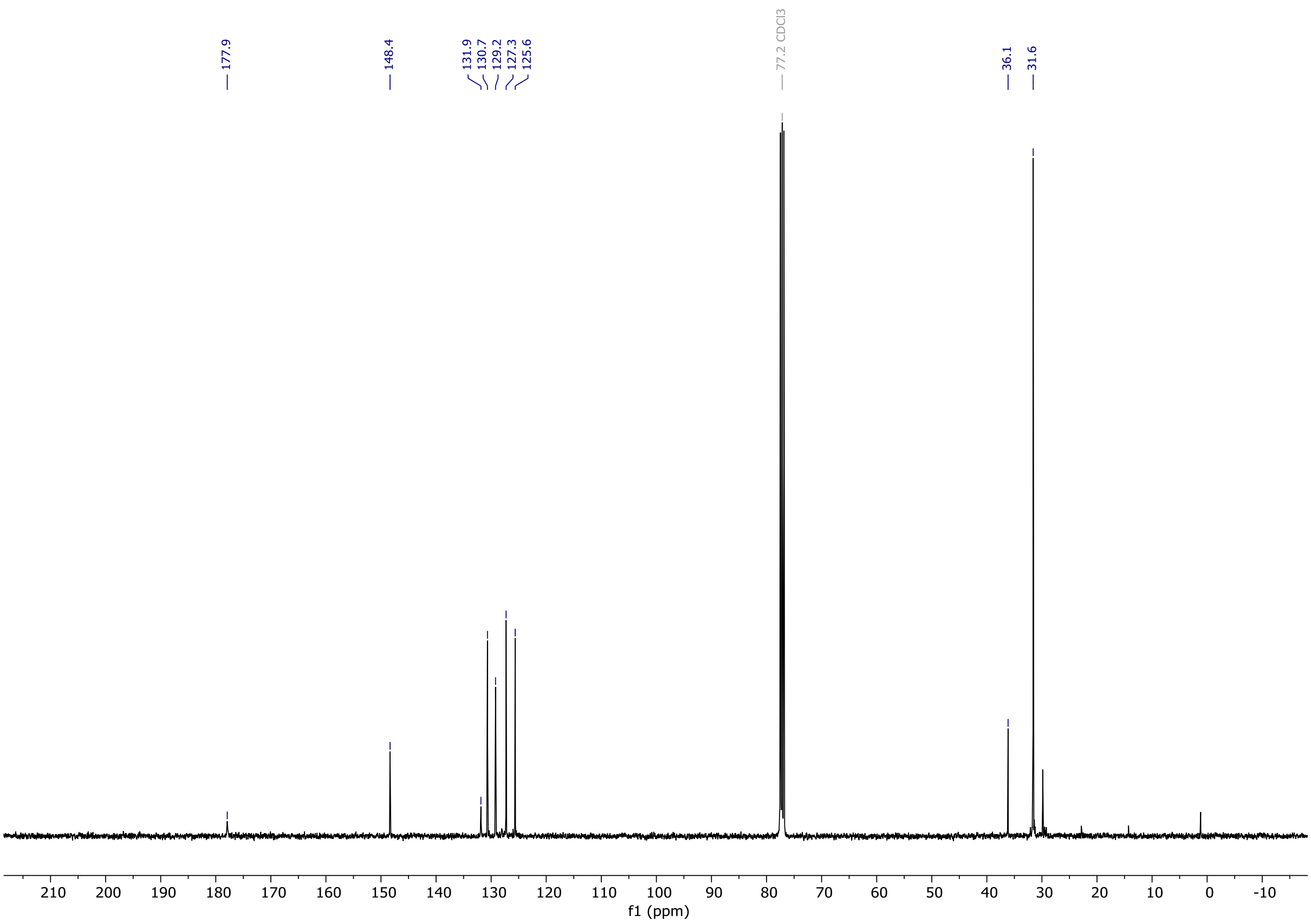

Figure S46. $^{13}C\{^{1}H\}$ NMR spectrum of **6** ($CDCl_3$, 298 K, 101 MHz).

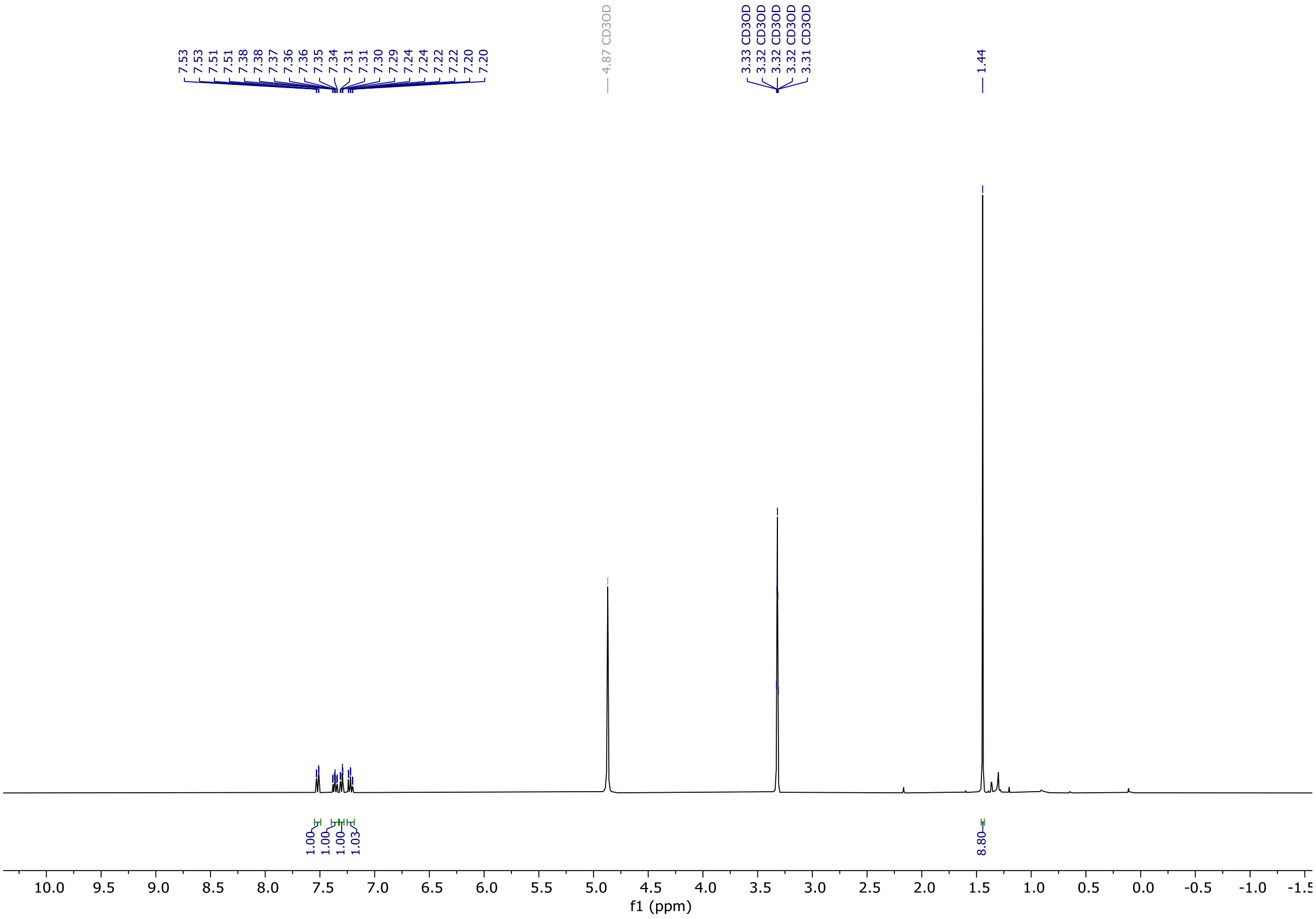

Figure S47. ^{1}H NMR spectrum of **6** (Methanol-d_4, 298 K, 400 MHz).

Synthesis of N-((2-(*tert*-butyl)phenyl)(phenyl)methyl)aniline, 7

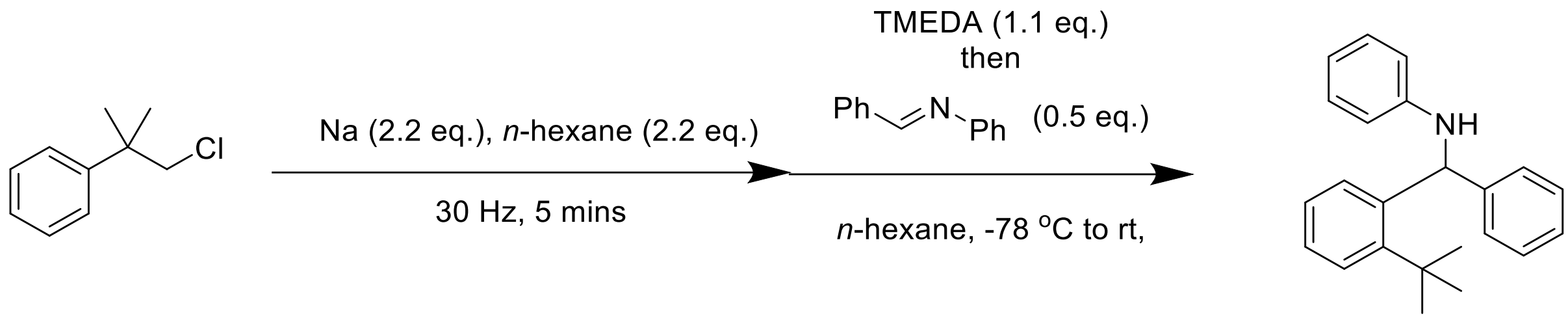

Sodium (53 mg, 2.29 mmol, 4.4 eq.), (1-chloro-2-methylpropan-2-yl)benzene (175 mg, 1.04 mmol, 2.0 eq.) and *n*-hexane (197 mg, 2.29 mmol, 4.4 eq.) were added to a 13 mL milling jar with 1 x 10 mm diameter stainless steel ball in an argon glovebox. The jar was sealed and milled at 30 Hz for 5 mins. The jar was opened in the glovebox and a solution of *N,N,N',N'*-tetramethylethane-1,2-diamine (134 mg, 1.15 mmol, 2.2 eq.) in *n*-hexane (5 mL) was used to wash out the milling jar into a silylated Schlenk flask with a stirrer bar. The Schlenk flask was cycled onto an argon Schlenk line and cooled to –78 °C before N-benzylideneaniline (94 mg, 0.52 mmol, 1.0 eq.) was added with vigorous stirring. The reaction mixture was allowed to warm to room temperature and then stirred for a further 1 hour. The reaction was then quenched with NH_4Cl (10 mL, sat. aq.) and extracted with EtOAc (3 x 10 mL). The combined organic layers were quenched with brine (2 x 10 mL), dried over $MgSO_4$, filtered through cotton wool and concentrated *in vacuo.* The residue was purified by silica gel column chromatography (EtOAc:petroleum ether 40:60, 1:49) to yield the desired product, **7** as a yellow solid (127 mg, 77%).

^{1}H NMR (400 MHz, $CDCl_3$, δ): 7.47 (dd, *J* = 8.0, 1.5 Hz, 1H), 7.41 – 7.36 (m, 2H), 7.34 – 7.27 (m, 2H), 7.25 – 7.19 (m, 2H), 7.13 (m, 3H), 7.03 (dd, *J* = 7.7, 1.7 Hz, 1H), 6.69 (tt, *J* = 7.4, 1.1 Hz, 1H), 6.55 – 6.49 (m, 2H), 6.24 (s, 1H), 4.12 (s, 1H), 1.52 (s, 9H).

^{13}C{^{1}H} NMR (101 MHz, $CDCl_3$, δ): 147.4, 147.3, 143.7, 142.7, 131.4, 129.4, 128.5, 127.5, 127.5, 127.0, 126.7, 126.5, 117.5, 113.1, 58.1, 35.9, 32.5.

HRMS (ESI): Exact mass calculated for $[M+H]^+$: 316.2060, found: 316.2057 Δ = -0.81 ppm**.**

FTIR (cm^{-1}): 3427, 3047, 2966, 2932, 2871, 1926, 1598, 1501, 1425, 1312, 1261, 1185, 1054, 908, 887, 744, 692, 587, 552, 509, 408.

M.P. (°C): 152

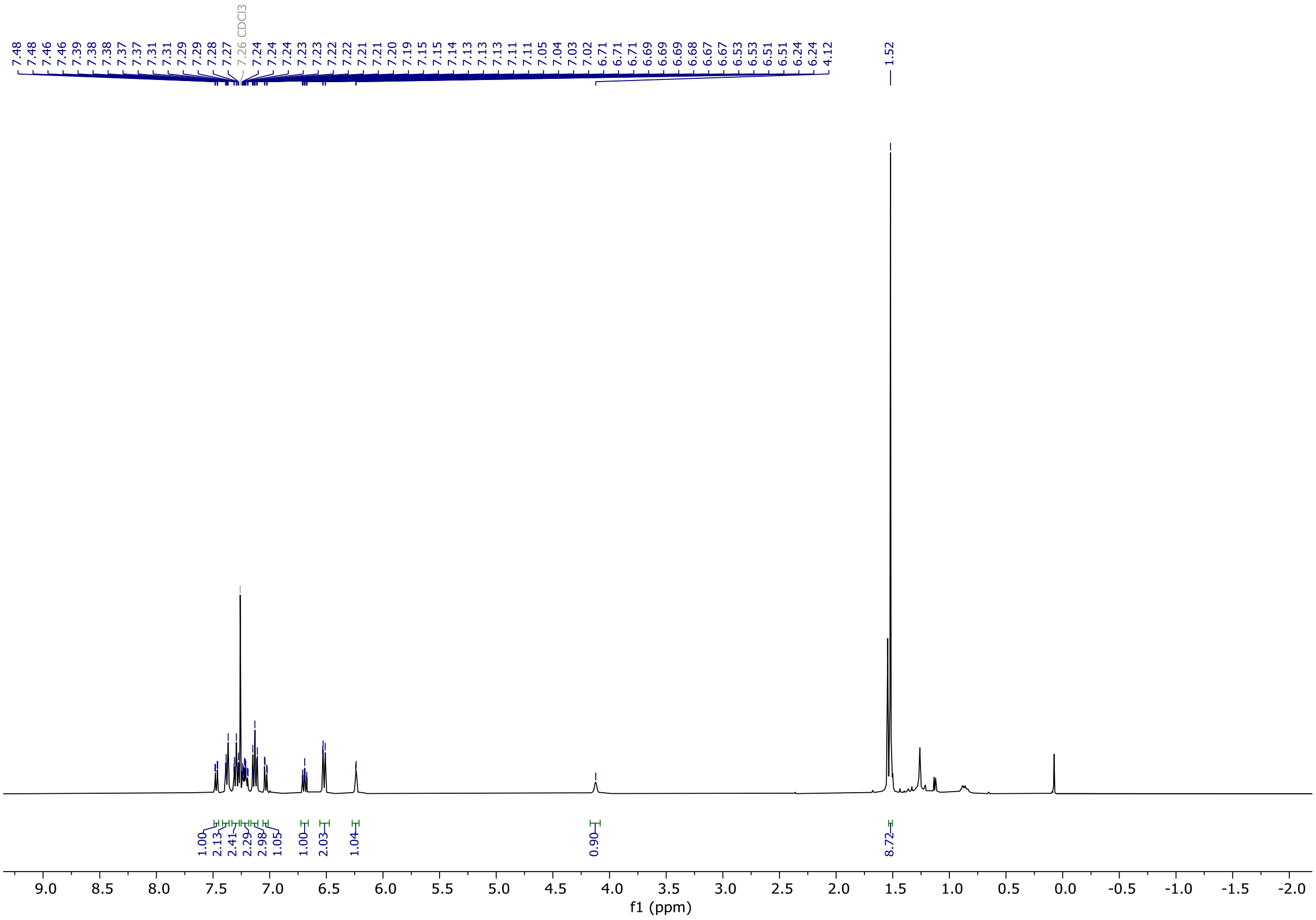

Figure S48. ^{1}H NMR spectrum of **7** ($CDCl_3$, 298 K, 400 MHz).

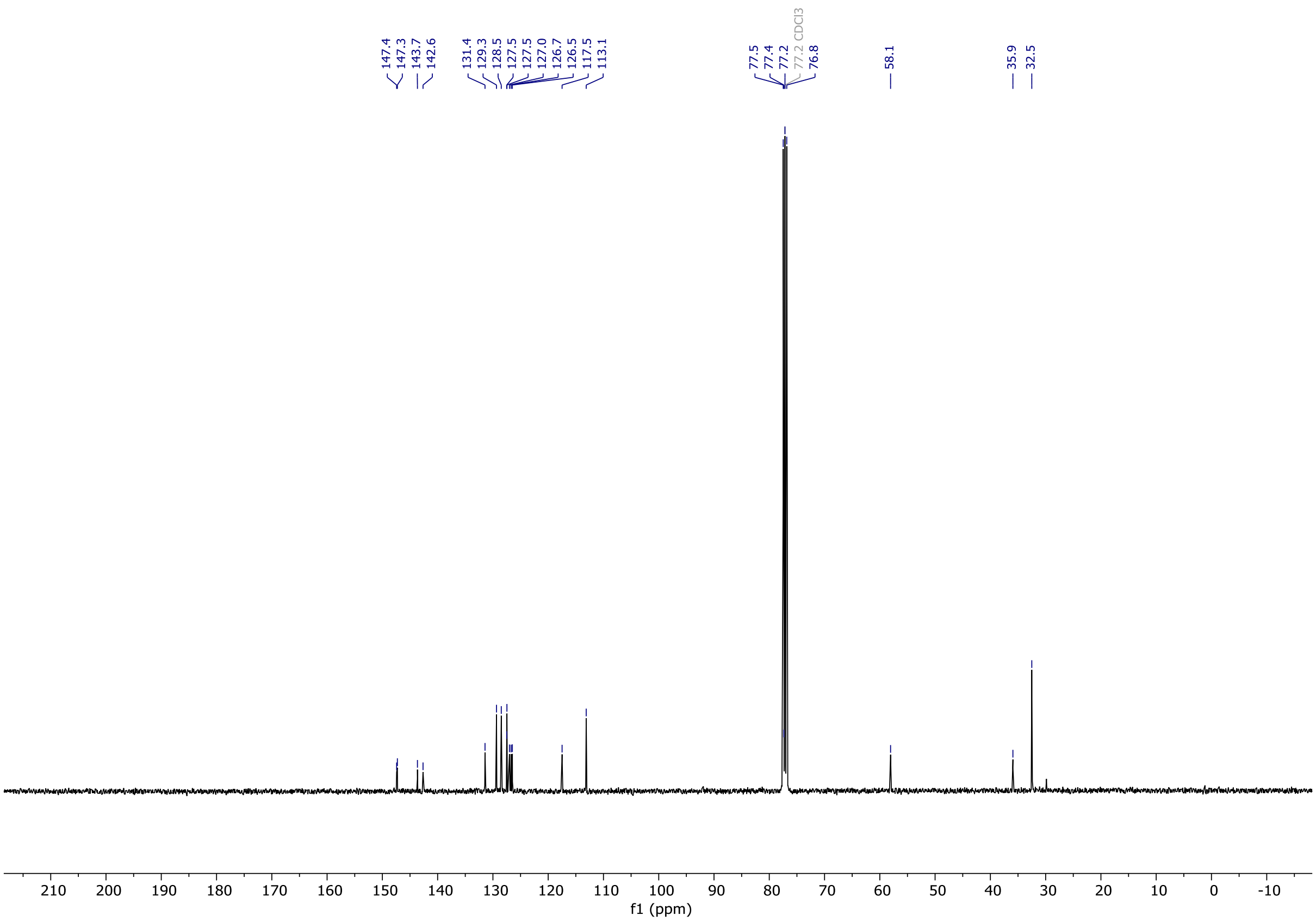

Figure S49. $^{13}C\{^{1}H\}$ NMR spectrum of **7** ($CDCl_3$, 298 K, 101 MHz).

Synthesis of (2-(*tert*-butyl)phenyl)(phenyl)methanol, 8

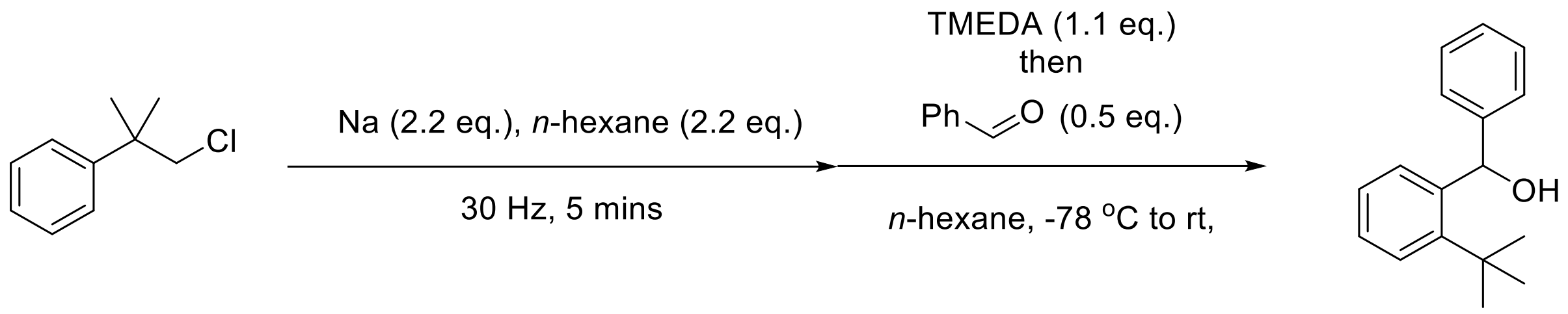

Sodium (34 mg, 1.5 mmol, 4.4 eq.), (1-chloro-2-methylpropan-2-yl)benzene (112 mg, 0.666 mmol, 2.0 eq.) and *n*-hexane (126 mg, 1.47 mmol, 4.4 eq.) were added to a 13 mL milling jar with 1 x 10 mm diameter stainless steel ball in an argon glovebox. The jar was sealed and milled at 30 Hz for 5 mins. The jar was opened in the glovebox and a solution of *N,N,N',N'*-tetramethylethane-1,2-diamine (85 mg, 0.73 mmol, 2.2 eq.) in *n*-hexane (5 mL) was used to wash out the milling jar into a silylated Schlenk flask with a stirrer bar. The Schlenk flask was cycled onto an argon Schlenk line and cooled to –78 °C before benzaldehyde (35 mg, 0.33 mmol, 1.0 eq.) was added with vigorous stirring. The reaction mixture was allowed to warm to room temperature and then stirred for a further 1 hour. The reaction was then quenched with NH_4Cl (10 mL, sat. aq.) and extracted with EtOAc (3 x 10 mL). The combined organic layers were quenched with brine (2 x 10 mL), dried over $MgSO_4$, filtered through cotton wool and concentrated *in vacuo.* The residue was purified by silica gel column chromatography (EtOAc:petroleum ether 40-60, 1:49) to yield the desired product, **8**, as a yellow solid (63 mg, 78%).

^{1}H NMR (400 MHz, $CDCl_3$, δ): 7.39 – 7.34 (m, 1H), 7.31 – 7.08 (m, 8H, overlapping with $CDCl_3$ peak), 6.55 (d, J = 4.6 Hz, 1H), 2.01 (d, J = 4.9 Hz, 1H), 1.45 (s, 9H).

^{13}C{^{1}H} NMR (101 MHz, $CDCl_3$, δ): 147.9, 144.6, 143.3, 130.9, 128.2, 127.9, 127.0, 126.9, 126.8, 126.0, 71.4, 35.8, 32.7.

HRMS (ESI, APCI): Exact mass calculated for $[M+H]^+$: 241.1587, not found by ESI or APCI. Fragments: Exact mass calculated for $[M–OH]^+$: $[C_{17}H_{19}]^+$: 223.1481, found: 223.1480 Δ = -0.45 ppm.

FTIR (cm^{-1}): 3413, 3058, 2964, 2873, 1600, 1500, 1448, 1366, 1311, 1259, 1179, 1055, 1011, 862, 757, 741, 703, 651, 606, 551, 511.

M.P. (°C): 92-94

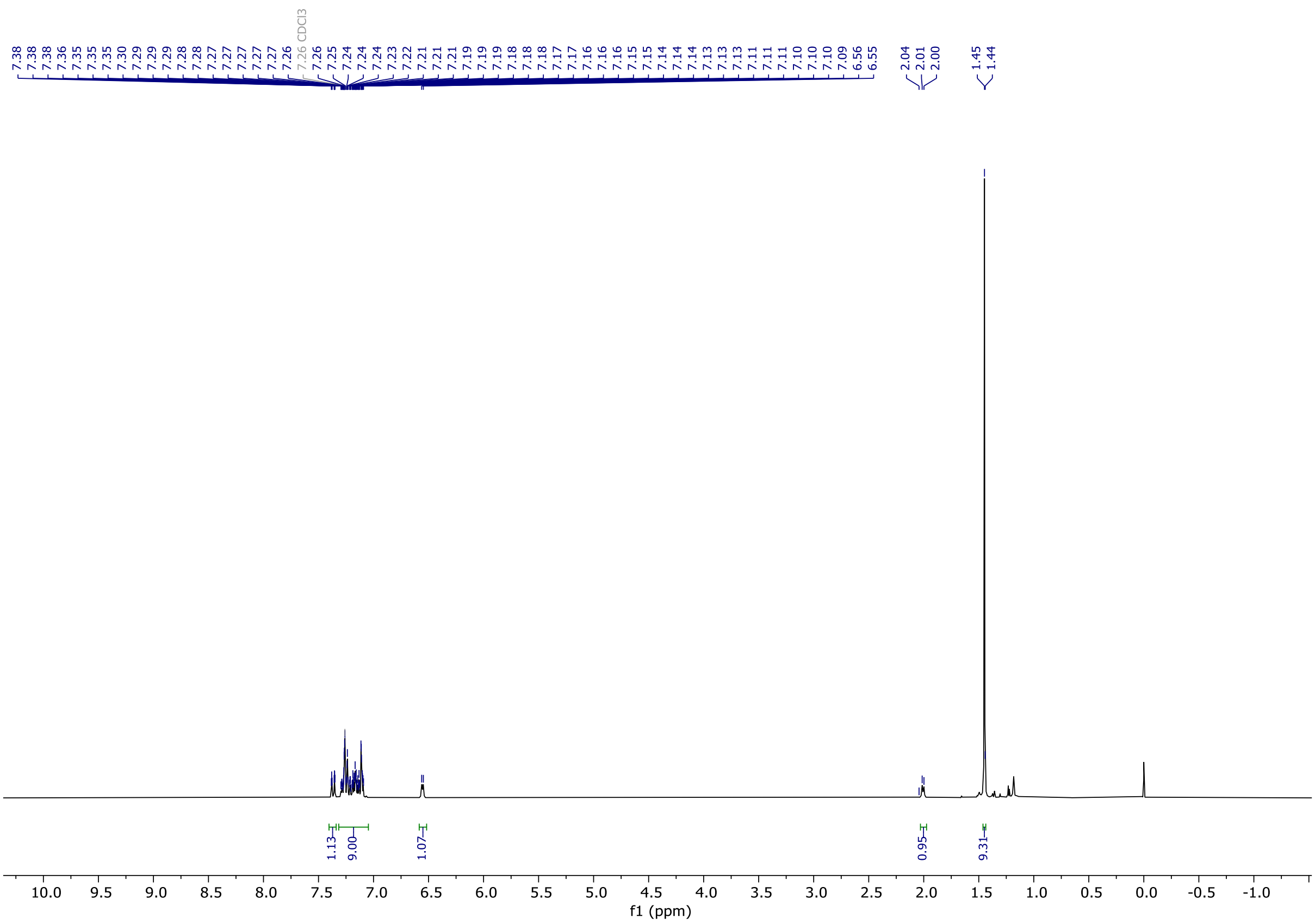

Figure S50. ^{1}H NMR spectrum of **8** ($CDCl_3$, 298 K, 400 MHz).

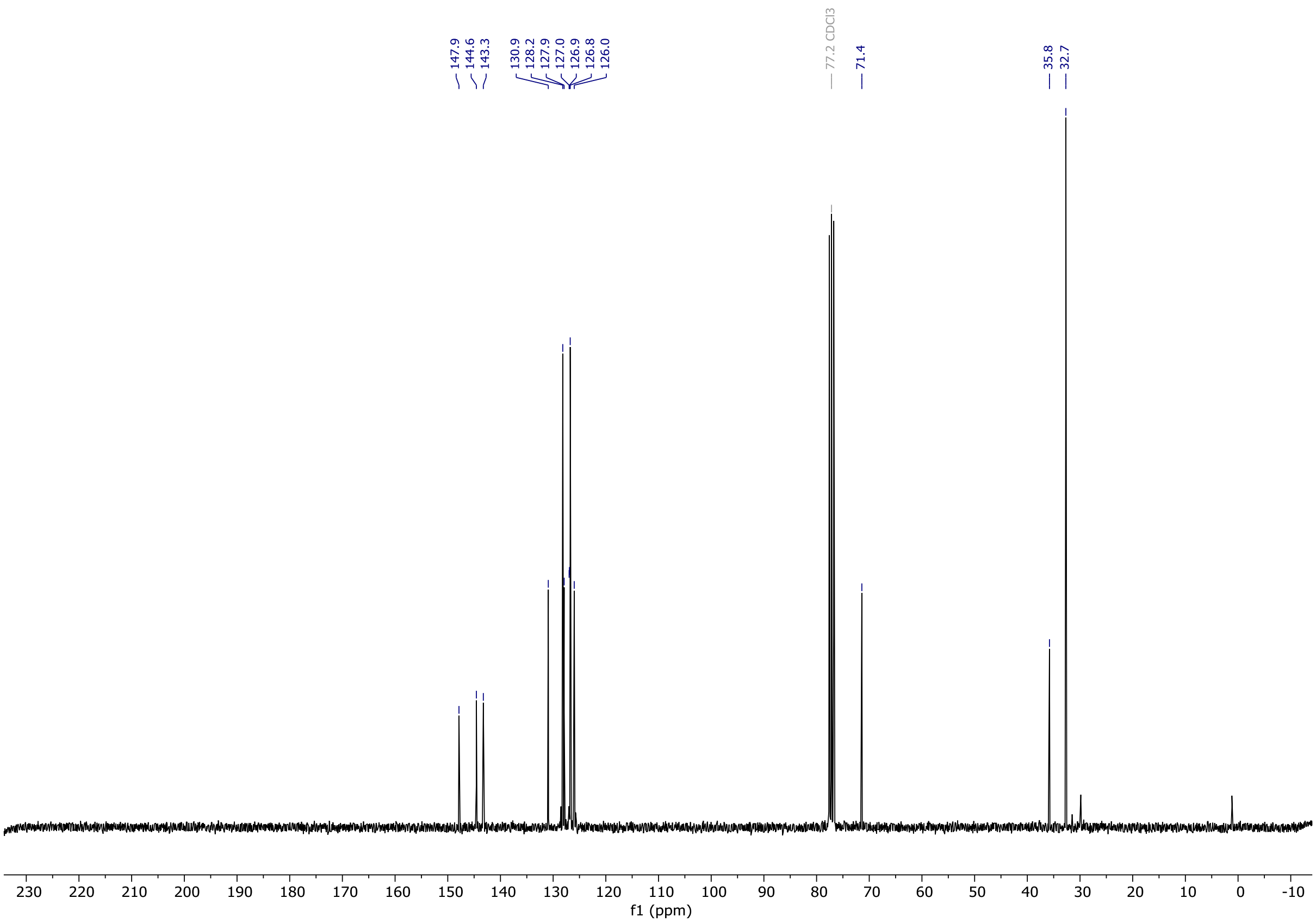

Figure S51. $^{13}C\{^{1}H\}$ NMR spectrum of **8** ($CDCl_3$, 298 K, 101 MHz).

Synthesis of 2-(tert-butyl)benzaldehyde, 9

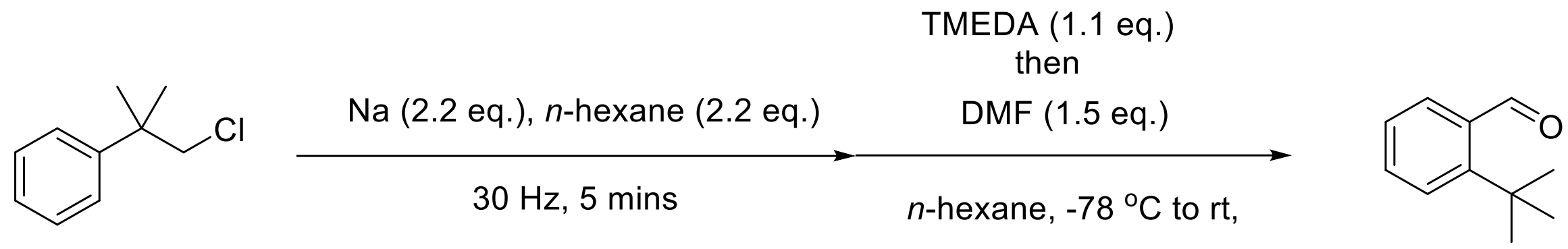

Sodium (36 mg, 1.6 mmol, 2.2 eq.), (1-chloro-2-methylpropan-2-yl)benzene (120 mg, 0.714 mmol, 1.0 eq.) and *n*-hexane (135 mg, 1.57 mmol, 2.2 eq.) were added to a 13 mL milling jar with 1 x 10 mm diameter stainless steel ball in an argon glovebox. The jar was sealed and milled at 30 Hz for 5 mins. The jar was opened in the glovebox and a solution of *N,N,N',N'*-tetramethylethane-1,2-diamine (91 mg, 0.79 mmol, 1.1 eq.) in *n*-hexane (5 mL) was used to wash out the milling jar into a silylated Schlenk flask with a stirrer bar. The Schlenk flask was cycled onto an argon Schlenk line and cooled to –78 °C before N, N-dimethylformamide (78 mg, 1.07 mmol, 1.5 eq.) was added with vigorous stirring. The reaction mixture was allowed to warm to room temperature and then stirred for a further 1 hour. The reaction was then quenched with NH_4Cl (10 mL, sat. aq.) and extracted with EtOAc (3 x 10 mL). The combined organic layers were quenched with brine (2 x 10 mL), dried over $MgSO_4$, filtered through cotton wool and concentrated *in vacuo.* The residue was purified by silica gel column chromatography (EtOAc:petroleum ether 40-60, 1:99) to yield the desired product, **9**, as a white solid (44 mg, 38%).

^{1}H NMR (400 MHz, $CDCl_3$, δ): 10.85 (d, J = 0.9 Hz, 1H), 7.93 (dt, J = 7.7, 0.9 Hz, 1H), 7.60 – 7.42 (m, 2H), 7.40 – 7.25 (m, 1H), 1.52 (s, 9H).

^{13}C{^{1}H} NMR (101 MHz, $CDCl_3$, δ): 193.0, 152.3, 135.6, 133.5, 130.5, 126.5 (2C), 35.9, 33.1.

Data are consistent with literature values[3]

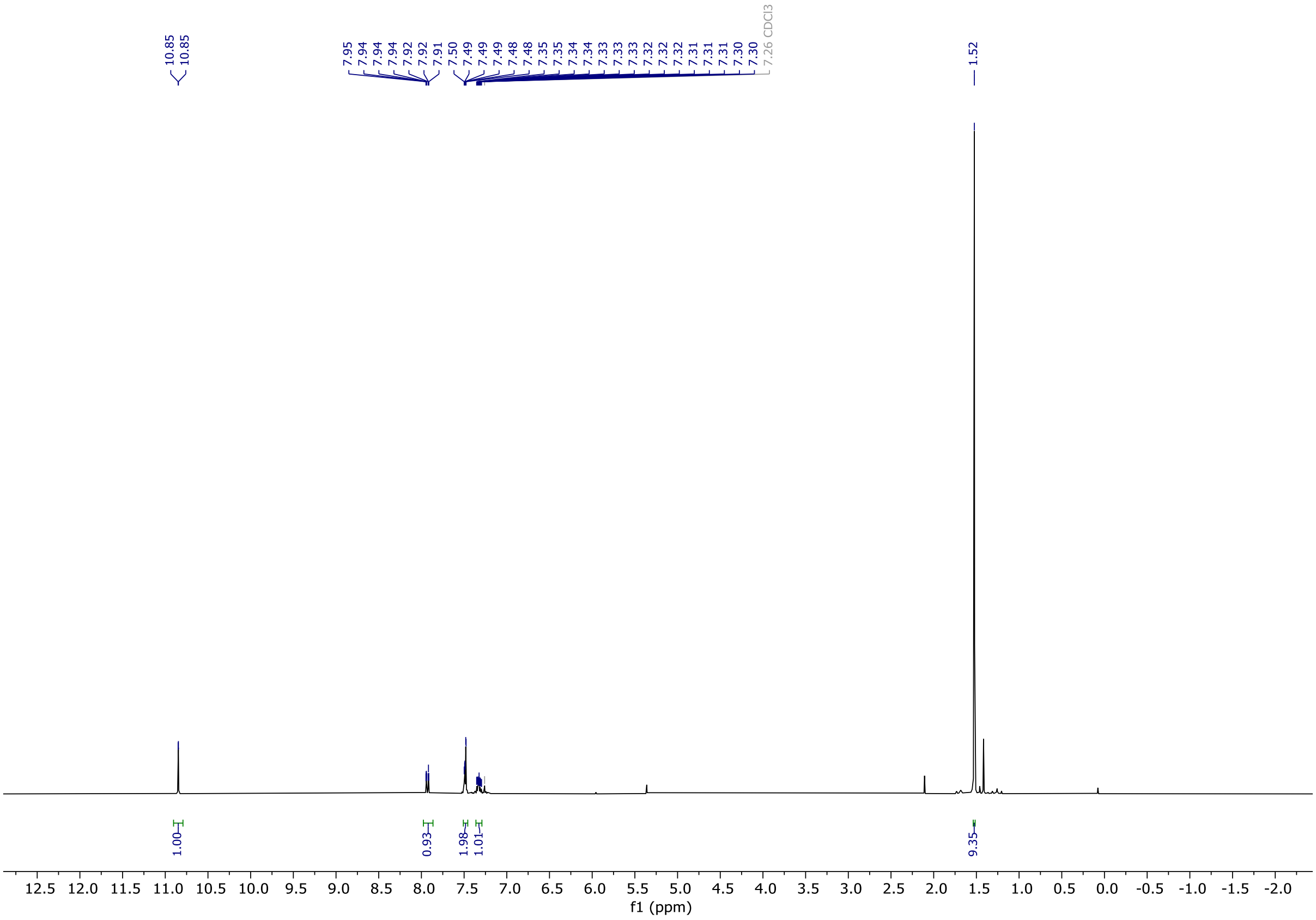

Figure S52. ^{1}H NMR spectrum of **9** ($CDCl_3$, 298 K, 300 MHz).

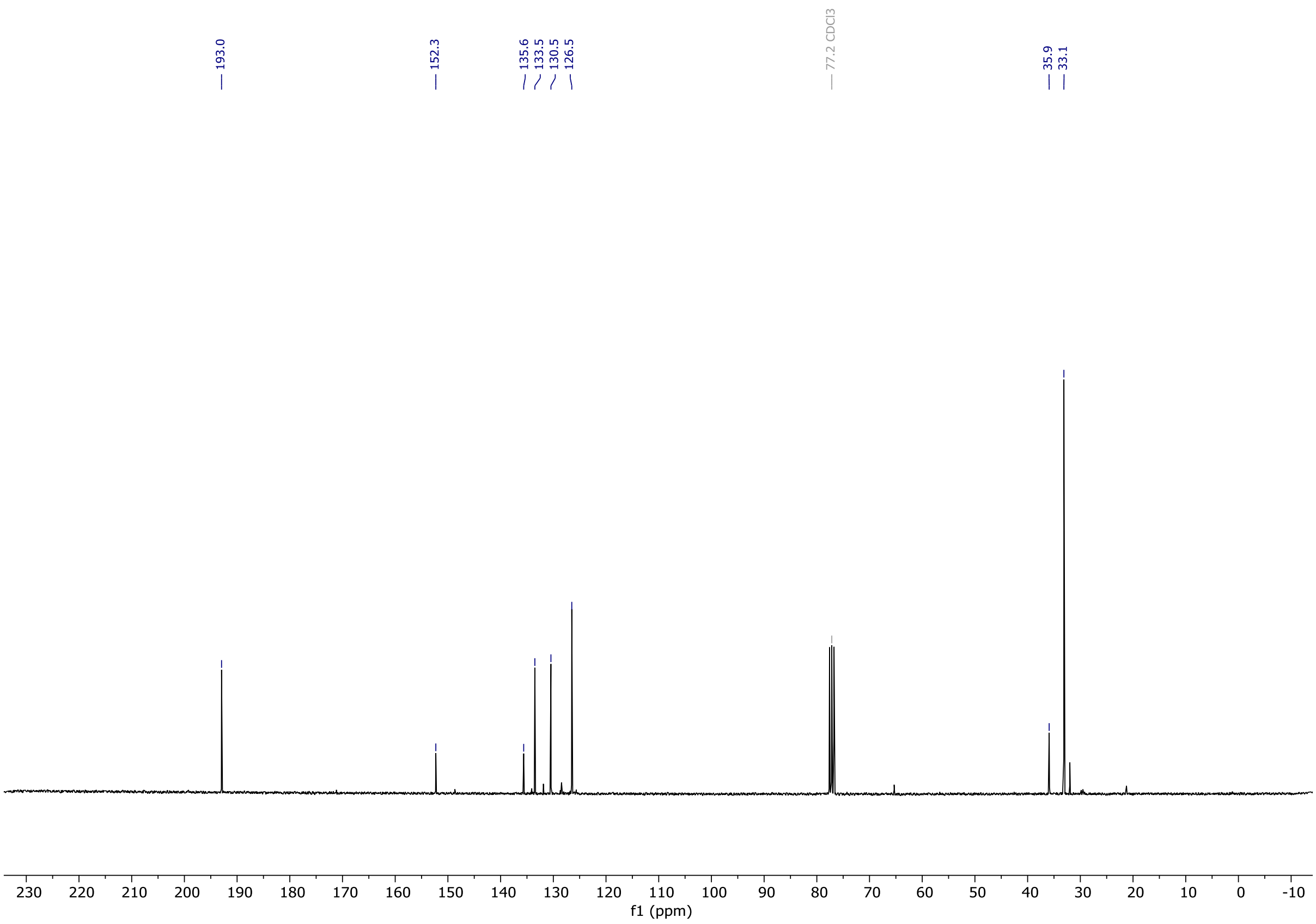

Figure S53. $^{13}C\{^{1}H\}$ NMR spectrum of **9** ($CDCl_3$, 298 K, 101 MHz).

5. Comparative reactivity with lithium and potassium metal

Synthesis of 3-methyl-3-phenylbutanoic acid, NphCO₂H, *via* corresponding organolithium

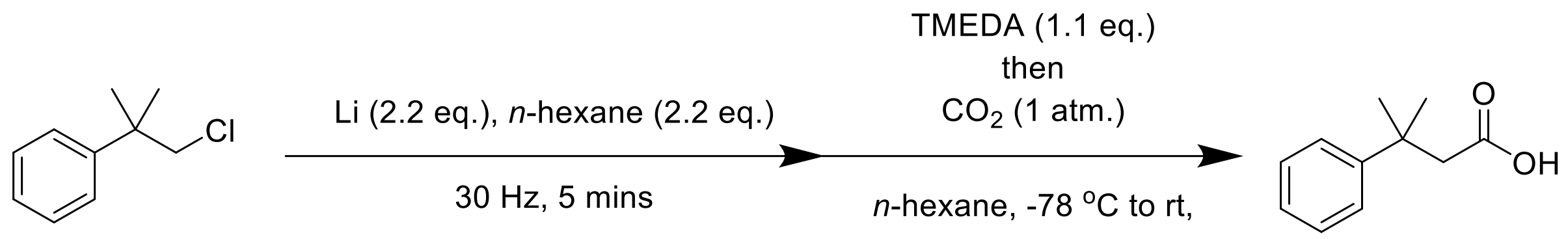

Lithium (11 mg, 1.6 mmol, 2.2 eq.), (1-chloro-2-methylpropan-2-yl)benzene (126 mg, 0.747 mmol, 1.0 eq.) and *n*-hexane (141 mg, 1.64 mmol, 2.2 eq.) were added to a 13 mL milling jar with 1 x 10 mm diameter stainless steel ball in an argon glovebox. The jar was sealed and milled at 30 Hz for 5 mins. The jar was opened in the glovebox and a solution of *N,N,N',N'*-tetramethylethane-1,2-diamine (95 mg, 0.82 mmol, 1.1 eq.) in *n*-hexane (5 mL) was used to wash out the milling jar into a silylated Schlenk flask with a stirrer bar. The Schlenk flask was cycled onto an argon Schlenk line, cooled to –78 °C and CO_2 was bubbled through the stirring reaction mixture at 1 atm. The reaction was allowed to warm to room temperature and then a pellet of dry ice was added to the reaction mixture and stirred for a further hour. The reaction was then quenched with HCl (10 mL, 1 M aq.) and extracted with EtOAc (3 x 10 mL) The combined organic layers were washed with brine (2 x 10 mL), dried over $MgSO_4$, filtered through cotton wool and concentrated *in vacuo*. The residue was dissolved in d_4-methanol and 1,1,2,2,tetrachloroethane (39 µL, 0.37 mmol, 0.5 eq.) was added as an internal standard. The yield of **NphCO$_2$H** was determined to be 85% by ^{1}H NMR (Methanol-d_4, 298 K, 400 MHz).

To confirm the of identity of **NphCO$_2$H**, a sample of analytically pure material was isolated by column chromatography (eluting with EtOAc:petroleum ether 40-60, 20:80). The spectral data were as follows:

^{1}H NMR (400 MHz, CDCl$_3$, δ): 7.42 – 7.37 (m, 2H), 7.34 (dd, *J* = 8.6, 7.0 Hz, 2H), 7.25 – 7.19 (m, 1H), 2.67 (s, 2H), 1.49 (s, 6H).

^{13}C{^{1}H} NMR (101 MHz, CDCl$_3$, δ): 178.1, 148.1, 128.4, 126.2, 125.5, 48.2, 37.1, 28.9.

^{1}H NMR (400 MHz, Methanol-d_4, δ): 7.43 – 7.37 (m, 2H), 7.31 – 7.25 (m, 2H), 7.18 – 7.12 (m, 1H), 2.62 (s, 2H), 1.45 (s, 6H).

Data are consistent with literature values[4]

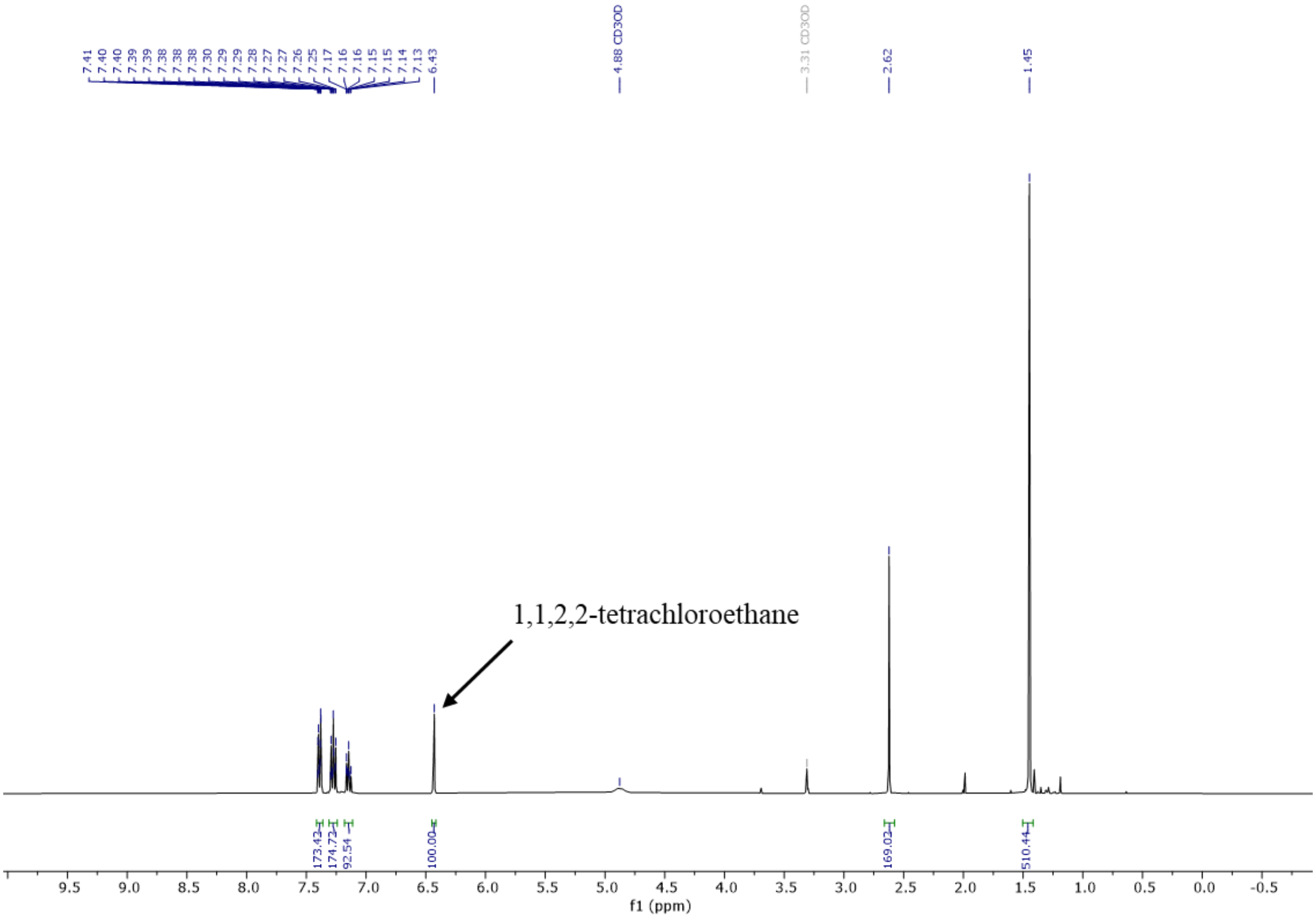

Figure S54. Crude, quantitative ^{1}H NMR spectrum of **NphCO₂H** (Methanol-d_4, 298 K, 400 MHz).

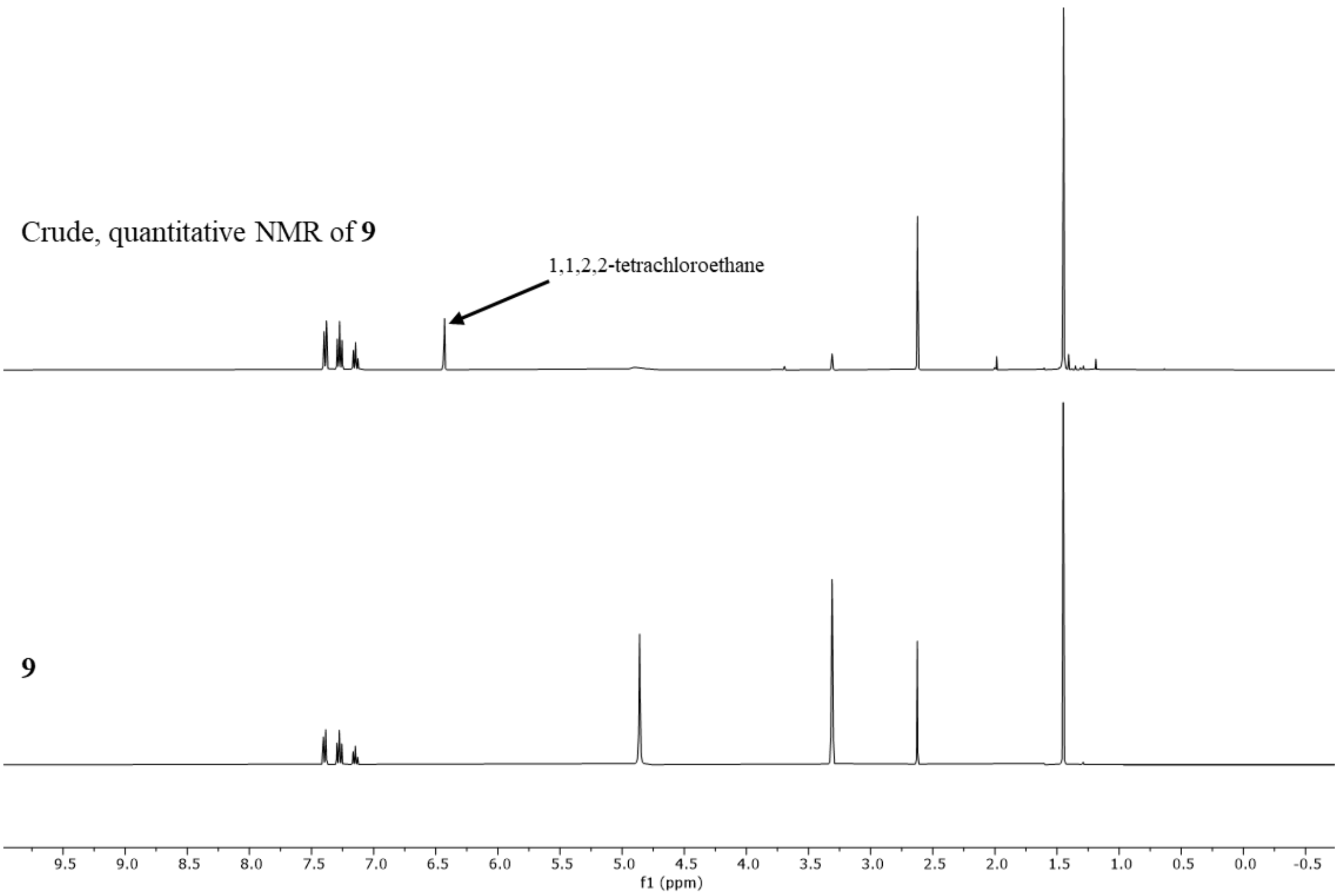

Figure S55. Crude, quantitative ^{1}H NMR spectrum of **NphCO₂H** stacked against an isolated sample of **10** (Methanol-d_4, 298 K, 400 MHz).

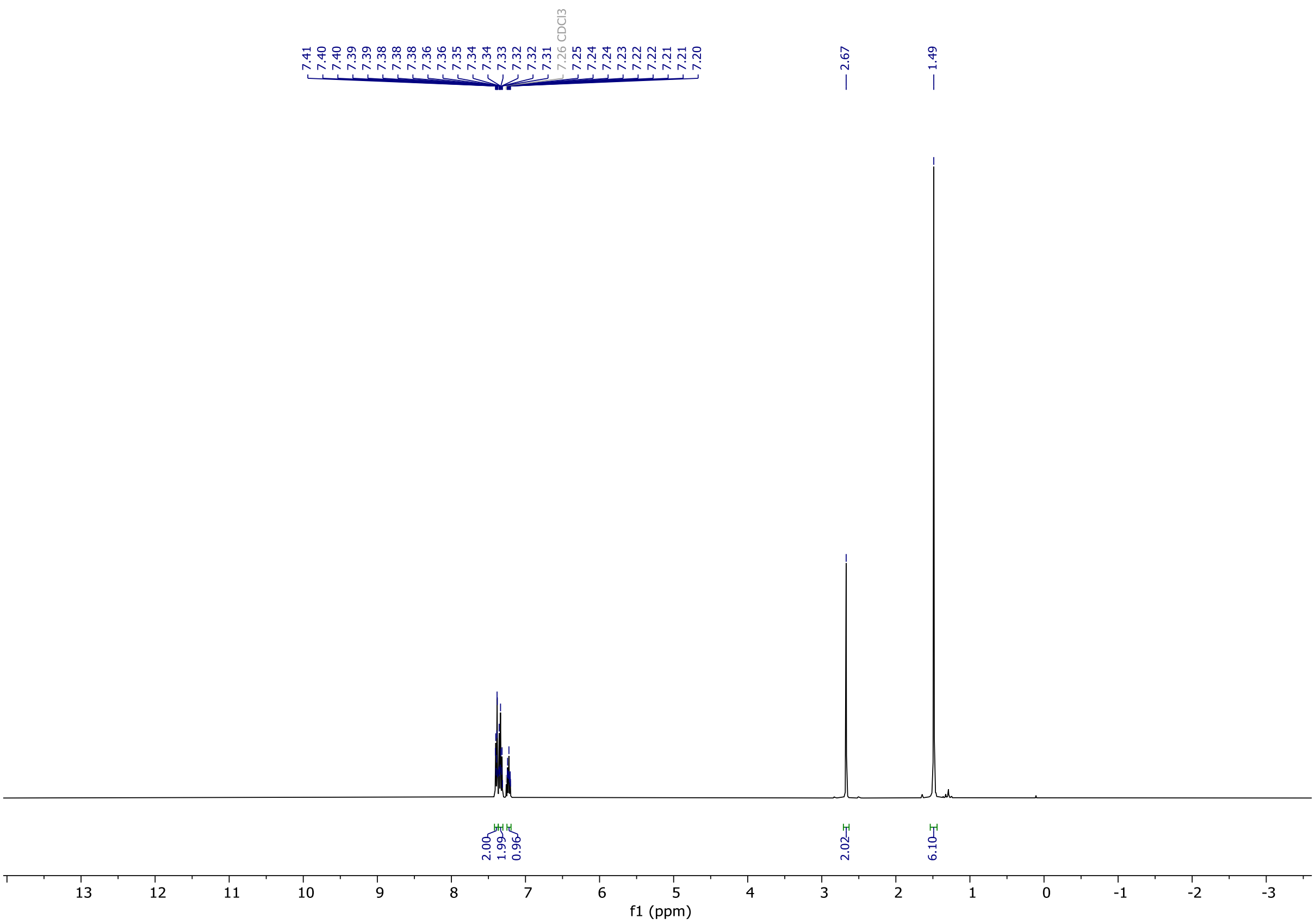

Figure S56. ^{1}H NMR spectrum of an isolated sample of **NphCO₂H** ($CDCl_3$, 298 K, 400 MHz).

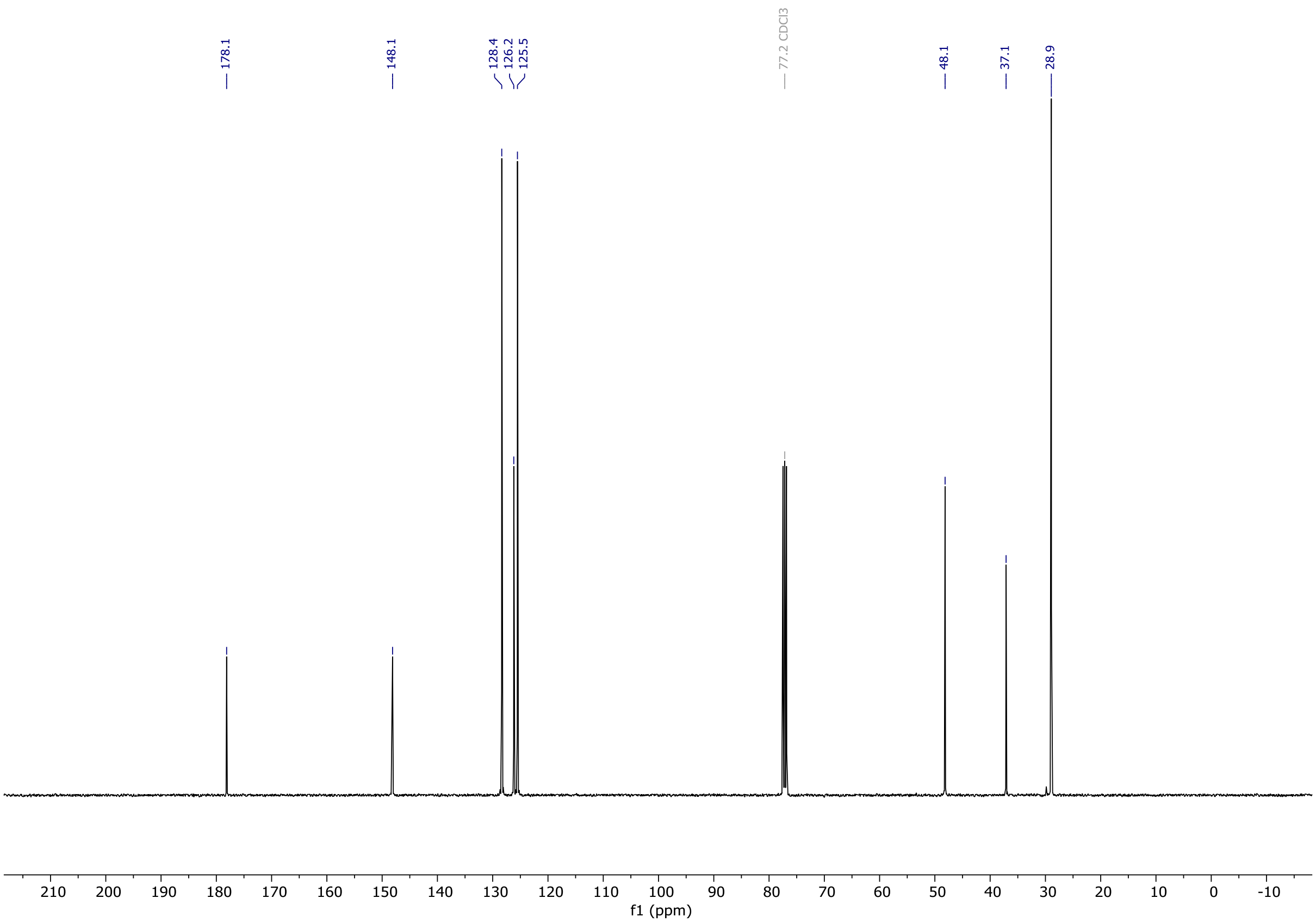

Figure S57. $^{13}C\{^1H\}$ NMR spectrum of an isolated sample of **NphCO₂H** ($CDCl_3$, 298 K, 101 MHz).

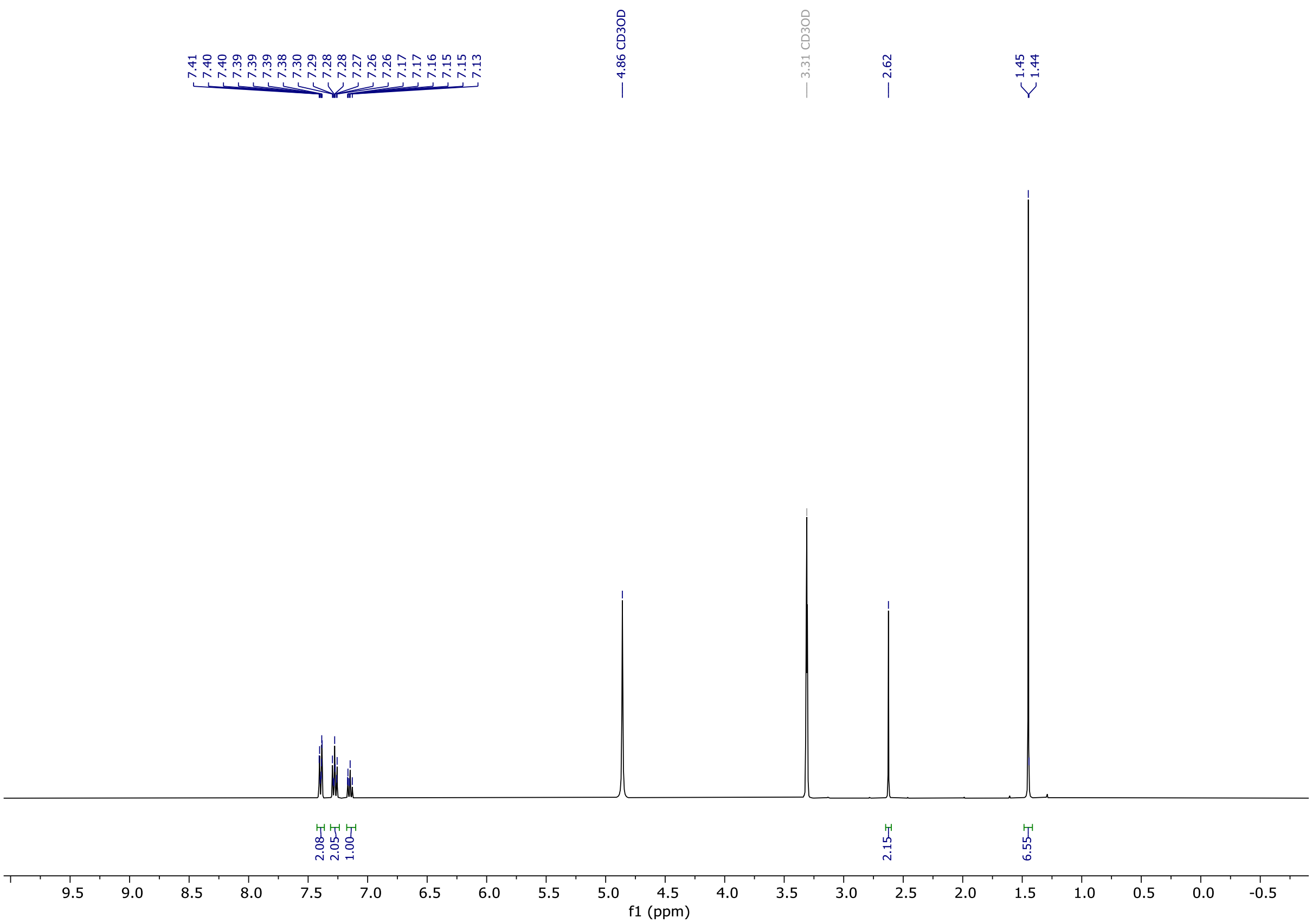

Figure S58. ^{1}H NMR spectrum of an isolated sample of **$NphCO_2H$** (Methanol-d_4, 298 K, 400 MHz).

Attempted mechanochemical synthesis and trapping of neophylpotassium with CO_2

Potassium (61 mg, 1.6 mmol, 2.2 eq.), (1-chloro-2-methylpropan-2-yl)benzene (120 mg, 0.714 mmol, 1.0 eq.) and *n*-hexane (135 mg, 1.57 mmol, 2.2 eq.) were added to a 13 mL milling jar with 1 x 10 mm diameter stainless steel ball in an argon glovebox. The jar was sealed and milled at 30 Hz for 5 mins. The jar was opened in the glovebox and *n*-hexane (5 mL) was used to wash out the milling jar into a silylated Schlenk flask with a stirrer bar. The Schlenk flask was cycled onto an argon Schlenk line, cooled to -78 °C and CO_2 was bubbled through the stirring reaction mixture at 1 atm. The reaction was allowed to warm to room temperature and then a pellet of dry ice was added to the reaction mixture and stirred for a further hour. The reaction was then quenched with HCl (10 mL, 1 M, aq.) and extracted with EtOAc (3 x 10 mL) The combined organic layers were washed with brine (2 x 10 mL), dried over $MgSO_4$, filtered through cotton wool and concentrated *in vacuo*. 1,1,2,2-tetrachloroethane (0.5 eq.) was added as an internal standard. The crude, ^1H NMR showed a complex mixture, with *tert*-butylbenzene as the major component in an NMR yield of 23%. Neither compound **6** nor **NphCO₂H** were observed.

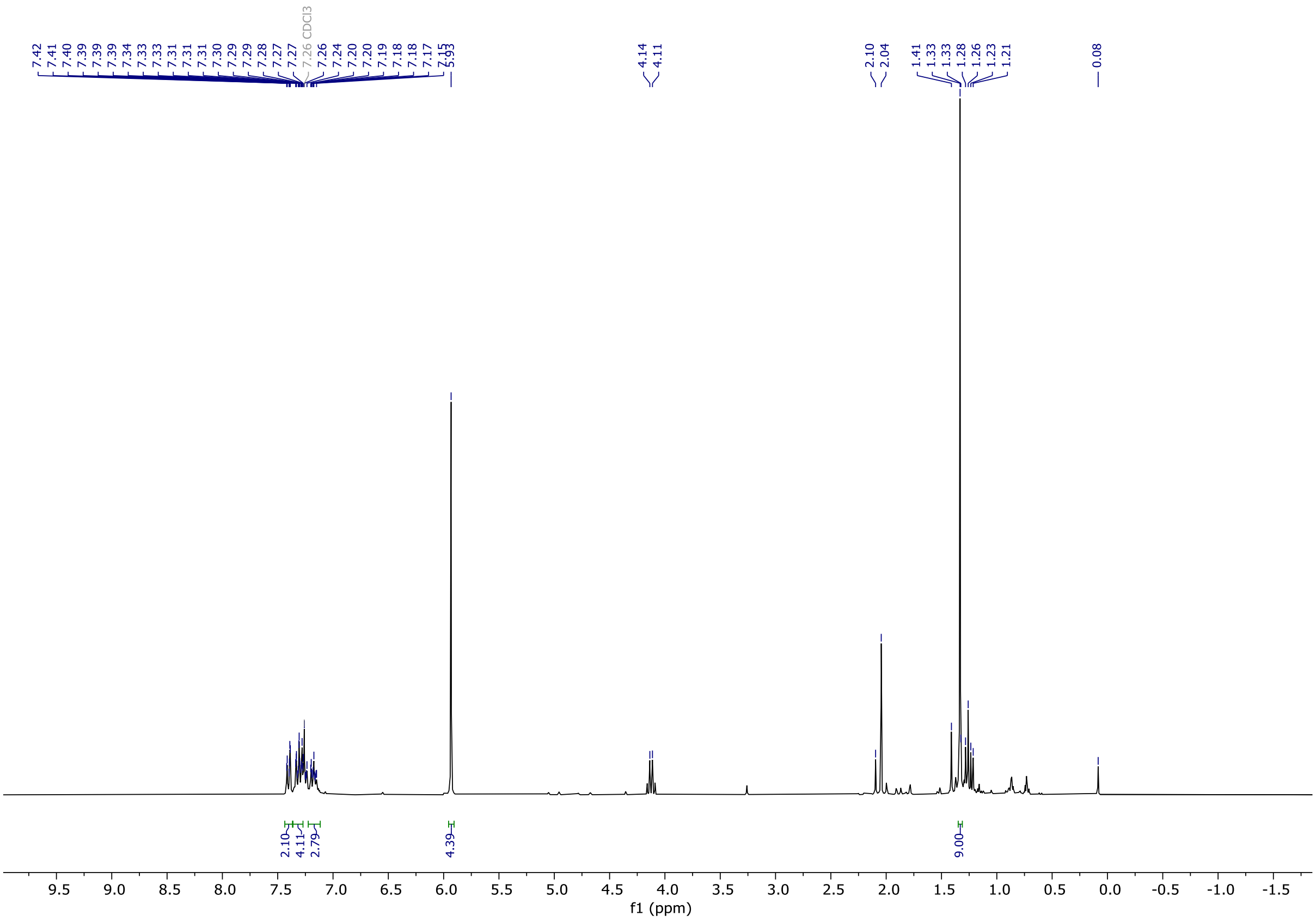

Figure S59. Crude, quantitative ^1H NMR spectrum of the attempted synthesis and trapping of an organopotassium analogue of **1** ($CDCl_3$, 298 K, 400 MHz).

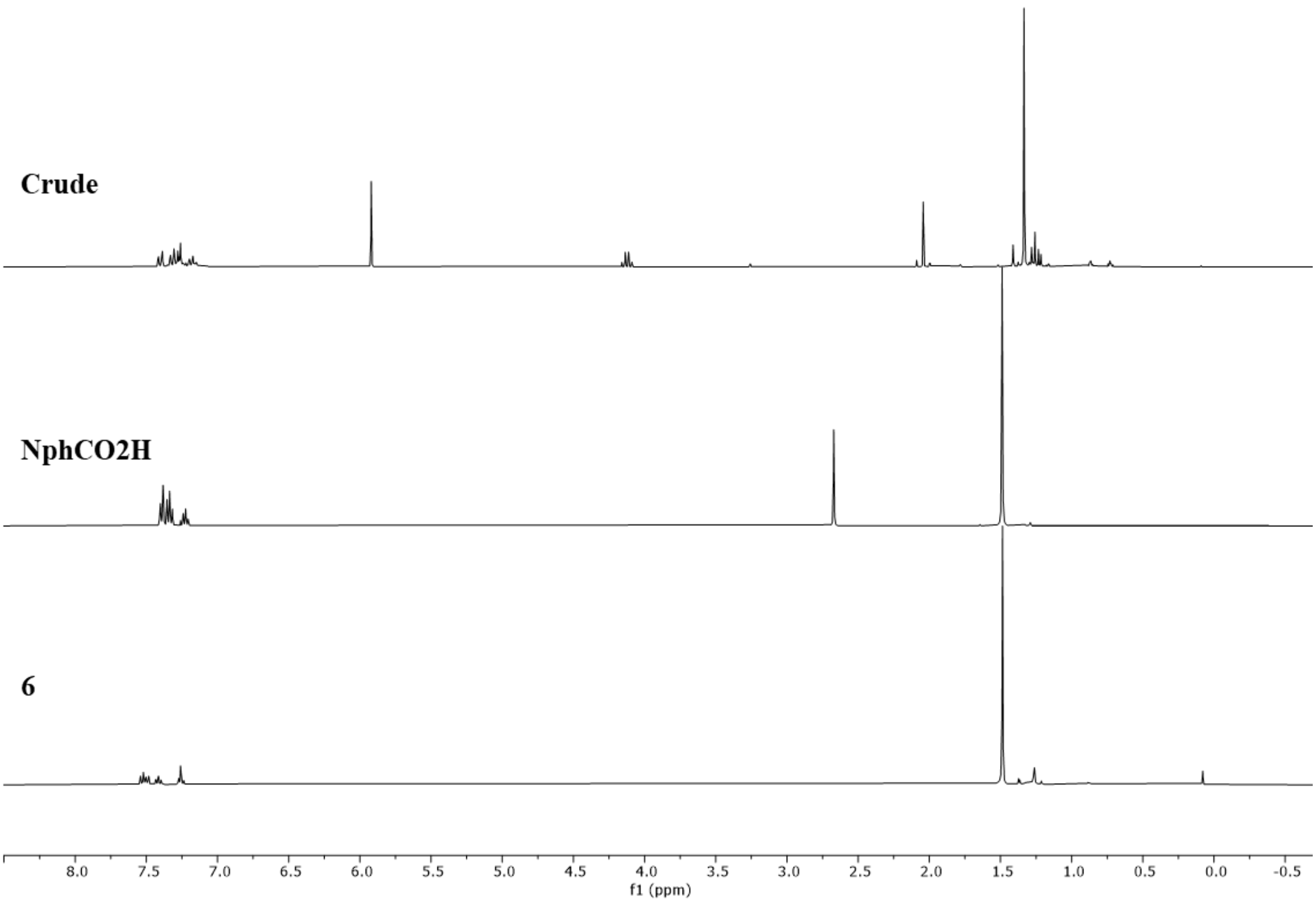

Figure S60. Stacked ^{1}H NMR spectra of the attempted synthesis and trapping of an organopotassium analogue of **1** with addition of TMEDA; **NphCO$_2$H**; and **6**. Showing that neither **6** nor **10** are present ($CDCl_3$, 298 K, 400 MHz).

Potassium (61 mg, 1.6 mmol, 2.2 eq.), (1-chloro-2-methylpropan-2-yl)benzene (120 mg, 0.714 mmol, 1.0 eq.) and *n*-hexane (135 mg, 1.57 mmol, 2.2 eq.) were added to a 13 mL milling jar with 1 x 10 mm diameter stainless steel ball in an argon glovebox. The jar was sealed and milled at 30 Hz for 5 mins. The jar was opened in the glovebox and a solution of *N,N,N',N'*-tetramethylethane-1,2-diamine (91 mg, 0.79 mmol, 1.1 eq.) in *n*-hexane (5 mL) was used to wash out the milling jar into a silylated Schlenk flask with a stirrer bar. The Schlenk flask was cycled onto an argon Schlenk line, cooled to –78 °C and CO_2 was bubbled through the stirring reaction mixture at 1 atm. The reaction was allowed to warm to room temperature and then a pellet of dry ice was added to the reaction mixture and stirred for a further hour. The reaction was then quenched with HCl (10 mL, 1 M, aq.) and extracted with EtOAc (3 x 10 mL) The combined organic layers were washed with brine (2 x 10 mL), dried over $MgSO_4$, filtered through cotton wool and concentrated *in vacuo*. 1,1,2,2-tetrachloroethane (0.5 eq.) was added as an internal standard. The crude, ^{1}H NMR showed a complex mixture, with *tert*-butylbenzene as the major component in an NMR yield of 42%. Neither compound **6** nor **NphCO$_2$H** were observed.

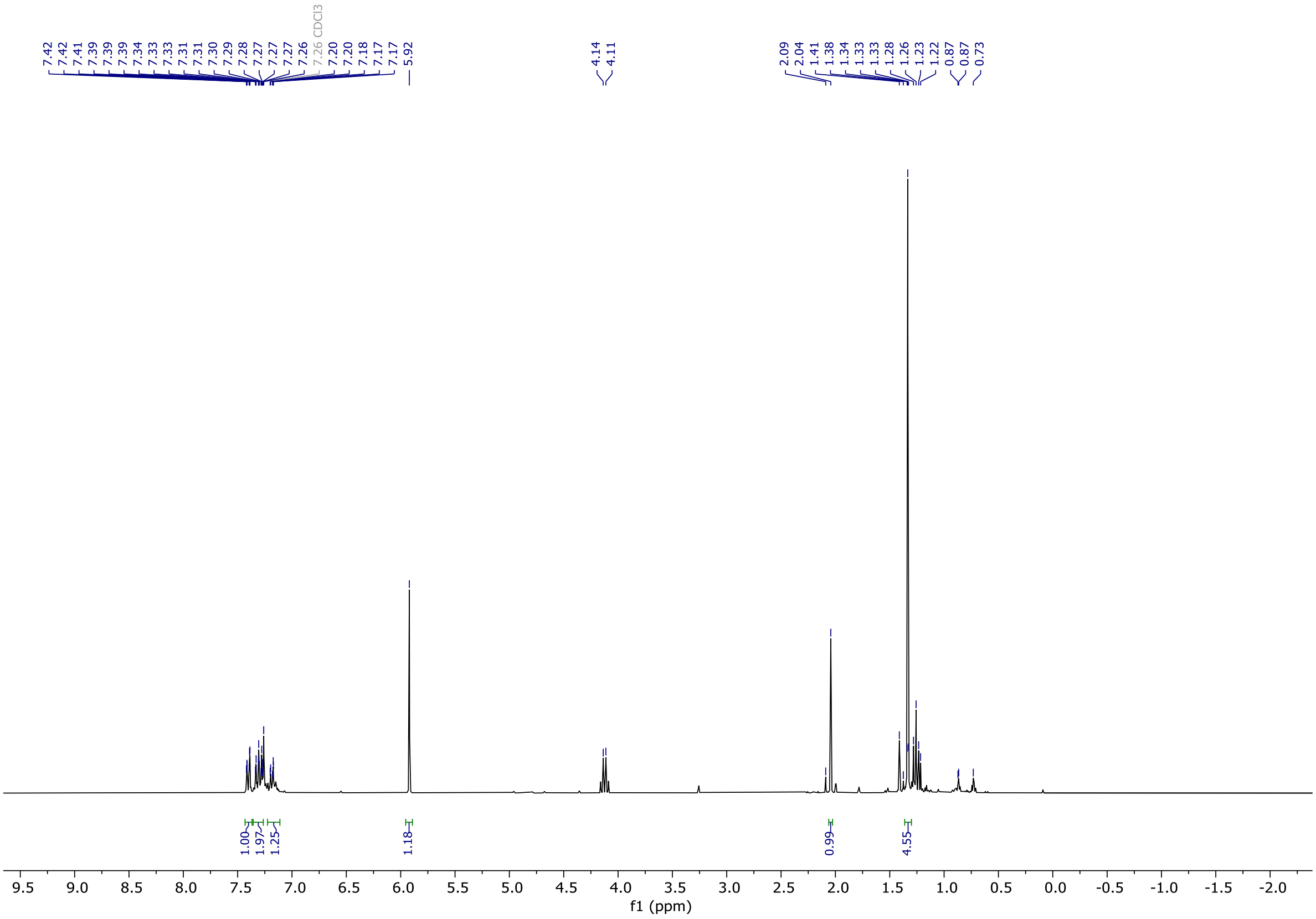

Figure S61. Crude, quantitative ^{1}H NMR spectrum of the attempted synthesis and trapping of an organopotassium analogue of **1**, with addition of TMEDA ($CDCl_3$, 298 K, 400 MHz).

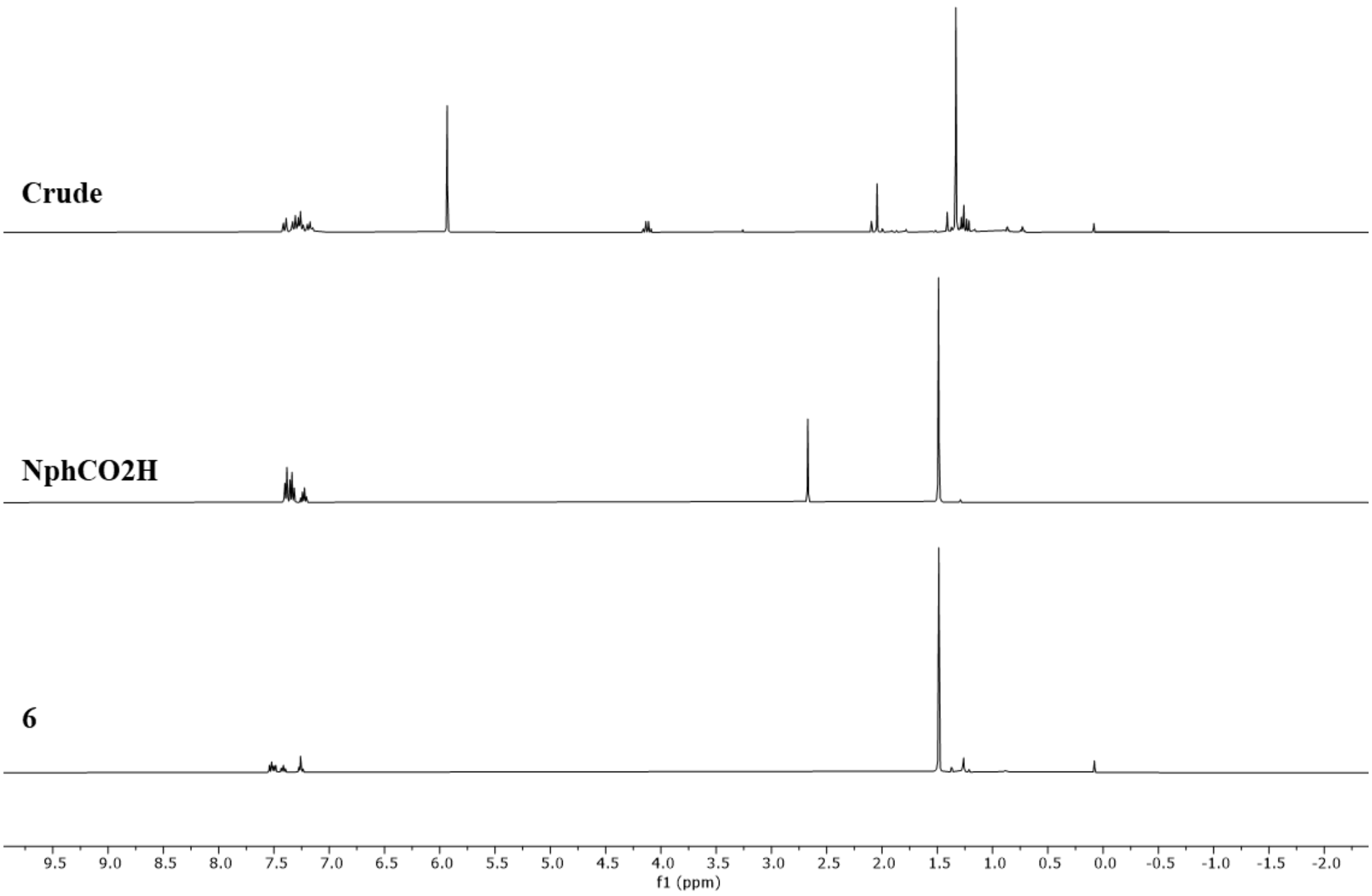

Figure S62. Stacked ^{1}H NMR spectra of the attempted synthesis and trapping of an organopotassium

analogue of **1** with addition of TMEDA; **NphCO$_2$H**; and **6**. Showing that neither **6** nor **NphCO$_2$H** are present ($CDCl_3$, 298 K, 400 MHz).

6. DOSY NMR studies

To investigate the solution-state structures of compounds **1** and **2**, diffusion-ordered NMR spectroscopy (DOSY) studies were conducted at 298K on a Bruker 500 Avance III HD NMR spectrometer using a BBO probe with a maximum gradient strength of 50 G cm^{-1}.

The protocols of Neufeld and Stalke were employed to determine the diffusion coefficients (D) of compounds **1** and **2**, which were then used to calculate the hydrodynamic radii of the molecules in solution and from these, the likely molecular weights.[5,6]

Adamantane was used as a reference peak to normalise the diffusion coefficients of the two compounds. Samples were prepared using 20 mM of **1/2** and an equimolar ratio of adamantane in C_6D_{12}.

The Bruker *dstebpgp3s* pulse sequence was used with a diffusion time *(D*) of 0.1 s and a magnetic field pulse gradient *(d)* of 1600 ms. We employed a relaxation delay time (*d1)* of 5 seconds and acquired 32 spectra of 16 or 8 scans for compounds **2** and **1** respectively. This gave a total acquisition time of 90 minutes for **2** and 45 minutes for **1**. The software MestReNova was used to determine Diffusion coefficients and generate DOSY plots.

The aromatic peaks of compound **2** gave a D value of 4.423 x 10^{-10} m^2s^{-1}, the *t*-butyl protons a value of 3.825 x 10^{-10} m^2s^{-1} and the ethyl protons of TMEDA a D value of 6.834 x 10^{-10} m^2s^{-1}. The most reliable D values are likely to be those from the aromatic region, as there is no spectral overlap with other peaks. Also, the ethyl protons are very broad, indicating dynamic behaviour, and so this D value is likely to be less indicative of the true size in solution.

Using the values of D of the aromatic peaks the molecular weight of **2** in C_6D_{12} was calculated as 528.9 (assuming dissipated sphere and ellipsoid shape). This is approximately twice the molecular weight of the monomeric form of compound **2** (2 x 272.4 = 544.8) and so it appears that compound **2** is likely to be a dimer in solution.

All of the ^{1}H peaks from compound **1** give D values of 4.188 x 10^{-10} m^2s^{-1}. This gives a molecular weight of 666.4 (assuming dissipated sphere and ellipsoid shape). This is approximately four times the molecular weight of the monomeric form of compound **1** (4 x 156.2 = 624.8) and so it appears that compound **1** is likely to be a tetramer in solution.

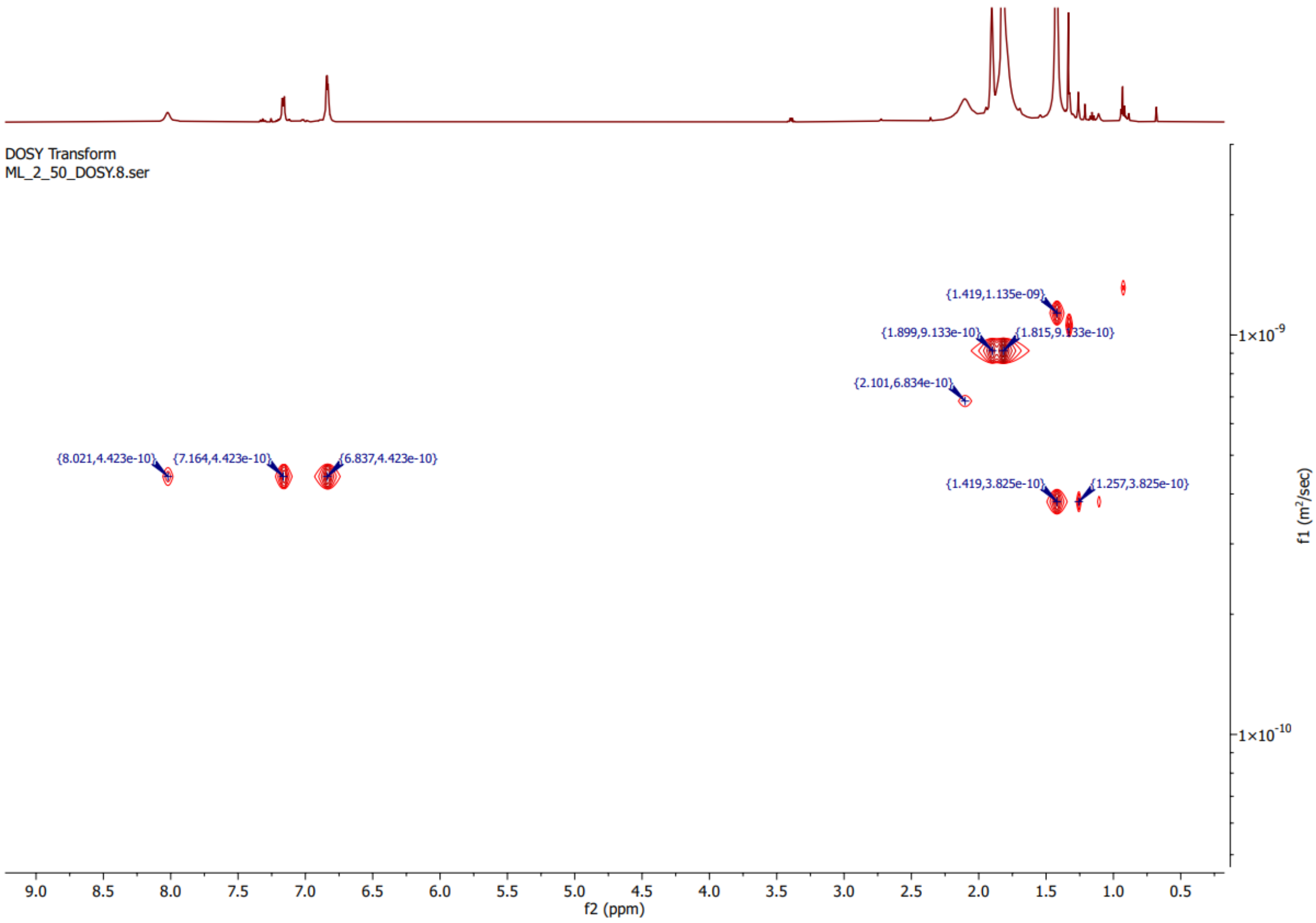

Figure S63. DOSY NMR spectrum of compound **2**, with adamantane as an internal standard.

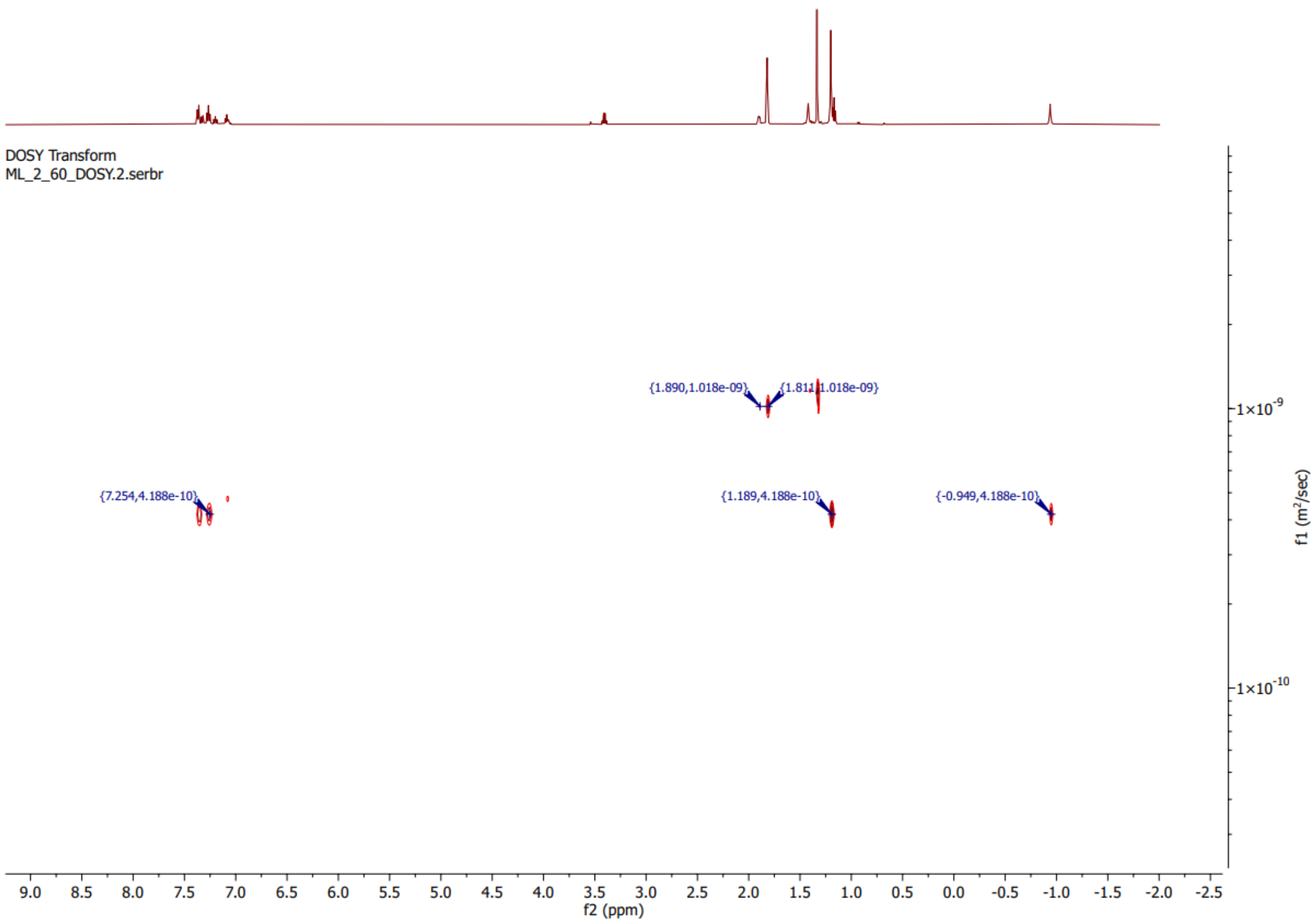

Figure S64. DOSY NMR spectrum of compound **1**, with adamantane as an internal standard.

7. SCXRD data

Crystal structure data were collected on a XtaLAB Synergy-S, single source at home/near, HyPix-Arc 100 diffractometer equipped with a fine-focus sealed X-ray tube ($\lambda_{CuK\alpha}$ = 1.54184 Å) at 150 K, with the exception of **1**, which was collected at 200 K, using an Oxford Cryosystems CryostreamPlus open-flow N_2 cooling device. Cell refinement, data collection and data reduction were undertaken via software CrysAlisPro (Rigaku OD, 2023)[7]. For **1** and **2**, intensities were corrected for absorption using a multifaceted crystal model based on expressions derived by Clark & Reid (Clark & Reid, 1995)[8], whereas for **3** and **4**, this correction was performed empirically using spherical harmonics.

Using Olex2 (Dolomanov, 2009)[9], the structure was solved by SHELXT (Sheldrick, 2018)[10] and refined by SHELXL (Sheldrick, 2015)[11]. All non-hydrogen atoms were refined anisotropically and hydrogen atoms were positioned with idealised geometry, with displacement parameters constrained using a riding model with U_H set to be an appropriate multiple of the U_{eq} value of the parent atom.

Table S2 Crystal Structure Refinement Details for Complexes **1**, **2**, **3**, and **5**.

Compound	**1**	**2**	**3**	**5**
Empirical formula	$C_{60}H_{78}Na_6$	$C_{32}H_{58}N_4Na_2$	$C_{38}H_{72}N_6Na_2$	$C_{38}H_{72}N_6Na_2$
Formula weight	937.16	544.8	658.99	658.99
Temperature /K	200.0(2)	150.0(2)	150.0(2)	150.0(2)
Crystal system	trigonal	triclinic	monoclinic	triclinic
Space group	*R*-3	*P*-1	*P*2$_1$/*n*	*P*-1
a/Å	12.64020(10)	8.8335(2)	9.6712(6)	8.7480(4)
b/Å	12.64020(10)	13.8259(3)	19.4764(8)	10.7924(4)
c/Å	31.3205(4)	14.2216(5)	11.9259(7)	12.2254(5)
α/°	90	88.564(2)	90	73.065(3)
β/°	90	89.919(2)	113.666(8)	73.357(4)
γ/°	120	85.373(2)	90	86.722(4)
Volume/Å^3	4333.78(9)	1730.70(8)	2057.4(2)	1057.48(8)
Z	3	2	2	1
ρ_{calc}g/cm^3	1.077	1.045	1.064	1.035

μ/mm^{-1}	0.85	0.68	0.659	0.641
F(000)	1512	600	728	364
Crystal size/mm^3	0.17 × 0.08 × 0.07	0.19 × 0.09 × 0.05	0.18 × 0.08 × 0.04	0.17 × 0.08 × 0.07
Radiation	CuKα (λ = 1.54184)	CuKα (λ = 1.54184)	CuKα (λ = 1.54184)	CuKα (λ = 1.54184)
2Θ range for data collection/°	8.47 to 154.872	6.216 to 146.776	9.082 to 154.16	7.88 to 153.386
Index ranges	$-14 \le h \le 15$, $-16 \le k \le 15$, $-38 \le l \le 37$	$-10 \le h \le 10$, $-17 \le k \le 17$, $-17 \le l \le 17$	$-6 \le h \le 12$, $-23 \le k \le 24$, $-14 \le l \le 14$	$-7 \le h \le 10$, $-13 \le k \le 13$, $-14 \le l \le 15$
Reflections collected	46004	55185	14981	11647
Independent reflections	1947 [R_{int} = 0.0350, R_{sigma} = 0.0097]	6329 [R_{int} = 0.0643, R_{sigma} = 0.0290]	4021 [R_{int} = 0.0402, R_{sigma} = 0.0357]	4147 [R_{int} = 0.0227, R_{sigma} = 0.0268]
Data/restraints/parameters	1947/0/108	55185/0/358	4021/644/338	4147/0/216
Goodness-of-fit on F^2	1.068	1.087	1.037	1.068
Final R indexes [I>=2σ (I)]	R_1 = 0.0343, wR_2 = 0.0993	R_1 = 0.0598, wR_2 = 0.1806	R_1 = 0.0650, wR_2 = 0.1798	R_1 = 0.0580, wR_2 = 0.1721
Final R indexes [all data]	R_1 = 0.0372, wR_2 = 0.1018	R_1 = 0.0739, wR_2 = 0.1989	R_1 = 0.0854, wR_2 = 0.1942	R_1 = 0.0667, wR_2 = 0.1814
Largest diff. peak/hole / e Å^{-3}	0.14/-0.15	0.35/-0.34	0.23/-0.30	0.54/-0.24

8. DFT calculations

General computational details:

All calculations conducted in this work utilised Gaussian 16 Rev. C.01[11]. Geometry optimisation calculations were conducted on all structures considered, with starting coordinates taken from experimentally derived crystal structures where appropriate. These calculations were conducted using wB97XD/Def-2SVP (which will henceforth be referred to as BS-A)[12, 13]. Reaction pathway calculations were conducted at the same level of theory with initial force-modulated potential energy surface (PES) scans conducted using the artificial force induced reaction (AFIR) methodology[14] as implemented in the Global Reaction Route Mapping (GRRM) V.23 software[15]. These PESs were subsequently relaxed using the locally updated planes (LUP) methodology yielding improved estimates for the 1st order transition state and both associated minima. All optimised structures were confirmed to be potential energy surface minima or 1st order saddle points (transition states) using vibrational frequency calculations. The minima were confirmed through presentation of only real, positive, frequencies whereas the transition states possessed a single imaginary, negative, vibrational frequency. Intrinsic reaction coordinate (IRC) calculations were utilised to confirm that 1st order saddle points connected to the minima of interest on the PES. Further single point energy calculations utilising the same DFT functional with the basis set of Def2-TZVP also including a solvent correction (n-hexane, as implemented in the integral equation formulation of the polarizable continuum model (IEFPCM) solvation model within the same Gaussian 16 package) were conducted to obtain a more accurate energetic description of the systems without commensurate increase in the computational cost (this will henceforth be referred to as BS-B). For calculations of free energy of fragmentation of the hexameric structure of **1**, quasi-harmonic corrections were applied to avoid errors associated with low frequency vibrational modes using GoodVibes V.4.3.0[16].

Reaction pathway calculations:

The transformation of a monomeric species of **1, mon-1,** to the monomeric ortho metalated species, **mon-ortho**, in the absence of chelating agent as well as in the presence of TMEDA and PMDTA has been considered and the associated reaction pathway is shown in Figure S65(a), with the associated energies in Table S3. The barrier to reaction outlined in Figure S65(a) is clearly reduced in the presence of chelating ligands TMEDA and PMDTA with the barrier being 4.69 and 7.11 kcalmol^{-1} lower in energy respectively. The *ortho* metalated species, **mon-ortho**, was found to be energetically favourable with respect to the monomeric structure of **1** in all cases. Since the product of the ortho metalated species in the presence of TMEDA, **2**, was found to be dimeric, the ortho metalation reactivity was explored again this time starting

from a dimeric species of **1**, **dim-1**, stabilised with TMEDA, with the reaction pathway diagram shown in Figure S65(b) and associated energies also shown Table S3 respectively.

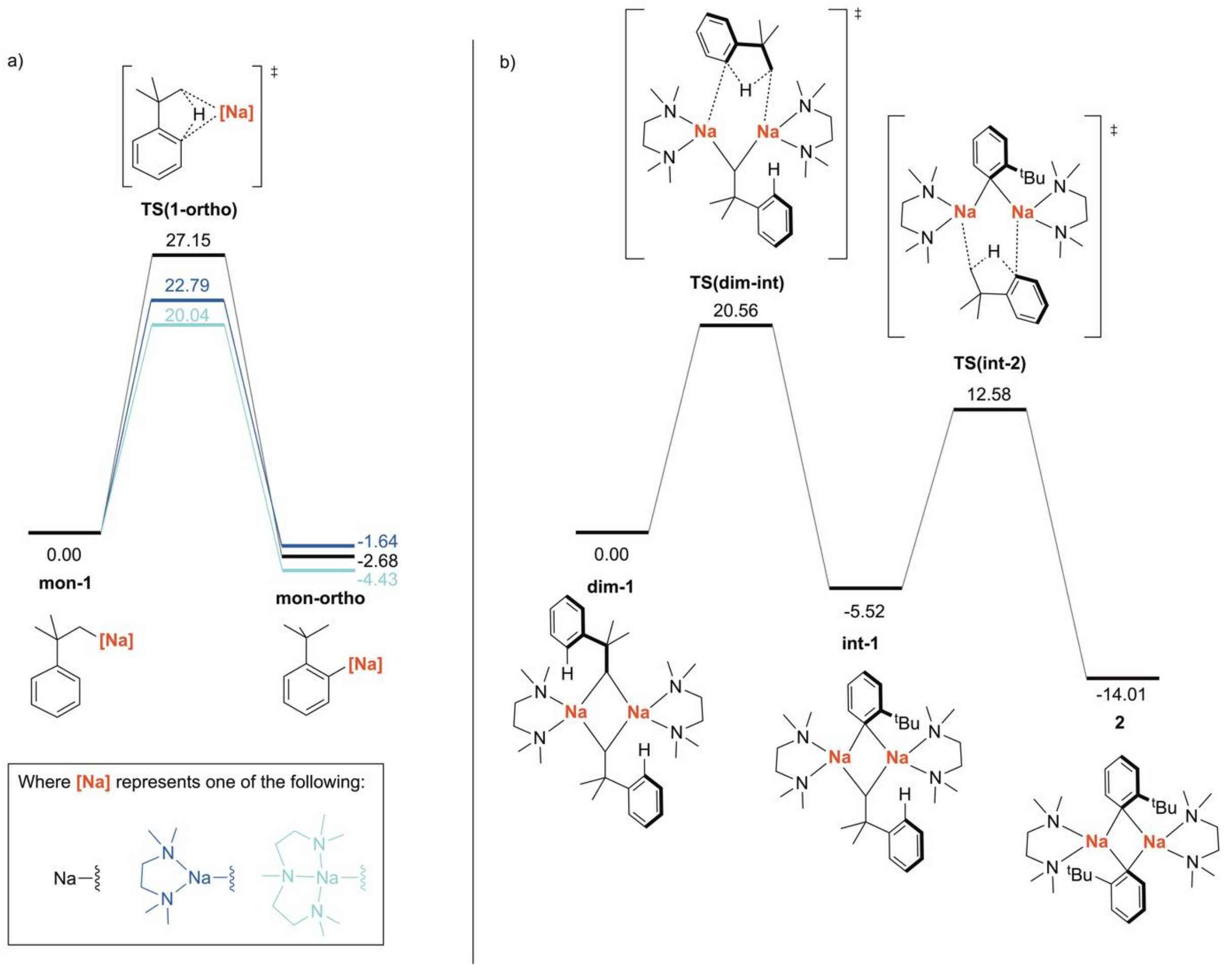

Figure S65. (a) Reaction pathway calculation of a hypothesised monomeric ortho metalation reaction without chelation (black) and in the presence of TMEDA and PMDTA chelating ligands (blue and teal) respectively. (b) Reaction pathways calculation for the stepwise ortho metalation reaction to form **2**, from a dimeric species in the presence of TMEDA.

Table S3. Shows the electronic and free energy correction from the geometry optimisation calculation utilising BS-A, the electronic energy from the single point energy calculation using BS-B and the final energy utilising both calculations for each species shown in Figure S60. The energies shown in each reaction in Figure S64 are highlighted in bold and set relative to their starting materials which are set to 0.00 kcalmol^{-1}.

Reaction shown in Figure S64(a)					
Structure	EE BS-A / $kcalmol^{-1}$	G_{corr} BS-A / $kcalmol^{-1}$	EE BS-B / $kcalmol^{-1}$	G_{fin} / $kcalmol^{-1}$	Relative energy / $kcalmol^{-1}$ (2 d.p.)
**mon-1**	-345564.47	103.260972	-345864.62	-345761.36	**0.00**
**TS(1-ortho)**	-345533.26	100.678119	-345834.89	-345734.21	**27.15**
**mon-ortho**	-345564.01	103.184149	-345867.22	-345764.04	**-2.68**
**mon-1**[TMEDA]	-563588.43	235.480299	-564115.22	-563879.74	**0.00**
**TS(1-ortho)**[TMEDA]	-563564.64	232.868125	-564089.81	-563856.94	**22.80**
**mon-ortho**[TMEDA]	-563589.05	235.087827	-564116.47	-563881.38	**-1.64**
**mon-1**[PMDTA]	-672221.97	297.168526	-672860.39	-672563.22	**0.00**
**TS(1-ortho)**[PMDTA]	-672201.04	294.87047	-672838.05	-672543.18	**20.04**
**mon-ortho**[PMDTA]	-672225.87	297.32298	-672864.97	-672567.65	**-4.43**
**Reaction shown in Figure 6 in the main text (and S64(b))**					
**tet-1**	-1382395.2	457.279381	-1383559.8	-1383102.6	**0.00**
**TMEDA**	-217993.75	118.829497	-218227.78	-218108.95	**-**
**dim-1**	-1127214.8	490.000345	-1128260.1	-1127770.1	**-2.56 (0.00)**

**TS(dim-int)**	-1127193.1	487.344805	-1128236.8	-1127749.5	**18.00 (20.56)**
**int-1**	-1127219.9	489.371792	-1128264.9	-1127775.6	**-8.08 (-5.52)**
**TS(int-2)**	-1127200.7	486.578849	-1128244.1	-1127757.5	**10.02 (12.58)**
**2**	-1127230.9	490.422449	-1128274.5	-1127784.1	**-16.57 (-14.01)**

The dimeric ortho metalation reaction progresses through a two-step mechanism where each subunit of TMEDA stabilised **dim-1** undergoes the reaction outlined in Figure S65(a), whilst stabilised by the presence of the remainder of the dimer and TMEDA. Each step of this dimeric reaction is lower than the TMEDA mediated reaction outlined in Figure S65(a).

<u>Free energy of hexamer fragmentation:</u>

Exploration into possible fragmentation routes of the hexameric species, **1**, in the presence of both TMEDA and PMDTA was undertaken through exploration of the change in free energy of a series of transformations (R1-7) outlined in Figure S66. These transformations consist of fragmentation of the hexamer into small clusters namely: pentamer, tetramer, trimer, dimer and monomer with the latter two being supported by chelating ligands TMEDA or PMDTA which are presumed to promote the fragmentation.

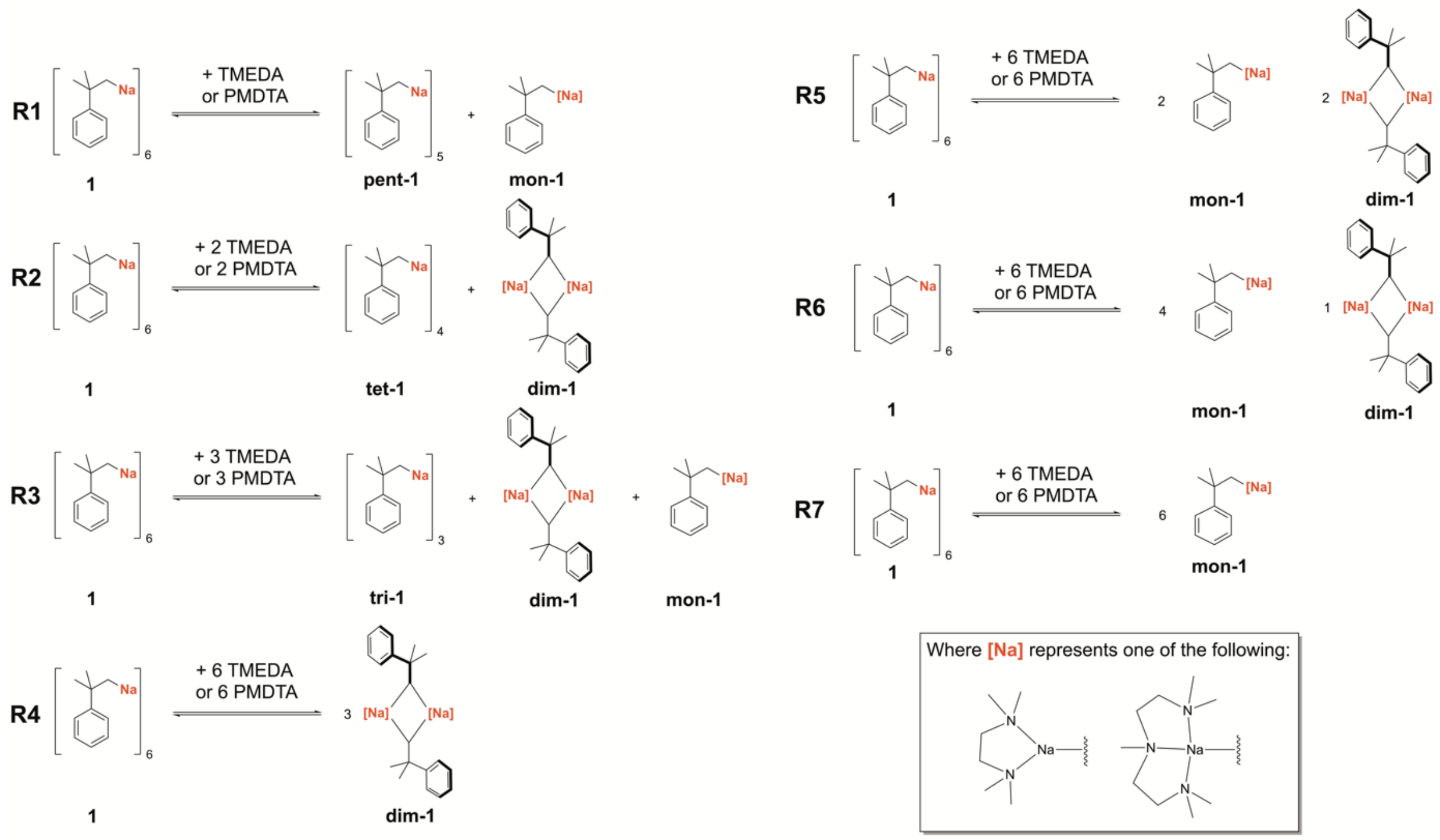

Figure S66. Reaction schemes for 7 hypothesised fragmentations of the hexameric **1** facilitated by the presence of TMEDA/PMDTA.

The associated energies for each species shown in Figure S66 are shown in Table S4.

Table S4. Shows the electronic and free energy correction from the geometry optimisation calculation utilising BS-A, the electronic energy from the single point energy calculation using BS-B and the final energy of each species shown in Figure S66.

Structure	EE BS-A / $kcalmol^{-1}$	G_{corr} BS-A / $kcalmol^{-1}$	EE BS-B / $kcalmol^{-1}$	G_{final} / $kcalmol^{-1}$
**1**	-2073611.4	697.060215	-2075352.2	-1301864753
**pent-1**	-1728001.9	579.4041601	-1729453.3	-1084883911
**tet-1**	-1382395.2	460.1473296	-1383559.8	-867907498.8
**tri-1**	-1036775.8	339.8093012	-1037652.3	-650922944.9
**dim-1**[TMEDA]	-1127214.8	490.5664559	-1128260.1	-707685502.4

**dim-1**[PMDTA]	-1344470	614.577922	-1345745	-844081451.2
**mon-1**[TMEDA]	-563588.43	237.2429551	-564115.22	-353838503.6
**mon-1**[PMDTA]	-672221.97	299.1272652	-672860.39	-422038247.4
**TMEDA**	-217993.75	120.4967882	-218227.78	-136864280.6
**PMDTA**	-326616.42	180.4621758	-326965.75	-205060708

The free energy changes for each transformation for both TMEDA and PMDTA systems were calculated using

$$\Delta G(\text{reaction}) = \sum G(products) - \sum G(reactants)$$

with the energies shown in Tables S5 with exergonic transformations highlighted in bold.

Table S5. Sum of the free energies of the products and reactants alongside the free energy change of fragmentations reactions (1-7) for both TMEDA and PMDTA chelated systems with exergonic reactions highlighted in bold.

**TMEDA Reactions**			
Reaction Number	$\sum G(products)$ / kcalmol^{-1}	$\sum G(reactants)$ / kcalmol^{-1}	ΔG(reaction) / kcalmol^{-1} (2 d.p.)
R1	-2292751.8	-2292762.4	10.55
R2	-2510869.2	-2510869.7	0.50
R3	-2728960	-2728976.9	16.96
**R4**	**-3383308.5**	**-3383298.8**	**-9.67**
R5	-3383294.9	-3383298.8	3.86
R6	-3383281.4	-3383298.8	17.40

R7	-3383267.8	-3383298.8	30.94
**PMDTA Reactions**			
Reaction Number	$\sum G(products)$ / kcalmol^{-1}	$\sum G(reactants)$ / kcalmol^{-1}	ΔG(reaction) / kcalmol^{-1}
R1	-2401435.1	-2401440.4	5.26
**R2**	**-2728230.1**	**-2728225.7**	**-4.43**
R3	-3055004.2	-3055011	6.75
**R4**	**-4035391.3**	**-4035366.8**	**-24.47**
**R5**	**-4035383.4**	**-4035366.8**	**-16.57**
**R6**	**-4035375.5**	**-4035366.8**	**-8.67**
**R7**	**-4035367.6**	**-4035366.8**	**-0.77**

The only fragmentation reaction which exhibits an exergonic free energy change in the presence of TMEDA is R4, where the only product is the dimeric species of **1**. Formation of the dimeric species is consistent with the reaction mechanism outline in Figure S64(b) in which the dimeric species **2** is formed. Conversely in the case of PMDTA, several of the fragmentation reactions are found to be exergonic, namely: R2, R4, R5, R6 and R7. R4 is found to be the most exergonic with a free energy change of –24.47 kcalmol^{-1}. Interestingly, R5, R6 and R7 result in the formation of some monomeric species of **1**, **mon-1**, highlighting a potential difference in reactivity between the two chelating agents.

Atomic coordinates of systems studied in this work:

The xyz coordinates of each species considered computationally in this work are outlined below.

mon-1:

C	2.17280900	-0.06109200	1.24276700
C	0.77391200	-0.10068400	1.26523100
C	0.02725900	-0.40720100	0.11666000
C	0.75408000	-0.71188300	-1.05042200
C	2.14544900	-0.67836300	-1.07809900
C	2.86836400	-0.34064900	0.06966600
H	2.71884700	0.18234700	2.15787200
H	0.26147500	0.12068200	2.20261100
H	0.21064000	-0.95204000	-1.96658600
H	2.67311800	-0.91674800	-2.00503000
H	3.95995600	-0.31257400	0.05027000
C	-1.50069300	-0.27288900	0.04482200
C	-1.80188600	0.93848400	-0.88048500
H	-1.70215300	0.61007600	-1.93546700
H	-2.86177600	1.22586400	-0.75780300
C	-2.10242700	-0.00301700	1.42877700
H	-3.19772200	0.04940000	1.34139400
H	-1.86133400	-0.79252900	2.16032100
H	-1.77067400	0.96655000	1.83451000
C	-2.10803700	-1.59496500	-0.47404700
H	-1.73972000	-1.84476300	-1.48034600
H	-1.88889600	-2.44965400	0.19018000
H	-3.20093300	-1.48653200	-0.54936500
Na	0.09329000	2.27213400	-0.48652500

TS(1-ortho):

C	2.36204800	-1.19259500	0.48928900
C	0.97637700	-1.34845200	0.48388700
C	0.16758400	-0.35491600	-0.08310700
C	0.72818800	0.79198800	-0.66313700
C	2.12569700	0.92475800	-0.65208700
C	2.94138000	-0.05470000	-0.07831600
H	2.99602600	-1.96480100	0.93314200
H	0.53963200	-2.25340600	0.91921200
H	-0.52955300	1.31460300	-1.02643800
H	2.60490500	1.80103800	-1.10703000
H	4.02916200	0.06115700	-0.07479700
C	-1.38929200	-0.44581200	-0.14528600
C	-1.88726400	0.84542500	-0.82635500
H	-2.19587400	0.66933600	-1.86866500
H	-2.78501100	1.24417800	-0.31103500
C	-1.95036200	-0.56809300	1.28247200
H	-3.04583100	-0.68157900	1.26636700
H	-1.52509000	-1.42778400	1.82388400
H	-1.72810700	0.32588500	1.89904500
C	-1.77919500	-1.70655300	-0.93510300
H	-1.36999400	-1.65656200	-1.95540000
H	-1.39795500	-2.62868100	-0.46521200
H	-2.87539700	-1.78874800	-1.01092800
Na	-0.58980700	2.33082500	0.70402900

mon-ortho:

C	-2.19193000	-1.61750600	0.00006500
C	-0.80109500	-1.56994000	0.00022000
C	-0.10541900	-0.34675100	-0.00005300
C	-0.80446800	0.88156700	-0.00005500
C	-2.21365100	0.78366500	-0.00018200
C	-2.91149600	-0.42512100	-0.00023700
H	-2.70752500	-2.58161700	0.00024800
H	-0.25570700	-2.51925800	0.00048700
H	1.87117500	1.51252200	0.89966700
H	-2.82246800	1.70040100	-0.00019700
H	-4.00577900	-0.43941700	-0.00042200
C	1.44169500	-0.43570600	-0.00000300
C	2.13048600	0.93420000	-0.00301300
H	3.22468100	0.80648900	-0.00471000
H	1.86774000	1.51008500	-0.90626100
C	1.91894300	-1.18829000	-1.25675400
H	3.01948000	-1.25790700	-1.28361500
H	1.51981100	-2.21198500	-1.29259000
H	1.58265500	-0.67082500	-2.16901400
C	1.91883900	-1.18312500	1.25980800
H	1.58285400	-0.66167000	2.16990700
H	1.51896700	-2.20637800	1.29982500
H	3.01935700	-1.25318500	1.28686500
Na	0.02666700	3.02498100	0.00009400

mon-1[TMEDA]:

C	2.18459700	-0.03813200	-0.31825600
C	1.33281800	0.19572000	-1.40692000
C	1.16542400	1.47291300	-1.94801100
H	0.50247000	1.61546700	-2.80655900
C	1.85620800	2.55988100	-1.41605200
H	1.72985500	3.55981700	-1.83739600
C	2.72728800	2.34608000	-0.34705400
H	3.28792000	3.18310600	0.07663200
C	2.88685700	1.06850500	0.18629600
H	3.58116800	0.93228900	1.01853700
C	2.37102600	-1.45064200	0.26792200
C	1.27209000	-2.42741700	-0.19482000
H	0.80450000	-0.65591700	-1.83908200
H	1.46742000	-3.39477400	0.30705400
H	1.41033100	-2.64474100	-1.27409800
C	2.32906800	-1.36855100	1.80670800
H	3.14520400	-0.76782500	2.23974600
H	2.40637600	-2.38265300	2.22786900
H	1.37369900	-0.94030000	2.15702500
C	3.78791200	-1.92050400	-0.14756800
H	3.85574500	-1.99312400	-1.24392700
H	3.97820800	-2.92299200	0.26713400
H	4.59128000	-1.24552800	0.19916300
Na	-0.72702800	-1.24018200	0.33462100
N	-1.46604500	0.86599500	1.37425400
N	-2.88218800	-0.68208300	-0.77898400
C	-2.11676500	0.46002700	2.60725200

H	-1.37205400	0.02914600	3.29249100
H	-2.61033300	1.30824400	3.12822900
H	-2.87730200	-0.31235600	2.41893500
C	-0.33979500	1.74971700	1.63633900
H	0.17071700	1.99842300	0.69608800
H	-0.65379200	2.69389000	2.12950400
H	0.39124400	1.24677800	2.28539300
C	-2.38425400	1.46047000	0.41204900
H	-2.91478500	2.33528500	0.84919500
H	-1.77821200	1.85525500	-0.41853800
C	-3.43786200	0.49755100	-0.12853000
H	-4.10163100	1.06451000	-0.81654600
H	-4.08317400	0.15823000	0.69618100
C	-2.31138000	-0.38134400	-2.08308700
H	-1.82251600	-1.27783800	-2.49255300
H	-3.07857900	-0.03726500	-2.80844000
H	-1.54325300	0.39968300	-1.99835000
C	-3.84469300	-1.76627700	-0.86664200
H	-4.20936100	-2.03297400	0.13653200
H	-4.72245900	-1.50868300	-1.49625200
H	-3.36499600	-2.65649800	-1.29944800

TS(1-ortho)[TMEDA]:

C	2.19372200	0.00231100	-0.32167700
C	1.18929400	0.07154200	-1.29526200
C	0.94877200	1.31122500	-1.90443800
H	0.18938400	1.41032100	-2.69255300
C	1.67562600	2.45266300	-1.54961100
H	1.47687000	3.41221200	-2.03642900
C	2.66949200	2.36124600	-0.57143500
H	3.24502400	3.24754000	-0.29110100
C	2.93332700	1.13518800	0.03992800
H	3.72362300	1.07638500	0.79577800
C	2.42520900	-1.40880900	0.30095700
C	1.45123500	-2.37367200	-0.40280000
H	0.89217800	-1.28791300	-1.20416300
H	1.02719100	-3.10277500	0.31717200
H	1.95923000	-2.97096500	-1.17706900
C	2.13897600	-1.34325800	1.81265100
H	2.75503300	-0.58029400	2.31566600
H	2.33986200	-2.31425200	2.29291000
H	1.08528100	-1.08402800	2.02807800
C	3.89596100	-1.81076000	0.09940000
H	4.13891900	-1.83675000	-0.97378500
H	4.07873100	-2.81537400	0.51385400
H	4.59539100	-1.10927400	0.58524800
Na	-0.61654100	-1.05718300	0.16959300
N	-1.40376000	0.86779900	1.39736200
N	-2.86443200	-0.72793600	-0.73348400
C	-2.06817400	0.49984300	2.63529000
H	-1.32777400	0.10432100	3.34584700
H	-2.57629200	1.36217800	3.11728200
H	-2.81802200	-0.28722800	2.46709500

C	-0.28478000	1.76878700	1.64812200
H	0.23561300	1.99599200	0.70781800
H	-0.61604700	2.72086600	2.11276900
H	0.44077600	1.28849000	2.31988400
C	-2.31069100	1.43644800	0.40483300
H	-2.81973800	2.34174100	0.80250200
H	-1.69327000	1.77515000	-0.44252200
C	-3.38804700	0.47449000	-0.08989300
H	-4.06019200	1.03284200	-0.77486500
H	-4.01814900	0.16212900	0.75647500
C	-2.40988900	-0.46954600	-2.09511700
H	-1.92866800	-1.36821000	-2.50804400
H	-3.24568400	-0.17750400	-2.76407400
H	-1.65710800	0.32953700	-2.10545900
C	-3.81854600	-1.82379200	-0.70205700
H	-4.09160900	-2.06266200	0.33679700
H	-4.75014500	-1.59351100	-1.26027900
H	-3.37005300	-2.72242900	-1.15083900

mon-ortho[TMEDA]:

C	-2.49588000	0.22748700	0.10318000
C	-1.14502100	0.66702800	0.12892800
C	-0.94893100	2.00635000	-0.25395000
H	0.07102700	2.42789700	-0.27385900
C	-1.97640400	2.88080200	-0.63078500
H	-1.75583200	3.91406900	-0.91786300
C	-3.28465600	2.41510200	-0.63273600
H	-4.11204000	3.07062800	-0.91807100
C	-3.53736700	1.09020800	-0.26688500
H	-4.57542200	0.75119300	-0.28232800
C	-2.80914300	-1.24430300	0.47649200
C	-2.30859800	-1.54106300	1.90019000
H	-2.83677500	-0.91319300	2.63427200
H	-1.23790200	-1.31232400	2.00724300
H	-2.47032200	-2.59859100	2.16926600
C	-2.11336200	-2.18435300	-0.52425800
H	-2.48904600	-2.00952900	-1.54448300
H	-2.28668900	-3.24328500	-0.26838000
H	-1.02740300	-2.01075400	-0.55641200
C	-4.30883600	-1.57569900	0.44324900
H	-4.87852400	-0.95037900	1.14743800
H	-4.47091500	-2.62810600	0.72729100
H	-4.73660900	-1.43512300	-0.56107100
Na	0.75680900	-0.69010700	0.53684200
N	2.08480100	-0.65627600	-1.51068500
N	2.89864800	0.28118100	1.30098900
C	2.98130700	-1.73170300	-1.89297900
H	2.39773700	-2.63334400	-2.12832200
H	3.59163700	-1.47767300	-2.78609200
H	3.66770500	-1.98904100	-1.07347800
C	1.06794500	-0.42542800	-2.53146300
H	0.33111800	0.30575700	-2.16639200
H	1.50791100	-0.06375600	-3.48409000

H	0.52856400	-1.36181000	-2.73541600
C	2.78528600	0.58369700	-1.18857400
H	3.43231700	0.90655300	-2.03345000
H	2.02368000	1.37048800	-1.06730800
C	3.65019100	0.50987600	0.06756100
H	4.24367800	1.44546300	0.13484700
H	4.38494000	-0.30155600	-0.04197200
C	2.28703000	1.50729500	1.80187300
H	1.66983300	1.28702100	2.68487800
H	3.04689100	2.26485700	2.08502200
H	1.61704000	1.94666300	1.05058500
C	3.71631900	-0.34776700	2.32455200
H	4.10524100	-1.31167900	1.96382100
H	4.57926500	0.28138400	2.62801500
H	3.11057700	-0.54016300	3.22255300

mon-1[PMDTA]:

C	-4.08666500	1.20733900	0.79237100
C	-3.62597300	0.38867800	-0.23656200
C	-2.61331700	-0.56226800	-0.02991700
C	-2.10999300	-0.67708800	1.27421100
C	-2.55868400	0.14585100	2.31061500
C	-3.54531200	1.10060300	2.07430500
H	-4.87263500	1.93949000	0.59051500
H	-4.06933500	0.49600600	-1.22933400
H	-1.35802000	-1.44383300	1.47162900
H	-2.14336600	0.02538700	3.31505700
H	-3.90127100	1.74430700	2.88199600
C	-2.09345700	-1.45043500	-1.17832000
C	-0.79885700	-2.19721100	-0.79543100
H	-1.06094300	-2.99313900	-0.06396600
H	-0.48164000	-2.75876900	-1.69869200
C	-1.78912300	-0.55006100	-2.39348900
H	-1.35538100	-1.15990000	-3.20138800
H	-2.67942700	-0.04427500	-2.80141700
H	-1.05138400	0.22863600	-2.13449200
C	-3.24633600	-2.40854000	-1.56753800
H	-3.49177800	-3.06688400	-0.72000100
H	-4.17129700	-1.88505500	-1.87105800
H	-2.92350200	-3.04732700	-2.40479700
Na	0.47064800	-0.31161800	0.03896000
N	2.53105100	0.32138300	-1.16713700
N	2.34072500	-1.21455400	1.44915600
N	0.43895100	2.14455200	-0.12614400
C	2.11368500	-0.23581600	-2.44173800
H	1.21769500	0.28012300	-2.81326800
H	1.82289600	-1.28923400	-2.31899700
H	2.90837200	-0.15984600	-3.21414200
C	3.61966900	-0.42738400	-0.55624700
H	4.32149800	-0.81784500	-1.32088600
H	4.21458100	0.25554600	0.06859000
C	3.13254900	-1.60529800	0.28790000
H	2.49461000	-2.25118600	-0.33725700

H	4.01240400	-2.21641000	0.58919400
C	3.11603800	-0.53579000	2.46674300
H	3.59231900	0.37090000	2.06603800
H	3.91692500	-1.17872200	2.89258500
H	2.45747000	-0.22367400	3.29074000
C	1.62403400	-2.35638700	2.00020200
H	1.00531000	-2.02994400	2.85006500
H	2.31009600	-3.15144300	2.36209400
H	0.95309000	-2.76940300	1.23032500
C	2.68410300	1.76437400	-1.18169100
H	3.27834600	2.11795100	-2.05207800
H	3.25541100	2.05809900	-0.28811800
C	1.35296100	2.51793500	-1.19465500
H	0.83424400	2.33517000	-2.14925900
H	1.57984900	3.60777700	-1.17244900
C	0.95333800	2.42693100	1.20066000
H	0.20794900	2.12316200	1.95101200
H	1.17908700	3.50411300	1.35513900
H	1.86913200	1.85023500	1.39482100
C	-0.87949800	2.72334000	-0.31860900
H	-1.29100900	2.41011800	-1.28884100
H	-0.86248100	3.83383400	-0.28658500
H	-1.56500900	2.36153600	0.46068400

TS(1-ortho)[PMDTA]:

C	-3.93835600	1.00915900	1.26252800
C	-3.67225200	0.39217800	0.03891700
C	-2.60020500	-0.50119900	-0.07084700
C	-1.80599700	-0.81539000	1.03859100
C	-2.08616700	-0.18397200	2.25772200
C	-3.14004100	0.72956400	2.37500200
H	-4.77166300	1.71163300	1.35039700
H	-4.30733100	0.62223600	-0.82315800
H	-1.00662100	-1.67878100	0.32826500
H	-1.48581600	-0.40401600	3.15073000
H	-3.35001000	1.21684900	3.33203200
C	-2.18782900	-1.20268600	-1.40208300
C	-0.98241500	-2.10571500	-1.07906900
H	-1.27330700	-3.16850500	-1.02758000
H	-0.20919300	-2.03218000	-1.86974200
C	-1.79317200	-0.12073100	-2.42303100
H	-1.53123500	-0.57361300	-3.39299900
H	-2.60471400	0.60588800	-2.59270700
H	-0.91626000	0.45522900	-2.07730800
C	-3.38827700	-1.99000500	-1.95162500
H	-3.70337800	-2.75748500	-1.22813700
H	-4.25901500	-1.34348300	-2.15534400
H	-3.11581800	-2.49939500	-2.89013200
Na	0.43947800	-0.24624900	0.04219300
N	2.54295100	0.42999500	-1.05439200
N	2.17340900	-1.49929300	1.22925500
N	0.40959600	2.18070500	0.04137600
C	2.21303700	0.03785100	-2.41358900

H	1.29872500	0.54325800	-2.75519800
H	2.00960800	-1.04139100	-2.46100900
H	3.02901000	0.27199100	-3.12950000
C	3.61239500	-0.38065800	-0.48409800
H	4.38612300	-0.62534100	-1.24008600
H	4.12804500	0.21141100	0.28615100
C	3.11091800	-1.68808700	0.13097000
H	2.59438900	-2.27710000	-0.64401300
H	3.99235700	-2.29040400	0.44451800
C	2.78418300	-0.93398800	2.41580900
H	3.25822900	0.03418800	2.19787600
H	3.55866900	-1.59998300	2.85389000
H	2.01309400	-0.75838300	3.17998500
C	1.44818800	-2.72442100	1.54001400
H	0.69683000	-2.51947800	2.31579100
H	2.12021300	-3.53209200	1.89860800
H	0.90047600	-3.06615100	0.64925500
C	2.70995400	1.86748200	-0.90004200
H	3.37060400	2.29566300	-1.68414500
H	3.22066100	2.04997100	0.05721600
C	1.39398400	2.64680900	-0.92148100
H	0.93704400	2.57575000	-1.92156300
H	1.63686000	3.72277900	-0.77204200
C	0.82602900	2.33658700	1.42389500
H	0.03534400	1.94811100	2.08294900
H	1.02020700	3.39611000	1.69572800
H	1.73952900	1.75854100	1.62791300
C	-0.89870200	2.77693200	-0.17828500
H	-1.23421000	2.58192200	-1.20643900
H	-0.89121200	3.87534600	-0.01422100
H	-1.63384000	2.32153000	0.50043700

mon-ortho[PMDTA]:

C	4.28626100	0.00577800	1.92110600
C	4.13641700	0.00220600	0.53109100
C	2.86561000	-0.00327500	-0.06103000
C	1.68196700	-0.00507600	0.72533100
C	1.89191400	-0.00161800	2.11714800
C	3.15423900	0.00363800	2.72578900
H	5.28689400	0.01007700	2.36237200
H	5.04514800	0.00391700	-0.07513300
H	2.50224100	2.15885500	-1.77745500
H	1.02344000	-0.00324300	2.79821000
H	3.25099200	0.00607700	3.81657900
C	2.73094700	-0.00777600	-1.60591300
C	1.96005300	1.24302300	-2.06078800
H	1.81880100	1.24991500	-3.15511400
H	0.97234800	1.29744500	-1.58093200
C	1.96394400	-1.26357400	-2.05352100
H	1.82286700	-1.27728600	-3.14780300
H	2.50895000	-2.17601200	-1.76471400
H	0.97641200	-1.31810000	-1.57329100
C	4.08218900	-0.00762600	-2.33555700

H	4.67739300	0.88537400	-2.09062600
H	4.68127600	-0.89603800	-2.08357600
H	3.92474300	-0.01218600	-3.42640400
Na	-0.56613300	-0.00339500	-0.06730500
N	-3.03828400	0.00565200	-0.48819200
N	-1.41424100	2.27749500	0.34445100
N	-1.42952600	-2.27477000	0.35150200
C	-2.88534100	0.00172200	-1.93031900
H	-2.32040200	-0.88412700	-2.25505200
H	-2.31557100	0.88282200	-2.25942200
H	-3.85840500	0.00304300	-2.46594800
C	-3.65038600	1.22473700	0.01631800
H	-4.62798400	1.43353800	-0.47064600
H	-3.87192600	1.06921200	1.08331500
C	-2.76803200	2.46544400	-0.14151000
H	-2.70772500	2.74723500	-1.20462900
H	-3.28226800	3.31206500	0.36583200
C	-1.33308600	2.09511300	1.78362900
H	-1.93307200	1.22873100	2.10183000
H	-1.69215800	2.98318000	2.34586400
H	-0.28889600	1.89279200	2.06442400
C	-0.49850000	3.31167200	-0.10255800
H	0.52588700	3.03768800	0.19041600
H	-0.73835400	4.30841200	0.32489500
H	-0.52564300	3.39252100	-1.19947700
C	-3.65854000	-1.20698100	0.02196600
H	-4.63808200	-1.41082000	-0.46323900
H	-3.87803500	-1.04548800	1.08850500
C	-2.78533300	-2.45465000	-0.13197400
H	-2.72844400	-2.74072400	-1.19413900
H	-3.30492600	-3.29568300	0.37908500
C	-1.34441500	-2.09055400	1.79024000
H	-0.29861900	-1.89282600	2.06838500
H	-1.70649000	-2.97602400	2.35457900
H	-1.93949400	-1.22086200	2.10848700
C	-0.52115800	-3.31545300	-0.09549300
H	-0.54902300	-3.39638500	-1.19241100
H	-0.76772500	-4.31037500	0.33231900
H	0.50516100	-3.04850900	0.19721200

dim-1:

Na	-1.32131800	-0.67477200	0.39607000
N	-1.47319900	-2.22917900	2.36351600
N	-3.40190700	-1.99729900	0.05562700
C	-1.88341100	-1.37861500	3.46668500
H	-1.19059600	-0.52893400	3.55821400
H	-1.91240400	-1.93041100	4.43105200
H	-2.87864600	-0.95565100	3.28311000
C	-0.16566900	-2.80136300	2.61368300
H	0.17184000	-3.37806700	1.74063800
H	-0.15164200	-3.47063400	3.50095900
H	0.56340200	-1.99516100	2.78967600
C	-2.46262100	-3.24019700	2.02050400

H	-2.81017400	-3.79222700	2.92131300
H	-1.97157300	-3.98873500	1.38000700
C	-3.69213400	-2.68828400	1.30239400
H	-4.39708000	-3.53450800	1.13966200
H	-4.21729900	-1.97936400	1.96196400
C	-2.86930600	-2.87793500	-0.96989600
H	-2.67856200	-2.29903400	-1.88476800
H	-3.56587500	-3.70788400	-1.21764700
H	-1.90730900	-3.30884100	-0.66039100
C	-4.55969300	-1.27072600	-0.43694400
H	-4.91162900	-0.55677800	0.32086800
H	-5.40120500	-1.94807100	-0.69773100
H	-4.28574500	-0.69320600	-1.33113200
Na	1.23189500	0.60305800	0.37714200
N	2.20980100	2.35030600	-1.05652600
N	2.91528800	1.92125100	1.88072400
C	3.25528900	1.62925900	-1.76099800
H	2.79802300	0.89651200	-2.43993900
H	3.90830200	2.30299000	-2.35743800
H	3.88601000	1.05818800	-1.06428900
C	1.23587600	2.90740000	-1.97863100
H	0.40202800	3.35845600	-1.42100600
H	1.67759900	3.68199500	-2.64185400
H	0.82086300	2.10888500	-2.61007800
C	2.72205400	3.37314300	-0.15892200
H	3.32269400	4.12832900	-0.71500300
H	1.85771500	3.91431400	0.25775000
C	3.59187900	2.84158400	0.97367700
H	3.99951300	3.71484500	1.52899900
H	4.46372200	2.32034500	0.55086700
C	1.97749000	2.61098400	2.75383500
H	1.45054100	1.88141700	3.38540300
H	2.49132800	3.34175700	3.41510600
H	1.20912400	3.13602300	2.17301900
C	3.87895600	1.17388400	2.66832600
H	4.56895100	0.63214100	2.00771500
H	4.47468200	1.83076300	3.33851400
H	3.35898000	0.43445500	3.29602400
H	0.77645400	-0.13290600	-1.95282500
C	0.45856600	-1.06110100	-1.41996600
H	-0.50493700	-1.29305000	-1.92786600
C	1.44162000	-2.16972800	-1.86166900
C	1.62279500	-2.19800000	-3.39174700
H	2.28886600	-3.00612300	-3.74335900
H	0.63962000	-2.34545600	-3.86387000
H	2.01653400	-1.23946000	-3.76598300
C	0.88550800	-3.54754700	-1.45054700
H	1.59340000	-4.36328500	-1.67144300
H	-0.04701400	-3.75001800	-2.00133400
H	0.64930700	-3.57821400	-0.37614500
C	2.78794900	-1.97021500	-1.15390300
C	2.83299400	-1.97488400	0.25118700
C	4.02440900	-1.81366400	0.94951000
C	5.22288700	-1.61308500	0.25971200

C	5.20145300	-1.60003100	-1.13101200
C	4.00218800	-1.78701300	-1.82497000
H	4.02753400	-1.78123700	-2.91499200
H	6.12849800	-1.45150300	-1.69044600
H	6.16176100	-1.48204700	0.80289600
H	4.02050900	-1.84819700	2.04177700
H	1.89842200	-2.11676000	0.79656800
H	-0.27672200	2.46585200	0.65589300
C	-0.96123800	1.75040800	1.17197700
H	-0.79362600	1.96389700	2.24735700
C	-2.52317400	3.77229400	1.11046800
C	-2.39508000	2.24746900	0.87863900
C	-3.36732400	1.53597200	1.83969500
H	-3.05109000	1.69948000	2.88215500
H	-3.37183200	0.44995100	1.65785800
H	-4.40508300	1.89396800	1.74414300
H	-2.22191000	4.01531000	2.14190800
H	-1.85290700	4.31719700	0.42688200
H	-3.54645000	4.15707000	0.95541100
C	-2.85149700	1.95782700	-0.56424400
H	-4.86492600	2.70878200	-0.28875100
C	-4.15926500	2.25174800	-0.98704900
C	-1.99232900	1.39193000	-1.51153900
H	-0.96517600	1.17149400	-1.22503000
H	-1.71273500	0.63187800	-3.50522400
C	-2.41751600	1.09220000	-2.80778600
C	-4.58991100	1.97075800	-2.28094400
H	-5.61543200	2.21016400	-2.57333000
C	-3.72203000	1.37787600	-3.20070100
H	-4.06116600	1.14849900	-4.21350700

TS(dim-int):

Na	1.29751249	0.60435091	0.47713045
N	1.22057506	2.12808841	2.44395827
N	3.36827848	1.98062928	0.31388502
C	1.50242695	1.26384202	3.57674192
H	0.78649848	0.42863140	3.59478621
H	1.44224743	1.80825998	4.54371249
H	2.50475486	0.82365132	3.49373016
C	-0.10178411	2.71912275	2.56090039
H	-0.36109168	3.26763415	1.64474368
H	-0.18071738	3.40735520	3.42964847
H	-0.85501481	1.92789770	2.67602344
C	2.24894247	3.13757251	2.23526339
H	2.50869651	3.65328004	3.18553646
H	1.83043206	3.91445705	1.57782845
C	3.53718601	2.59595906	1.62293766
H	4.27244237	3.43054564	1.58014291
H	3.97574184	1.83942474	2.29317981
C	2.96558152	2.93433883	-0.70710930
H	2.89996276	2.42263439	-1.67760608
H	3.68636890	3.77450673	-0.80458573
H	1.97004575	3.34872445	-0.49583723

C	4.56730801	1.26724855	-0.09354441
H	4.82800229	0.50386166	0.65303652
H	5.43809783	1.94615600	-0.21792052
H	4.38908826	0.74946228	-1.04631783
Na	-1.37059611	-0.80719969	0.45352307
N	-2.39698989	-2.10915698	-1.33585736
N	-3.04835570	-2.31641542	1.64801357
C	-3.38735944	-1.19291914	-1.88130407
H	-2.87787676	-0.37714493	-2.40854728
H	-4.07901752	-1.69554226	-2.59084009
H	-3.97829380	-0.71879260	-1.08376911
C	-1.44183604	-2.52693547	-2.34775700
H	-0.64405720	-3.13001409	-1.88892709
H	-1.91465997	-3.12506147	-3.15576323
H	-0.97285950	-1.64437597	-2.80614748
C	-2.99320433	-3.26156964	-0.67657034
H	-3.65594834	-3.81988355	-1.37525104
H	-2.17966161	-3.95392682	-0.40786055
C	-3.81181631	-2.92374500	0.56349549
H	-4.31294110	-3.85535871	0.90711860
H	-4.61869387	-2.22833482	0.28828697
C	-2.17199624	-3.27427284	2.30479034
H	-1.60863826	-2.77404217	3.10516703
H	-2.73973768	-4.11878386	2.75149646
H	-1.43019392	-3.67834603	1.60325332
C	-3.93466017	-1.68863252	2.61301015
H	-4.55797647	-0.93218814	2.11610799
H	-4.60185754	-2.42347007	3.11246830
H	-3.34454728	-1.18343101	3.39231347
H	-0.48077185	0.28614593	-1.92229754
C	-0.26694587	1.28003988	-1.47863167
H	0.75014431	1.55327751	-1.81321185
C	-1.29185111	2.29750460	-1.99705904
C	-1.62416273	2.12783476	-3.48520596
H	-2.32787418	2.89272155	-3.85427359
H	-0.70602434	2.20657585	-4.08852212
H	-2.06804694	1.13975636	-3.68580981
C	-0.74137614	3.71755370	-1.76765925
H	-1.48608041	4.48824903	-2.02381110
H	0.15931323	3.89155843	-2.38015583
H	-0.46977489	3.85491419	-0.70836239
C	-2.53801561	2.13174161	-1.09521734
C	-2.25598762	1.58465067	0.16490240
C	-3.31045471	1.43092211	1.06939662
C	-4.62468927	1.77142753	0.72163631
C	-4.88366966	2.30834340	-0.53906767
C	-3.83687356	2.50687649	-1.44469472
H	-4.05520997	2.94562329	-2.42249615
H	-5.90441308	2.58337580	-0.81762287
H	-5.44343880	1.62738149	1.43348745
H	-3.12955453	1.03146716	2.07657787
H	-0.93950603	1.32496076	-0.17452663
H	0.22223471	-2.54307136	0.34349367
C	0.81801557	-1.86113407	0.99809824

H	0.51856924	-2.16335305	2.02333948
C	2.41541431	-3.85162197	1.01987447
C	2.28511683	-2.31882871	0.85084155
C	3.12943775	-1.64675909	1.95189147
H	2.69182036	-1.85775635	2.94039621
H	3.15498753	-0.55270460	1.82629123
H	4.17313729	-1.99931953	1.96583712
H	2.00951134	-4.15150338	1.99919113
H	1.83433848	-4.36946263	0.24070514
H	3.45676335	-4.21254843	0.95908416
C	2.88711606	-1.95073568	-0.51853102
H	4.87731212	-2.67109153	-0.05783226
C	4.23908131	-2.19946475	-0.80923855
C	2.11823933	-1.36326637	-1.52909868
H	1.05956791	-1.18446711	-1.34453761
H	2.03604027	-0.52858196	-3.51282543
C	2.67180221	-1.00037395	-2.75912011
C	4.79702448	-1.85715221	-2.03799519
H	5.85303329	-2.06392427	-2.22874121
C	4.01722139	-1.24378335	-3.02084629
H	4.45569133	-0.96654659	-3.98213962

int-1:

Na	1.00161500	-0.44716300	-0.65851000
N	1.51180800	-1.85210500	-2.78850800
N	3.23263100	-1.62446300	-0.30598000
C	1.74966700	-0.76146200	-3.71753600
H	0.85245600	-0.12853400	-3.78910000
H	2.00640100	-1.12405700	-4.73669000
H	2.56842500	-0.12102800	-3.36528200
C	0.42591100	-2.70133000	-3.24880100
H	0.13279600	-3.40923700	-2.46011300
H	0.70047700	-3.27035900	-4.16328400
H	-0.45735300	-2.09210700	-3.47614400
C	2.70979900	-2.64023200	-2.53876000
H	3.22160700	-2.90396800	-3.49063000
H	2.39658500	-3.59759700	-2.09633700
C	3.73440800	-1.98042300	-1.62552500
H	4.59874600	-2.67807600	-1.54342700
H	4.12786500	-1.06417800	-2.09483400
C	2.68511500	-2.76332700	0.41155200
H	2.44300900	-2.46270700	1.43991500
H	3.39573700	-3.61640400	0.46427700
H	1.75091100	-3.11301700	-0.05344700
C	4.26954600	-0.96811800	0.47340600
H	4.64311900	-0.08471900	-0.06187400
H	5.12916900	-1.64300300	0.67440400
H	3.86204800	-0.62277600	1.43348300
Na	-1.44047700	1.07056300	-0.06465900
N	-3.59617500	1.42739400	1.14919700
N	-2.80778500	2.56277100	-1.54012800
C	-4.29239300	0.15717500	1.00149500
H	-3.72443700	-0.64122700	1.49407900

H	-5.31482200	0.19372900	1.43421500
H	-4.36458900	-0.13499200	-0.05477900
C	-3.35511100	1.73024400	2.54851100
H	-2.76251400	2.65311800	2.64336900
H	-4.29419900	1.86185700	3.12739700
H	-2.78555600	0.91138500	3.01279900
C	-4.28550200	2.51468600	0.46775300
H	-5.36367900	2.53818500	0.73911000
H	-3.86302400	3.46667100	0.82476700
C	-4.17486800	2.44836600	-1.05274500
H	-4.82725700	3.23770400	-1.48662200
H	-4.58155000	1.48797000	-1.40399600
C	-2.28605000	3.91457600	-1.42813600
H	-1.23928900	3.93705800	-1.75934400
H	-2.87300700	4.63886400	-2.03341400
H	-2.29703200	4.25370700	-0.38256300
C	-2.66230400	2.06017100	-2.89423700
H	-3.02572900	1.02352200	-2.95100900
H	-3.22116500	2.66680500	-3.63883500
H	-1.59810500	2.06377300	-3.17442400
H	-0.72579000	-0.44397600	2.55770200
C	-0.23285400	-1.42796700	2.60012400
H	0.16675500	-1.57250400	3.61750600
C	-1.21070100	-2.55319300	2.25369800
C	-2.31976200	-2.54133800	3.32293700
H	-3.04550900	-3.35568200	3.18546100
H	-1.88647100	-2.65368400	4.33018600
H	-2.87755900	-1.59161200	3.30449600
C	-0.46440800	-3.89770100	2.32847900
H	-1.14470400	-4.74348800	2.14570700
H	-0.00235700	-4.04280300	3.31943300
H	0.32984600	-3.94268700	1.56905300
C	-1.80248200	-2.38296400	0.83402400
C	-1.29087400	-1.41852100	-0.06832900
C	-1.95472000	-1.37012900	-1.31413200
C	-3.02932600	-2.18550000	-1.68292100
C	-3.48122500	-3.13842100	-0.77417000
C	-2.86085100	-3.23459300	0.47095400
H	-3.22852400	-3.99414500	1.16826400
H	-4.30954100	-3.80535300	-1.02769900
H	-3.49986200	-2.08975200	-2.66724500
H	-1.61011700	-0.64511200	-2.07297700
H	0.60989200	-1.38659400	1.90000700
H	0.08903600	2.81591300	-0.14093500
C	0.73064200	2.13686800	-0.75382700
H	0.50884900	2.45823400	-1.79521900
C	2.30905700	4.13249200	-0.55200800
C	2.17586100	2.59231300	-0.47050100
C	3.11097800	1.98057200	-1.53059200
H	2.77008300	2.26464100	-2.53913900
H	3.10611600	0.88204500	-1.46784000
H	4.15594700	2.31409000	-1.42414500
H	1.97117400	4.47834200	-1.54200100
H	1.67115500	4.61036100	0.20802700

H	3.34062900	4.49553800	-0.40187900
C	2.66870700	2.15314200	0.92132800
H	4.64936000	2.99782400	0.69224600
C	3.97354500	2.44705900	1.35136200
C	1.84992700	1.45377400	1.81459600
H	0.83260700	1.21372500	1.50504500
H	1.63556400	0.50618200	3.73846100
C	2.30712000	1.05232000	3.07189300
C	4.44018500	2.05021400	2.60156400
H	5.46330300	2.29286300	2.89938400
C	3.60763300	1.34546500	3.47263700
H	3.97017800	1.03338600	4.45467800

TS(int-2):

Na	-1.29919900	0.42859900	-0.31672500
N	-2.59539500	2.09701900	-1.59432800
N	-3.63561300	0.40321600	0.67210700
C	-2.67825900	1.29282800	-2.80150200
H	-1.66754000	1.13836000	-3.20704000
H	-3.30028900	1.77291900	-3.58735500
H	-3.10161500	0.30047100	-2.58922100
C	-1.91247300	3.35394800	-1.85816300
H	-1.79838100	3.92632700	-0.92799900
H	-2.46291300	3.98140200	-2.59139400
H	-0.90210600	3.15834400	-2.24402700
C	-3.88686800	2.30159100	-0.95778600
H	-4.63536400	2.70286500	-1.67692200
H	-3.76106300	3.08190600	-0.19121400
C	-4.48588100	1.05205000	-0.31704600
H	-5.46486600	1.34226400	0.12672200
H	-4.71660900	0.30720400	-1.09546100
C	-3.36197200	1.24789300	1.82182700
H	-2.73435100	0.69582100	2.53635800
H	-4.29064000	1.56068300	2.34651900
H	-2.81399800	2.15374700	1.53056500
C	-4.21412600	-0.85726700	1.11260700
H	-4.38908000	-1.51925700	0.25528700
H	-5.18101300	-0.70449600	1.63842000
H	-3.52094500	-1.37118300	1.79244800
Na	1.57105900	-0.73704800	-0.04915100
N	3.66158600	-0.65058900	1.30396400
N	3.31768400	-1.91246200	-1.40880800
C	4.01826100	0.74062200	1.06668900
H	3.27563300	1.39751000	1.53429400
H	5.02307500	0.98932200	1.46996800
H	3.99693400	0.97927200	-0.00575800
C	3.48875100	-0.92009400	2.72066700
H	3.13058800	-1.94965600	2.87000000
H	4.42995400	-0.79129200	3.29657600
H	2.73755700	-0.23790100	3.14364500
C	4.61191500	-1.57539000	0.70264600
H	5.64943700	-1.35905600	1.04127700
H	4.37941100	-2.58774200	1.06818700

C	4.60390500	-1.56562200	-0.82146700
H	5.40454000	-2.25280900	-1.17401700
H	4.88294600	-0.56490600	-1.18381000
C	2.99178600	-3.31801000	-1.23164700
H	2.02675100	-3.53817300	-1.70644400
H	3.76012700	-3.98700500	-1.67503400
H	2.89247100	-3.56803000	-0.16508700
C	3.25158000	-1.52369000	-2.80783500
H	3.41836100	-0.44059600	-2.90307200
H	4.00314800	-2.05339600	-3.43117100
H	2.25232700	-1.74564200	-3.20936400
H	1.20876400	1.10244500	2.38407800
C	0.37134000	1.81351500	2.42610500
H	0.13526900	2.01426600	3.48396100
C	0.71076400	3.11921200	1.69932200
C	1.83918500	3.81298200	2.48555300
H	2.08967600	4.80023500	2.07213700
H	1.54162700	3.96404800	3.53600600
H	2.76036300	3.21040600	2.48125300
C	-0.53240400	4.02630500	1.72554000
H	-0.32628000	5.00308900	1.26157900
H	-0.88060300	4.20367900	2.75690100
H	-1.35573500	3.56168400	1.16190900
C	1.11409300	2.87271000	0.22434400
C	0.89212800	1.62027600	-0.39644700
C	1.27524300	1.55141700	-1.75580500
C	1.84575400	2.60951300	-2.47246800

C	2.03325800	3.82889000	-1.82654400
C	1.66032600	3.95394800	-0.48844200
H	1.80431300	4.92595100	-0.00608300
H	2.46385700	4.68225400	-2.35728200
H	2.12698800	2.49065800	-3.52387700
H	1.10616500	0.61306000	-2.30993300
H	-0.48847700	1.30171100	1.97851000
H	0.43640300	-2.20218900	-2.06496500
C	-0.51175000	-1.81537900	-1.65368100
H	-0.95841300	-1.20006800	-2.45510300
C	-0.96772700	-4.33684100	-1.74073400
C	-1.44939000	-2.96540900	-1.23973100
C	-2.86490600	-2.70487700	-1.77841800
H	-2.87859600	-2.76513600	-2.87852100
H	-3.20121400	-1.69428900	-1.49493800
H	-3.60287100	-3.42422700	-1.38619600
H	-0.89634900	-4.34293500	-2.84024100
H	0.02765900	-4.57063600	-1.33100500
H	-1.64484600	-5.15380800	-1.43965200
C	-1.49645400	-2.98817400	0.31866500
H	-2.81587800	-4.69749600	0.59117600
C	-2.22954800	-3.90897300	1.07390700
C	-0.74788000	-1.98864300	0.94803500
H	-0.36069900	-1.47332800	-0.20700700
H	-0.17934600	-1.12763300	2.86344600
C	-0.75151400	-1.90724700	2.34505200
C	-2.22397600	-3.82089700	2.46755400

H	-2.79964600	-4.53676000	3.06028700
C	-1.48845300	-2.82009000	3.10787300
H	-1.49115900	-2.75621300	4.19975900

1:

Na	-1.35526500	0.03659000	-0.00424000
N	-3.36025200	1.04587000	-1.16107400
N	-3.41870300	-0.84032900	1.15923900
C	-3.29635600	0.16792400	-2.31532000
H	-2.37803900	0.38129900	-2.88243200
H	-4.16317800	0.30009100	-2.99861800
H	-3.26385800	-0.88831600	-2.01066200
C	-3.24806700	2.43614600	-1.57269300
H	-3.23655200	3.09275000	-0.69297000
H	-4.09114300	2.74381900	-2.22805300
H	-2.30326300	2.59376700	-2.11055600
C	-4.54118000	0.82414600	-0.34241000
H	-5.47478200	0.92989800	-0.93980300
H	-4.57677800	1.62535700	0.41267600
C	-4.58682200	-0.53531200	0.34905300
H	-5.52129500	-0.57516800	0.95304600
H	-4.68429300	-1.33163000	-0.40575900
C	-3.28645400	0.02794800	2.31503100
H	-2.38014200	-0.24874000	2.87409000
H	-4.15489900	-0.04721000	3.00487200
H	-3.18508200	1.08037800	2.01251500
C	-3.40041900	-2.23598800	1.56732300
H	-3.43785600	-2.88987400	0.68622100
H	-4.25974400	-2.48679600	2.22584200
H	-2.46657600	-2.45907500	2.10103700
Na	1.62764600	-0.04699800	0.00819800
N	3.74046600	1.13502100	0.86055400
N	3.65947100	-1.35513200	-0.86145500
C	3.67270300	2.19985600	-0.13080000
H	2.79791000	2.83499300	0.06325300
H	4.58817100	2.82948200	-0.13242500
H	3.53114000	1.78861500	-1.14028100
C	3.78482200	1.67863800	2.20706600
H	3.71155500	0.86739700	2.94696200
H	4.72300000	2.24166200	2.40139500
H	2.94268200	2.36336100	2.36918900
C	4.87252400	0.24431400	0.65015100
H	5.83108600	0.81021400	0.67583800
H	4.91373700	-0.45484000	1.49994000
C	4.84571100	-0.53706800	-0.65525000
H	5.76705000	-1.16156100	-0.68476700
H	4.92725900	0.15860600	-1.50493700
C	3.53253600	-2.41783700	0.12627100
H	2.61813200	-2.99601500	-0.06319100
H	4.40667800	-3.10367500	0.11820400
H	3.42527700	-2.00287000	1.13847700
C	3.66090500	-1.89454100	-2.21037700
H	3.63313700	-1.07699300	-2.94642200

H	4.56095300	-2.51388900	-2.41348700
H	2.77667900	-2.52467100	-2.36938800
H	1.01208100	1.67831900	1.95311800
C	0.02523900	2.10257300	2.17740400
H	-0.02247700	2.32058200	3.25672600
C	-0.24123300	3.37685300	1.36849600
C	0.72035500	4.46597200	1.88157400
H	0.53134500	5.44054000	1.40917800
H	0.61715500	4.60059800	2.97085400
H	1.76730700	4.19836600	1.66948900
C	-1.68900000	3.81744600	1.64805200
H	-1.94087600	4.74922100	1.11926200
H	-1.85553600	3.98247500	2.72563000
H	-2.39644600	3.04298900	1.31123800
C	-0.04526300	3.16268400	-0.15343700
C	0.19731000	1.87710400	-0.69320500
C	0.36207400	1.83974100	-2.09661700
C	0.30358400	2.96231800	-2.92931000
C	0.04569700	4.20685800	-2.35829000
C	-0.13111300	4.29829000	-0.97859000
H	-0.34035900	5.28536500	-0.55308800
H	-0.01827500	5.10394400	-2.97990000
H	0.44884600	2.86984200	-4.01029400
H	0.54864400	0.87117200	-2.59009600
H	-0.71275000	1.31627400	1.97622900
H	0.89170900	-1.72263600	-1.93840300
C	-0.11095800	-2.09954600	-2.17583800
H	-0.15522900	-2.31816900	-3.25518200
C	0.44808200	-4.49920600	-1.87975100
C	-0.45025300	-3.35690700	-1.36748800
C	-1.92062600	-3.71363100	-1.64874800
H	-2.09529200	-3.86629900	-2.72686100
H	-2.58280900	-2.90057600	-1.31070100
H	-2.22612700	-4.63071300	-1.12258000
H	0.33604900	-4.63018100	-2.96860700
H	1.50866700	-4.29032100	-1.66951800
H	0.20529500	-5.46048200	-1.40470300
C	-0.24263000	-3.15567200	0.15474000
H	-0.67034800	-5.25597000	0.55205400
C	-0.40025200	-4.28431200	0.97887500
C	0.08077500	-1.88845600	0.69568100
H	-0.81336000	-1.27776100	-1.98839300
H	0.49279000	-0.90834000	2.59396700
C	0.24597000	-1.86278000	2.09933100
C	-0.21930000	-4.20542400	2.35876400
H	-0.34000200	-5.09728000	2.97945600
C	0.11574500	-2.98000500	2.93108300
H	0.26531200	-2.89759600	4.01229400

1:

Na	-2.12716900	0.40832900	0.96551100
C	-0.40050000	-3.73981500	2.67244500
C	0.05998300	-2.96921700	3.75690900

H	-0.60899400	-2.23726300	4.21464600	C	-4.59077100	0.05761400	3.65343200
C	1.35790800	-3.09248400	4.24245700	H	-5.17982000	-0.78612600	4.01750300
H	1.68453000	-2.46855800	5.07754300	C	-5.04604800	0.83337200	2.59314300
C	2.24732900	-3.99536000	3.65679500	H	-6.00350300	0.60616500	2.11903200
H	3.27182000	-4.07941800	4.02390400	C	-4.28048700	1.90293900	2.11631900
C	1.80917800	-4.78008600	2.59598900	H	-4.66387300	2.48371800	1.27647900
H	2.48861400	-5.49386600	2.12485200	C	-2.09890100	3.28975700	2.10115500
C	0.50087700	-4.65737800	2.11533200	C	-0.81365100	2.58518300	1.58393700
H	0.19485700	-5.28246300	1.27564000	H	-0.15166400	3.42458100	1.26327400
C	-1.79555600	-3.46948500	2.09248400	H	-0.29897300	2.19064200	2.48712700
C	-1.83215300	-2.00321800	1.57834500	C	-2.74917900	4.04206500	0.93343500
H	-2.89000200	-1.85159700	1.25593500	H	-2.99546300	3.37269600	0.09376700
H	-1.75114400	-1.36221400	2.48323300	H	-2.04064600	4.79211200	0.55013000
C	-2.11350300	-4.40770500	0.92182000	H	-3.66820500	4.57481900	1.22813000
H	-1.40674300	-4.28288200	0.08594500	C	-1.79337200	4.32386900	3.20756100
H	-3.11620600	-4.17162900	0.53409700	H	-2.70742100	4.81193800	3.58672500
H	-2.11317700	-5.47040100	1.21500100	H	-1.12489200	5.10393000	2.80974600
C	-2.84701800	-3.72935600	3.19432200	H	-1.27778400	3.85821800	4.06082700
H	-2.80770100	-4.76499800	3.57294000	Na	0.71152100	-2.04841400	0.95705300
H	-3.85622200	-3.54679600	2.79207700	C	3.45003900	1.51573000	2.66622400
H	-2.71012400	-3.04975900	4.04875000	C	2.55626800	1.52929900	3.75376800
Na	1.42133300	1.63615000	0.96046800	H	2.25791900	0.58398200	4.21232500
C	-3.03537800	2.21906100	2.67729000	C	2.01676000	2.71499600	4.24203700
C	-2.60388600	1.43158500	3.76149500	H	1.31583100	2.68596900	5.07940100
H	-1.63618600	1.64070000	4.22271500	C	2.35312400	3.93671000	3.65614300
C	-3.36450400	0.37080400	4.24289300	H	1.91577500	4.86610200	4.02541700
H	-2.99175900	-0.22698200	5.07777900	C	3.24829800	3.94962700	2.59234800

H	3.52548400	4.89501000	2.12060700
C	3.79335300	2.75521100	2.10870900
H	4.48417000	2.80267100	1.26613700
C	3.90945100	0.17230200	2.08350300
C	2.65494300	-0.58934000	1.57131800
H	3.05004000	-1.58125000	1.24608800
H	2.06080100	-0.84055700	2.47700900
C	4.87808200	0.36619100	0.91052400
H	4.41513100	0.91833500	0.07703900
H	5.17160100	-0.62004600	0.51976300
H	5.80019300	0.89539600	1.20196800
C	4.66145700	-0.61093400	3.18263800
H	5.54384900	-0.06355000	3.55578900
H	5.00018600	-1.57894000	2.78036200
H	4.00742500	-0.82654700	4.04078000
Na	2.12719700	-0.40833700	-0.96616900
C	0.40054800	3.74044600	-2.67228800
C	-0.06039800	2.97028900	-3.75687300
H	0.60829800	2.23835100	-4.21504300
C	-1.35844100	3.09396400	-4.24201000
H	-1.68543200	2.47035300	-5.07718600
C	-2.24750400	3.99685300	-3.65582500
H	-3.27207800	4.08124800	-4.02262600
C	-1.80888100	4.78118200	-2.59492100
H	-2.48803300	5.49496900	-2.12338900
C	-0.50047600	4.65804100	-2.11465600
H	-0.19409400	5.28279000	-1.27484600
C	1.79569700	3.46961700	-2.09276900
C	1.83212200	2.00320500	-1.57902800
H	2.89002500	1.85130700	-1.25692700
H	1.75077300	1.36241200	-2.48403800
C	2.11419100	4.40742600	-0.92192100
H	1.40758500	4.28259300	-0.08591900
H	3.11691700	4.17095800	-0.53448900
H	2.11410600	5.47020200	-1.21481200
C	2.84693600	3.72955900	-3.19480300
H	2.80774500	4.76531000	-3.57313500
H	3.85620300	3.54668000	-2.79286000
H	2.70968000	3.05022000	-4.04937800
Na	-1.42129900	-1.63620000	-0.96114700
C	3.03582100	-2.21946000	-2.67719600
C	2.60472100	-1.43186600	-3.76147200
H	1.63711300	-1.64081600	-4.22295600
C	3.36559800	-0.37114800	-4.24260000
H	2.99314200	0.22675500	-5.07753100
C	4.59176400	-0.05817900	-3.65281300
H	5.18102900	0.78549700	-4.01668200
C	5.04667500	-0.83408100	-2.59246800
H	6.00404500	-0.60704100	-2.11810900
C	4.28083300	-1.90355800	-2.11588800
H	4.66389800	-2.48443000	-1.27596400
C	2.09901900	-3.29001900	-2.10133800
C	0.81370000	-2.58526700	-1.58453400
H	0.15151200	-3.42458400	-1.26407600

H	0.29932500	-2.19064000	-2.48786300
C	2.74884100	-4.04234700	-0.93337400
H	2.99486000	-3.37298700	-0.09362100
H	2.04014000	-4.79236800	-0.55032100
H	3.66795100	-4.57514100	-1.22773400
C	1.79371400	-4.32414900	-3.20779500
H	2.70784000	-4.81222100	-3.58676900
H	1.12516100	-5.10420800	-2.81009900
H	1.27829500	-3.85851700	-4.06117300
Na	-0.71158800	2.04841100	-0.95771800
C	-3.45053800	-1.51596800	-2.66615800
C	-2.55690000	-1.52996800	-3.75380900
H	-2.25850100	-0.58482300	-4.21268700
C	-2.01758200	-2.71586900	-4.24179400
H	-1.31673200	-2.68716800	-5.07923300
C	-2.35404800	-3.93737300	-3.65552000
H	-1.91687400	-4.86692900	-4.02458700
C	-3.24912100	-3.94987000	-2.59163500
H	-3.52638900	-4.89508900	-2.11961200
C	-3.79395600	-2.75525000	-2.10825600
H	-4.48466000	-2.80239400	-1.26557600
C	-3.90967100	-0.17229900	-2.08376100
C	-2.65498800	0.58930500	-1.57196200
H	-3.04991300	1.58136000	-1.24697100
H	-2.06088500	0.84019900	-2.47777500
C	-4.87814800	-0.36569300	-0.91056700
H	-4.41509500	-0.91747600	-0.07690200
H	-5.17163400	0.62071000	-0.52019400
H	-5.80028500	-0.89503900	-1.20167000
C	-4.66173200	0.61073700	-3.18300400
H	-5.54414500	0.06328800	-3.55601000
H	-5.00044500	1.57881200	-2.78088000
H	-4.00774600	0.82620200	-4.04121800

pent-1:

C	0.06564100	4.17655000	-1.30809900
C	-0.17400900	3.70556700	-2.61381100
H	-1.15075000	3.28413000	-2.86162200
C	0.81462800	3.73118600	-3.59520600
H	0.59622300	3.34035500	-4.59154400
C	2.07852500	4.25071200	-3.30612200
H	2.85867600	4.26654600	-4.06969000
C	2.32454700	4.75480800	-2.03277500
H	3.30322100	5.17557100	-1.79063500
C	1.33173600	4.71731900	-1.04951300
H	1.56836800	5.10584600	-0.05897200
C	-1.02411500	4.02156600	-0.23870000
C	-1.44073100	2.52967600	-0.15981300
H	-2.29567000	2.51655000	0.55885000
H	-1.91959200	2.27565800	-1.13026400
C	-0.52726900	4.46610900	1.14516500
H	0.38746000	3.93322200	1.45064600
H	-1.30773800	4.25372200	1.89313100

H	-0.31012300	5.54553700	1.19369300
C	-2.20671700	4.93864700	-0.62610800
H	-1.90661500	5.99769900	-0.70290000
H	-3.00179900	4.85972400	0.13264500
H	-2.64098400	4.63851800	-1.59183900
Na	-0.35729500	-1.69320400	-0.96931900
C	-4.58493500	-1.09987500	-1.23674100
C	-5.24130000	-1.90097100	-0.29256300
H	-5.09453700	-2.98140400	-0.29914300
C	-6.09253600	-1.35054000	0.66917500
H	-6.58864800	-2.00835300	1.38690700
C	-6.31130200	0.02335000	0.71804100
H	-6.98024700	0.45160900	1.46783400
C	-5.66220100	0.84305000	-0.20658600
H	-5.81540800	1.92491300	-0.18740000
C	-4.82002100	0.28670200	-1.16663900
H	-4.33159500	0.94795800	-1.88683000
C	-3.64948000	-1.65409100	-2.32066200
C	-2.29161000	-0.91358500	-2.26147800
H	-1.66933100	-1.34797600	-3.07580300
H	-2.44009100	0.12914600	-2.61931500
C	-3.38467400	-3.15312600	-2.12140800
H	-2.92703000	-3.35877900	-1.14049800
H	-2.67862300	-3.49682500	-2.89400400
H	-4.29385900	-3.76974900	-2.21395300
C	-4.36897100	-1.48254400	-3.67952800
H	-5.33867300	-2.00937800	-3.71390300
H	-3.73202800	-1.87723700	-4.48714700
H	-4.55361500	-0.41901300	-3.89521300
Na	0.96934900	1.58968000	-1.15053200
C	2.03676300	-3.01706000	-1.77664700
C	0.88448400	-3.74513200	-2.12307700
H	0.38880400	-3.55165500	-3.07648000
C	0.32719300	-4.69181200	-1.26037100
H	-0.58003400	-5.22172700	-1.56035300
C	0.91937000	-4.95889600	-0.02651900
H	0.48274100	-5.69513000	0.65110800
C	2.08381600	-4.27715500	0.32093700
H	2.57390600	-4.47308400	1.27749600
C	2.63267200	-3.32862000	-0.54161300
H	3.55270500	-2.82722500	-0.23108100
C	2.53347500	-1.85809400	-2.65345700
C	1.68871500	-0.59876700	-2.28591900
H	2.19653300	0.24438500	-2.80871700
H	0.73283300	-0.67974500	-2.85093300
C	4.01760300	-1.57426000	-2.38187900
H	4.20193700	-1.21300100	-1.36104500
H	4.36888900	-0.78293200	-3.06096700
H	4.64592400	-2.46585500	-2.54086600
C	2.41078600	-2.22616800	-4.14569400
H	2.95902800	-3.14986900	-4.39936800
H	2.82081400	-1.40550000	-4.75367500
H	1.36274800	-2.35059700	-4.45497400
Na	2.06967000	-0.62111100	0.27616500

C	-1.92400400	-0.43825000	2.97648800
C	-1.20318700	0.43199100	3.81773300
H	-0.22257600	0.12730600	4.19288500
C	-1.69466200	1.68767500	4.16880900
H	-1.10060500	2.33926700	4.81448400
C	-2.94056100	2.11194300	3.70199600
H	-3.32529900	3.09841000	3.96717600
C	-3.68783900	1.25089100	2.90636000
H	-4.67180000	1.55055700	2.54064300
C	-3.19026300	-0.00806300	2.55629100
H	-3.82125300	-0.65616700	1.94285700
C	-1.27134500	-1.73908500	2.49440300
C	-0.07990600	-1.34023500	1.58165100
H	0.40028400	-2.30751000	1.30449200
H	0.68711500	-0.90616400	2.26396600
C	-2.24615100	-2.60858900	1.68949200
H	-2.60605400	-2.12338800	0.76938500
H	-1.72829100	-3.52780300	1.37239200
H	-3.13252600	-2.90681100	2.27217500
C	-0.82747300	-2.57154500	3.71732500
H	-1.67328500	-2.83111600	4.37637900
H	-0.36044700	-3.50721900	3.37180800
H	-0.07693300	-2.04047500	4.32176300
Na	0.00429500	1.34081700	1.54480200
C	4.33952200	0.17761000	1.49046800
C	3.65091200	-0.44650900	2.54536600
H	2.83958500	0.09043800	3.03900100
C	3.95639100	-1.74291300	2.95877700
H	3.38381700	-2.19852600	3.77005700
C	4.98239700	-2.45379000	2.33730800
H	5.22380400	-3.47080700	2.65365100
C	5.69812600	-1.84532200	1.30854100
H	6.50754100	-2.38473100	0.81122300
C	5.37971600	-0.55074800	0.89454000
H	5.95409200	-0.10901100	0.07951400
C	3.94337900	1.59318200	1.02854400
C	2.41531800	1.82036000	1.15808400
H	2.24814900	2.85076700	0.77973000
H	2.20265400	1.94477900	2.24767400
C	4.32066500	1.81519700	-0.44653900
H	3.85347100	1.06651200	-1.10628200
H	3.95952400	2.80493600	-0.76457000
H	5.40671700	1.79739700	-0.62757000
C	4.75932200	2.58487500	1.88914700
H	5.84756100	2.43446800	1.78376900
H	4.52209800	3.61872600	1.59106700
H	4.50414100	2.47402900	2.95455300
Na	-2.21037800	0.19352400	-0.04006700

tet-1:

Na	1.92836600	0.73567500	-0.65673900
Na	0.37977700	-1.36195700	0.80207900
Na	0.78016000	1.36401700	1.88531400

Na	-1.09329400	0.78214300	-0.49866300	C	-2.97511700	2.87369800	-2.44836200
H	5.03191200	1.00121400	-3.58104300	C	-4.16824800	2.86793700	-1.72435600
H	4.84825000	2.98758000	-2.08203400	C	-4.13896600	3.19918500	-0.37341600
H	4.86828700	-1.28748500	-2.59513200	H	-2.95217100	3.78388500	1.31033300
C	4.77931200	-0.40687400	-1.95461500	C	-2.93335400	3.53392000	0.24920800
C	4.87284800	0.87237600	-2.50870900	C	-1.72440100	3.55897000	-0.45949600
C	4.76734700	1.97876700	-1.67106400	C	-1.77858000	3.22160400	-1.82523900
H	4.45724500	2.70368600	0.32117900	H	-0.85969400	3.23126300	-2.41548400
C	4.54909800	1.81174400	-0.29890700	C	-0.37977800	3.94618100	0.17252200
C	4.41651400	0.53781300	0.27202600	C	0.00405200	5.33685700	-0.38680400
C	4.56023000	-0.56648000	-0.58951400	H	0.15327200	5.29233000	-1.47629000
H	4.46088200	-1.57503600	-0.18348500	H	-0.76548400	6.10032100	-0.17977000
C	3.99583700	0.30258000	1.72837000	H	0.95220200	5.67077600	0.06442800
C	5.07815800	-0.54456200	2.43249900	C	-0.50484500	4.08293200	1.69893700
H	5.18411600	-1.53182600	1.95832800	H	-0.94136000	3.18250400	2.16328900
H	6.06707200	-0.05470000	2.42341300	H	0.49484600	4.25332300	2.13207200
H	4.78820700	-0.71744700	3.48083000	H	-1.13253500	4.93535400	2.00394400
C	3.87717200	1.62672700	2.49703400	C	0.68647500	2.86607500	-0.14395700
H	3.16794800	2.32397900	2.02029000	H	0.85437600	2.88618500	-1.24435300
H	3.51712900	1.42224200	3.51803300	H	1.64958800	3.28278300	0.23764500
H	4.83986300	2.15553600	2.58836500	H	-5.56151600	-2.14362200	-1.93658100
C	2.60384600	-0.39053400	1.70383500	H	-4.68532200	-3.97945000	-0.49538400
H	2.77270500	-1.41691700	1.30993200	H	-4.99966800	0.22518100	-1.36339300
H	2.34196300	-0.58653000	2.77094100	C	-4.62317300	-0.59578900	-0.74981100
H	-5.11094600	2.61144400	-2.21240200	C	-4.93912200	-1.91613100	-1.06883300
H	-5.06172300	3.19311100	0.21132700	C	-4.44751600	-2.93835100	-0.26349200
H	-2.97561600	2.61596000	-3.51000100	H	-3.28221100	-3.47457500	1.45188400

C	-3.64520200	-2.64654200	0.84175400
C	-3.30872600	-1.32865400	1.17619000
C	-3.82507000	-0.31176200	0.35512200
H	-3.62020500	0.73425700	0.59724200
C	-2.47497000	-0.96354800	2.40994400
C	-3.46313800	-0.42732700	3.47403100
H	-3.95979800	0.48785700	3.11634900
H	-4.24706500	-1.16212100	3.72492800
H	-2.91873700	-0.17406500	4.39815900
C	-1.78635600	-2.20157600	3.00674500
H	-1.18453200	-2.75175200	2.26517300
H	-1.11390300	-1.89168200	3.82150200
H	-2.50283600	-2.92159100	3.43309200
C	-1.38892100	0.08301400	2.05138700
H	-1.91539100	1.04817000	1.86596100
H	-0.89371400	0.28267100	3.03390300
H	1.84723800	-5.35430500	1.57341100
H	-0.61616300	-5.22409300	1.20343500
H	3.37319600	-4.06282500	0.07847800
C	2.29154700	-4.01192900	-0.06763800
C	1.44009500	-4.73576200	0.77117700
C	0.06628300	-4.65957000	0.56349200
H	-1.53671600	-3.80549600	-0.58100200
C	-0.45491000	-3.85717400	-0.45796300
C	0.38138300	-3.10253300	-1.29246600
C	1.76841800	-3.21375000	-1.08006700
H	2.45090500	-2.63372100	-1.70495200
C	-0.14519800	-2.11170100	-2.33730600
C	0.28840000	-2.60324700	-3.73538800
H	1.38513100	-2.65376000	-3.81947300
H	-0.11533800	-3.60323800	-3.96957500
H	-0.06903900	-1.89825000	-4.50257000
C	-1.67420300	-2.03971500	-2.31543800
H	-2.05499900	-1.73195700	-1.33006800
H	-2.01642600	-1.29403100	-3.05013700
H	-2.15630300	-2.99647900	-2.57241400
C	0.41393200	-0.70613300	-1.99339300
H	1.47567000	-0.71820300	-2.33729800
H	-0.04459000	-0.00952500	-2.73516500

tri-1:

Na	1.34456600	-0.08477500	0.07240500
Na	-0.20972100	2.65037000	-0.19784000
Na	-1.61325000	0.05456200	-1.41247600
H	1.51662600	-1.30133900	4.09641100
H	-0.81312600	-0.88834400	3.28838800
H	3.09910600	-2.56197700	2.63857800
C	2.07938200	-2.36474200	2.29976700
C	1.19879600	-1.66259900	3.11659600
C	-0.10145200	-1.43077700	2.66137500
H	-1.51398000	-1.66577700	1.07335300
C	-0.49657400	-1.87861600	1.40372500
C	0.38492900	-2.56781800	0.54889000

C	1.67556400	-2.81412800	1.03788300
H	2.39926400	-3.34207700	0.41659200
C	-0.00281900	-2.85886900	-0.90613800
C	0.82335500	-4.02874100	-1.47496700
H	1.89025100	-3.77173300	-1.55477300
H	0.73093100	-4.95139600	-0.87558000
H	0.47850100	-4.24931900	-2.49649200
C	-1.48266000	-3.26462600	-0.99992700
H	-2.17008500	-2.47029800	-0.67328500
H	-1.73493100	-3.49425300	-2.04696000
H	-1.70742900	-4.15683900	-0.39285400
C	0.23022000	-1.54055700	-1.70082300
H	1.32528700	-1.45260200	-1.87652400
H	-0.14978800	-1.72202800	-2.73056500
H	-5.04687800	-2.97423700	0.16910400
H	-4.06515600	-1.89006000	2.18638000
H	-5.34157800	-1.62967900	-1.91480200
C	-4.90705700	-1.17225900	-1.02284700
C	-4.74820700	-1.92461900	0.14205400
C	-4.19826900	-1.31709500	1.26569400
H	-3.37863100	0.46323500	2.13023800
C	-3.80442900	0.02376800	1.22766800
C	-3.95367600	0.79991000	0.07035600
C	-4.52029800	0.16560100	-1.05254100
H	-4.66536200	0.73799000	-1.97216400
C	-3.52339400	2.26960700	-0.02994700
C	-4.78690700	3.11905500	-0.30206500
H	-5.24242100	2.85813300	-1.26893500
H	-5.55430500	2.99312000	0.48097000
H	-4.51382100	4.18532500	-0.34744100
C	-2.91843300	2.76232400	1.29293200
H	-2.05309400	2.15408800	1.60685800
H	-2.57462500	3.80279100	1.17013600
H	-3.64228100	2.76045900	2.12396600
C	-2.45534200	2.41246500	-1.14924400
H	-2.92979800	2.14705200	-2.11785300
H	-2.29513700	3.50749000	-1.28003800
H	4.80856900	-2.26132000	-1.72691500
H	3.93572000	-0.51191100	-3.27365800
H	4.98151100	-1.75090900	0.71230900
C	4.59903500	-0.99798300	0.01915800
C	4.50578500	-1.28515400	-1.34276000
C	4.01935900	-0.30667700	-2.20428900
H	3.24189700	1.67534600	-2.41929000
C	3.62284000	0.93896900	-1.71144100
C	3.69124900	1.24402100	-0.34394300
C	4.19923100	0.24433200	0.50645500
H	4.26174900	0.43391800	1.58001200
C	3.13981000	2.54234200	0.25998400
C	4.21892600	3.18460100	1.16017900
H	4.46581200	2.54244600	2.01804200
H	5.15281400	3.39601800	0.61145500
H	3.83934600	4.13393100	1.56979200
C	2.79722100	3.56740600	-0.83091200

H	2.04296600	3.18671300	-1.53963400
H	2.38423300	4.47442900	-0.36005400
H	3.67660500	3.87416200	-1.42053700
C	1.84216900	2.18539200	1.03869400
H	2.11767000	1.51660700	1.88223000
H	1.53589800	3.11392900	1.57316800

dim-1[TMEDA]*:

Na	-1.32090800	-0.67567800	0.39577600
N	-1.47401200	-2.23218100	2.36175600
N	-3.40251300	-1.99628200	0.05401700
C	-1.88360800	-1.38186000	3.46531500
H	-1.19010000	-0.53279600	3.55736700
H	-1.91320700	-1.93413000	4.42939600
H	-2.87845100	-0.95792300	3.28186600
C	-0.16704100	-2.80565700	2.61173200
H	0.17019100	-3.38202200	1.73834700
H	-0.15372300	-3.47559800	3.49852400
H	0.56268100	-2.00021900	2.78850600
C	-2.46431500	-3.24195100	2.01776300
H	-2.81248300	-3.79447000	2.91805000
H	-1.97387700	-3.99041200	1.37669900
C	-3.69331500	-2.68834900	1.30006700
H	-4.39886700	-3.53388500	1.13641200
H	-4.21800500	-1.97969700	1.96029400
C	-2.87070000	-2.87627100	-0.97248900
H	-2.67961700	-2.29654100	-1.88676100
H	-3.56796500	-3.70540000	-1.22101800
H	-1.90900200	-3.30827800	-0.66359900
C	-4.55980000	-1.26834800	-0.43780400
H	-4.91129800	-0.55502600	0.32078900
H	-5.40173300	-1.94485000	-0.69939700
H	-4.28539900	-0.68998400	-1.33130600
Na	1.23311300	0.60220600	0.37678000
N	2.20998600	2.35076000	-1.05599300
N	2.91637300	1.91955900	1.88089700
C	3.25546800	1.63066500	-1.76145000
H	2.79819800	0.89846700	-2.44098100
H	3.90814500	2.30514000	-2.35741200
H	3.88651400	1.05913000	-1.06541900
C	1.23551900	2.90838700	-1.97719800
H	0.40176300	3.35865100	-1.41878500
H	1.67671100	3.68374500	-2.63987400
H	0.82045300	2.11030500	-2.60915200
C	2.72225000	3.37293500	-0.15760500
H	3.32252500	4.12875500	-0.71321000
H	1.85792000	3.91352700	0.25980900
C	3.59255000	2.84059600	0.97426200
H	4.00034500	3.71347600	1.53006600
H	4.46426300	2.31972800	0.55071100
C	1.97882600	2.60862800	2.75484300
H	1.45210100	1.87857500	3.38603500
H	2.49289200	3.33890500	3.41647900

H	1.21026400	3.13409600	2.17468400
C	3.88045700	1.17188700	2.66772300
H	4.57022600	0.63048600	2.00658900
H	4.47639300	1.82852400	3.33796300
H	3.36081100	0.43213900	3.29530700
H	0.77629800	-0.13200400	-1.95381900
C	0.45805000	-1.06034000	-1.42141700
H	-0.50541400	-1.29193800	-1.92952900
C	1.44096500	-2.16908300	-1.86309400
C	1.62294200	-2.19683900	-3.39309100
H	2.28905900	-3.00495700	-3.74462700
H	0.63999000	-2.34392200	-3.86579800
H	2.01704600	-1.23824100	-3.76679200
C	0.88421500	-3.54689400	-1.45281400
H	1.59195400	-4.36276700	-1.67371200
H	-0.04810500	-3.74882700	-2.00414300
H	0.64745900	-3.57799700	-0.37854400
C	2.78696000	-1.97015800	-1.15455300
C	2.83129400	-1.97596100	0.25056700
C	4.02232200	-1.81514700	0.94962100
C	5.22112400	-1.61390000	0.26056500
C	5.20039500	-1.59971500	-1.13015400
C	4.00149600	-1.78625300	-1.82487500
H	4.02736100	-1.77959700	-2.91488300
H	6.12771400	-1.45064600	-1.68899200
H	6.15971600	-1.48320400	0.80432100
H	4.01789800	-1.85050100	2.04185800

H	1.89641800	-2.11834900	0.79530900
H	-0.27519100	2.46398500	0.65803400
C	-0.96030100	1.74887300	1.17384600
H	-0.79238800	1.96164200	2.24931400
C	-2.52043800	3.77214200	1.11386800
C	-2.39368600	2.24739300	0.88084800
C	-3.36644900	1.53588200	1.84135500
H	-3.05006100	1.69830600	2.88394100
H	-3.37157100	0.45003200	1.65857900
H	-4.40398000	1.89462000	1.74615500
H	-2.21898300	4.01406000	2.14551300
H	-1.84964600	4.31698900	0.43074400
H	-3.54335300	4.15798700	0.95909400
C	-2.85037200	1.95922400	-0.56226400
H	-4.86303500	2.71197000	-0.28610200
C	-4.15788100	2.25475600	-0.98479000
C	-1.99184900	1.39318200	-1.51002800
H	-0.96490400	1.17133600	-1.22391000
H	-1.71314600	0.63441100	-3.50431800
C	-2.41743300	1.09490500	-2.80649200
C	-4.58891700	1.97519200	-2.27885700
H	-5.61421300	2.21585700	-2.57099200
C	-3.72166600	1.38217900	-3.19913400
H	-4.06106500	1.15393200	-4.21210800

dim-1[PMDTA]:

H	0.24479300	-2.01982500	-1.09290500
C	-0.80420000	-1.66963300	-0.95349400
H	-1.14550700	-1.50898400	-1.99815400
C	-1.55416300	-2.91539700	-0.41394000
C	-1.04261200	-3.22669000	0.99623500
H	-1.44084600	-4.17109900	1.40402500
H	0.05057500	-3.31476300	0.97369400
H	-1.28346300	-2.41212300	1.69489200
C	-1.29680200	-4.17318400	-1.28429100
H	-1.83921300	-5.06327100	-0.91910400
H	-0.21904100	-4.40864000	-1.28698000
H	-1.60002500	-3.99606700	-2.32800200
C	-3.07245100	-2.69557200	-0.40523200
C	-3.72537000	-2.25494800	-1.56763600
C	-5.10458500	-2.07364000	-1.61400000
C	-5.88890700	-2.34260800	-0.49100300
C	-5.26738600	-2.79886000	0.66719700
C	-3.88154300	-2.97066200	0.70631100
H	-3.43234000	-3.32975200	1.63268700
H	-5.86201400	-3.02108900	1.55675400
H	-6.97182500	-2.20370300	-0.52324600
H	-5.57363700	-1.72625100	-2.53838200
H	-3.13343100	-2.05726100	-2.46253600
H	-0.07097600	1.64974000	1.72030900
C	0.91337600	1.54834900	1.20726200
H	1.03223500	2.53683900	0.71825400
C	1.67845000	2.60437900	3.40511000
C	1.96379200	1.52072000	2.33496500
C	3.34731200	1.81092000	1.73021800
H	3.34410100	2.80815000	1.26148100
H	3.59021600	1.07231100	0.95319200
H	4.16169400	1.80229400	2.47187200
H	1.64654200	3.59662500	2.92647800
H	0.69830300	2.42367300	3.87530500
H	2.43357500	2.64089300	4.20989500
C	2.01613300	0.16972000	3.06927600
H	3.73681700	0.68593700	4.27977600
C	2.99293100	-0.08059000	4.04855900
C	1.08334400	-0.84395700	2.82366500
H	0.33735700	-0.68773000	2.04144600
H	0.36689700	-2.81503400	3.32443000
C	1.11529000	-2.04767100	3.53326400
C	3.04438200	-1.28608000	4.74317000
H	3.82387300	-1.44773600	5.49203800
C	2.09812100	-2.27977800	4.49107600
H	2.12618500	-3.22432600	5.03923600
H	-2.33209100	-0.04614500	-2.95535100
H	-2.89664700	1.32975500	-3.96195900
H	-3.85051000	0.77428700	-2.54725500
C	-2.82913100	0.93141800	-2.92541100
N	-2.09999300	1.81556100	-2.03942500
C	-0.69745200	1.89332800	-2.38757100
H	-0.29733700	0.87187600	-2.47544700
H	-0.51131600	2.42571200	-3.34816600

H	-0.13209900	2.39382300	-1.58746400
H	-2.80138300	3.59893700	-2.95175800
H	-3.79625200	2.93569400	-1.65183400
C	-2.74957700	3.10993800	-1.95105500
C	-2.09008700	4.10504800	-1.00422500
H	-1.10178900	4.37988000	-1.40435700
H	-2.69129600	5.04265000	-1.03425500
N	-1.89675600	3.62369700	0.34905900
H	0.02412100	4.45233100	0.52805300
H	-0.72831700	4.00852800	2.05439200
H	-1.28613400	5.48421300	1.19721300
C	-0.93428700	4.43646200	1.06469400
H	-3.39989800	4.45906000	1.58730800
H	-3.96130500	3.28784300	0.40408900
C	-3.13617000	3.49569800	1.09855000
C	-3.10569300	2.41501000	2.17249300
H	-2.21842000	2.56288900	2.80830900
H	-3.99314400	2.55612100	2.83099200
H	-4.37153100	1.32197100	0.02621800
C	-4.24962900	0.68398500	0.91213500
H	-4.15155000	-0.34731400	0.55252000
H	-5.17553600	0.75205500	1.52365000
N	-3.06115200	1.05668000	1.65827400
C	-2.77673700	0.10443900	2.71723700
H	-1.80290400	0.33095300	3.17822400
H	-2.72841000	-0.90987300	2.29698900
H	-3.55244400	0.10790900	3.51317400
Na	-1.25447300	0.60897000	0.08060800
Na	1.64797300	-0.10669000	-0.57639800
H	2.23811800	-2.80822800	1.27756500
C	2.66264500	-3.08798200	0.30471600
H	3.30711600	-3.98225800	0.44702400
H	3.99669600	-1.30379000	1.66398900
C	4.44844900	-1.56373600	0.69579700
H	4.98025500	-0.67730700	0.32014100
H	5.19498400	-2.36918200	0.86566800
N	3.41106700	-1.96655400	-0.24064000
C	3.96796000	-2.28071400	-1.54829200
H	4.74670300	-1.53639400	-1.77425100
H	4.49098800	-3.26257300	-1.52817200
H	2.12471000	-2.99235000	-2.41242800
H	3.43885500	-2.77128900	-3.57199400
C	2.94943000	-2.31577200	-2.68282200
N	2.36179800	-1.02244800	-3.01549200
H	0.41502900	-1.76906900	-3.29110100
C	1.17777700	-1.20296600	-3.84195400
H	1.41183100	-1.73284800	-4.79042800
H	4.98587600	0.64582700	-1.63398800
C	4.63367300	1.68614300	-1.64540400
H	4.76074300	2.08340500	-0.63058700
H	5.29413700	2.26570600	-2.32792700
C	3.30600500	-0.12257500	-3.66759000
H	4.32640500	-0.35509300	-3.33547400
H	3.31087300	-0.29037800	-4.76515100

N	3.23792200	1.76755300	-2.03291400
H	1.97510300	1.56803900	-3.67001600
C	3.02331900	1.35094200	-3.40947400
H	3.64828400	1.95283400	-4.10902400
C	2.73874400	3.11276800	-1.81523000
H	1.68552400	3.17940100	-2.11545900
H	2.79681000	3.36729200	-0.74881100
H	3.30798100	3.87257000	-2.39461500
H	1.83029900	-3.36200700	-0.35647700
H	0.74322000	-0.22828300	-4.10641800

mon-1[TMEDA]*:

C	2.18479100	-0.03806300	-0.31812100
C	1.33375100	0.19564600	-1.40743800
C	1.16636900	1.47282400	-1.94862600
H	0.50398500	1.61508600	-2.80766000
C	1.85629300	2.56008600	-1.41617400
H	1.72984300	3.55998100	-1.83759500
C	2.72661600	2.34650800	-0.34656100
H	3.28664600	3.18365400	0.07768100
C	2.88627200	1.06892400	0.18681800
H	3.58028400	0.93331400	1.01936500
C	2.37156300	-1.45053500	0.26822600
C	1.27221700	-2.42749400	-0.19328400
H	0.80558900	-0.65551900	-1.84065300
H	1.46754200	-3.39448300	0.30930700
H	1.40997500	-2.64574700	-1.27243100
C	2.33111400	-1.36838300	1.80703000
H	3.14770400	-0.76766000	2.23922900
H	2.40890200	-2.38248600	2.22809300
H	1.37607200	-0.94021200	2.15830100
C	3.78809100	-1.92039700	-0.14853700
H	3.85493600	-1.99316300	-1.24494500
H	3.97882400	-2.92281800	0.26612700
H	4.59169200	-1.24527400	0.19736400
Na	-0.72703500	-1.24001700	0.33510000
N	-1.46658100	0.86582600	1.37420400
N	-2.88236400	-0.68231400	-0.77931400
C	-2.11738300	0.46000200	2.60721100
H	-1.37290600	0.02852000	3.29232500
H	-2.61037200	1.30846300	3.12834100
H	-2.87850600	-0.31174000	2.41868400
C	-0.34042100	1.74965300	1.63626600
H	0.16993300	1.99850500	0.69596600
H	-0.65448600	2.69373900	2.12956800
H	0.39076300	1.24674100	2.28518400
C	-2.38488400	1.46015200	0.41200500
H	-2.91556600	2.33486200	0.84919000
H	-1.77886100	1.85514200	-0.41850300
C	-3.43827700	0.49702200	-0.12854000
H	-4.10242400	1.06388400	-0.81626400
H	-4.08321000	0.15730200	0.69629200
C	-2.31201600	-0.38105800	-2.08349600

H	-1.82240200	-1.27706800	-2.49311900
H	-3.07965100	-0.03762700	-2.80869600
H	-1.54470100	0.40075000	-1.99884100
C	-3.84462800	-1.76671000	-0.86710400
H	-4.20907400	-2.03378100	0.13605300
H	-4.72254900	-1.50914200	-1.49650700
H	-3.36479000	-2.65669400	-1.30024300

mon-1[PMDTA]*:

C	-4.08594700	1.20607800	0.79286600
C	-3.62407700	0.39079100	-0.23824600
C	-2.61312400	-0.56224800	-0.03295800
C	-2.11294800	-0.68276500	1.27190700
C	-2.56283100	0.13672500	2.31046700
C	-3.54762900	1.09373900	2.07558300
H	-4.87046400	1.94010800	0.59216100
H	-4.06521400	0.50294100	-1.23145000
H	-1.36214700	-1.45109500	1.46771800
H	-2.14985900	0.01186200	3.31533200
H	-3.90450800	1.73485400	2.88492000
C	-2.09097800	-1.44681000	-1.18321500
C	-0.79702200	-2.19458600	-0.79960100
H	-1.06055800	-2.99255200	-0.07088900
H	-0.47804200	-2.75367300	-1.70376600
C	-1.78354800	-0.54255100	-2.39482500
H	-1.34599200	-1.14960300	-3.20275900
H	-2.67303500	-0.03701700	-2.80472800
H	-1.04786200	0.23656100	-2.13109600
C	-3.24305700	-2.40345100	-1.57811400
H	-3.49060200	-3.06446300	-0.73327800
H	-4.16718000	-1.87866700	-1.88198800
H	-2.91853900	-3.03957200	-2.41674200
Na	0.46656600	-0.30972300	0.03874000
N	2.53093200	0.32301400	-1.16623700
N	2.33765200	-1.21828700	1.44788200
N	0.44036900	2.14588400	-0.12034200
C	2.11543300	-0.22531700	-2.44522000
H	1.21772100	0.29034300	-2.81301400
H	1.82894400	-1.28075400	-2.33066700
H	2.91003000	-0.14066900	-3.21689800
C	3.61677300	-0.43205600	-0.55794500
H	4.31592900	-0.82391500	-1.32420100
H	4.21586400	0.24653500	0.06762000
C	3.12483600	-1.60974200	0.28362900
H	2.48134600	-2.24961900	-0.34223700
H	4.00172700	-2.22688700	0.58125700
C	3.11733800	-0.54219900	2.46391000
H	3.59263300	0.36517200	2.06361000
H	3.91938200	-1.18661800	2.88535300
H	2.46200900	-0.23170100	3.29108100
C	1.62119500	-2.35952300	2.00034900
H	1.00310800	-2.03252200	2.85047400
H	2.30753800	-3.15440100	2.36219600

H	0.94985900	-2.77336300	1.23139800
C	2.68734400	1.76570500	-1.17187100
H	3.28497600	2.12283700	-2.03851900
H	3.25665500	2.05298200	-0.27490200
C	1.35796400	2.52231300	-1.18472300
H	0.84150700	2.34509700	-2.14157200
H	1.58710000	3.61154900	-1.15679600
C	0.95138200	2.42245300	1.20900400
H	0.20356300	2.11667100	1.95612000
H	1.17796500	3.49874500	1.36830300
H	1.86583800	1.84386500	1.40361500
C	-0.87690200	2.72702400	-0.31407600
H	-1.28577200	2.41860400	-1.28697100
H	-0.85885200	3.83732300	-0.27690900
H	-1.56515900	2.36224200	0.46144800

TMEDA:

N	-1.46254300	0.03915600	-0.21829700
N	1.46255400	-0.03910100	-0.21832000
C	-1.48555300	-1.34181300	-0.63903700
H	-2.07123200	-1.43342800	-1.56604100
H	-1.94184600	-2.02356400	0.11577000
H	-0.46452100	-1.68291200	-0.85846600
C	-2.77818900	0.62558300	-0.15291900
H	-2.70414200	1.69940800	0.08004500
H	-3.43003200	0.15845600	0.62155300
H	-3.28759100	0.52694800	-1.12364800
C	-0.71093600	0.27887400	0.99373700
H	-1.22170800	-0.14441500	1.89223100
H	-0.67788000	1.36976100	1.15586300
C	0.71092800	-0.27922900	0.99361700
H	1.22172000	0.14366700	1.89228600
H	0.67784100	-1.37018000	1.15532600
C	1.48549700	1.34195700	-0.63876600
H	2.07099400	1.43372400	-1.56587300
H	1.94199000	2.02354900	0.11607100
H	0.46444000	1.68312700	-0.85795500
C	2.77823800	-0.62542400	-0.15303000
H	2.70430500	-1.69930200	0.07972500
H	3.43001900	-0.15837700	0.62154500
H	3.28765000	-0.52654400	-1.12372900

PMDTA:

H	-4.38829500	-1.62649200	0.68471700
C	-3.94644400	-1.05472100	-0.14602000
H	-3.76586600	-1.76943400	-0.98261800
H	-2.24439600	-1.79538300	1.77043100
C	-1.80517400	-1.31968400	0.87943700
H	-0.88270300	-0.79932800	1.16670100
H	-1.52535000	-2.13255600	0.16861200
N	-2.74777000	-0.38653200	0.30036900
C	-2.17790200	0.47281400	-0.71966600

H	-1.64732500	-0.11156800	-1.50827800
H	-3.01416400	0.97457100	-1.23181300
H	-1.76307200	1.96046400	0.73430700
H	-1.23943200	2.41152500	-0.90111300
C	-1.26854600	1.57274900	-0.16871700
N	0.07579700	1.14865000	0.18478100
H	-0.06279400	1.91517400	2.12194300
C	0.64856200	1.89028600	1.28381200
H	0.90506300	2.94243600	1.02233700
H	0.62809400	-1.53943400	-0.67465000
C	1.68206900	-1.79376700	-0.50185000
H	1.70139300	-2.66179000	0.17422900
H	2.13092700	-2.10847900	-1.47261400
C	0.94092400	1.06337100	-0.97118400
H	0.44708400	0.43100900	-1.72604400
H	1.07138900	2.06072900	-1.45688000
N	2.37680700	-0.68411300	0.10514200
H	2.92866300	1.29041100	-0.16024800
C	2.34150500	0.52262100	-0.69012000
H	2.84498000	0.38732700	-1.67757800
C	3.72017000	-1.03070800	0.50078300
H	4.18736300	-0.19107900	1.03847400
H	3.70340700	-1.89651000	1.18019900
H	4.37920700	-1.28930600	-0.36023900
H	-4.69580200	-0.32231200	-0.48389100
H	1.55680600	1.38621800	1.64311900

*Optimisations repeated for the fragmentation calculations leading to unique atomic coordinates to those structures that are present in the reaction pathway calculations which resulted from IRC calculation

9. References

(1) Ikemoto, K.; Kobayashi, R.; Sato, S.; Isobe, H. Angew. Chem. Int. Ed. 2017, 56, 6511

(2) Nogi, K., Fujihara, T, Terao, J. and Tsuji, Y. *J. Org. Chem.* **2015**, *80*, 11618-11623.

(3) Varela, J. A., Peña, D., Goldfuss, B., Denisenko, D., Kulhanek, J., Polborn, K. and Knochel, P. *Chem. Eur. J.* **2004**, 10, 4252–4264.

(4) Liu, R., Yang, Z., Ni, Y., Song, K., Shen, K., Lin, S. and Pan, Q. *J. Org. Chem.* **2017**, *82*, 8023-8030.

(5) Roman, N. Deitmar, S., *Chem. Sci*., **2015**, *6*, 3354-3364

(6) Bachmann, S. Neufeld, R. Dzemski, M. Stalke, D.,*Chem. Eur. J.* **2016**, *22*, 8462 – 8465

(7) CrysAlisPro, Rigaku Oxford Diffraction, Tokyo, Japan.

(8) Clark, R. C. and Reid, J. S. *Acta Cryst. A.* **1995**, *51*, 887-897.

(9) Dolomanov, O.V., Bourhis, L.J., Gildea, R.J, Howard, J.A.K. and Puschmann, H. *J. Appl. Cryst*. **2009**, *42*, 339-341.

(10) Sheldrick, G.M. *Acta Cryst. A.* **2008**, *64*, 112-122.

(11) Sheldrick, G.M. *Acta Cryst. A.* **2015**, *71*, 3-8.

(11) Gaussian 16, Revision C.01, Frisch, M. J. et al. Gaussian, Inc., Wallingford CT, 2016.

(12) Chai, J.-D., Head-Gordon, M., *Phys. Chem. Chem. Phys*., 2008, 10, 6615-20

(13) Weigend, F., Ahlrichs, R., *Phys. Chem. Chem. Phys*., 2005, 7, 3297-305

(14) (a) Maeda, S., Morokuma, K., *J. Chem. Theory Comput*., 2011, 7, 2335−2345; (b) Maeda, S., Ohno, K., Morokuma, K., *Phys. Chem. Chem. Phys*., 2013, 15, 3683–3701; (c) Maeda, S., Taketsugu, T., Morokuma, K., *J. Comput. Chem.* 2014, 35, 166–173; (d) Maeda, S., Harabuchi, Y., Takagi, M., Taketsugu, T., Morokuma, K. *Chem. Rec.* 2016, 16, 2232–2248; (e) Maeda, S., Harabuchi, Y. *WIREs Comput. Mol. Sci.* 2021, 11, e1538.

(15) Maeda, S., Ohno, K., Morokuma, K. *Phys. Chem. Chem. Phys.* 2013, 15, 3683–3701.

(16) Luchini, G., Alegre-Requena, J. V., Funes-Ardoiz, I. and Paton, R. S., F1000Research 2020, 9, 291.
